# Disassembly of the TRIM56-ATR complex promotes cytoDNA/cGAS/STING axis–dependent intervertebral disc inflammatory degeneration

**DOI:** 10.1172/JCI165140

**Published:** 2024-01-23

**Authors:** Weifeng Zhang, Gaocai Li, Xingyu Zhou, Huaizhen Liang, Bide Tong, Di Wu, Kevin Yang, Yu Song, Bingjin Wang, Zhiwei Liao, Liang Ma, Wencan Ke, Xiaoguang Zhang, Jie Lei, Chunchi Lei, Xiaobo Feng, Kun Wang, Kangcheng Zhao, Cao Yang

**Affiliations:** 1 Department of Orthopaedics, Union Hospital, Tongji Medical College, Huazhong University of Science and Technology, Wuhan, China.; 2 Wuhan Britain-China School, Wuhan, China.

**Keywords:** Bone biology, Bone disease, Orthopedics

## Abstract

As the leading cause of disability worldwide, low back pain (LBP) is recognized as a pivotal socioeconomic challenge to the aging population and is largely attributed to intervertebral disc degeneration (IVDD). Elastic nucleus pulposus (NP) tissue is essential for the maintenance of IVD structural and functional integrity. The accumulation of senescent NP cells with an inflammatory hypersecretory phenotype due to aging and other damaging factors is a distinctive hallmark of IVDD initiation and progression. In this study, we reveal a mechanism of IVDD progression in which aberrant genomic DNA damage promoted NP cell inflammatory senescence via activation of the cyclic GMP-AMP synthase/stimulator of IFN genes (cGAS/STING) axis but not of absent in melanoma 2 (AIM2) inflammasome assembly. Ataxia-telangiectasia–mutated and Rad3-related protein (ATR) deficiency destroyed genomic integrity and led to cytosolic mislocalization of genomic DNA, which acted as a powerful driver of cGAS/STING axis–dependent inflammatory phenotype acquisition during NP cell senescence. Mechanistically, disassembly of the ATR–tripartite motif–containing 56 (ATR-TRIM56) complex with the enzymatic liberation of ubiquitin-specific peptidase 5 (USP5) and TRIM25 drove changes in ATR ubiquitination, with ATR switching from K63- to K48-linked modification, c thereby promoting ubiquitin-proteasome–dependent dynamic instability of ATR protein during NP cell senescence progression. Importantly, an engineered extracellular vesicle–based strategy for delivering ATR-overexpressing plasmid cargo efficiently diminished DNA damage–associated NP cell senescence and substantially mitigated IVDD progression, indicating promising targets and effective approaches to ameliorate the chronic pain and disabling effects of IVDD.

## Abstract 2

A molecule-targeting therapeutic strategy alleviates cellular senescence in nucleus pulposus tissue and mitigates intervertebral disc inflammatory degeneration in mice.

## Graphical abstract

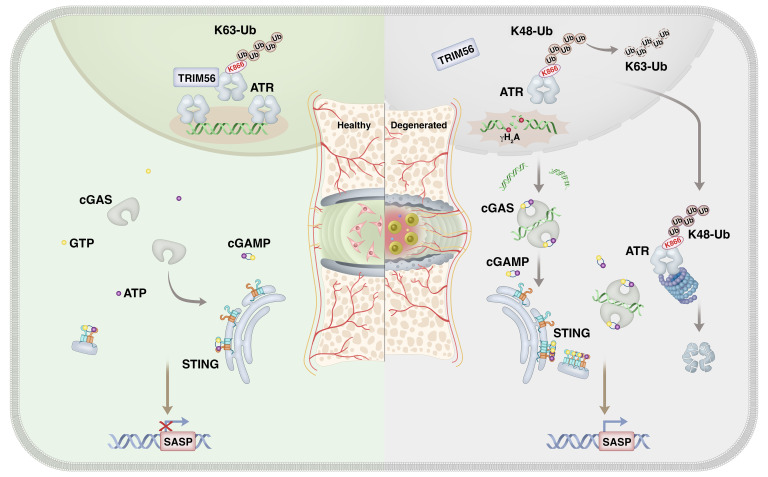

## Introduction

Low back pain (LBP), the leading cause of disability worldwide and the second most common reason for medical consultation in industrialized countries, is the most prevalent and burdensome musculoskeletal disorder (1). Approximately 40% of LBP cases can be attributed to degeneration and herniation of the intervertebral disc (IVD) (2, 3). IVDs are regional and avascular fibrocartilaginous tissues located between 2 adjacent bony vertebrae and are composed of 3 distinct anatomical areas, namely the gelatinous nucleus pulposus (NP), the collagen-rich annulus fibrosus (AF), and the hyaline cartilage endplate (CEP), which are essential for preserving spinal movement and coordinating axial and torsional stress (4, 5). Native NP cells exhibit crucial functions for the regulation of extracellular matrix homeostasis, construction of an accommodating biomechanical environment, and maintenance of the gelatinous property of NP tissue (2, 5, 6). Recently, the accumulation of senescent NP cells due to aging and other damaging factors was determined to be a distinctive hallmark of the initiation and progression of IVD degeneration (IVDD), which leads to impaired cell proliferation and increased catabolic remodeling of the extracellular matrix, thereby driving an enhanced inflammatory response in the IVD tissue and culminating in degenerative biomechanical modifications, discogenic pain, and disability (6–8).

Cell senescence is a permanent and terminal cellular state that is eminently characterized by prolonged and irreversible cell-cycle arrest, a proinflammatory hypersecretory phenotype, macromolecular damage, and dysregulated metabolism, all of which are instigated by triggers, including genotoxic agent exposure, nutrient deprivation, oncogene activation, and organelle stress (9–13). Senescent cells adopt a senescence signaling secretome that includes increased secretion of inflammatory cytokines, angiogenic modulators, and matrix metalloproteinases (MMPs). This secretome is termed the senescence-associated secretory phenotype (SASP), and it remodels the microenvironment to promote inflammation and contributes to impaired tissue integrity, accelerated organismal aging, and chronic age-associated disorder such as osteoporosis, fibrosis, atherosclerosis, Alzheimer’s and Parkinson’s diseases, and so on (9, 12). Accumulated genomic DNA damage and excessive DNA damage signaling activation are recognized as key inducers of senescence initiation and maintenance and are quintessential characteristics of cellular senescence (12). During aging, the excessive genomic DNA damage and damage-associated response activation in quiescent or terminally differentiated cells have inspired the question: What triggers the persistent DNA damage and shifts the cell phenotype to an inflammatory state?

Ataxia-telangiectasia–mutated and Rad3-related protein (ATR) is a multifunctional phosphoinositide 3 kinase–like kinase that is essential for protecting the genomic integrity of mitotic, progenitor, and somatic cells (14, 15). ATR serves as a critical DNA damage response (DDR) regulator when challenged by replication stress, ssDNA breaks, or bulk DNA lesions, which is required to restart stagnant replication, control proliferating cell viability, and protect the quiescent cell from premature activation (14–17). Constitutive deletion of the developmentally essential ATR gene predominantly leads to destructive genomic stability, exhausted tissue renewal capacity, and accelerated premature aging (14, 15, 18). Given that ATR is the ubiquitous master regulator of genomic integrity, the role of ATR in the initiation and progression of cellular inflammatory senescence and aging-related IVDD needs to be further investigated.

In this study, we demonstrate what we believe to be an important finding indicating that during IVDD progression, ATR deficiency destroyed genomic integrity and promoted inflammatory senescence of NP cells through a cytosolic DNA/cyclic GMP-AMP synthase/stimulator of IFN genes (cytoDNA/cGAS/STING) axis–dependent pathway. Mechanically, loss of TRIM56 binding to ATR promoted a shift in ATR ubiquitination from a K63-linked to a K48-linked modification and drove ubiquitin-proteasome–dependent ATR proteolysis. Notably, extracellular vesicles (EVs) are lipid bilayer particles derived from eukaryotic cells with a profound capacity to mediate intracellular communication via cargo delivery, and treatment of ATR-overexpressing, plasmid-containing engineered EVs efficiently alleviated DNA damage–associated NP cell senescence and substantially mitigated IVDD progression.

## Results

### Aberrant genomic DNA damage promotes NP cell senescence during IVDD.

**Figure 1:**
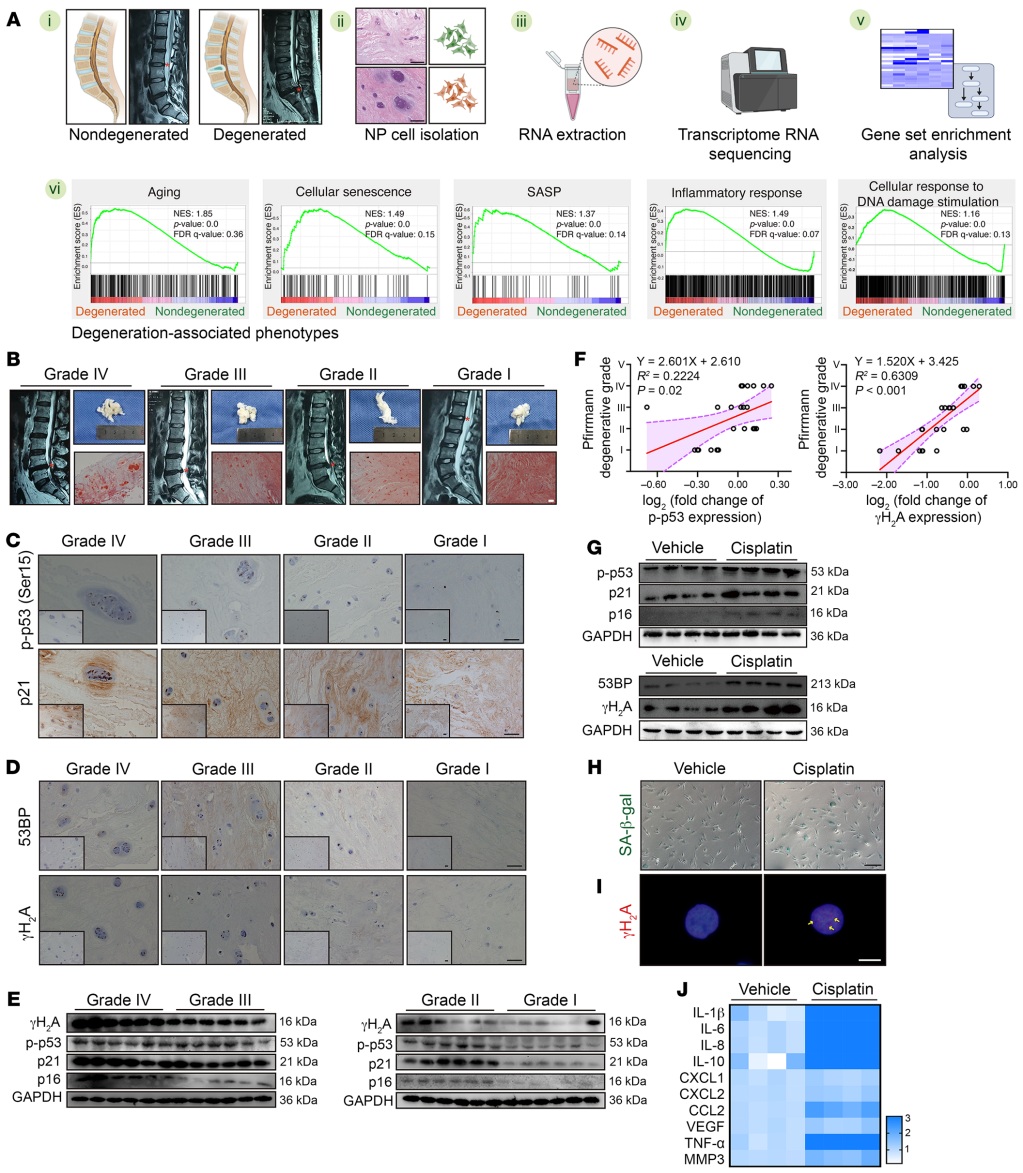
Aberrant genomic DNA damage promotes NP cell senescence during IVDD. (**A**) Schematic workflow showing RNA-Seq and GSEA from human NP tissues (*n* = 6). (**B**) Representative MRIs, general views, and SO&FG staining of human NP tissues. Scale bar: 100 μm. (**C**) IHC staining of p-p53 and p21 in human NP tissues. Scale bars: 100 μm. (**D**) IHC staining of γH_2_A and 53BP in human NP tissues. Scale bars: 100 μm. (**E**) Representative Western blots showing expression of p-p53, p21, p16, and γH_2_A in human NP tissues (*n* = 24). (**F**) Correlation analysis between p-p53/γH_2_A and Pfirrmann degenerative grades (*n* = 24). (**G**) Representative Western blots showing expression of p-p53, p21, p16, γH_2_A, and 53BP in human NP cells treated with 50 μM cisplatin for 24 hours (*n* = 4 biological replicates). (**H**) Representative images of SA–β-gal staining of human NP cells. Scale bar: 100 μm. (**I**) IF staining of γH_2_A foci in human NP cells. Scale bar: 10 μm. (**J**) Heatmap of reverse transcription quantitative PCR (RT-qPCR) analysis showing the SASP in human NP cells (*n* = 4 biological replicates). At least 3 independent experiments were performed. Data are presented as the mean ± SEM. Simple linear regression analysis was performed in **F**.

Transcriptional sequencing (RNA-Seq) of isolated NP cells from human NP tissues and gene set enrichment analysis (GSEA) showed that NP cellular senescence with a hyperactivated inflammatory response and aberrant cellular response to DNA damage stimuli was strongly associated with IVDD phenotypes (Figure 1A). An MRI-based Pfirrmann grading system showed an obviously decreased and heterogeneous T2-weighted hyperintense white signal that was ultimately replaced by a hyperintense black signal, which indicated reduced moisture content with increased fibrosis in NP tissues during IVDD progression (Figure 1B) (19). Safranin O and fast green (SO&FG) staining further confirmed a profound disorganization and calcification of the NP matrix as well as chondroid-like proliferation and clustered cellularity of NP cells occurring in the degenerated NP tissues, especially in those of grade IV group (Figure 1B) (20). Furthermore, the levels of cellular senescence–associated markers were highly increased in degenerated tissues and positively associated with the degenerative levels (9, 11) (Figure 1C). Similarly, IHC staining revealed that several cells carrying markers of cellular genomic DNA damage were easily detected in degenerated NP tissues (9, 11) (Figure 1D). Furthermore, the results of Western blotting revealed that cellular senescence– and DNA damage–associated proteins were abundant, showing a gradient increase during IVDD procession (Figure 1, E and F).

**Figure 2:**
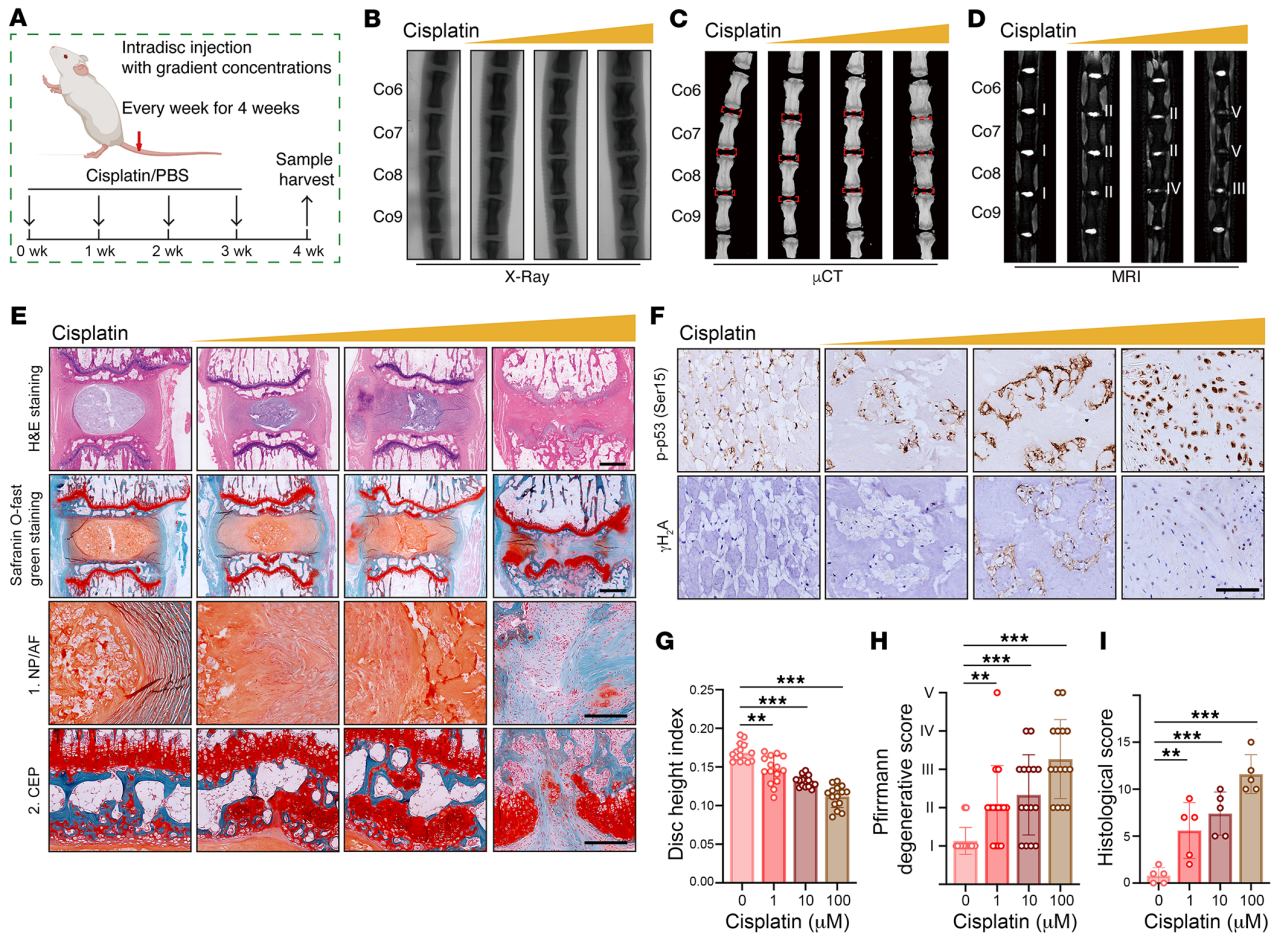
Genomic DNA damage–induced NP cell senescence promotes the degeneration of IVDs in vivo. (**A**) Schematic illustration of the experimental design. (**B**–**D**) Representative x-ray images (**B**), μCT images (**C**), and MRI (**D**) of rat coccygeal IVDs treated with intradisc injection of cisplatin at different concentrations (*n* = 5). (**E**) H&E staining and SO&FG staining of rat coccygeal IVDs. Scale bar: 1 mm. (**F**) IHC staining of p-p53 and γH_2_A in rat coccygeal IVDs. Scale bar: 250 μm. (**G**–**I**) Disc height index (**G**) (*n* = 15 biological replicates), Pfirrmann degenerative grades (**H**) (*n* = 15 biological replicates) and histological score (**I**) (*n* = 5 biological replicates) of rat coccygeal IVDs. Data are presented as the mean ± SEM. At least 3 independent experiments were performed. **P < 0.01 and ***P < 0.001, by 2-way ANOVA (**G**–**I**).

Given that aberrant genomic DNA damage is a key inducer of cellular senescence, NP cells were cultured with the genotoxic agent cisplatin to determine whether pharmacological induction of genomic DNA damage leads to a hyperactivated inflammatory response (9, 11). After cisplatin treatment, NP cells exhibited markedly increased levels of senescence and DNA damage markers (Figure 1G). Increased positive staining of senescence-associated β-galactosidase (SA–β-gal) indicated that cisplatin treatment enhanced the senescence-associated activity of this lysosomal enzyme (9) (Figure 1H). Immunofluorescence (IF) staining showed that NP cells exposed to cisplatin harbored nuclear DNA damage foci (9) (Figure 1I). Moreover, when exposed to an inducer of DNA damage, NP cells secreted a plethora of proinflammatory factors (9, 10) (Figure 1J). To verify that cellular senescence triggered by genomic DNA damage promoted the degeneration of IVDs, cisplatin treatment at gradient concentrations was intradiscally injected into the NP region of coccygeal IVDs of Sprague-Dawley (SD) rats (Figure 2A). Microcomputed tomography (μCT) and x-ray analysis suggested that a high dosage of cisplatin markedly reduced the height of coccygeal IVDs and destructed subchondral bone (Figure 2, B, C, and G). Moreover, the T2-weighted MRI signal was decreased and showed greater inhomogeneity with increased concentrations of cisplatin administration, which meant that the relatively low dosage of cisplatin initiated the degeneration of coccygeal IVDs (Figure 2, D and H). H&E staining and SO&FG staining of coccygeal IVD sections revealed NP tissue atrophy, loss of a clear boundary between the NP and AF, as well as disorganization of the NP and AF matrix. Furthermore, a high dosage of cisplatin destructed the integrity of the coccygeal IVD, causing IVD fibrosis and CEP rupture (Figure 2, E and I). The number of phosphorylated p53^+^ (p-p53^+^) and γH_2_A^+^ cells was obviously increased with gradient dosages of cisplatin treatment, which positively correlated with the degenerative degrees (Figure 2F). Collectively, the data indicated that aberrant genomic DNA damage caused NP cellular senescence and progressive IVDD.

### CytoDNA triggers NP cell inflammatory senescence via cGAS/STING axis sensing but not AIM2 inflammasome activation.

**Figure 3:**
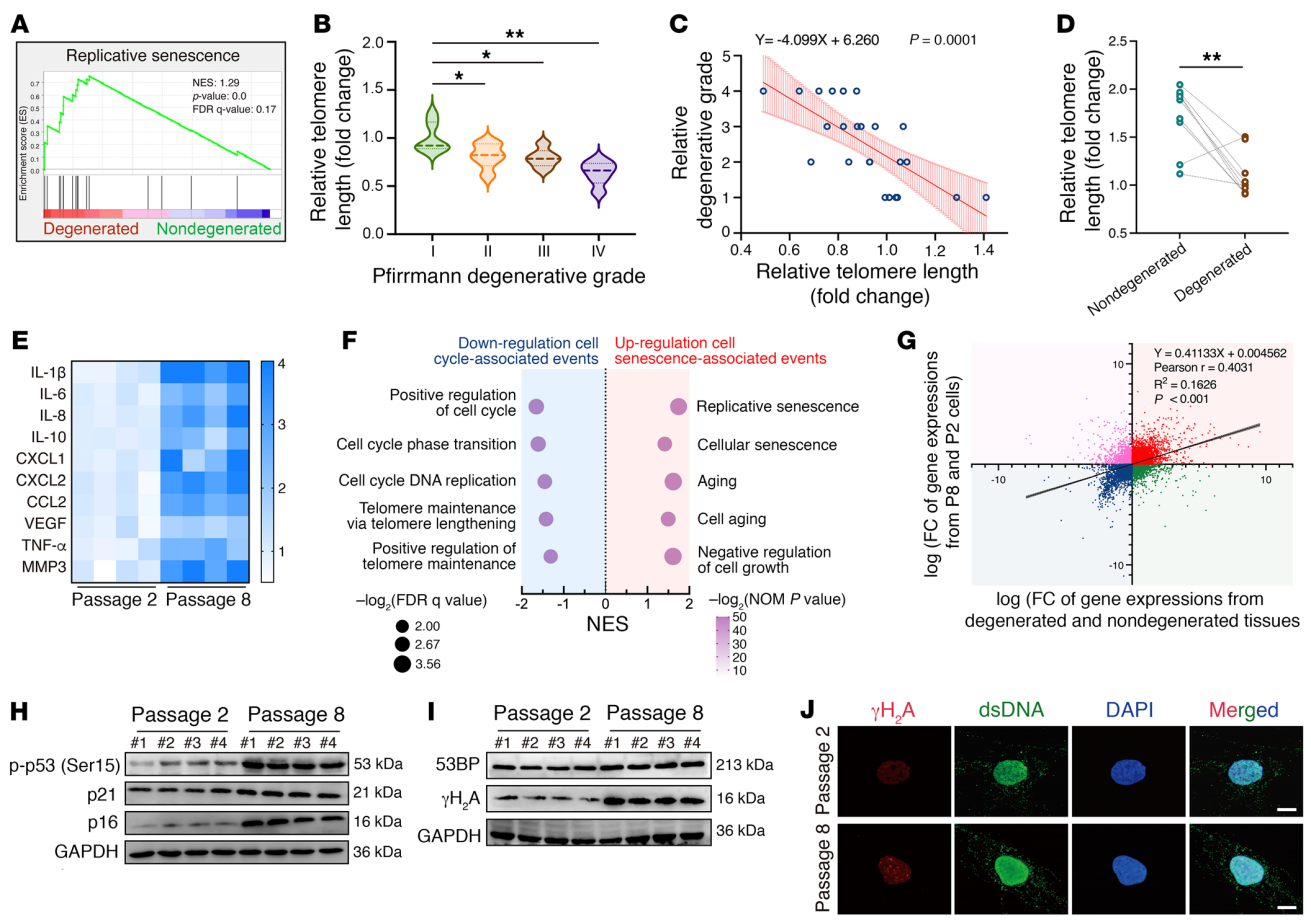
Replicative senescence triggers the inflammatory senescence phenotype acquisition of NP cells during IVDD progression. (**A**) GSEA showing enrichment of “replicative senescence” in degenerated NP tissues. (**B**) Relative telomere lengths of isolated NP cells from human NP tissues with different Pfirrmann degenerative grades (*n* = 24). (**C**) Correlation analysis between relative telomere length and Pfirrmann degenerative grades (*n* = 24). (**D**) Relative telomere lengths from age- and sex-matched case pairs of NP tissues (*n* = 20). (**E**) Differential heatmap of the SASP in NP cells (*n* = 4 biological replicates). (**F**) Change pathways of transcriptional profiles enriched in P8 NP cells compared with P2 NP cells (*n* = 3 biological replicates). (**G**) Correlation analysis of differential transcriptional profiles in cultured NP cells (*y* axis) compared with those in isolated NP cells from human NP tissues (*x* axis). (**H**) Representative Western blots of p-p53, p21, and p16 levels in NP cells (*n* = 4 biological replicates). (**I**) Representative Western blots of γH_2_A, and 53BP levels in NP cells (*n* = 4 biological replicates). (**J**) IF staining of γH_2_A foci in NP cells. Scale bars: 10 μm. Data are presented as the median ± IQR or the mean ± SEM. At least 3 independent experiments were performed. *P < 0.05, **P < 0.01, by permutation test (**A**), rank-sum test (**B**), paired Student’s *t* test (**D**), and simple linear regression (**C** and **G**).

Gene expression profiling showed that replicative senescence was significantly enriched in degenerated NP tissues (Figure 3A). Previous studies have suggested that replicative senescence, which is characterized by irreversible loss of the proliferative potential of human somatic cells and caused by the shortening of telomeres accumulated in somatic cells undergoing repeated cell divisions, may contribute to a higher percentage of senescent cells in NP cell clusters and trigger a cell-mediated response to induce progressive destruction of NP integrity and accelerated IVD aging (21, 22). The analysis of genomic DNA from nondegenerated and degenerated NP cells showed that the relative telomere length progressively decreased with increased degeneration severity, and, unexpectedly, relative telomere lengths were negatively associated with age as well, as age is regarded as a conclusive etiological factor of IVDD (6, 23, 24) (Figure 3, B and C, and Supplemental Figure 1, A and B; supplemental material available online with this article; https://doi.org/10.1172/JCI165140DS1). Furthermore, age- and sex-matched case pairs demonstrated that in degenerated NP tissues, the telomere lengths were dramatically shortened, excluding the confounding effects of age, suggesting that the replicative senescence of NP cells accompanied by a shortened telomere length is a characteristic of IVDD (Figure 3D). To further explore the molecular mechanism and the damaged effect of NP cell replicative senescence during progressive IVDD, we established a replicative senescence model of NP cells via continuous passage culturing, and the relative telomere length was shortened throughout the serial passages (22, 25) (Supplemental Figure 1C). We identified a higher incidence of multidimensional senescence-like phenotypes in the eighth-generation cells (P8) compared with those in second-generation cells (P2), including increased cell-cycle arrest–associated marker levels, enhanced enzymatic SA–β-gal activity, a morphological shift, and HP1γ-containing senescence-associated heterochromatin foci (SAHFs) formation (26, 27) (Figure 3, E and H, and Supplemental Figure 1, D and E). Transcriptional sequencing profiling showed significantly decreased cell-cycle–associated events in P8 NP cells, accompanied by upregulated senescence-associated events, and the significant genetic changes in P8 cells compared with P2 cells were similar to those observed in human degenerated NP tissues compared with human nondegenerated NP tissues (Figure 3, F and G). We also found that the P8 cells harbored an inflammatory state, and those NP cells lost the capacity to maintain tissue homeostasis accompanied by an extracellular matrix catabolic imbalance and degenerative microenvironment formation with osteogenic, angiogenic, and neurogenic potentials (Figure 3E and Supplemental Figure 1, F and G). Additionally, the expression levels of γH_2_A and 53BP were drastically increased in the P8 cells (Figure 3, I and J).

**Figure 4:**
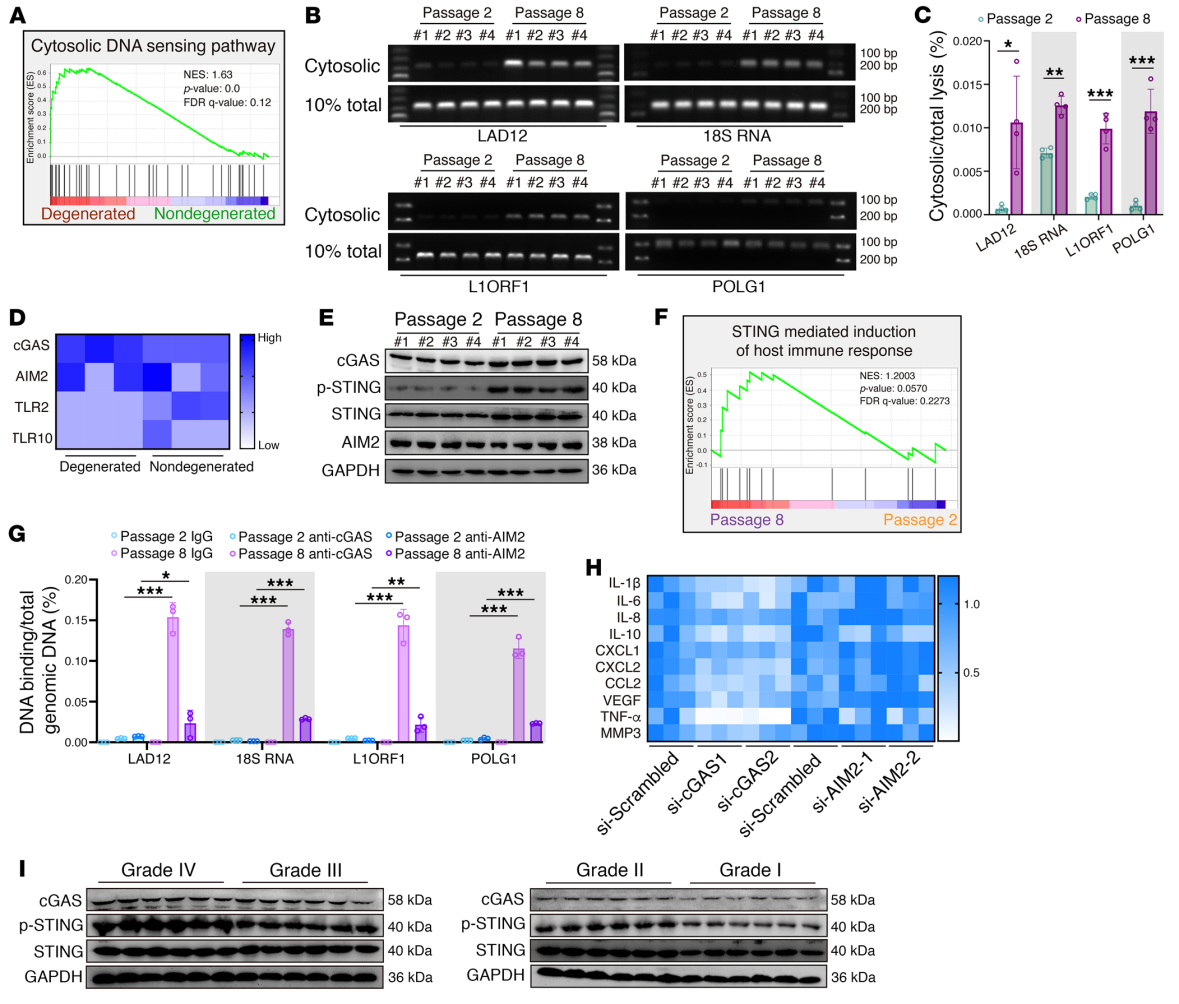
CytoDNA acts as the NP cell inflammatory senescence trigger via cGAS/STING axis sensing, not AIM2 inflammasome activation. (**A**) GSEA showing enrichment of the “cytosolic DNA sensing pathway” in degenerated NP tissues. (**B** and **C**) Representative DNA electrophoresis images and the ratio of cytosolic genomic DNA to total genomic DNA in NP cells (*n* = 4 biological replicates). (**D**) Differential expression heatmap showing cytosolic DNA sensors from RNA-Seq of human NP tissues (*n* = 6). (**E**) Representative Western blots showing cGAS, STING, p-STING, and AIM2 levels in NP cells (*n* = 4 biological replicates). (**F**) GSEA showing enrichment of “STING-mediated induction of the host immune response” in senescent P8 NP cells. (**G**) DNA IP and the quantitative ratio of genomic DNA binding with cGAS or AIM2 to total genomic DNA in NP cells (*n* = 3 biological replicates). (**H**) Differential heatmap showing the SASP in senescent P8 NP cells after treatment with si-cGAS and si-AIM2 (*n* = 3 biological replicates). (**I**) Representative Western blots showing cGAS and STING levels in human NP samples (*n* = 24). Data are presented as the mean ± SEM. *P < 0.05, **P < 0.01, and ***P < 0.001, by permutation test (**A** and **F**), paired Student’s *t* test (**C**), and 2 way ANOVA (**G**).

Upstream signals that trigger SASP acquisition are complex and multifactorial, varying according to the senescence inducer (9). Gene set enrichment analysis (GSEA) of RNA-Seq revealed that a cytoDNA-sensing pathway was aberrantly activated during degenerative progression (Figure 4A). Mislocalized cytoDNA derived from the host genome, which is normally confined to the nucleus or mitochondria, is a powerful trigger for activation of the inflammatory response via DNA-sensing–associated pattern recognition receptors (PRRs) (10, 11, 28). Cytoplasmic chromatin fragments (CCFs) due to nuclear envelope blebbing or the nucleus-cytoplasm export process are hallmarks of persistent genomic instability in senescent cells (9, 10). Although not yet proved, specific genomic regions, such as lamin-associated domains and repetitive DNA, are thought to contribute to CCF formation and a cytoDNA-sensing–dependent inflammatory response (11, 29–31). To further examine whether aberrant genomic DNA is mislocalized to the cytoplasm of senescent NP cells, we isolated the cytosolic fraction, which lacked cross-contamination from P2 and P8 NP cells, and found marked enrichment of genomic DNA, such as open reading frame 1 of L1 (L1ORF1) and lamin-associated domain of chromosome 12 (LAD12) in the cytosolic compartments of P8 cells when compared with the levels in P2 cells (29, 30, 32) (Figure 4, B and C, and Supplemental Figure 1H). Interestingly, some extranuclear DNA domains in addition to lamin-associated domains and repetitive DNA were also evident in the cytoplasm (32, 33) (Figure 4, B and C). CytoDNA sensors, including cGAS, absent in melanoma 2 (AIM2), TLR2, and TLR10, are primarily activated by mislocalized endogenous cytoDNA and essentially drive the cell-autonomous response (10). Additionally, the transcriptional levels of cGAS markedly increased, and those of AIM2, TLR2, and TLR10 were insignificantly varied or aberrantly decreased in degenerated NP tissues (Figure 4D). Analysis of master influential protein expression levels in senescent NP cells revealed that the levels of cGAS, STING, and activated STING (p-STING), but not of AIM2, were drastically increased in the P8 NP cells (Figure 4E). GSEA similarly showed that the “cytosolic DNA–sensing pathway” and “STING-mediated induction of host immune response,” rather than the “inflammasome,” were obviously enriched in senescent P8 NP cells (Figure 4F and Supplemental Figure 1, I and J). The AIM2 inflammasome, which is composed of AIM2, ASC, and pro–caspase 1, is a protein complex that acts as a platform for pro–caspase 1 cleavage, which initiates caspase 1 enzyme activity (34–36). The assembly of the AIM2 inflammasome is essentially for the cytoDNA-mediated inflammatory response via AIM2 sensing, and co-IP showed no significant enrichment of ASC or pro–caspase 1 in the AIM2-bound precipitate, indicating that senescent NP cells acquired the SASP independent of AIM2 activation (Supplemental Figure 1K). More important, genomic DNA sequences were highly enriched with cGAS but not AIM2 precipitants from senescent NP cells (32, 37) (Figure 4G and Supplemental Figure 1L). Genetic knockdown via siRNA treatment verified that cGAS knockdown reversed the inflammatory hypersecretory state but exerted no influence on persistent genomic DNA damage and cell-cycle arrest–associated protein levels in P8 cells (Figure 4H and Supplemental Figure 1, M–O). Additionally, AIM2 knockdown did not affect the senescence phenotype acquisition of P8 cells (Figure 4H and Supplemental Figure 1, M–O). Moreover, the genotoxic agent increased the expression of cGAS and STING, which suggested that cGAS/STING axis activation was involved in the genomic DNA damage effect but not its initiation (Supplemental Figure 1P). Furthermore, the expression of cGAS and STING was upregulated and positively associated with the severity degree of IVDD (Figure 4I). Collectively, the data indicated that, due to replicative senescence–induced genomic instability, cytoDNA acted as the trigger of the NP cell inflammatory response mediated via cGAS/STING axis–induced sensing, not AIM2 inflammasome activation.

### cGAS/STING axis activation drives inflammatory phenotype acquisition of senescent NP cells via p65-mediated transcriptional modulation.

**Figure 5:**
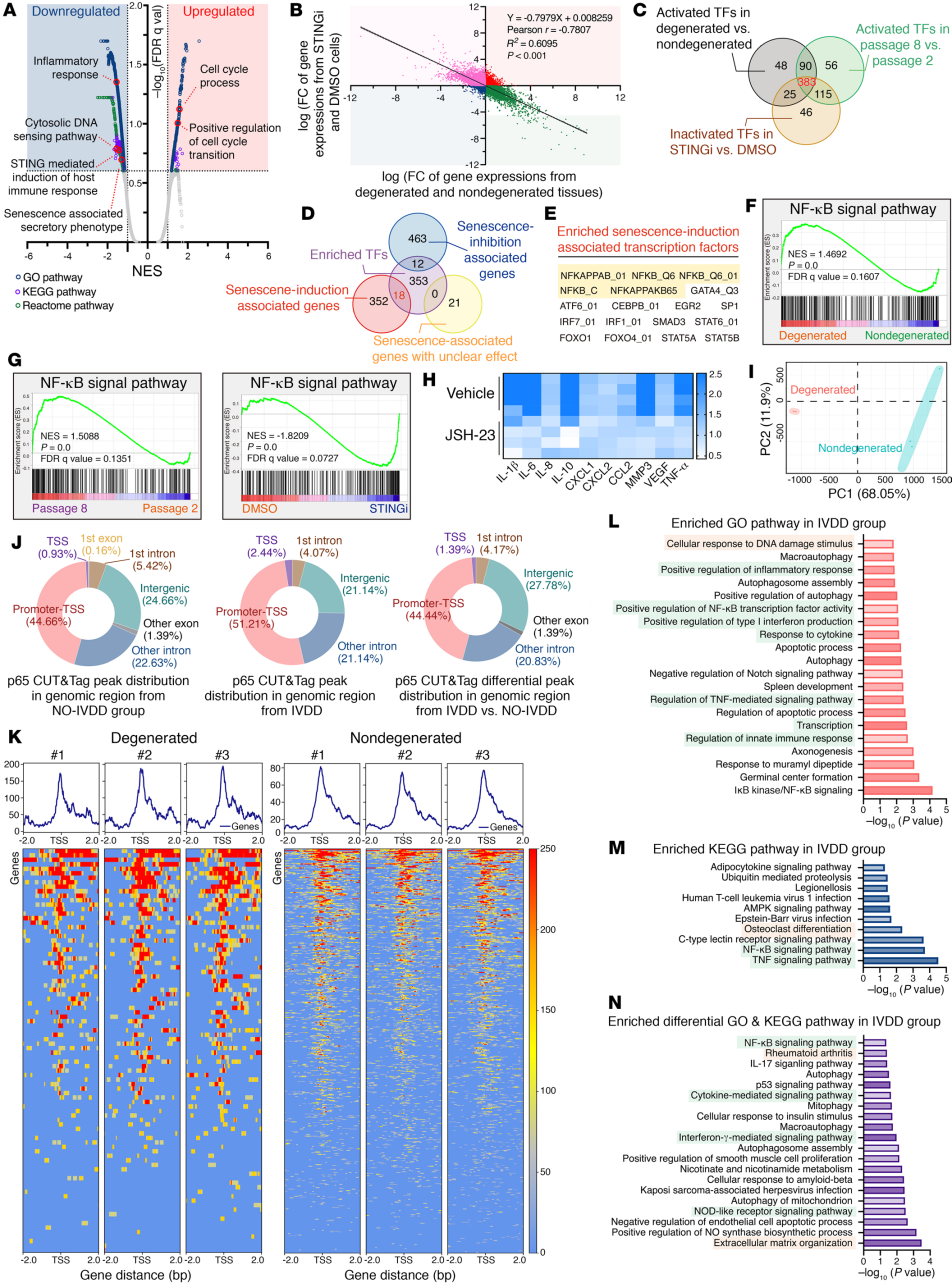
cGAS/STING axis activation drives inflammatory phenotype acquisition of senescent NP cells via p65-mediated transcriptional modulation. (**A**) Volcano plot showing enriched differential pathways of transcriptional profiles in senescent NP cells after treatment with H-151 (1 μM for 24 h) (*n* = 3 biological replicates). (**B**) Correlation analysis of differential transcriptional profiles in senescent NP cells treated with H-151 or DMSO (*y* axis) compared with those in isolated NP cells from human NP tissues (*x* axis). Simple linear regression was performed to determine significance. (**C**) Venn diagram showing overlapping TFs involved in the degenerative and senescent processes. (**D** and **E**) Venn diagram (**D**) and table (**E**) showing overlapping senescence induction–associated TFs based on the integrated analysis of enriched TFs and senescence-associated genes from the CellAge database. (**F**) GSEA-enriched “NF-κB signal” pathways in degenerated NP tissues. (**G**) GSEA-enriched “NF-κB signal pathways” in senescent NP cells and STING inhibitor–pretreated senescent NP cells. (**H**) Differential heatmap of the SASP in senescent P8 NP cells after treatment with the NF-κB inhibitor JSH-23 (10 μM for 24 h) (*n* = 4 biological replicates). (**I**) PCA plot showing clustering of p65-bound peaks in human NP tissues via CUT&Tag-Seq analysis (*n* = 6). (**J**) Distribution of p65-bound genomic regions. (**K**) Heatmap showing p65-binding peaks in human NP tissues (*n* = 6). (**L**) GO analysis of p65-bound peak annotations. (**M**) KEGG analysis of p65-bound peak annotations. (**N**) Differentially enriched GO and KEGG pathways upregulated in degenerated NP tissues.

GSEA of transcriptional profiles revealed that the STING pharmacological inhibitor H-151 notably suppressed the inflammatory response formation and SASP acquisition of senescent cells (38) (Figure 5A). Furthermore, the genomic changes in P8 NP cells after STING inhibitor administration were inversely associated with the transcriptional changes in human disc degeneration (Figure 5B). Mechanistically, to identify essential transcript factor(s) promoting inflammatory phenotype acquisition, we integrated multidimensional transcriptomic analysis and subsequently found that 383 activated transcript factors (TFs) or transcript-associated pathways were enriched in degenerated tissues and senescent P8 cells, as well as suppressed in P8 cells after STING inhibitor treatment (Figure 5C). The integrated analysis with senescence-associated genes revealed that the NF-κB signaling pathway and the NF-κB–associated TF p65 were the most highly enriched senescence induction–associated TFs, ranked according to the number of activated TFs in IVDD progression (Figure 5, D and E). Additionally, the NF-κB signaling pathway was aberrantly activated during the degenerative progression of IVD, and administration of the NF-κB inhibitor JSH-23 could block hyperactivated secretion of inflammatory messages from senescent P8 cells (Figure 5, F–H). Cleavage under targets and tagmentation sequencing (CUT&Tag-Seq) in NP cells from human NP tissues was performed to further verify the critical role of p65 in regulating inflammatory phenotype acquisition on the genomic scale. Principle component analysis (PCA) confirmed that degenerated NP cells clustered apart from the corresponding nondegenerated NP cells (Figure 5I). When we further evaluated the differential genomic regions occupied by p65, we found, interestingly, that enriched the p65-binding peak markedly increased at the transcription start site (TSS), which suggested the transcriptional remodeling of degenerative NP cells during IVDD progression (Figure 5, J and K). Kyoto Encyclopedia of Genes and Genomes (KEGG) analysis and Gene Ontology (GO) analysis revealed that the p65-bound peak annotation was involved in inflammatory response activation and a degenerative phenotype such as osteoblast differentiation (Figure 5, L and M). To study the role of the cGAS/STING axis in promoting an inflammatory response, we examined whether a STING pharmacological inhibitor could suppress p65-mediated transcriptional remodeling and inflammatory phenotype acquisition. To this end, we utilized CUT&Tag-Seq to test p65-bound peaks in senescent P8 NP cells after STING inhibition. Notably, PCA showed that p65-bound peaks in P8 cells after treatment with H-151 were clearly distinct and clustered separately (Supplemental Figure 2A). Predictably, genomic regions of p65-bound peaks were mainly distributed in promoter TSS (Supplemental Figure 2B). In addition, STING blocking suppressed p65 binding at the TSS and reconstructed the transcriptional state of senescent NP cells, and this was accompanied by increased cell division– and DNA repair–associated events and decreased inflammatory response activation (Supplemental Figure 2, C–E).

Furthermore, the increased differential p65-bound peak in degenerated NP cells was associated with genes involved in inflammatory sensing, cellular response, and musculoskeletal inflammatory disorders such as rheumatoid arthritis (Figure 5N). Surprisingly, genes whose promoter TSS increased the differential p65-bound peak in degenerated NP cells were enriched in the NOD-like receptor (NLR) signaling pathway. NLRP3 presents a typical NLR that senses exogenous pathogenic patterns and endogenous damaged signals, resulting in the assembly and activation of the NLRP3 inflammasome (39). Our previous study revealed that cytosolic escape of mitochondrial DNA and opening of mitochondrial permeability transition pores via the cGAS/STING axis increased the expression of NLRP3 inflammasome–associated components and subsequently promoted activation of the NLRP3 inflammasome, which contributed to the inflammatory death of NP cells (40). Interestingly, GSEA showed no differential enrichment of the “NLRP3 inflammasome” in senescent P8 NP cells, and co-IP experiments revealed that no markedly differential assembly of the NLRP3 inflammasome in NP cells with different passages (Supplemental Figure 3, A and B). Visually, transmission electron microscope (TEM) showed that senescent NP cells acquired senescence-like phenotypes including loss of long, shuttle–shaped morphology accompanied by swelling, high-electron gathering in the cellular nucleus, and a thin high-electron region of the nuclear membrane, whereas the mitochondria did not show swelling or vacuolization, and cellular membranes maintained their structural integrity without large bubbles, which was different from what was observed under oxidative stress (9, 40) (Supplemental Figure 2C). Surprisingly, the expression levels of NLRP3 and ASC were obviously increased in senescent cells (Supplemental Figure 3D).

Mechanistically, NLRP3 inflammasome–mediated inflammatory response activation is considered to be a 2-step process of priming and activation. Priming serves an important function in upregulating the expression of inflammasome components, and NF-κB activation–mediated gene expression remodeling is responsive to transcriptional upregulation of NLRP3 upon cell sensing of damage-associated signals (39). Given that p65 acted as a core TF of the NF-κB signal pathway in cGAS/STING axis–mediated inflammatory regulation, we inferred that p65 might play a critical role in the upregulated expression of the NLRP3 inflammasome during NP cell senescence. CUT&Tag-Seq technology revealed that STING inhibition could suppress the binding of p65 to the TSSs of NLRP3, ASC, pro–IL-1, and pro–caspase 1, with no obvious increase in the binding of P65 to the TSS of GSDMD (Supplemental Figure 3E). Additionally, to further explore the effect of the NLRP3 inflammasome on senescent NP cells under damaging stimulation, we cultured P2 and P8 NP cells with Tert-butyl hydroperoxide (TBHP) at concentration gradients and found that a low concentration of TBHP could markedly promote the assembly of the NLRP3 inflammasome and upregulation of NLRP3 inflammasome effectors including cleaved caspase 1 and cleaved GSDMD, suggesting that senescent NP cells harbored in an inflammatory sensitive state was accompanied by increased damaged sensors via cGAS/STING axis–mediated facilitation of damaged stimulation (Supplemental Figure 3, F and G). Collectively, these results indicated that during NP cell senescence progression, cGAS/STING axis activation drove inflammatory phenotype acquisition and inflammatory hypersensitivity to damaged signals via p65-mediated transcriptional remodeling.

### ATR deficiency promotes genomic instability and cGAS/STING axis–dependent inflammatory senescence of NP cells.

**Figure 6:**
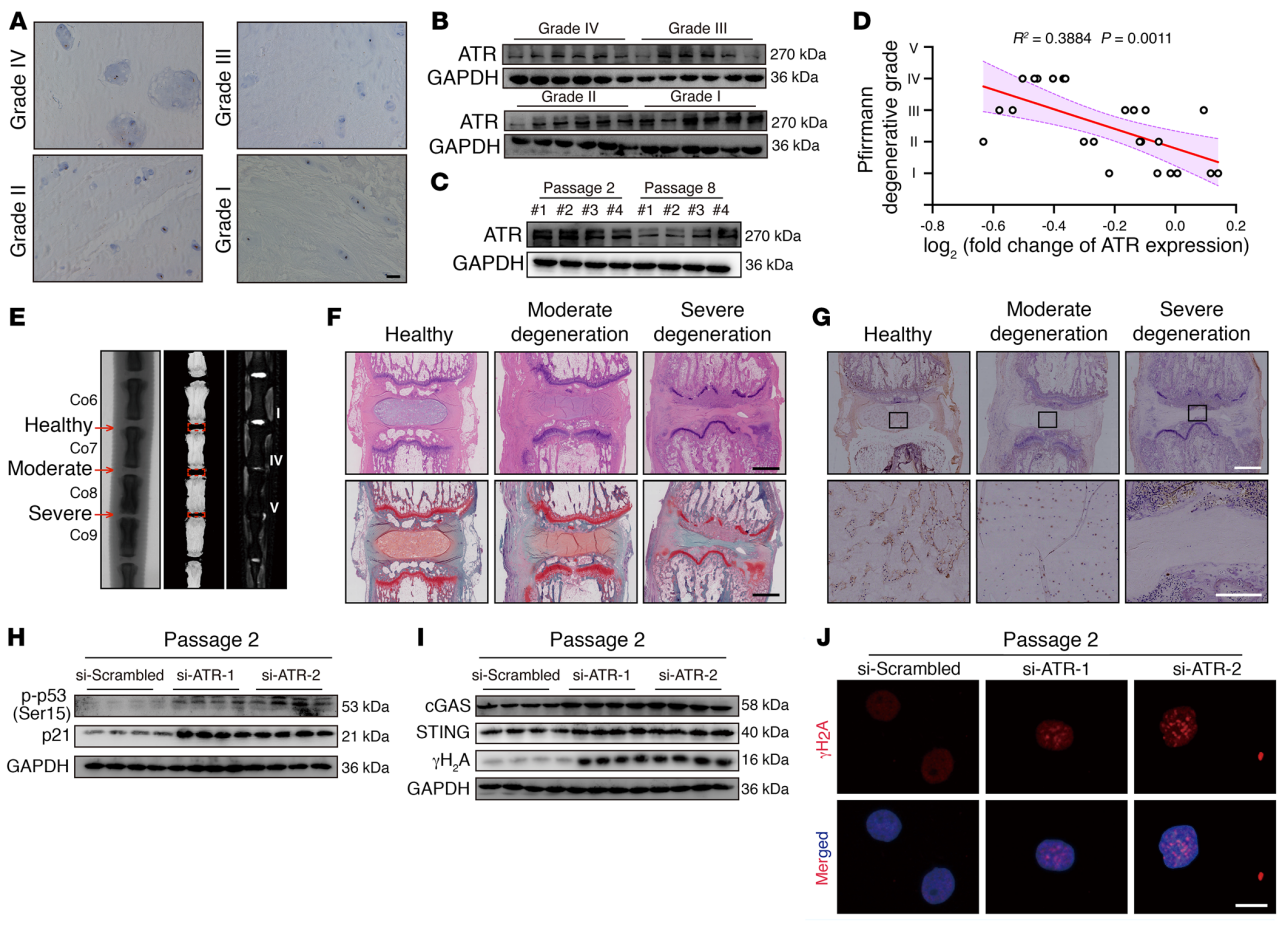
ATR deficiency promotes genomic instability and cGAS/STING axis–dependent inflammatory senescence of NP cells during IVDD progression. (**A**) IHC staining of ATR in human NP tissues. Scale bars: 100 μm. (**B**) Representative Western blots of ATR expression in human NP tissues (*n* = 24). (**C**) Representative Western blots of ATR expression in NP cells (*n* = 3 biological replicates). (**D**) Correlation analysis between ATR expression and Pfirrmann degenerative grades (*n* = 24). (**E**) Representative x-ray images, μCT images, and MRIs of rat coccygeal IVDs. (**F**) H&E staining and SO&FG staining of rat coccygeal IVDs. Scale bars: 1 mm. (**G**) IHC staining of ATR in rat coccygeal IVDs. Scale bars: 250 μm. (**H**) Representative Western blots showing p-p53 and p21 levels in P2 NP cells transfected with si-ATR (*n* = 4 biological replicates). (**I**) Representative Western blots showing cGAS, STING, and γH_2_A levels in P2 NP cells (*n* = 4 biological replicates). (**J**) IF staining of γH_2_A foci in P2 NP cells. Scale bar: 10 μm.

ATR is a master regulator essential for regulating the genomic integrity of numerous cells, modulating nuclear envelope plasticity and preventing the acquisition of premature age-associated phenotypes (14, 15). IHC staining showed that ATR^+^ cells were clearly lost in degenerated NP tissues (Figure 6A). Additionally, ATR expression levels were reduced and inversely associated with the severity of degenerative and senescent progression (Figure 6, B–D). In a surgical needle puncture–induced rat coccygeal IVDD model, radiographic analysis revealed a decline in the coccygeal IVD height, a destruction of subchondral bone with loss of homogeneous T2-weighted signal in the severe IVDD group, and only inhomogeneous T2-weighted signals in the moderate IVDD group (Figure 6E). H&E and SO&FG staining confirmed that IVD tissues with greater degeneration exhibited more severe fibrosis, greater matrix disorganization, and narrower NP regions (Figure 6F). Importantly, IHC staining showed that the number of ATR^+^ cells markedly decreased with increased degeneration progression (Figure 6G). Collectively, these results indicated that ATR might be involved in the maintenance of NP cell genomic integrity.

Knockdown of ATR expression promoted the acquisition of a senescence-like phenotype by normal NP cells (Figure 6H and Supplemental Figure 4, A–C). Furthermore, ATR silencing increased genomic DNA damage and drove activation of the cGAS/STING axis (Figure 6, I and J). Similarly, ATR deficiency aggravated genomic DNA damage and cellular senescence in serial passage– or genotoxic agent–induced senescent NP cells (Supplemental Figure 4, D and E). In contrast, ATR overexpression in senescent NP cells abolished genomic DNA damage stimulation and rescued the senescence phenotype (Supplemental Figure 4, F–M). Surprisingly, we observed no significant changes in the expression of senescence-associated markers or genomic damage markers in normal NP cells after ATR overexpression (Supplemental Figure 4N). Collectively, gain- and loss-of-function assays demonstrated that ATR was crucial for maintaining genomic stability and preventing cGAS/STING axis–dependent senescence of NP cells.

### ATR interactomic analysis reveals that the ubiquitylation shift from K63-linked to K48-linked modification contributes to ATR deficiency in senescent NP cells.

**Figure 7:**
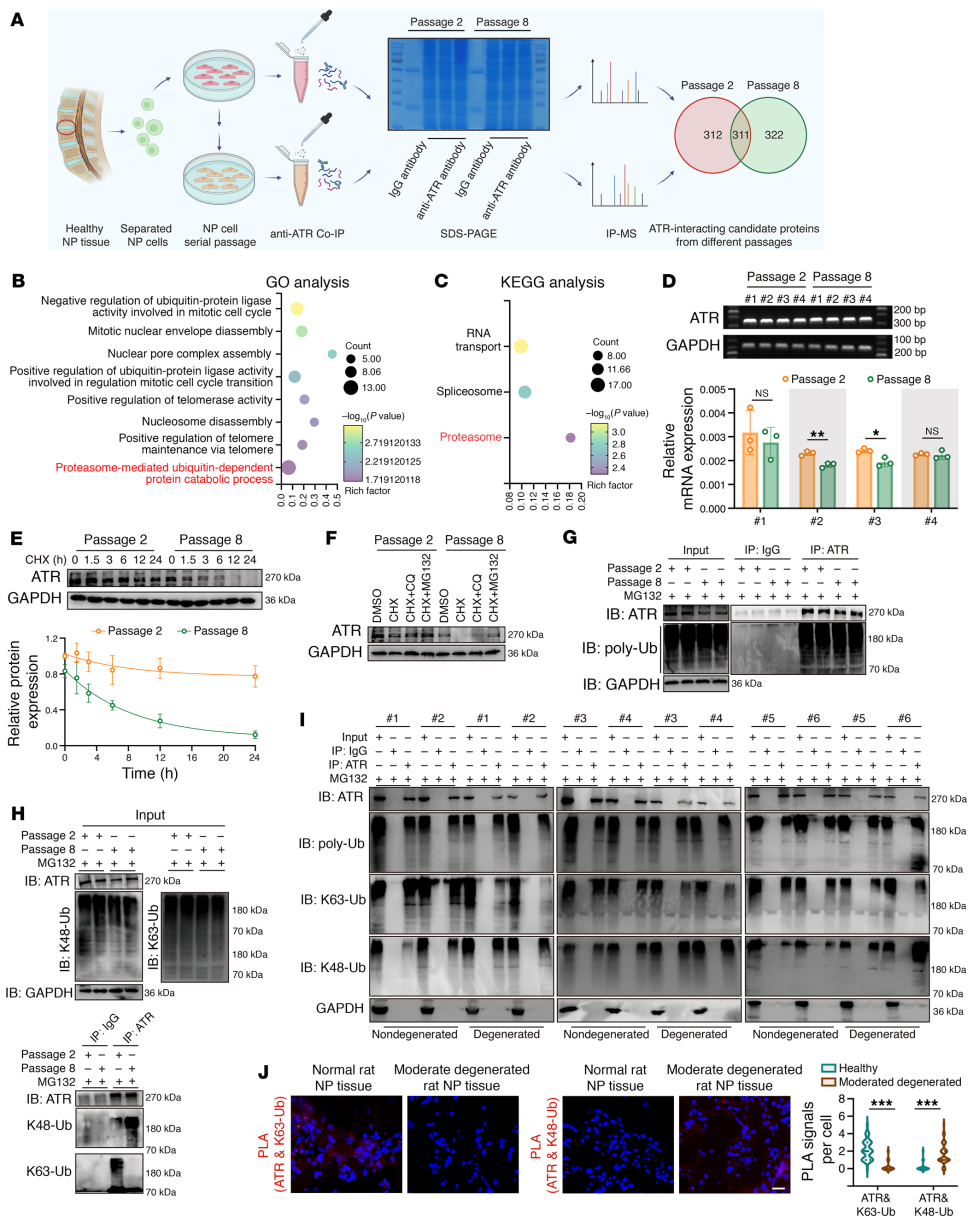
Ubiquitylation shift from K63-linked to K48-linked modification and ubiquitylation proteolysis–mediated ATR dynamic instability contribute to ATR deficiency in senescent NP cells. (**A**) Schematic workflow showing IP-MS performed to identify ATR-interacting proteins in NP cells (*n* = 3 biological replicates). (**B** and **C**) KEGG (**B**) and GO (**C**) analysis showing the differential ATR interactome enriched in senescent NP cells. (**D**) Relative mRNA levels of ATR in NP cells (*n* = 3 biological replicates). (**E**) Half-life analysis of endogenous ATR protein in NP cells treated with 100 μg/mL cycloheximide (CHX) at different time points (*n* = 3 biological replicates). (**F**) Representative Western blots showing ATR expression in NP cells treated with 100 μg/mL CHX and 10 μM MG132 or 50 μM CQ for 12 hours (*n* = 3 biological replicates). (**G**) Endogenous ubiquitination of ATR in NP cells after treatment with 10 μM MG132 for 6 hours (*n* = 2 biological replicates). (**H**) Endogenous K48-linked and K63-linked ubiquitination of ATR in NP cells after treatment with 10 μM MG132 for 6 hours (*n* = 2 biological replicates). (**I**) K48-linked and K63-linked ubiquitination of ATR in human sample pairs (*n* = 12). (**J**) Representative PLA images and quantitative analysis of rat coccygeal IVDs (*n* = 50 cells per condition). Scale bar: 20 μm. Data are presented as the mean ± SEM or the median ± IQR. At least 3 independent experiments were performed. *P < 0.05, **P < 0.01, and ***P < 0.001, by unpaired Student’s *t* test (**D**) and rank-sum test (**J**). IB, immunoblot.

Endogenous co-IP and the mass spectrometry (MS) analysis of proteins pulled down were performed to further investigate the potential mechanism that mediated the loss of ATR in degenerative progression of IVDs (Figure 7A). Overall, we identified 312 differential candidate proteins in normal cells and 322 in senescent cells (Figure 7A). KEGG pathway analysis of these differential candidate proteins from senescent cells revealed that the proteasome served as the significantly enriched biological process, and GO analysis similarly indicated that these differential proteins were markedly enriched in the proteasome-mediated, ubiquitin-dependent protein catabolic process (Figure 7, B and C).

Interestingly, we found no obvious transcriptional changes in ATR expression during the senescence process (Figure 7D). Furthermore, a cycloheximide (CHX) chase experiment showed that this pharmacological blockade of de novo protein synthesis contributed to the time-dependent loss of ATR protein and a shortened half-life of endogenous ATR protein in senescent NP cells, which meant that ATR loss during the senescence process was dependent on a posttranslational modification (Figure 7E). The ubiquitin-proteasome system and the autophagosome/lysosome pathway are 2 principal proteolytic pathways for maintaining proteostasis. Importantly, the proteasome inhibitor MG132, but not chloroquine (CQ), the pharmacological inhibitor of lysosomal activity, prevented CHX-induced loss of ATR protein in senescent NP cells (Figure 7F). Surprisingly, an endogenous co-IP assay showed that the ubiquitylation of ATR in senescent NP cells was not markedly elevated (Figure 7G). Cellular ubiquitin ligases (E3s) catalyze the covalent attachment of different polymeric ubiquitin chains to substrates, coupling to the distinct biological processes (41). The ubiquitin chain linked via K48-branched chains can be sensed by the proteasome, which triggers proteasome-dependent protein degradation, whereas chains linked via K63 are stabilized to the protein structure to prevent aberrant proteolysis and regulate the assembly of signaling complexes (41). More important, we found that ATR was more heavily modified via K63-linked ubiquitylated chains in normal NP cells, unlike K48-linked ubiquitylation in senescent NP cells, indicating a ubiquitinated modification shift from K63- to K48-linked ubiquitination (Figure 7H). Furthermore, co-IP analysis experimentally verified increased K48-linked ubiquitination accompanied by decreased K63-linked ubiquitinated modification of ATR in degenerated NP cells from human sample pairs. Proximity ligation assay (PLA) staining showed a notable loss of fluorescent signals between ATR and K63-linked ubiquitination with the presence of binding signals between ATR and K48-linked ubiquitination in degenerated rat coccygeal NP tissues, indicating a physical interaction shift of ATR and different topological polymeric ubiquitin chains during the degenerated progression of IVDs (Figure 7, I and J).

### Loss of interaction with TRIM56 drives the ATR ubiquitination shift during NP cell senescence progression.

**Figure 8:**
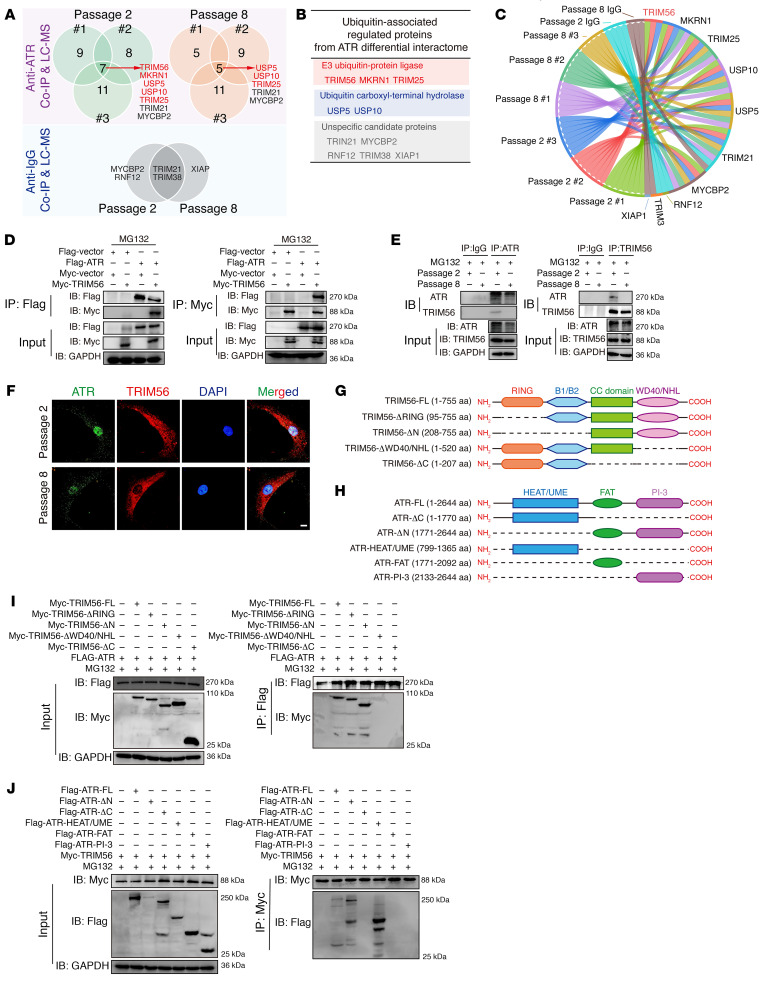
ATR interactomic analysis identifies TRIM56 as an important E3 ligase in the regulation of ATR ubiquitination. (**A** and **B**) Venn diagrams (**A**) and table (**B**) of the ATR interactome showing the intersection of E3 ligases and USPs from NP cells (*n* = 3 biological replicates). (**C**) Chordal graph showing the differential abundance of ATR-interacting E3 ligases and USPs in NP cells. (**D**) Exogenous interaction analysis of ATR and TRIM56 in HEK293T cells. (**E**) Endogenous co-IP analysis showing the difference in ATR and TRIM56 interaction in normal and senescent NP cells. **F**) IF analysis of endogenous colocalization of ATR and TRIM56 in NP cells. Scale bar: 10 μm. (**G** and **H**) Schematic illustration showing TRIM56 (**G**) and ATR (**H**) with mutations in different domains. (**I**) Exogenous co-IP analysis of the interaction domain of TRIM56 binding with ATR. (**J**) Exogenous co-IP analysis of the interaction domain of ATR binding with TRIM56. At least 3 independent experiments were performed.

To identify the E3 ligases or ubiquitin-specific proteases (USPs) that interact with ATR and essentially regulate the dynamic stability of ATR protein in senescent NP cells, we screened E3 ligases and USPs that interacted with ATR, as identified in the interactomics analysis, and found 5 potential E3 ligases and 2 potential USPs as candidate ATR-interacting proteins in normal NP cells, 3 potential E3 ligases and 2 potential USPs in senescent NP cells, as well as 5 unspecific ligases potentially combining with the IgG isotype (Figure 8, A–C). Experimentally, exogenous co-IP experiments verified the interaction between ATR and TRIM56, TRIM25, and USP5, rather than MKRN1 and USP10 (Figure 8D and Supplemental Figure 5, C and D). Similarly, endogenous co-IP experiments verified that ATR strictly interacted with TRIM56, TRIM25, and USP5 in normal NP cells, whereas ATR interacted with TRIM25 and USP5 in senescent NP cells (Figure 8E and Supplemental Figure 5, A and B). Collectively, we focused on the potential function of TRIM56 in the dynamic regulation of ATR protein stability: TRIM56 acted as the only E3 ligase to catalyze the covalent attachment of K63-linked polymeric ubiquitin chains, which interacted with ATR in the normal NP cells, but not in the senescent NP cells. Although IP and MS analysis predicted that MKRN1 is a potential E3 ligase that interacts with ATR in normal NP cells, we found that MKRN1 was a K48-linked ubiquitin-associated E3 ligase, contrary to the K63-linked modification and enhanced protein stability of ATR in normal NP cells. Furthermore, exogenous and endogenous co-IP experiments confirmed no interaction between ATR and MKRN1. Additionally, TRIM25 and USP5 interacted with ATR in both normal and senescent NP cells, which indicates that the 2 ubiquitylation-associated enzymes do not act as the master regulators in guiding the change in ATR dynamic state. Visually, IF staining confirmed the colocalization of ATR and TRIM56 in the normal cellular nucleus but less intense colocalization signaling in the senescent cellular nucleus (Figure 8F). ATR harbors an amino (N)-terminal HEAT region, UME domain, FAT domain, and a carboxyl (C)-terminal PIKKc domain, whereas TRIM56 harbors an N-terminal RING E3 ligase domain, B-box domain, coiled-coil domain, and a C-terminal WD40/NHL homologous region (42) (Figure 8, G and H). Furthermore, exogenous co-IP experiments demonstrated that TRIM56 interacted with ATR largely via the TRIM56 C-terminal WD40/NHL homologous regions, as TRIM56 mutants with these regions deleted (TRIM56-ΔWD40/NHL, TRIM56-ΔC-terminal) failed to bind ATR (Figure 8I). ATR interacted with TRIM56 largely via the ATR N-terminal HEAT/UME region, as ATR with deletion of the N-terminal region failed to bind TRIM56, whereas ATR with HEAT/UME region truncation bound TRIM56 (Figure 8J).

**Figure 9:**
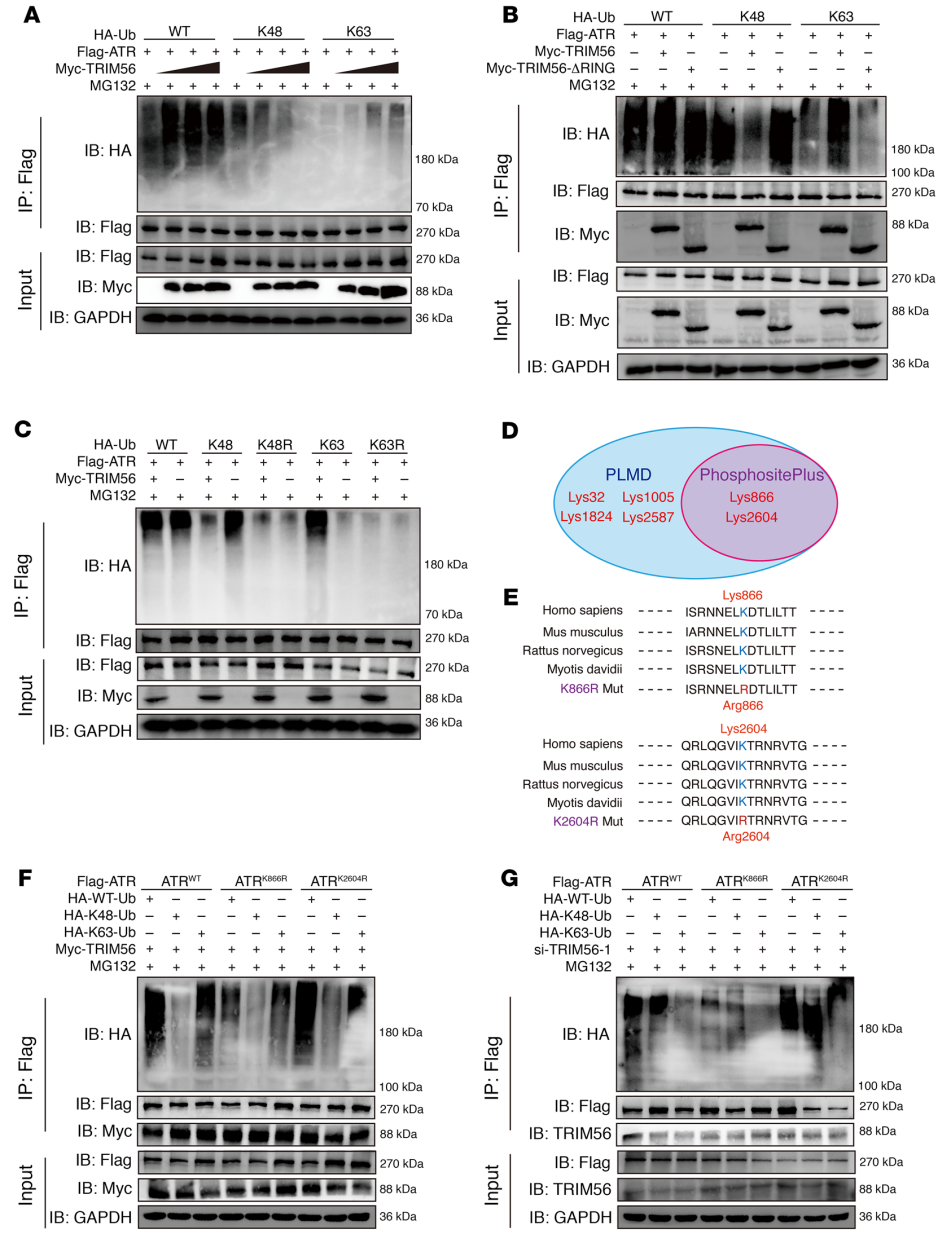
Loss of interaction with TRIM56 drives the ATR ubiquitination shift from K63-linked to K48-linked modification. (**A**) Exogenous K48-and K63-linked ubiquitination (Ub) of ATR in HEK293T cells cotransfected with Myc-tagged TRIM56 at different doses. (**B**) Exogenous K48-linked and K63-linked ubiquitination of ATR in HEK293T cells cotransfected with Myc-tagged TRIM56 or the RING domain–deleting mutant (TRIM56-ΔRING). (**C**) Exogenous K48- and K63-linked ubiquitination of ATR in HEK293T cells cotransfected with specific HA-tagged ubiquitin K-only or K-mutated constructs. (**D**) Venn diagram of the predicted ubiquitinated modification of lysine residues in ATR from the PhosphoSitePlus database and PLMD. (**E**) Sequence conservation analysis of predicted lysine residues in multiple species. (**F** and **G**) Exogenous K48-linked and K63-linked ubiquitination of WT ATR, ATR^K866R^, or ATR^K2604R^ mutants in HEK293T cells in the presence (**F**) or absence (**G**) of TRIM56. At least 3 independent experiments were performed.

Moreover, knockdown of TRIM56 led to a shortened half-life of endogenous ATR protein after exposure to CHX (Supplemental Figure 5, D, F, and G). Interestingly, destabilization of the ATR protein was reversed by MG132 but not by CQ in NP cells with siRNA-induced TRIM56 deficiency (Supplemental Figure 5E). Exogenous gradient overexpression of TRIM56 led to the dose-dependent elimination of the K48-linked ubiquitin chain on ATR and an increase in K63-linked ubiquitylation on ATR (Figure 9A). The RING domain is an essential functional catalytic domain of TRIM56 that directly promotes ubiquitin transfer from an E2 ubiquitin–conjugating enzyme to a specific substrate (41). However, an E3 ligase–inactive truncation due to deletion of the RING domain (TRIM56-ΔRING) failed to catalyze the K63-linked ubiquitination of ATR but increased the K48-linked ubiquitination with no significant change in the interaction between ATR and TRIM56, indicating that, via its RING catalytic domain, TRIM56 played a crucial regulatory role in E3 ligase activity to govern ATR ubiquitination (Figure 9B). To confirm that TRIM56 regulated the assembly of the ubiquitin K63–linked chain on ATR, we cotransfected ATR with ubiquitin K–only (K48-linked or K63-linked) or ubiquitin K–mutated constructs (K48R or K63R) into HEK293T cells. Co-IP results revealed that regulation of the ubiquitinated modification shift of ATR through TRIM56 was abolished by exogenous constructs carrying ubiquitin-specific lysine residues replaced with arginine residues (Figure 9C).

To identify the lysine residues in ATR that are ubiquitinated, we further analyzed the potential ubiquitination of lysine residues in ATR using the PhosphoSitePlus database and the Protein Lysine Modifications Database (PLMD) and noticed 2 predicted ubiquitinated lysine residues (K866 and K2604) in ATR (Figure 9D). Importantly, both K866 and K2604 are highly conserved in multiple species (Figure 9E). The replacement of K866 with an arginine residue (ATR^K866R^) markedly reduced K48-linked and K63-linked ubiquitination levels on ATR with or without TRIM56 expression (Figure 9, F and G). Additionally, the ATR^K866R^ and ATR^K2604R^ mutations did not abolish the interaction between TRIM56 and ATR (Figure 9, F and G). Predictably, knockdown of TRIM56 enhanced the K48-linked ubiquitination of endogenous ATR and was accompanied by the loss of K63-linked ubiquitination in NP cells (Supplemental Figure 5H). A previous study revealed that TRIM56 binds with STING and targets STING for K63-linked ubiquitination (42). However, the K63-linked ubiquitination of STING was not markedly decreased in the TRIM56-deficient NP cells, which may indicate that other E3 ligases regulate the ubiquitination of STING in NP cells to modulate the proinflammatory function of the cGAS/STING axis (Supplemental Figure 5I).

To examine the potentially regulated functions of other ATR-interacting E3 ligases and USPs (TRIM25 and USP5) involved in ubiquitination-associated ATR protein dynamic homeostasis, we knocked down TRIM25 and USP5 expression in NP cells. Interestingly, we found that knocking down TRIM25 or USP5 in normal NP cells had no effect on ATR protein stability; on the contrary, the half-life of endogenous ATR protein was dramatically prolonged in TRIM25- and USP5-deficient senescent NP cells, further indicating the regulated functions of TRIM25 and USP5 in the dynamic imbalance of ATR protein during the NP cell senescence process (Supplemental Figure 5, J–O). Additionally, knockdown of TRIM25 expression endogenously inhibited K48-linked ubiquitination on ATR, with no change in K63-linked ubiquitination in the senescent NP cells. Interestingly, in normal NP cells, TRIM25 deficiency had no effect on K48- or K63-linked ubiquitination on ATR (Supplemental Figure 5W). Notably, inhibition of USP5 using genetic depletion noticeably enhanced endogenous K63-linked ubiquitination on ATR and was accompanied by reduced K48-linked ubiquitination in senescent NP cells (Supplemental Figure 5V). In contrast, genetic silencing of USP5 did not change the ubiquitination state of ATR in normal NP cells. These results, together, suggest that TRIM25 and USP5 participated in the ubiquitination and deubiquitination process of ATR in senescent NP cells but have minor roles in the maintenance of ATR dynamic stability in normal NP cells.

Furthermore, genetic silencing of TRIM25 or USP5 dramatically enhanced ATR protein stability with a prolonged half-life in siRNA-induced TRIM56-deficient NP cells (Supplemental Figure 5, P–U). Moreover, in the presence of TRIM56, knockdown of TRIM25 or USP5 could not drive the ubiquitination change of ATR , whereas in TRIM56-deficient NP cells, TRIM25 silencing clearly reduced endogenous K48-linked ubiquitination without elevated K63-linked ubiquitination of ATR, and USP5 silencing dramatically increased endogenous K63-linked ubiquitination with reduced K48-linked ubiquitination of ATR (Supplemental Figure 5, X and Y). Collectively, these results revealed that TRIM56 acted as a master regulator in maintaining ATR dynamic stability and guiding ubiquitination and deubiquitination of ATR protein: in normal NP cells, assembly of the TRIM56-ATR complex promoted K63-linked ubiquitination of ATR and enhanced the dynamic stability of ATR, whereas during the NP cell senescence process, disassembly of the TRIM56-ATR complex drove aberrant deubiquitination and ubiquitination processes of ATR and promoted the ubiquitylation shift from K63- to K48-linked modification of ATR. USP5 exhibited deubiquitination enzyme activity and removed the K63-linked ubiquitination, and TRIM25 acted as the K48-linked E3 ligase to catalyze the covalent attachment of K48-linked ubiquitin chains, which presented a signal to trigger proteasome-mediated proteolysis.

### Loss of TRIM56 promotes ATR/cytoDNA/cGAS/STING axis–dependent NP cell senescence and IVDD progression.

**Figure 10:**
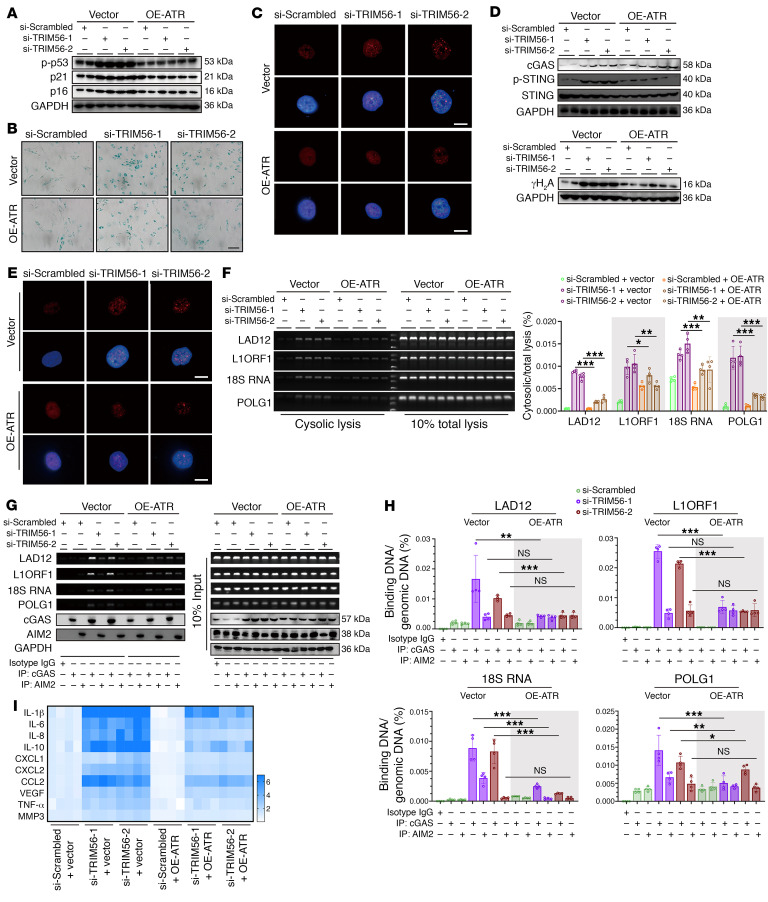
Loss of TRIM56 promotes ATR/cytoDNA/cGAS/STING axis–dependent NP cell senescence. (**A**) Representative Western blots showing p-p53, p21, and p16 in TRIM56-silenced NP cells with or without ATR overexpression (*n* = 2 biological replicates). (**B**) Representative SA–β-gal staining in P2 NP cells with the indicated treatments. Scale bar: 100 μm. (**C**) Representative images of SAHFs in P2 NP cells with the indicated treatments. Scale bars: 10 μm. (**D**) Representative Western blots showing cGAS, STING, p-STING, and γH_2_A expression in P2 NP cells with the indicated treatments (*n* = 2 biological replicates). (**E**) IF staining of γH_2_A foci in P2 NP cells with the indicated treatments. Scale bars: 10 μm. (**F**) Ratio of cytosolic genomic DNA to total genomic DNA in P2 NP cells with the indicated treatments (*n* = 4 biological replicates). (**G** and **H**) DNA IP and ratio of genomic DNA binding with cGAS or AIM2 to total genomic DNA in P2 NP cells with the indicated treatments (*n* = 4 biological replicates). (**I**) Heatmap of differential SASP in P2 NP cells with the indicated treatments (*n* = 4 biological replicates). Data are presented as the mean ± SEM. At least 3 independent experiments were performed. *P < 0.05, **P < 0.01, and ***P < 0.001, by 2-way ANOVA (**F** and **H**).

Overexpression of ATR in NP cells reestablished the TRIM56 deficiency–induced senescence phenotype (Figure 10, A–C). Additionally, a TRIM56 loss-of-function assay showed enhancement of the formation of genomic DNA damage and activation of the cGAS/STING axis, which was partly inhibited by a genetically induced increase in ATR expression (Figure 10, D–H). Interestingly, some genomic DNA was enriched in AIM2-interacting immunoprecipitants from TRIM56-deleting NP cells, which was independent of endogenous ATR expression (Figure 10, G and H). Moreover, increased ATR expression alleviated the inflammatory hypersecretory state of TRIM56-deficient NP cells (Figure 10I). Collectively, the data suggested that genetically increased ATR expression reversed genomic DNA instability and cGAS/STING axis–mediated inflammatory senescence in TRIM56-deficient NP cells.

**Figure 11:**
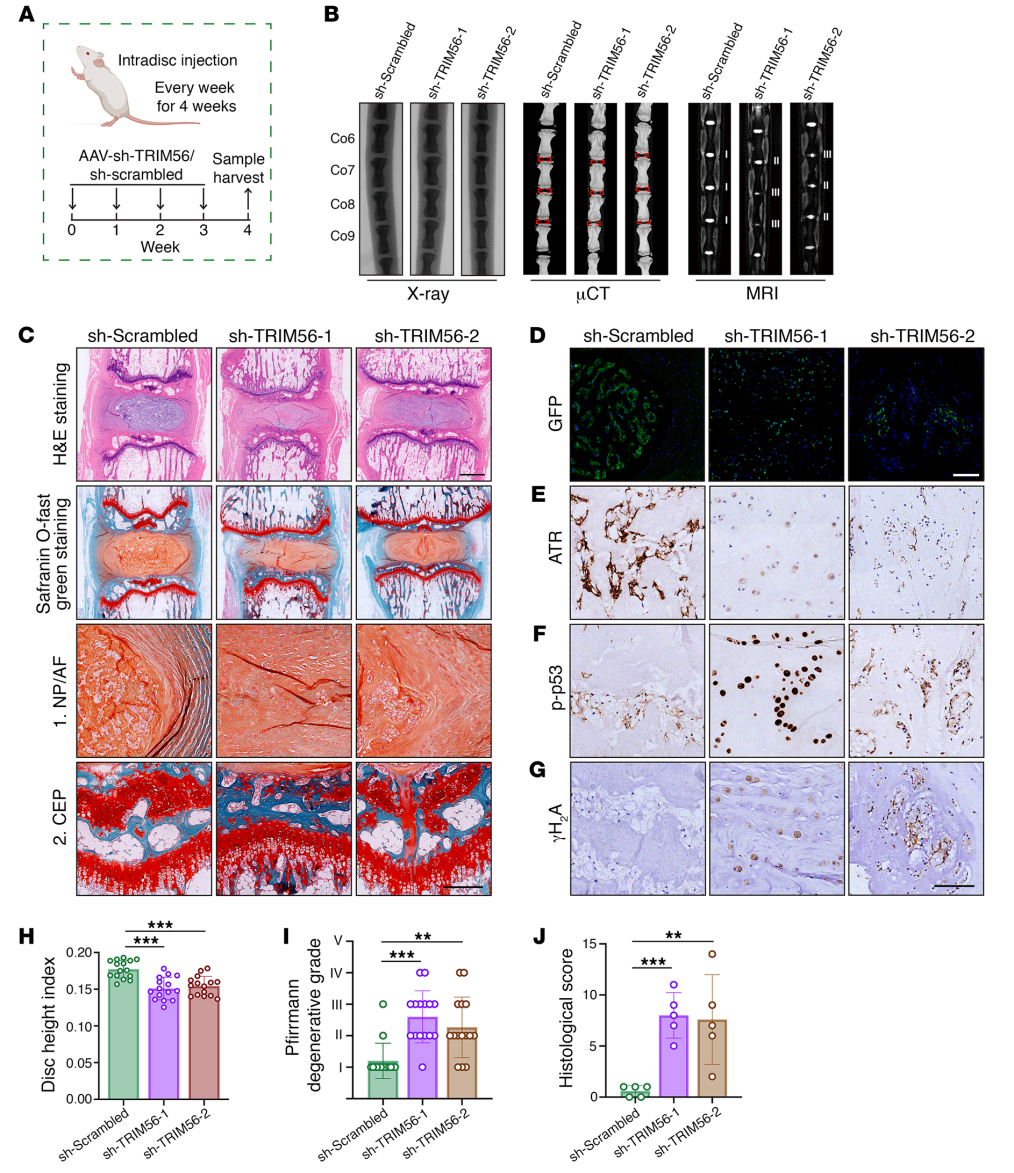
TRIM56 deficiency decreases ATR expression and promotes senescence-associated degeneration of rat coccygeal IVDs. (**A**) Schematic illustration of the experimental design (*n* = 5). (**B**) Representative x-ray images, μCT images, and MRIs of rat coccygeal IVDs treated with an intradisc injection of AAV–sh-scrambled or AAV–sh-TRIM56. (**C**) H&E staining and SO&FG staining of rat coccygeal IVDs. Scale bars: 1 mm. (**D**) IF staining of GFP in rat coccygeal IVDs. Scale bars: 20 μm. (**E**–**G**) IHC staining of ATR (**E**), p-p53 (**F**), and γH_2_A (**G**) in rat coccygeal IVDs. Scale bars: 250 μm. (**H**–**J**) Disc height index (**H**) (*n* = 15 biological replicates), Pfirrmann degenerative grades (**I**) (*n* = 15 biological replicates), and histological score (**J**) (*n* = 5 biological replicates) for rat coccygeal IVDs. Data are presented as the mean ± SEM. At least 3 independent experiments were performed. **P < 0.01 and ***P < 0.001, by 2-way ANOVA (**H**–**J**).

Furthermore, adeno-associated viruses (AAVs) carrying an shRNA targeting TRIM56 (sh-TRIM56) were used to knock down TRIM56 in vivo and were injected into the NP region of rat coccygeal IVDs (Figure 11A). Markedly, x-ray and μCT revealed a reduction in the height of the coccygeal IVDs (Figure 11, B and H). MRI results showed decreased T2-weighted signaling in the coccygeal IVDs, indicating a loss of moisture content in IVDs after TRIM56 knockdown (Figure 11, B and I). H&E and SO&FG staining histologically confirmed that TRIM56 deficiency contributed to degeneration-like phenotype acquisition in coccygeal IVDs, showing a disorganized extracellular matrix, a vague interface between the AF and the NP, and the presence of hypertrophic cells in the inner AF (Figure 11, C and J). Additionally, we observed GFP^+^ NP cells in the IVDs injected with sh-TRIM56–carrying or sh-scrambled–carrying AAVs, demonstrating a high infection efficiency (Figure 11D). Importantly, administration of sh–TRIM56-AAV decreased ATR expression, enhanced the expression of senescence-associated and genomic DNA damage markers, and promoted progressive degeneration of coccygeal IVDs (Figure 11, E–J).

### Cytosolic escape of TRIM56 triggers an ATR dynamic imbalance and promotes NP cell inflammatory senescence via activation of the cGAS/STING axis.

**Figure 12:**
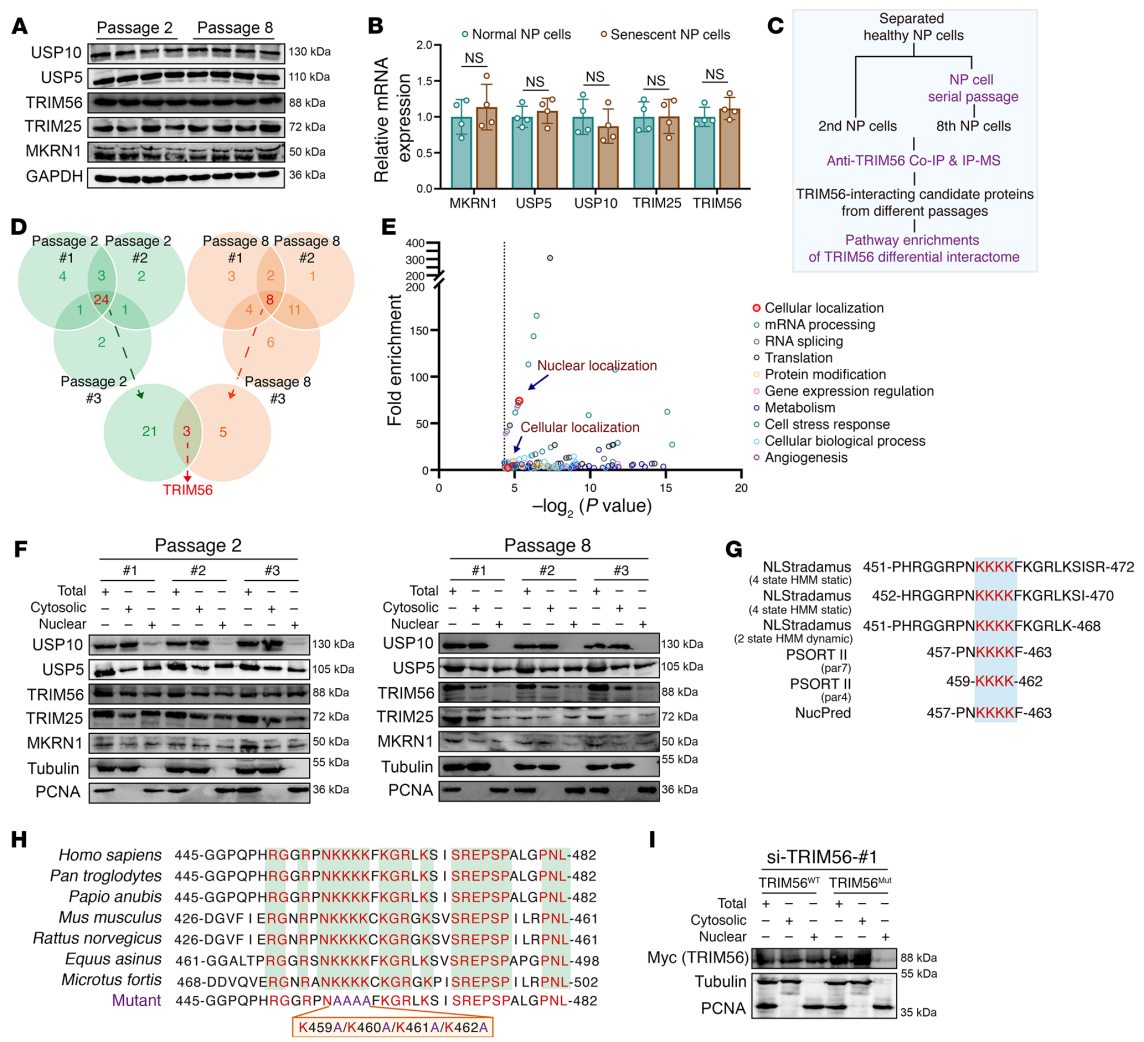
Mislocalization of TRIM56 from the cellular nucleus to the cytoplasm is a unique molecular event occurring in senescent NP cells. (**A**) Representative Western blots of USP10, USP5, TRIM56, TRIM25, and MKRN1 expression in NP cells (*n* = 4 biological replicates). (**B**) Relative mRNA expression of *USP10*, *USP5*, *TRIM56*, *TRIM25*, and *MKRN1* in NP cells (*n* = 4 biological replicates). (**C**) Schematic workflow showing the IP-MS assay to identify TRIM56-interacting proteins (*n* = 3 biological replicates). (**D**) Venn diagrams showing differential ATR interactome in normal (*n* = 3) and senescent (*n* = 3) NP cells. (**E**) GO analysis of TRIM56 interactome showing differential pathways enriched in normal NP cells. (**F**) Representative Western blots showing total, cytosolic, and nuclear TRIM56 in NP cells (*n* = 3 biological replicates). (**G**) Protein sequence diagram showing the predicted NLS in TRIM56 from the NLStradamus database and PSORT. (**H**) Sequence conservation analysis of predicted lysine residues in multiple species. (**I**) Representative Western blots showing total, cytosolic, and nuclear amounts of TRIM56^WT^ and TRIM56^Mut^ in siRNA-induced, TRIM56-deficient P2 NP cells after transfection with Myc-tagged plasmids. Data are presented as the mean ± SEM. At least 3 independent experiments were performed. NS, not significant; unpaired Student’s *t* test (**B**).

**Figure 13:**
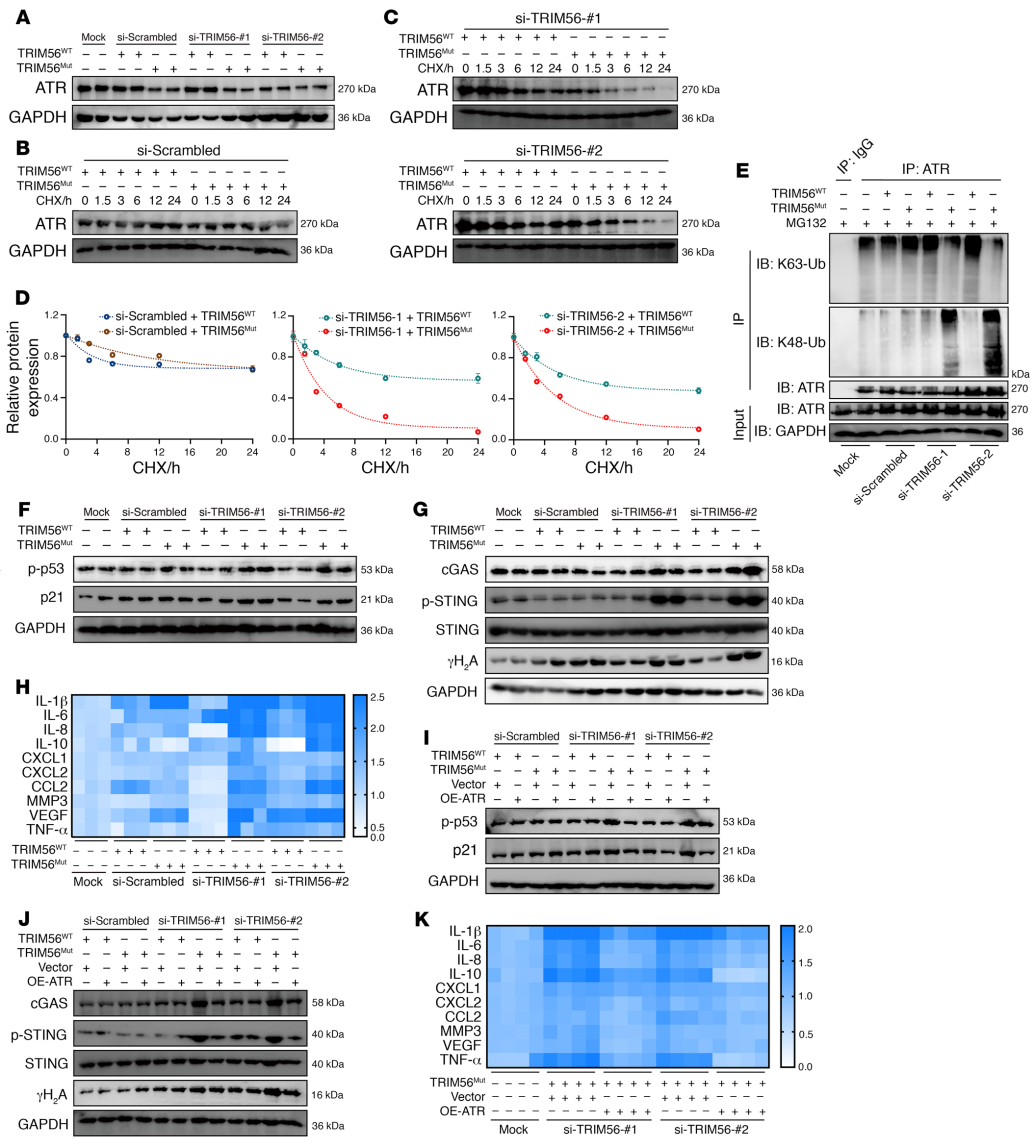
Cytosolic escape of TRIM56 triggers an ATR dynamic imbalance and promotes NP cell inflammatory senescence via activation of the cGAS/STING axis. (**A**) Representative Western blots of ATR in P2 NP cells with the indicated treatments (*n* = 2 biological replicates). (**B**–**D**) Half-life analysis showing endogenous ATR protein levels in siRNA-induced TRIM56-deficient P2 NP cells after transfection with TRIM56^WT^ or TRIM56^Mut^ plasmids and treatment with 100 μg/mL CHX at different time points (*n* = 3 biological replicates). (**E**) Endogenous K48-linked and K63-linked ubiquitination of ATR in P2 NP cells with the indicated treatments. (**F**) Representative Western blots of p-p53 and p21 in P2 NP cells with the indicated treatments (*n* = 2 biological replicates). (**G**) Representative Western blots of cGAS, STING, p-STING, and γH_2_A in P2 NP cells with the indicated treatments (*n* = 2 biological replicates). (**H**) Differential heatmap of SASP in P2 NP cells with the indicated treatments (*n* = 3 biological replicates). (**I**) Representative Western blots of p-p53 and p21 in P2 NP cells with the indicated treatments (*n* = 2 biological replicates). (**J**) Representative Western blots of cGAS, STING, p-STING, and γH_2_A in siRNA-induced TRIM56-deficient P2 NP cells after cotransfection with TRIM56^WT^ or TRIM56^Mut^ and vector or OE-ATR plasmids (*n* = 2 biological replicates). (**K**) Differential heatmap of SASP in P2 NP cells with the indicated treatments (*n* = 4 biological replicates). Data are presented as the mean ± SEM. At least 3 independent experiments were performed.

Surprisingly, we observed no significant difference in mRNA transcriptional levels or protein synthesis of TRIM56 between normal and senescent NP cells (Figure 12, A and B). Given the visual images of IF staining, we inferred that mislocalization of TRIM56 from the cellular nucleus to the cytoplasm spatially blocked the physical exposure of TRIM56 and ATR and further disturbed the ubiquitination modification of ATR (Figure 8F). At the protein interactomic level, endogenous IP-MS analysis identified 21 differential candidate proteins in normal NP cells (Figure 12, C and D). GO analysis notably indicated that these differential proteins were markedly enriched in cellular localization, especially nuclear localization, indicating a loss of nuclear localization of TRIM56 in senescent NP cells (Figure 12E). Experimentally, we found that the ratio of nuclear to cytosolic TRIM56 was drastically decreased in senescent NP cells (Figure 12F). Besides, there were no obvious differences in protein expression or subcellular distributions of other enzymes between normal and senescent NP cells (Figure 12F). To determine whether mislocalization of TRIM56 might trigger ATR dynamic instability and senescence phenotype acquisition of NP cells, we further analyzed the potential nuclear localization signal (NLS) in TRIM56 protein using the NLStradamus database and Protein Subcellular Localization Prediction Tool (PSORT) and noticed a predicted NLS containing 4 continuous lysine residues (K459, K460, K461, and K462) in the TRIM56 peptide, which overlapped in 2 databases (Figure 12G). Importantly, these continuous lysine residues are highly conserved in multiple species (Figure 12H). The replacement of continuous alkaline lysine residues (TRIM56^K459-K460-K461-K462^) with hydrophobic alanine residues (TRIM56^K459A-K460A-K461A-K462A)^) markedly in siRNA-induced TRIM56-deficient NP cells, indicating that continuous lysine residues (aa 459–462) were sufficient for TRIM56 subcellular localization (Figure 12I). To further explore whether nuclear localization of TRIM56 is critical for regulating NP cell senescence, we used WT (TRIM56^WT^) and a localization mutant of TRIM56 (TRIM56^Mut^) plasmid to reconstruct the differential subcellular distribution of TRIM56 protein in siRNA-induced TRIM56-deficient NP cells. Localization-mutant TRIM56 failed to upregulate ATR protein levels and extend the protein half-life of ATR in TRIM56-deficient NP cells, indicating that nuclear localization of TRIM56 was responsible for the maintenance of ATR dynamic stability (Figure 13, A–D). Mechanistically, TRIM56^Mut^ obviously affected the ubiquitination modification of ATR, with reduced K63-linked and enhanced K48-linked ubiquitination in TRIM56-deficient NP cells, collectively suggesting that mislocalization of TRIM56 regulated ATR dynamic stability at the ubiquitination-mediated posttranslational level (Figure 13E). Additionally, TRIM56^Mut^ failed to inhibit the activity of the cGAS/STING axis and inflammatory senescence phenotype acquisition of TRIM56-deficient NP cells (Figure 13, F–H). Predictably, overexpression of ATR in NP cells notably inhibited TRIM56 mislocalization–induced inflammatory senescence phenotype acquisition (Figure 13, I–K). Taken together, these results pinpointed that cytosolic escape of TRIM56 triggered the ATR dynamic imbalance, contributing to the promotion of NP cell inflammatory senescence via cGAS/STING axis activity.

### EV-based ATR-overexpressing plasmid cargo alleviates NP cell senescence and IVDD progression.

Efficient approaches to deliver cargo targeting specific regulators or master signal pathways to NP cells in vivo are critical for the development of disease-modified treatments and clinical applications (43–45). Engineered EVs with low immunogenicity and limited potential to induce side effects integrate natural abilities to mediate cell-cell communication and transfer membrane-encapsulated cargoes to acceptor cells for targeting exogenous intervention to key endogenous signaling pathways, which prevents strong immunogenic reactions and damaging bioactive effects that comprise the safety concerns associated with possible tumor formation (46).

**Figure 14:**
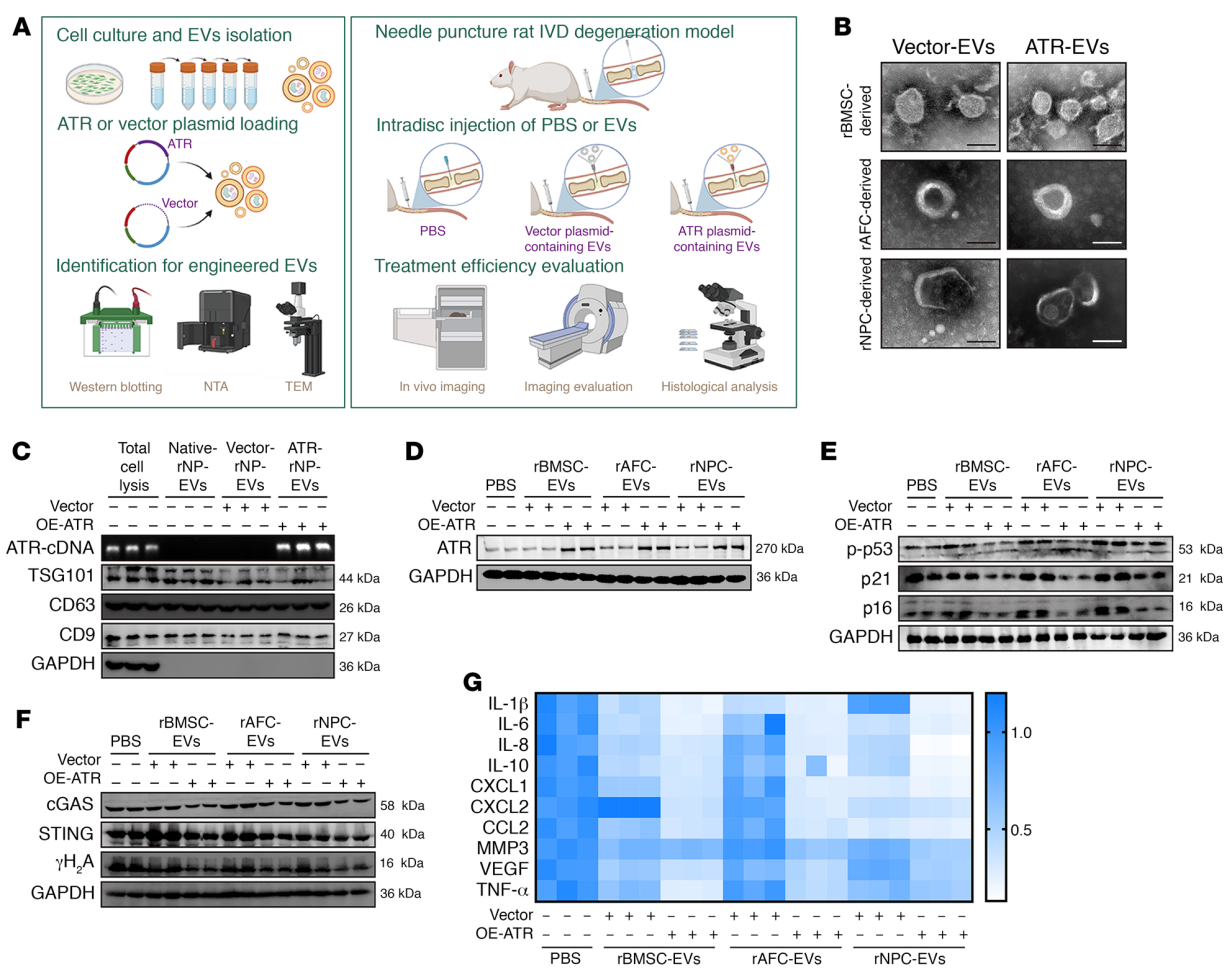
EV-based ATR-overexpressing plasmid cargo alleviates NP cell inflammatory senescence. (**A**) Schematic illustration showing the experimental design. (**B**) Representative TEM images of EVs loaded with ATR-overexpressing (ATR-EVs) or vector plasmid (vector-EVs). Scale bars: 100 nm. (**C**) Western blots of EV markers and cDNA electrophoresis to determine the efficiency of an ATR-overexpressing plasmid loaded into rNPC-derived EVs (*n* = 3 biological replicates). (**D**) Representative Western blots of ATR levels in P8 rat NP cells after culturing with ATR-EVs or vector-EVs for 72 hours (*n* = 2 biological replicates). (**E**) Representative Western blots of p-p53 (Ser15) and p21 levels in P8 rat NP cells with the indicated treatments (*n* = 2 biological replicates). (**F**) Representative Western blots of cGAS, STING, and γH_2_A levels in P8 rat NP cells with the indicated treatments (*n* = 2 biological replicates). (**G**) Differential heatmap of SASP in P8 rat NP cells with the indicated treatments (*n* = 3 biological replicates).

**Figure 15:**
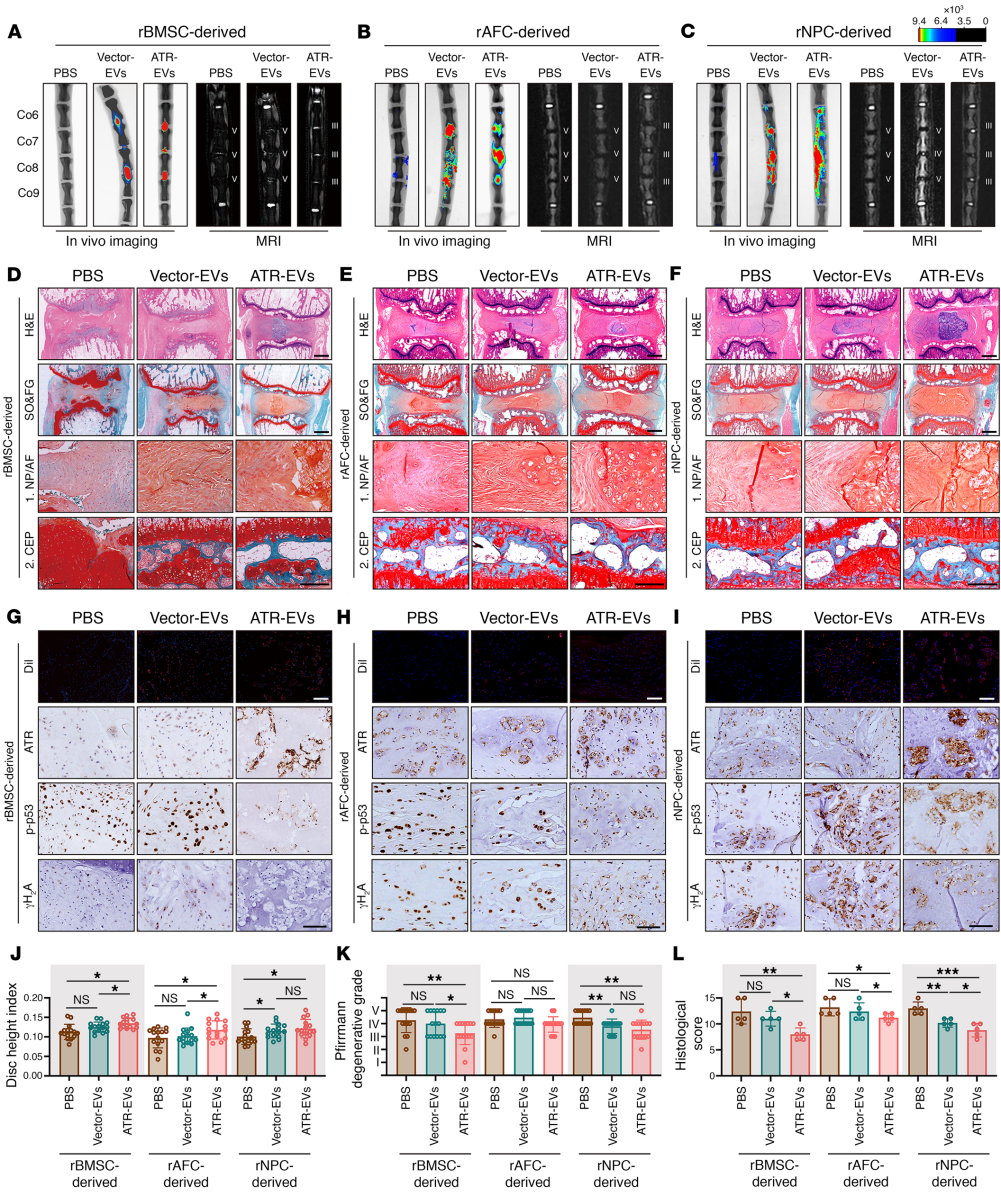
EV-based ATR-overexpressing plasmid cargo exhibits efficient therapeutic effects in ameliorating the severity of IVDD. (**A**–**C**) Representative in vivo images of coccygeal IVDs in rats immediately after treatment with the intradisc injection of ATR-EVs or vector-EVs and representative MRIs of rat coccygeal IVDs treated with an intradisc injection of ATR-EVs or vector-EVs for 4 weeks (*n* = 5). (**D**–**F**) H&E staining and SO&FG staining of rat coccygeal IVDs. Scale bar: 1 mm. (**G**–**I**) IF staining of DiI-labeled EVs in rat coccygeal IVDs (scale bars: 50 μm) and IHC staining of ATR, p-p53, and γH_2_A expression in rat coccygeal IVDs (scale bars: 250 μm). (**J**–**L**) Disc height index (**J**) (*n* = 15 biological replicates), Pfirrmann degenerative grades (**K**) (*n* = 15 biological replicates), and histological score (**L**) (*n* = 5 biological replicates) for rat coccygeal IVDs. Data are presented as the mean ± SEM. At least 3 independent experiments were performed. *P < 0.05, **P < 0.01, and ***P < 0.001, by 2-way ANOVA (**J**–**L**).

Given the large base sequence of the ATR gene, the limited feasible packing approaches, and the excellent properties of cell-derived EVs in the horizontal transfer of cargo among neighboring cells, we used an EV-based strategy to deliver an ATR-overexpressing plasmid that targets NP cells undergoing senescence and progressively damaged IVDDs (Figure 14A). EVs derived from various eukaryotic cells including rat bone marrow mesenchymal stem cells (rBMSCs), rat AF cells (rAFCs), and rat NP cells (rNPCs) were purified via differential ultracentrifugation, and ATR-overexpressing plasmids (ATR-EVs) or vector plasmids (vector-EVs) were subsequently loaded via lipofection (Figure 14A). Isolated EVs were characterized on the basis of the levels of EV-associated protein markers including CD9, CD63, and TSG101 (Figure 14C and Supplemental Figure 6A). Results from cDNA electrophoresis revealed that ATR-containing plasmids or vector plasmids were efficiently loaded into EVs (Figure 14C and Supplemental Figure 6A). ATR-EVs or vector-EVs exhibited an irregular spherical or cup-shaped morphology and were similar in size (Figure 14B and Supplemental Figure 6B). Interestingly, ATR-EV treatment dramatically compensated for the ATR deficiency in senescent rNP cells and obviously alleviated rNP cell senescence with reduced genomic damage and less activation of the cGAS/STING axis (Figure 14, D–G). Surprisingly, the therapeutic effects were not limited to secretory cell sources, suggesting that ATR-EVs might have natural properties in the horizontal transfer of cargo among neighboring cells. Caveolae/lipid raft–dependent endocytosis mediates the internalization of extracellular cargo, and binding of cavin 2, a caveolae-associated protein, to caveolin 1 induced the formation of plasma membrane curvature, which plays a critical role in EV uptake of NP cells (47, 48). To explore whether cavin 2/lipid raft–dependent endocytosis is sufficient for cellular uptake and the protective effect of ATR-EVs, we used an anti–cavin 2–specific antibody and the dynamin pharmacological inhibitor Dynasore to block cavin 2/lipid raft–dependent endocytosis of EVs. Notably, antibody blocking and pharmacological inhibition clearly reduced PKH26-labeled EV uptake of NP cells and significantly resisted the antisenescence and antiinflammatory effects of NPC-derived ATR-EVs despite the homologous NPC–derived EV uptake preference of senescent NP cells (Supplemental Figure 6, C–J). To evaluate the protective effects of ATR-EVs in vivo, DiI-labeled engineered EVs were injected into the NP region of rat coccygeal IVDs after surgical needle puncture. Initially, as shown in the animal images, the signals of the engineered EVs were located in the center of the intervertebral space, ensuring the enrichment of EVs in the NP region (Figure 15, A–C). Importantly, the treatment of ATR-EVs substantially diminished the decline in disc height, the destruction of subchondral bone, and the loss of water content in coccygeal IVDs as determined by radiological assay (Figure 15, A–C, Figure 15, J and K, and Supplemental Figure 6, K–M). Additionally, histological staining suggested that the alleviation of IVDD progression benefited from ATR-EV administration (Figure 15, D–F and L). Furthermore, the enrichment of DiI-labeled red signal in the NP region and the increased expression of ATR protein in NP cells indicated efficient EV uptake and functional cargo release (Figure 15, G–I). The number of NP cells with DNA damage–associated and senescence-associated markers was obviously decreased following ATR-EV treatment, which demonstrated that EV-based delivery of ATR-overexpressing plasmids in coccygeal IVDs efficiently eliminated ATR-deficient NP cell senescence and IVDD progression (Figure 15, G–I). Interestingly, although ATR-engineered EVs had the therapeutic effect of alleviating IVD degenerative progression, rNPC-derived ATR-EVs exhibited the most significant histologically protective effects, followed by rBMSC-derived ATR-EVs, with the least protective effects observed in rAFC-derived ATR-EVs, which might be attributed to an EV uptake preference of NP cells. As shown in flow cytometry and animal IF staining, there were more PKH26^+^ NP cells in vitro and more DiI^+^ NP cells in vivo when NP cells were exposed to NPC-derived ATR-EVs (Supplemental Figure 6, C and D, and Figure 15, G–I). Overall, these results revealed a molecular mechanism and potential strategy of using EV-based ATR-overexpressing plasmid cargo to alleviate NP cell senescence and ameliorate the severity of IVDD.

## Discussion

**Figure 16:**
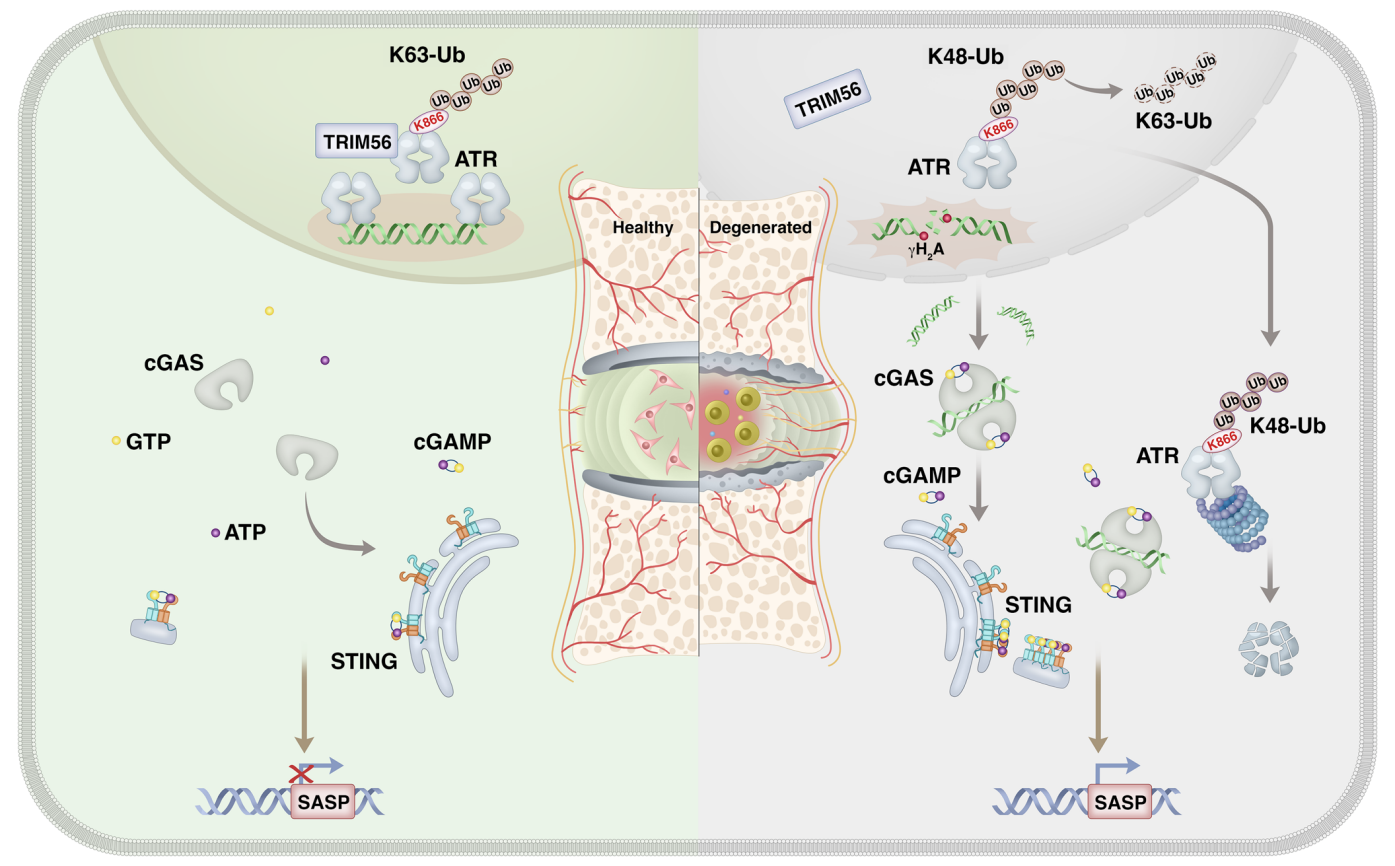
Disassembly of the TRIM56-ATR complex promotes cytoDNA/cGAS/STING axis–dependent intervertebral disc inflammatory degeneration. Schematic illustration showing a mechanism linking ubiquitination of ATR and cytoDNA sensing of the cGAS/STING axis in genomic DNA damage–associated NP cell senescence and IVDD progression.

Although links between genomic instability–induced cellular senescence and premature onset of age-associated disorders have been established, relatively little is known about the mechanism that triggers genomic DNA damage and cellular senescence during organismal inflammation–based degeneration. This study identifies a mechanism linking the ubiquitination of ATR and cytoDNA sensing of the cGAS/STING axis in genomic DNA damage–associated NP cell senescence and IVDD progression (Figure 16).

Recently, studies on IVDD have emphasized the senescence of resident cell populations and the aberrant cell–mediated response to progressive IVD tissue integrity destruction (2). Limitations of self-renewal potential and continuous environmental stresses may contribute to a higher percentage of senescent NP cell clusters and a resultant failure to maintain IVD tissue homeostasis. Our study showed that senescence-associated cytoDNA was driven from specific genomic foci including repetitive DNA, lamin-associated domains, and, interestingly, other genomic regions, which were powerful triggers in mediating the activation of the inflammatory response (31, 49). Importantly, we showed that the cGAS/STING axis was the master nucleic acid sensor in response to the senescence-associated paracrine function of cytoDNA during NP senescence progression. cGAS/STING axis acts as a crucial signaling pathway in sterile inflammation– and age-associated degenerative diseases, in which it senses and responds to cytoDNA in a DNA sequence–independent, but DNA length–dependent, manner. However, the mechanism by which cytoDNA preferentially drives the activation of cGAS, but not AIM2, and other sensors needs to be further explored (50, 51). The NLRP3 inflammasome is a hetero-oligomeric protein complex that is activated by damaged signals, which could be activated by oxidative mitochondrial DNA (mtDNA) (39, 52). In this study, cGAS/STING axis activation promoted p65-mediated transcriptional upregulation of NLRP3 and facilitated assembly of the NLRP3 inflammasome upon sensing damaged stimulation. Thus, we infer that the cGAS/STING axis drives the priming of the NLRP3 inflammasome and improves the cell’s sensitivity to other damaged stimulators such as dysfunctional mitochondria and that the cGAS/STING axis might be sufficient for the hyperinflammatory state of degenerated NP cells.

ATR essentially protects chromosome integrity and accurately controls chromatin dynamics in different cell phases via several independent pathways (14). Additionally, when activated by mechanical stress, ATR modulates nuclear envelope–associated repair of perinuclear chromatin, which is essential for maintaining nuclear envelope integrity and controlling chromatin epigenetics (14, 18). Here, our research indicated that ATR deficiency promoted IVDD, with an increased number of senescent NP cells accompanied by aberrant genomic instability and an enhanced SASP. IVD tissue is a hostile microenvironment for native NP cells with high mechanical loading and high osmotic pressure, and the observation that ATR is required for cellular mechano-responsiveness led to the question: Could ATR regulate nuclear envelope plasticity and chromatin dynamics in NP cells under high mechanical load (2, 5, 14)? Furthermore, maintenance of quiescence or a terminally stable differentiated state upon challenge by intrinsic stresses and extrinsic disturbance is essential for tissue renewal and homeostasis, and the mechanism of action of ATR, which is essential for the regulation of the NP cell-cycle phase, biomechanical response of NP cells, and regenerative capacity of IVD tissues (15).

In summary, we revealed that during IVDD progression, ATR deficiency destructed genomic stability and promoted inflammatory senescence of NP cells through a cytoDNA-cGAS/STING axis–dependent pathway. Mechanically, the loss of TRIM56 interaction with ATR promoted K48-linked ubiquitination–dependent proteolysis of the ATR protein. Notably, administration of an ATR-overexpressing plasmid delivered by EVs efficiently alleviated DNA damage–induced NP cell senescence and substantially mitigated IVDD progression.

## Methods

Additional details on the materials and methods used in this study are available in the supplemental materials. Information on the primer sequences used for PCR genotyping is provided in Supplemental Table 6. Details on the siRNA sequences used in this study are provided in Supplemental Table 7. Detailed characteristics of the antibodies used in this study are provided in Supplemental Table 8.

### Human NP samples.

Human NP samples were collected from volunteers who had lumbar vertebral fracture, idiopathic scoliosis, lumbar disc herniation, or lumbar spondylolisthesis. The clinical characteristics of the volunteers enrolled in this study are provided in Supplemental Tables 1–5.

### RNA-Seq and GSEA.

Total RNA from human NP samples was harvested using TRIzol reagent (Thermo Fisher Scientific) for construction of the RNA-Seq library using the Illumina TruSeq RNA Sample Prep Kit (Illumina).

GSEA was conducted using GSEA 4.3.2 software.

### CUT&Tag-Seq.

Isolated NP cells from human NP tissues were cultured with concanavalin A–coated magnetic beads, incubated with a specific anti-p65 antibody (Cell Signaling Technology) overnight at 4°C. Protein A/G–tagmentation 5 (pA/G-Tn5) was added to the solution and activated with Mg^2+^ to cleave the sequence near the target protein. After PCR amplification, DNA fragments were used to construct a library for high-throughput CUT&Tag-Seq.

For sequencing, FASTQ format raw reads were filtered to clean reads. The peak annotation genes were enriched by GO and KEGG analysis.

### Animals.

Three-month-old male SD rats (weight, 200 ± 20 g) were obtained from the Laboratory Animal Center of the Huazhong University of Science and Technology.

### Coccygeal IVD needle puncture IDD model.

Three-month-old SD rats were housed under a 12-hour light/12-hour dark cycle at 21°C, as described above, and the coccygeal IVD needle puncture IDD model was established using a 20 gauge needle at the Co6-7, Co7-8, and Co8-9 levels (53, 54).

### Statistics.

Data in this study are shown as the mean ± SEM or the median ± IQR. Data were obtained from at least 3 independent experiments. GraphPad Prism, version 9.3.0 (GraphPad Software) was used to analyze data statistical results. An unpaired or paired, 2-tailed Student’s *t* test was used to analyze significance between 2 groups, whereas a 2-way ANOVA was used to analyze significance between multiple groups. A *P* value of less than 0.05 was considered significant.

### Study approval.

The use of volunteer samples was approved by the Ethics Committee of Tongji Medical College, Huazhong University of Science and Technology (approval no. S341). All animal experiments were approved by the Laboratory Animal Center of Huazhong University of Science and Technology (approval no. S2394).

### Data availability.

The raw data from the RNA-Seq and CUT&Tag-Seq analysis have been deposited in the NCBI’s Gene Expression Omnibus (GEO) database (GEO GSE245147 and GSE244403). The mass spectrometry results for ATR and TRIM56 interactomics, uncropped scans of Western blotting experiments, and all raw data used to generate graphs are provided in the Supplemental Supporting Data Values file.

## Author contributions

CY, KZ, KW, WZ, and GL designed the experiments. WZ, GL, and X Zhou performed most of the experiments and analyzed the data. HL, BT, DW, KY, and YS helped perform animal surgeries. BW, ZL, LM, WK, X Zhang, JL, CL, XF, and KW collected the clinical specimens. WZ, KZ, and CY wrote the manuscript.

## Supplementary Material

Supplemental data

Unedited blot and gel images

Supporting data values

## Version 1 01/23/2024

In-Press Preview

## Version 2 03/15/2024

Electronic publication

## Version 3 06/03/2026

revised supporting data values file
