# Supplementary material for: Disassembly of the TRIM56-ATR complex promotes cytoDNA/cGAS/STING axis–dependent intervertebral disc inflammatory degeneration
Source: J Clin Invest. 2024 Jan 23;134(6):e165140. doi: 10.1172/JCI165140 (PMC10940101; doi:10.1172/JCI165140)
Supplement: Supplemental data [file jci-134-165140-s017.pdf]

Supplementary Materials

Disassembly of the TRIM56-ATR complex promotes cytoDNA/cGAS/STING axis-dependent intervertebral disc inflammatory degeneration

Weifeng Zhang, Gaocai Li, Xingyu Zhou, Huaizhen Liang, Bide Tong, Di Wu, Kevin Yang, Yu Song, Bingjin Wang, Zhiwei Liao, Liang Ma, Wencan Ke, Xiaoguang Zhang, Jie Lei, Chunchi Lei, Xiaobo Feng, Kun Wang, Kangcheng Zhao, Cao Yang

These authors contributed equally: Weifeng Zhang, Gaocai Li, Xingyu Zhou

Corresponding authors: Kun Wang, Kangcheng Zhao, Cao Yang

This file includes:

Supplementary Materials and Methods

Supplementary Figure S1 to S6

Supplementary Table 1 to 8

Materials and Methods

Human NP samples

Human NP samples were collected from volunteers who suffered from lumbar vertebral fracture, idiopathic scoliosis, lumbar disc herniation or lumbar spondylolisthesis without hereditary diseases, neurologic abnormalities, cancer, infective diseases, autoimmunity and endocrine diseases, and underwent spinal open or minimally invasive surgery. Clinical medical records and radiographic materials of magnetic resonance imaging (MRI) were used to evaluate the degenerative grades of human NP samples according to Pfirrmann grading system(1). Human NP samples graded as Grade I were classified as the no-degenerated group, NP samples graded as Grade II and Grade III were classified as the mild degenerative group and NP samples graded as Grade IV were classified as the severe degenerative group. Among these samples, three no-degenerative samples and three severe degenerative samples were used to perform RNA transcriptome sequencing, and other samples were used to western blotting analysis and histological staining. Ten age- and sex-matched case pairs were used to evaluate telomere length to avoid the effects of confounding factors. Six no-degenerated and degenerated NP tissues were used to isolate NP cells for CUT-Tag sequencing. Twelve no-degenerated and degenerated NP tissues were used to isolate NP cells for Co-IP analysis. The characteristics details of volunteers enrolled in this study were provided in Supplementary materials (Supplementary Table 1-5). The ethics for using volunteer samples was approved by the Ethics Committee of Tongji Medical College, Huazhong University of Science and Technology (No. S341).

RNA sequencing and Gene set enrichment analysis (GSEA)

Six human NP samples with three replicates from the no-degenerated group and three replicates from the degenerated group as previously described. Human NP samples were digested by 0.2% collagenase II (Invitrogen, Carlsbad, CA, USA). Isolated NP cells were collected by centrifugation and total RNA was harvested using TRIzol reagent (Thermofisher, Waltham, MA, USA). 1 μg of RNA was used to construct RNA sequencing library via Illumina TruSeq RNA Sample Prep Kit (Illumina, San Diego, CA, USA). Libraries were prepared using Illumina HiSeq X ten accompanying the Ovation RNA-Seq System V2, qualitatively checked by a Bioptic Qse100 Analyzer and sequenced on the Illumina HiSeq X ten.

GSEA was conducted using GSEA 4.3.2 software with 1000 permutations and default parameters according to standard procedure. The C2 (Curated gene sets) collection and C5 (Ontology gene set)

collection of the Molecular Signatures Database (MSigDB) version 7.5.1 were used to map overlaps between gene sets in MSigDB and expression set.

After treated with indicated treatments, cultured cells were collected and used for transcriptional sequencing as like the RNA-sequencing and GSEA of human NP samples.

Cleavage Under Targets & tagmentation sequencing (CUT&Tag-seq)

CUT&Tag-seq was conducted according to the previous protocol (Vazyme, Nangjing, China). Briefly, human degenerated and no-degenerated tissues were used to isolate NP cells, and isolated NP cells were cultured with concanavalin A-coated magnetic beads, incubated with specific anti-p65 antibody (Cell Signaling Technology, Danvers, MA, USA) overnight at 4 °C and incubated with second antibody for 1 h at room temperature. After washed, protein A/G-tagmentation 5 (pA/G-Tn5) was added to the solution for 1 h at room temperature. Adding Mg^{2+} triggered the enzyme activity of Tn5 to cleavage the sequence near the target protein, and the target protein and its binding DNA sequences were released to the solution. Dissociative DNA was extracted, and magnetic beads were used to sort DNA fragments below 700 bp. After PCR amplification, DNA fragments were used to constructed a library for high-throughput sequencing.

For sequencing, FASTQ format raw reads were filtrated to clean reads. After quality control, clean reads were mapped according to reference genome to show sample correlation and reads distribution. The peak annotation genes were enriched by GO and KEGG analysis.

Cell culture and treatments

Human NP samples classified as Grade I were obtained from volunteers who were diagnosed with lumbar vertebral fracture or idiopathic scoliosis, as previous described(2, 3). And the samples were cut into pieces, washed twice with phosphate buffer saline (PBS), digested with 0.2% collagenase II (Invitrogen, Carlsbad, CA, USA) for 8 h at 37 ºC, centrifuged at 800 rpm for 5 min and washed twice with PBS. Cellular precipitates were suspended and cultured in 1:1 Dulbecco's Modified Eagle's Medium: F12 (1:1 DMEM: F12; Gibco, Grand Island, NY, USA) with 10% fetal bovine serum (FBS; Invitrogen) and 1% penicillin-streptomycin (Sigma-Aldrich, St. Louis, MO, USA) in the incubator with 5% CO_2 at 37 °C condition. After serial passage culture, NP cells from passage 8 were considered as senescent cells with senescent phenotypes in multi-dimensional and multi-organelle levels. NP cells from passage 2 were used as the control(4, 5).

HEK293T cell line was purchased from the American Type Tissue Culture Collection (ATTC) and

cultured in DMEM (Gibco) supplemented with 10% FBS (Invitrogen).

To mimics the effects of genomic DNA damage, 50 μM cisplatin (MedChem Express, Monmouth Junction, NJ, USA), a chemotherapeutic agent causing DNA interstrand-crosslink lesions, was used to treat NP cells from passage 2 for 24 h and after treatment, NP cells were used in further experiment(6).

To inhibit the activation of STING and NF-κB signal pathway, 1 μM H-151 (MedChem Express) and 10 μM JSH-23 (MedChem Express) were used to treat NP cells from passage 8 for 24 h.

To explore the dynamic stability of ATR protein, 100 μg/ml cycloheximide (CHX; MedChem Express) to inhibit *de novo* protein synthesis was treated to NP cells or HEK293T cells in time gradients (0, 1.5, 3, 6, 12, 24 h), 10 μM MG132 (MedChem Express) to block proteasomal function was treated to NP cells or HEK293T cells for 6 h and 50 μM chloroquine (CQ; MedChem Express) to block lysosomal function was treated to NP cells or HEK293T cells for 6 h.

To induce the oxidative stress, TBHP (MedChem Express) in concentration gradients (0, 20, 40, 60, 80 and 100 μM) was used to treat NP cells for 24 h.

RNA Interference and Plasmid Transfection

Knockdown the expressions of ATR, cGAS, AIM2, TRIM56, USP5 and TRIM25 proteins in NP cells were obtained via using small interfering RNA (siRNA) technology. 50 nM siRNA (TsingKe, Beijing, China) against ATR (si-ATR), cGAS (si-cGAS), AIM2 (si-AIM2), TRIM56 (si-TRIM56), USP5 (si-USP5), or TRIM25 (si-TRIM25) and scrambled siRNA (si-Scrambled) were transfected to NP cells or HEK293T cells with Lipofectamine™ 2000 (Invitrogen) for 72 h. The information of siRNA sequences used in this study was provided in Supplementary materials (Supplementary Table 7). After confirmed the highly-efficient silencing, NP cells or HEK293T cells were used to further explore. The adeno-associated virus (AAVs) containing short hairpin RNA of TRIM56 or scrambled sequence (AAV-sh-TRIM56 or AAV-sh-Scrambled) were generated using the pAAV5-CMV-EGFP system (OBIO, Shanghai, China) according to the manufacture's protocols.

Human ATR wild type (WT), truncations (amino acid 1-1770, 1771-2644, 799-1365, 1771-2092, 2133-2644), mutants (K866R, K2604R) and rat ATR WT were separately tagged with Flag and cloned into empty vector plasmid pCDNA3.1-CMV-ZsGreen to obtain the overexpression plasmids. Human TRIM56 WT, truncations (amino acid 97-755, 206-755, 1-307, 1-222) and mutant (K459A-K460A-K461A-K462A), human USP10, human USP5, human TRIM25 and human MKRN1 were separately tagged with Myc and cloned into empty vector plasmid GV471-CMV-mCheery to

obtain the overexpression plasmids. Human ubiquitin WT, K-only (K48-linked and K63-linked) constructs and K-mutated (K48R or K63R) constructs were cloned into empty vector plasmid pmCherry-HA-6×His. To efficiently transient transfection, NP cells and HEK293T cells were transfected with the above-mentioned plasmids using Lipofectamine™ 2000 (Invitrogen) for 72 h.

Reverse transcription quantitative polymerase chain reaction (RT-qPCR)

After indicated treatment, total RNA from NP cells was extracted with TRIzol reagent (Thermofisher, Waltham, MA, USA) according to the standard protocols. Complimentary DNA (cDNA) was synthetized using the HiScript III RT SuperMix for qPCR (Vazyme, Nanjing, China). Quantitative PCR was conducted using AceQ qPCR SYBR Green Master Mix (Vazyme) and the signals were detected by CFX96 Touch sequence detection system (Bio-Rad, Hercules, CA, USA) and GAPDH was recognized as an internal control to calculate the relative fold changes in gene expressions. The information of primer sequences used in PCR genotyping was provided in Supplementary materials (Supplementary Table 6).

Relative telomere length measurement

Genomic DNA from human NP samples and NP cells was extracted using TIANamp Genomic DNA Kit (TIANGEN, Beijing, China). Purified DNA was amplified using specific telomere primer and AceQ qPCR SYBR Green Master Mix (Vazyme). Single gene copy 36B4u was recognized as an internal control and relative telomere length was calculated with a comparative threshold cycle (Ct) method using the formula $2^{-(\Delta Ct\ (telomere)-\Delta Ct\ (36B4u))}$(7, 8).

Cytosolic genomic DNA measurement

After indicated treatment, genomic DNA in the cytosolic fraction was extracted, measured by quantitative PCR (qPCR) and visualized by DNA agarose gel electrophoresis(9, 10). Briefly, treated cells were divided into two parts: one half was lysed with mild detergent (0.1% NP-40), incubated for 30 min on the ice and centrifuged at 13000 rpm for 15 min at 4 ºC, and the half was lysed with strong detergent. TIANamp Genomic DNA Kit (TIANGEN) was used to purify cytosolic genomic DNA from supernatant cytosolic fraction and whole cellular lysate. Purified DNA was amplified using specific primers and AceQ qPCR SYBR Green Master Mix (Vazyme). DNA content of whole cellular lysates was recognized as an internal control. Then 1.5% agarose gel was used to visualize the PCR products and the images were pictured under ultraviolet light (Bio-Rad, Hercules, CA, USA).

DNA immunoprecipitation

After indicated treatment, DNA fragments binding with cGAS or AIM2 were extracted by specific antibodies, measured by quantitative PCR (qPCR) and visualized by DNA agarose gel electrophoresis(11). Briefly, treated cells were divided into two parts: one half was lysed with pre-cooled NP-40 supplemented with 1% Cocktail and 1% PMSF (MedChem Express), incubated for 30 min on the ice and centrifuged at 12000 rpm for 15 min at 4 ºC, and the half was lysed with strong detergent. Supernatant was immunoprecipitated with protein A/G beads (MedChem Express) conjugated with anti-cGAS or anti-AIM2 antibodies at 4 ºC overnight on a rotator. After washed twice, the beads were divided into two parts: one half was used to elute coprecipitated protein for western blotting analysis, and the other half was used to extract DNA immunoprecipitants by using TIANamp Genomic DNA Kit (TIANGEN). Quantification and visualization coprecipitated DNA were conducted as previous described. And cellular lysate with incubation unspecific isotype IgG antibody was recognized as a negative control.

Western blotting analysis

After indicated treatment, treated cells were lysed with RIPA (Boster, Wuhan, China) supplemented with 1% PMSF (MedChem Express) for 30 min on the ice, centrifuged at 12000 rpm for 15 min, and the protein content of supernatant was measured by BCA protein assay kit (Boster). 20-50 µg proteins were separated by 6-12% SDS-PAGE gel according to molecular weight of targeted protein. And the proteins in SDS-PAGE gel were transferred to polyvinylidene difluoride (PVDF) membrane (Millipore, Billerica, MA, USA), which was subsequently blocked with 5% milk and incubated with primary antibodies at 4 ºC overnight. After washed twice with Tris-buffer saline (TBS) with 0.1% tween-20 and incubated with secondary antibodies, the membrane was visualized with chemiluminescence reagents (Affinity, Liyang, China) and imaged by ChemiDoc MP Imaging System (Bio-Rad). The characteristics details of antibodies used in this study were provided in Supplementary materials (Supplementary Table 8).

Co-immunoprecipitation (Co-IP)

After indicated treatment, treated cells were lysed with NP-40 (Boster) supplemented with 1% Cocktail and 1% PMSF (MedChem Express) for 30 min on the ice, centrifuged at 12000 rpm for 15 min, and the protein content of supernatant was measured by BCA protein assay kit (Boster). 500-1000 µg proteins with the concentration of 1-2 µg/µl were used to incubate with protein A/G beads (MedChem Express) conjugated with specific primary antibodies at 4 ºC overnight on a rotator. And the

immunoprecipitation was eluted by 1 × loading buffer (Boster) and analyzed by western blotting analysis as previous described.

ATR- and TRIM25-interacting protein sequence analysis by immunoprecipitation mass spectrometry (IP-MS)

After indicated treatment, Co-IPs of endogenous human ATR or TRIM56 protein of NP cells from passage 2 or passage 8 were conducted using anti-ATR or anti-TRIM56 antibody or isotype antibodies with protein A/G beads (MedChem Express) at 4 ºC overnight on a rotator. After washed 5 times with pre-cooled NP-40 lysis buffer, immunoprecipitants were eluted with 1 × loading buffer (Boster) and separated SDS-PAGE gel. According to the manufacture's protocol, SDS-PAGE gel piece was decolorized, alkylated and enzymatically disintegrated. The peptides were extracted, identified using Orbitrap Fusion Lumos (Thermo Scientific, Waltham, MA, USA) and analyzed using Proteome Discovery 1.4, Mascot server (v2.3, Matrix Science, London, UK).

Immunofluorescence

NP cells were planted in the glass coverslip. After the medium was replaced twice, NP cells were exposed to indicated treatment. The cellular coverslip was washed twice with PBS, fixed with 4% paraformaldehyde for 20 min, permeabilized with 0.5% Triton-X-100 for 15 min, blocked with normal goat serum for 30 min, incubated with primary antibodies at 4 ºC overnight, washed twice with PBS supplemented with 0.1% tween-20 and incubated with secondary antibodies of anti-mouse/rabbit Alexa Fluor 488 nm or 568 nm. Cellular nucleus was stained with DAPI (Invitrogen). The fluorescent images were captured using a microscope (Olympus, BX53, Tokyo, Japan).

Senescence-associated β-galactosidase (SA-β-gal) staining

After indicated treatment, NP cells were washed twice with PBS, fixed with special fixative for 20 min, incubated in working solution at 37 ºC overnight, washed twice with PBS. The images were captured using an optical microscope (Olympus).

Animals

Three-month-old Sprague-Dawley rats (SD rat, male, 200 ± 20 g) were obtained from the Laboratory Animal Center of Huazhong University of Science and Technology and housed under 12:12 h light:dark cycle at 21 °C condition. All animal experiments were approved by the Laboratory Animal Center of Huazhong University of Science and Technology (No. S2394).

Intradiscal injection of cisplatin

Three-month-old SD rats were housed in the previous condition and randomly divided into 4 groups with 5 rats in each group. After anesthesia with an intraperitoneal injection of 3% pentobarbital (40 mg/kg), a total of 2 μl solution (PBS or cisplatin with gradient concentrations of 1 μM, 10 μM and 100 μM) was slowly injected into the center of NP regions of Co6-7, Co7-8 and Co8-9 coccygeal IVDs every week for one month by using a 33-gauge needle (Hamilton, Bonaduz, Switzerland) attached to a microliter syringe (Hamilton) in order to eliminate the influence of injection solution. And Co5-6 and Co9-10 IVDs were left as self-negative control. After one month, the rats were sacrificed for radiographic and histological analysis.

Intradiscal injection of AAVs

Three-month-old SD rats were housed in the previous condition and randomly divided into 4 groups with 5 rats in each group. After anesthesia with 3% pentobarbital, a total of 2 μl solution containing AAV-sh-TRIM56 or AAV-sh-Scrambled (10^6 plaque-forming units) was slowly injected into the center of NP regions of Co6-7, Co7-8 and Co8-9 coccygeal IVDs every week for one month by using a 33-gauge needle (Hamilton) attached to a microliter syringe (Hamilton)(2, 12, 13). And Co5-6 and Co9-10 IVDs were left as self-negative control. After one month, the rats were sacrificed for radiographic and histological analysis.

Coccygeal IVDs needle puncture IDD model

Three-month-old SD rats were housed in the previous condition and coccygeal IVDs needle puncture IDD model was established by using 20-gauge needle at the Co6-7, Co7-8 and Co8-9 levels(2-4, 13). Briefly, after anesthetized with 3% pentobarbital, rats were placed in a prone position. The Co6-7, Co7-8 and Co8-9 coccygeal IVDs were punctured by a 20-gauge needle along vertical direction and parallel to the CEPs. The needle was inserted for 5 mm to reach the center of NP region, and rotated by 180° in the axial direction and held for 10 seconds. Co5-6 and Co9-10 IVDs were left as self-negative control.

Rat primary cell isolation and culture

Rat bone marrow mesenchymal stem cells (rBMSCs) were obtained from the bilateral femurs of three-month-old SD rats, isolated by density gradient centrifugation and adhered to tissue culture plastic. rBMSCs were cultured in the 1:1 DMEM: F12 growth medium combined with 10% FBS and 1:100 penicillin-streptomycin in the incubator with 5% CO_2 at 37 °C condition. The medium was changed every three days. Rat NP and AF cells (rNPCs and rAFs) were obtained from the coccygeal

IVDs of three-month-old SD rats, and isolated as above mentioned.

EVs isolation and plasmid loading

After rBMSCs, rNPCs and rAFs were passaged to second generation, the medium was replaced with 1:1 DMEM: F12 treated with 15% EV-free FBS (System Bioscience, San Francisco, CA, USA) and collected for isolating EVs. EVs were purified according to the differential ultracentrifugation protocols(8, 14-16). Briefly, collected medium was centrifuged sequentially at 600×g for 10 min, 2000×g for 30 min and 10000×g for 30 min to remove dead cells or cell debris, and ultra-centrifuged at 20000×g for 60 min and washed twice with PBS to isolate EVs. Finally, EVs were suspended with PBS and stored in 4 °C for further experiments. BCA protein assay kit (Boster) was using to measure the protein concentration of EVs. A total of 4 μg vector or ATR-overexpressing plasmids were loaded into 200 μl EVs (100 μg/ml) using Lipofectamine™ 2000 (Invitrogen) according to the standard protocols.

EVs identification

For observing the morphology of engineered EVs, a total of 20 μl EVs was loaded onto a formvar/carbon-coated grid for 5 min. After dried on bibulous paper, the grid was immediately negatively stained with 3% aqueous phosphotungstic acid for 1 min, and dried overnight. The grid was pictured using transmission electron microscopy (TEM) (FEI Tecnai G20 TWIN, Hillsboro, OR, USA).

For analyzing the concentration and size of engineered EVs, a total of 20 μl EVs were diluted 100- to 500-fold with precooled PBS for measurement. ZetaView Multiple Parameter Particle Tracking Analyzer (Particle Metrix, Meerbusch, Germany) was using to assess the number and size of EVs via nanoparticle tracking analysis.

For identifying protein markers of EVs, western blotting was used to analyze the protein content of EVs involved in EV-markers including CD9, CD63 and TSG101. Briefly, as described previously, protein concentration was measured by BCA protein assay kit and a total of 10 μg protein were separated on 10% SDS-PAGE gel, transferred to 0.45 μm PVDF membranes and incubate with special primary antibodies. Cellular proteins were used as positive condition.

For evaluating the loading of ATR-overexpressing plasmids, a total of 200 μl EVs (100 μg/ml) was used to exact DNA cargo in engineered EVs via TIANamp Genomic DNA Kit (TIANGEN). Isolated DNA cargo was amplified by using the special primers of ATR gene, and visualization of amplified

DNA was conducted as previous described. Cellular cDNA was used as positive condition.

For evaluating the uptake of EVs in vitro, FACSCalibur flow cytometer (BD Biosciences, San Jose, CA, USA) was used to detect the fluorescent intensity of PKH26 in NP cells cocultured with PKH26-labelled EVs for 72 h.

To block the EV uptake, 50 μM Dynasore (MedChem Express) and 100 ng/ml anti-Cavin-2 (Proteintech, Chicago, IL, USA) or isotype IgG (Proteintech) were used to treat NP cells cocultured with EVs for 72 h.

Intradiscal injection of EVs

After the initial coccygeal IVDs needle puncture, rats were randomly and double-blindly assigned to 3 treatment groups with 5 rats in each group. After anesthesia with 3% pentobarbital, a total of 2 μl solution containing PBS, vector-EVs (100 μg/L) and ATR-EVs (100 μg/L) was slowly injected into the center of NP regions of Co6-7, Co7-8 and Co8-9 coccygeal IVDs every week for one month by using a 33-gauge needle. Immediately, the rats were sacrificed for in vivo imaging analysis to confirm that engineered EVs were injected into the NP regions of coccygeal IVDs in vivo, and after one month, the rats were sacrificed for radiographic and histological analysis.

EVs labeling and in vivo animal imaging system

Injected EVs were pre-labeled with 5 μM DiI (Sigma-Aldrich) according to the manufacturer's protocols. After anesthetized 3% pentobarbital with injection with labeled EVs, the rat-tails were imaging by using a FX RPO animal imaging system (Bruker, Karlsruhe, Germany) at a 550 nm excitation wavelength and a 600 nm emission wavelength.

Radiological analysis

X-ray, micro-Computed Tomography (μCT) and MRI analyses were used to evaluate the radiological degenerative degrees of coccygeal IVDs of rats. After 4 weeks of treatments, the rats were anesthetized with 3% pentobarbital and sacrificed for collecting rat-tails. The following X-ray parameters was set for scanning rat-tails: an exposure time of 0.06 s; a distance of 100 cm; a current of 160 mA and a voltage of 50 kV using DRX Ascend system (Carestream, Rochester, NY, USA). The following μCT parameters was set: an imaging pixel size of 18 μm; a current of 100 μA and a voltage of 60 kV using μCT scanning system (SkyScan 1176, Bruker). Subsequently, coccygeal IVDs were three-dimensionally restricted by CT-Vox software (Bruker). The following parameters of T2-weighted imaging were set: a fast spin echo sequence with a time to repetition of 3000 ms, a time to echo of 70

ms and a slice thickness of 0.5 mm with a 0 mm gap using a 7.0 T animal specific MRI system (Bruker Pharmascan, Germany).

Histological analysis

Human NP specimens were washed with PBS twice and fixed for two days with 4% paraformaldehyde, dehydrated, embedded in paraffin, and cut into 5 μm sections. After radiological analysis, coccygeal IVD specimens of rats were washed with PBS twice and fixed for two days with 4% paraformaldehyde, decalcified with 10% EDTA solution, dehydrated, embedded in paraffin, and cut into 5 μm sections. The sections were stained by hematoxylin/eosin (H&E) and safranin O/fast green (SO&FG) to analyze the degenerative degrees of IVDs. The sections were imaged using the BX53 microscope (Olympus).

Immunohistochemistry (IHC) staining

Slides of human NP samples or rat coccygeal IVD specimens were deparaffinized with xylene, rehydrated with ethanol in gradient concentrations, antigen-retrieved with citrate buffer, blocked with normal goat serum, incubated with special primary antibodies overnight. After incubated with secondary antibodies and visualized with DAB peroxidase substrate kit, histological images were analyzed using the BX53 microscope (Olympus).

Proximity ligation assays (PLA)

The slides were prepared according to IHC staining as previously described, and PLA was performed according to the published protocol (Sigma-Aldrich). After deparaffinized, rehydrated and antigen-retrieved, the slides were blocked for 60 min at 37 °C condition and incubated with primary antibodies at 4 °C condition overnight. After washed, the slides were incubated with PLA probes at 37 °C condition for 60 min and the ligase at 37 °C condition for 30 min. After amplificated at 37 °C condition for 100 min, the slides were washed and stained with DAPI for 15 min at room temperature.

Statistical analysis

Data in this study were showed as mean ± SEM or median ± IQR. The data were obtained from at least three independent experiments. Unpaired student's t test or pair t test was used to analyze the significance between two groups, whereas two-way ANOVA was used to analyze the significance among multiple groups. GraphPad Prism version 9.3.0 (La Jolla, CA, USA) was used to analyze data and statistical results. The number of stars was used to present the significant degree p value: * $p <0.05$; ** $p <0.01$; *** $p <0.001$.

Figure S1. cGAS was responsive for cytoDNA sensing during NP cell inflammatory senescence

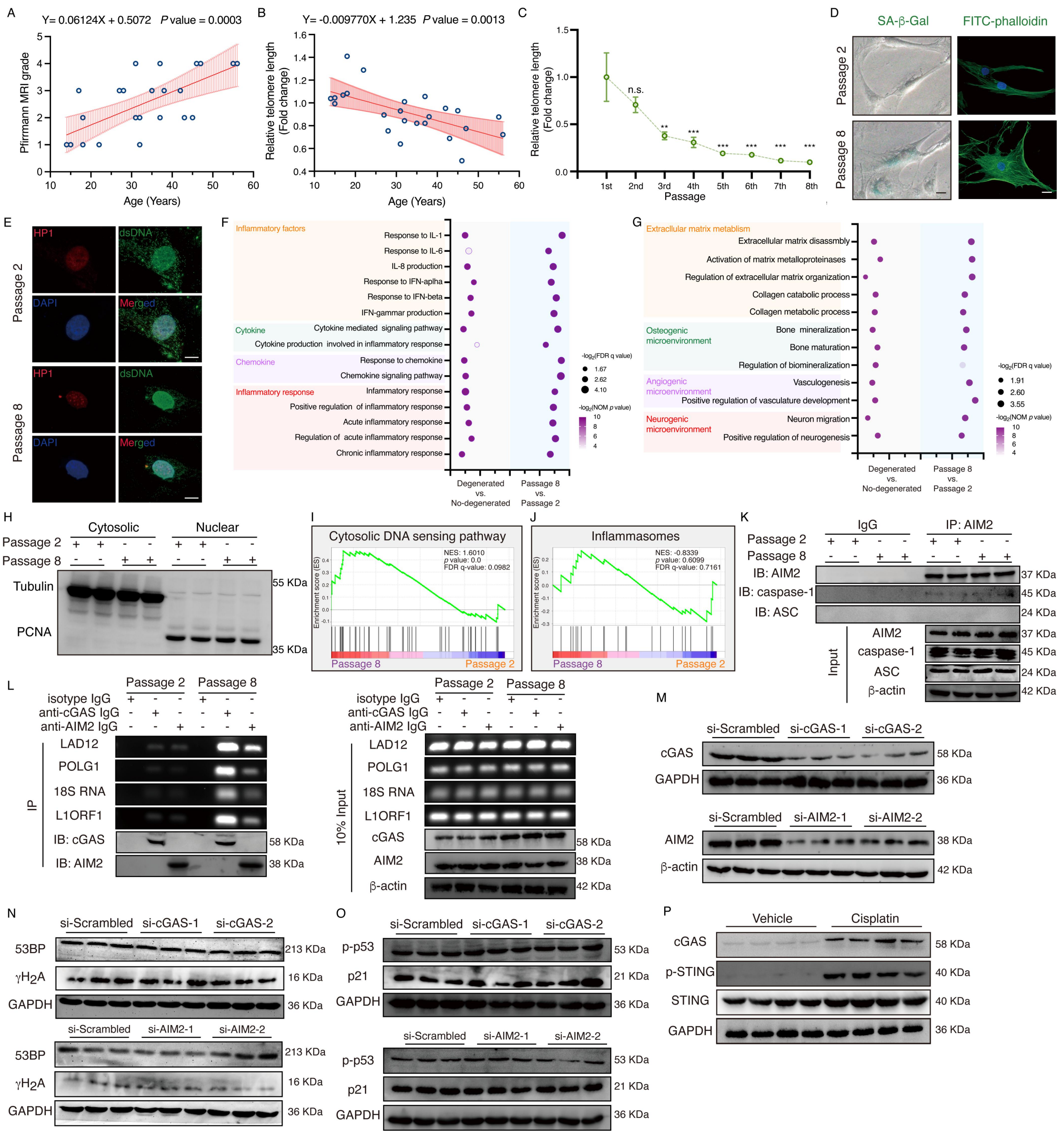

A) Correlation analysis between age (Years) and Pfirrmann MRI-degenerative grade (n = 24). **B**) Correlation analysis between age (Years) and relative telomere length (n = 24). **C**) Change in relative telomere lengths in NP cells in different passages during continuously passage culturing (n = 4 biological replicates). **D**) Representative images showing SA-β-gal staining in P2 and P8 NP cells. Scale bar: 20 μm. And representative images showing morphological differences of P2 and P8 NP cells with cytoskeleton stained with FITC-conjugated phalloidin. Scale bar: 100 μm. **E**) Representative images showing HP1γ-containing SAHFs from P8 NP cells. Scale bar: 10 μm. **F**) GSEA-enriched inflammation-associated pathways in P8 NP cells. **G**) GSEA-enriched degeneration-associated pathways in P8 NP cells. **H**) Representative images of western blotting showing Tubulin and PCNA in

isolated cytosolic and nuclear components from P2 and P8 NP cells (n = 2 biological replicates). **I**) GSEA of RNA-Seq data from normal and senescent NP cells (n = 3 biological replicates). “Cytosolic DNA sensing pathway” was enriched in senescent P8 NP cells. **J**) GSEA of RNA-seq data from normal and senescent NP tissues (n = 3 biological replicates). “Inflammasome” wasn’t significantly enriched in P8 NP cells. **K**) Co-IP assay performed to detect the endogenous formation of AIM2 inflammasome in normal and senescent NP cells (n = 2 biological replicates). **L**) Representative images of western blotting combined with agarose gel electrophoresis of coimmunoprecipited genomic DNA and proteins from normal and senescent NP cells after incubation with anti-cGAS or anti-AIM2 antibody. **M**) Representative western blotting image showing the efficient reduction in cGAS and AIM2 levels in P8 NP cells after treatment with specific siRNAs targeting cGAS (si-cGAS) and AIM2 (si-AIM2) (n = 3 biological replicates). **N**) Representative western blotting images showing γH_2A and 53BP in P8 NP cells after treatment with si-cGAS and si-AIM2 (n = 3 biological replicates). **O**) Representative western blotting images showing p-p53 (Ser15) and p21 in P8 NP cells after treatment with si-cGAS and si-AIM2 (n = 3 biological replicates). **P**) Representative western blotting images showing cGAS, STING and p-STING in P2 NP cells treated with 50 μM cisplatin for 24 h (n = 4 biological replicates). Simple linear regression (**A**, **B**) and Two-way ANOVA (**C**) were used to performed, and data are represented as mean ± SEM. At least 3 independent experiments were performed. * $p < 0.05$; ** $p < 0.01$; *** $p < 0.001$; n.s. not significant.

Figure S2. STING pharmacological inhibitor could suppress p65-mediated transcriptional remodeling and inflammatory phenotype acquisition

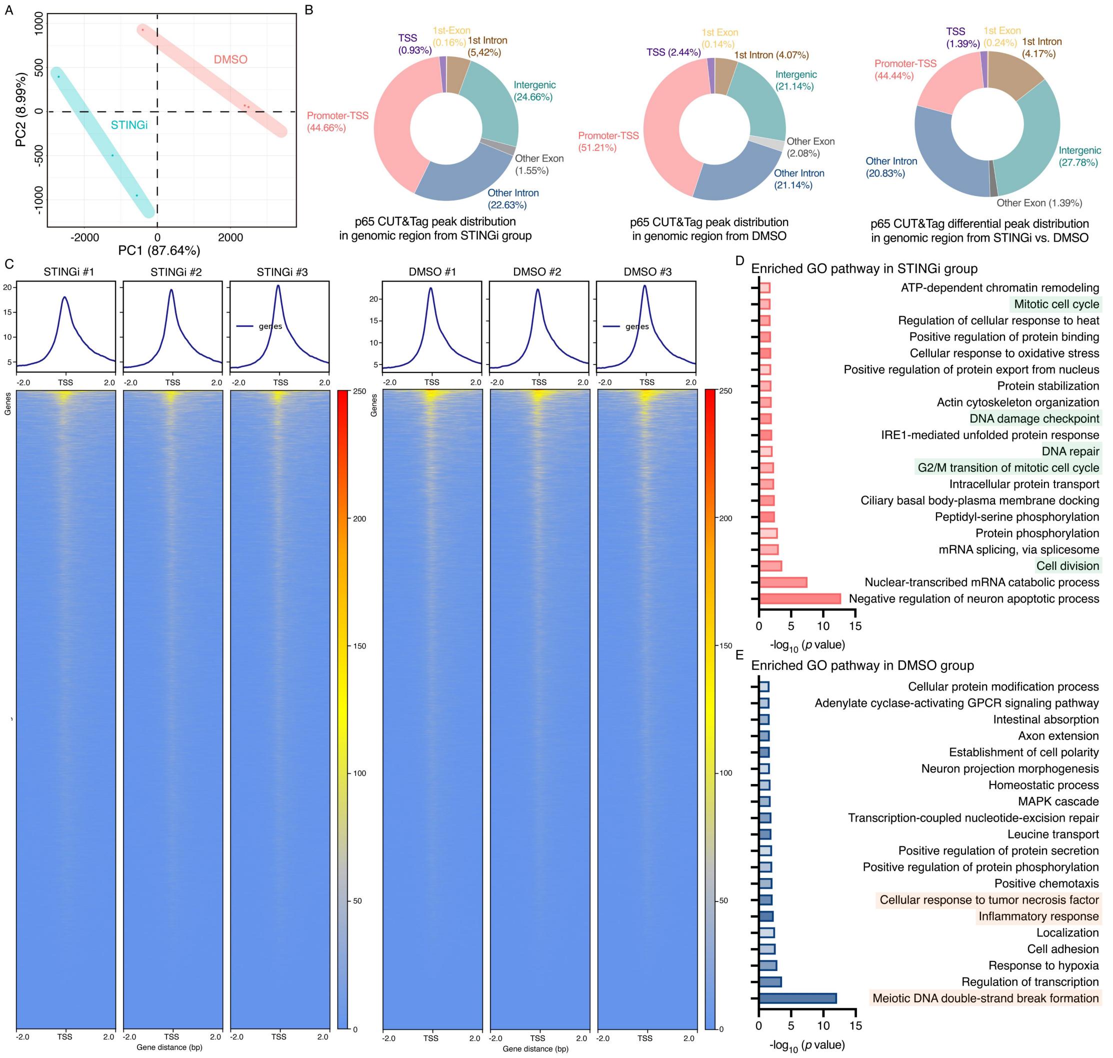

A) PCA plot showing clustering of p65-bound peaks of P8 NP cells after treated with DMSO or H-151 via CUT&Tag-Seq analysis (n = 3 biological replicates). **B**) Distribution of p65-bound genomic regions. **C**) Heatmap showing p65-binding peaks detected by CUT&Tag-Seq between DMSO- and H-151-treated P8 NP cells. **D**) GO analysis of p65-bound peak annotations after H-151 administration. **E**) GO analysis of p65-bound peak annotations after DMSO administration.

Figure S3. cGAS-STING axis activation facilitated the inflammatory hypersensitivity to damaged signals via p65-mediated NLRP3 inflammasome priming

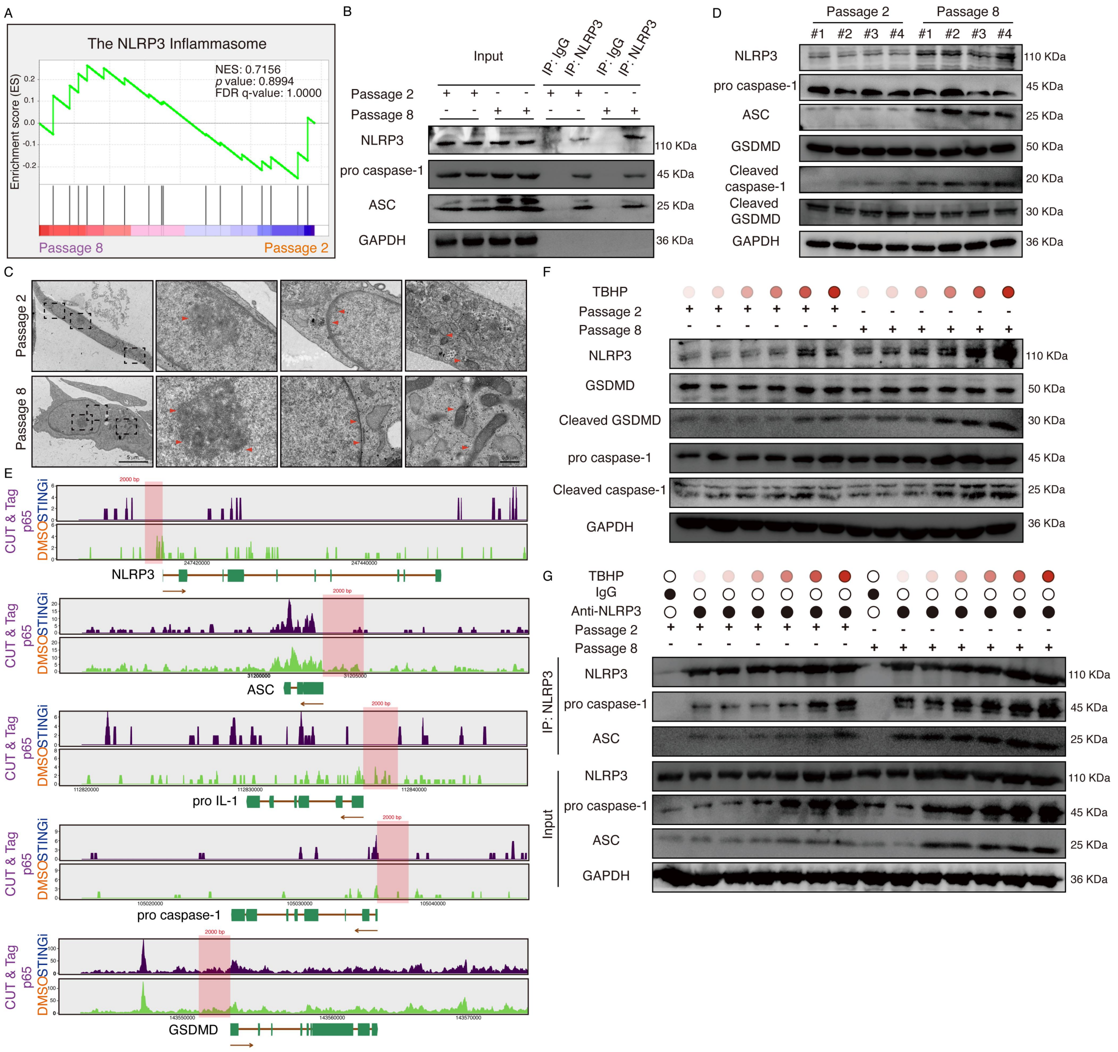

A) GSEA of RNA-Seq data from normal and senescent cells. "The NLRP3 inflammasome" wasn't significantly enriched in senescent P8 NP cells. **B**) Co-IP assay performed to detect the endogenous formation of NLRP3 inflammasome in normal and senescent NP cells (n = 2 biological replicates). **C**) Representative TEM images of P2 and P8 NP cells. Red-triangle showing high-electron gathering in cellular nucleus, thin high-electron region of nuclear membrane and mitochondria. Scale bar: 5 µm, 0.5 µm. **D**) Representative western blotting images showing NLRP3, ASC, pro caspase-1, GSDMD, cleaved caspase-1 and cleaved GSDMD in normal and senescent NP cells (n = 4 biological replicates). **E**) Integrative genomic viewer (IGV) browser views showing p65-binding peaks in H-151-treated P8 NP cells, near the NLRP3, ASC, pro IL-1, pro-caspase-1 and GSDMD gene promotors. **F**) Representative western blotting images showing NLRP3, ASC, pro caspase-1, GSDMD, cleaved

caspase-1 and cleaved GSDMD in normal and senescent NP cells after treated with TBHP in concentration gradients (0, 20, 40, 60, 80, 100 μM). **G**) Co-IP assay performed to detect the endogenous formation of NLRP3 inflammasome in normal and senescent NP cells after treated with TBHP in concentration gradients. At least 3 independent experiments were performed.

Figure S4. Loss- and gain-of-function assay revealed the protective effects of ATR in maintaining NP cell homeostasis and resisting genomic damage

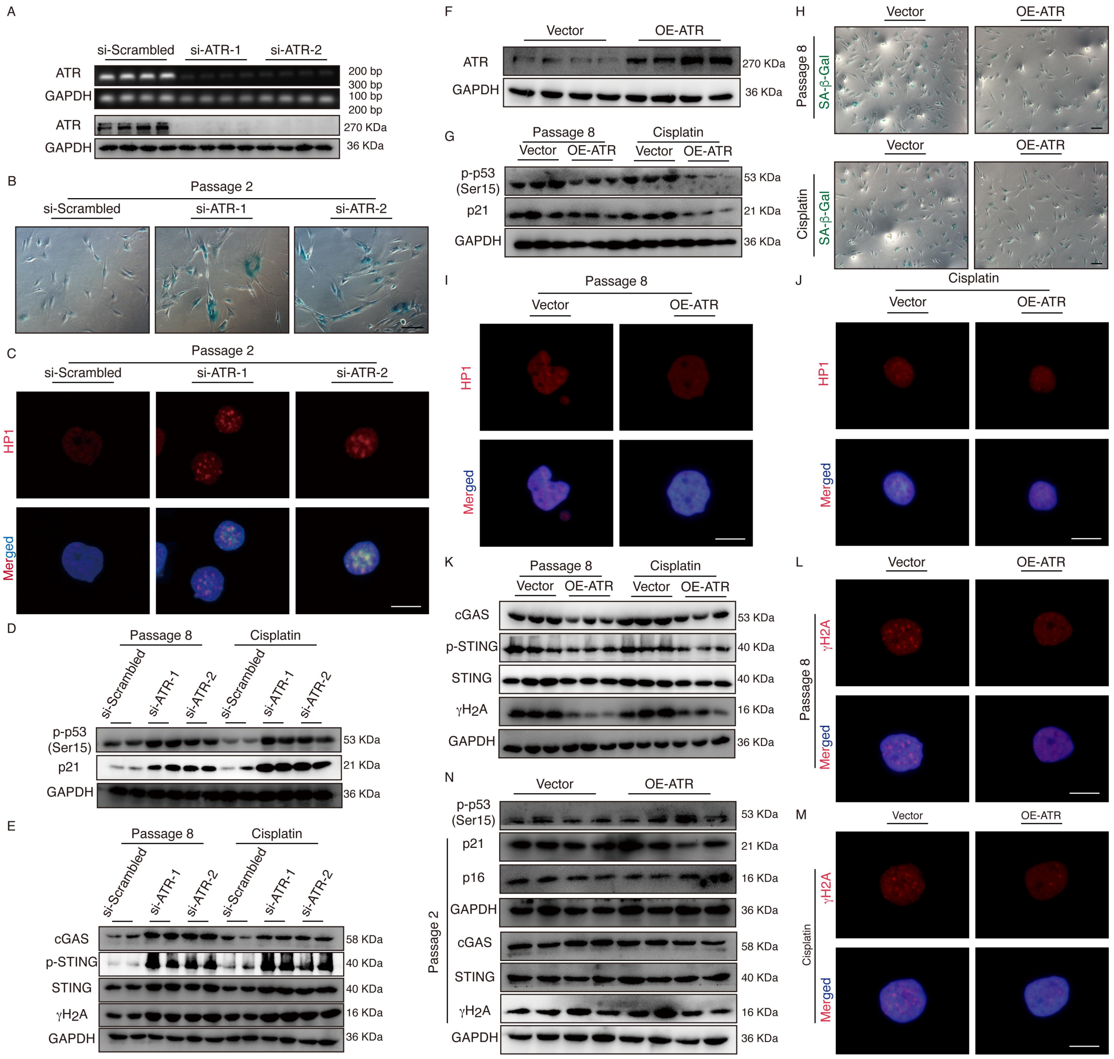

A) RT-qPCR followed by cDNA agarose gel electrophoresis and western blotting analysis detecting the efficient knockdown of ATR expression level in P2 NP cells after treated with specific siRNAs targeting ATR (si-ATR) (n = 4 biological replicates). **B**) Representative imaged of SA-β-gal staining in P2 NP cells after the indicated treatment. Scale bar: 100 μm. **C**) Representative images of HP1γ-containing SAHFs from P2 NP cells after the indicated treatment. Scale bar: 10 μm. **D**) Representative western blotting images of p-p53 and p21 in P8 and cisplatin-treated NP cells after transfected with two specific si-ATR (n = 2 biological replicates). **E**) Representative western blotting images of cGAS, STING, p-STING and γH_2A in P8 and cisplatin-treated NP cells after transfected with two specific si-ATR (n = 2 biological replicates). **F**) Representative western blotting analysis detecting the efficiency of ATR overexpression in P2 NP cells transfected with ATR-overexpressing

plasmids (n = 4 biological replicates). **G**) Representative western blotting images of p-p53 and p21 in P8 and cisplatin-treated NP cells after transfected with ATR-overexpressing plasmids (n = 3 biological replicates). **H**) Representative SA-β-gal staining images from P8 and cisplatin-treated NP cells after the indicated treatment. Scale bar: 100 μm. **I** and **J**) Representative images of HP1γ-containing SAHFs from P8 **I**) and cisplatin-treated NP cells **J**) after the indicated treatment. Scale bar: 10 μm. **K**) Representative western blotting images showing cGAS, STING, p-STING and γH_2A in P8 and cisplatin-treated NP cells after the indicated treatment (n = 3 biological replicates). **L** and **M**) IF staining of γH_2A foci in P8 **L**) and cisplatin-treated NP cells **M**) after the indicated treatment. Scale bar: 10 μm. **N**) Representative western blotting images of p-p53 (Ser15), p21, cGAS, STING and γH_2A in P2 NP cells after transfection with ATR-overexpressing plasmids (n = 3 biological replicates). At least 3 independent experiments were performed.

Figure S5. Lost interaction of ATR with TRIM56 triggered enzyme activity liberation of USP5 and TRIM25, disturbing ATR dynamic homeostasis during NP cell senescence progression

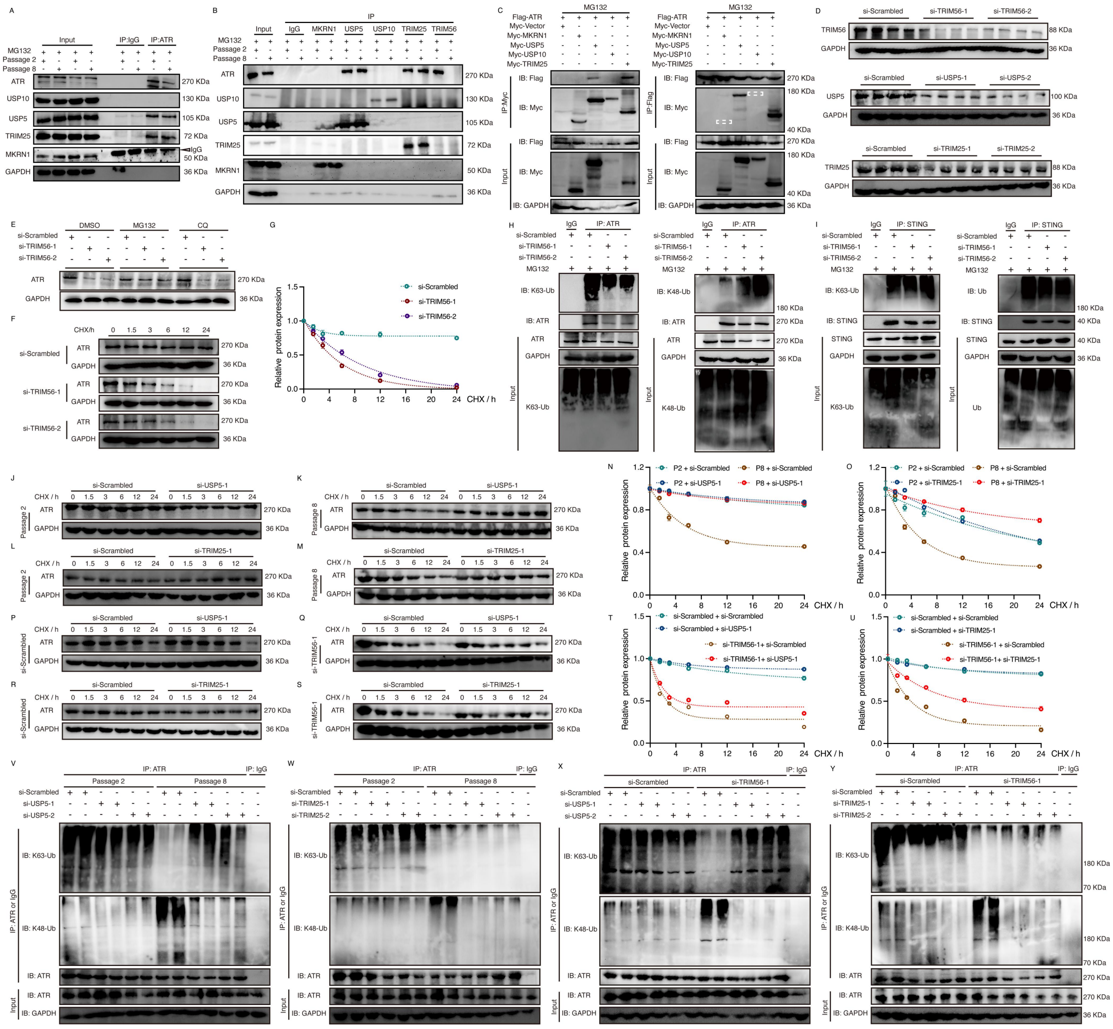

A) Endogenous Co-IP analysis showing the interaction of ATR and other E3 ligases/USPs in P2 and P8 NP cells using anti-ATR antibody. **B**) Endogenous Co-IP analysis showing the interaction of other E3 ligases/USPs and ATR in normal and senescent NP cells using anti-E3 ligases/USPs (TRIM25, MKRN1, USP5 and USP10) antibodies. **C**) Exogenous forward and reverse Co-IP analysis of the interaction of ATR and other E3 ligases/USPs in HEK293T cells cotransfected with Flag-tagged ATR and Myc-tagged other E3 ligases/USPs (TRIM25, MKRN1, USP5 and USP10) plasmids. **D**) Representative western blotting analysis performed determining the efficiency of TRIM56, USP5 and TRIM25 expression knockdown in P2 NP cells after treated with specific siRNAs targeting TRIM56, USP5 and TRIM25 (si-TRIM56, si-USP5 and si-TRIM25) (n = 4 biological replicates). **E**) Representative western blotting images showing endogenous ATR expression in siRNA-induced TRIM56-deficient P2 NP cells treated with 100 µg/ml CHX and 10 µM MG132 or 50 µM CQ for 12 h.

F and **G**) Representative western blotting images **F**) and half-life analysis **G**) showing endogenous ATR protein levels in siRNA-induced TRIM56-deficient P2 NP cells treated with 100 μg/ml CHX at different time points (n = 3 biological replicates). **H**) Endogenous Co-IP assay of K48-linked and K63-linked ubiquitination of ATR in siRNA-induced TRIM56-deficient P2 NP cells. **I**) Endogenous Co-IP assay of nonspecific and K63-linked ubiquitination of STING in siRNA-induced TRIM56-deficient P2 NP cells. **J** and **K**, **N**) Representative western blotting images **J** and **K**) and half-life analysis **N**) showing endogenous ATR protein levels in siRNA-induced USP5-deficient P2 and P8 NP cells treated with 100 μg/ml CHX at different time points (n = 3 biological replicates). **L** and **M**, **O**) Representative western blotting images **L** and **M**) and half-life analysis **O**) showing endogenous ATR protein levels in siRNA-induced TRIM25-deficient P2 and P8 NP cells treated with 100 μg/ml CHX at different time points (n = 3 biological replicates). **P** and **Q**, **T**) Representative western blotting images **P** and **Q**) and half-life analysis **T**) showing endogenous ATR protein levels in siRNA-induced TRIM56-deficient P2 NP cells after transfected with si-USP5 and treated with 100 μg/ml CHX at different time points (n = 3 biological replicates). **R** and **S**, **U**) Representative western blotting images **R** and **S**) and half-life analysis **U**) showing endogenous ATR protein levels in siRNA-induced TRIM56-deficient P2 NP cells after transfected with si-TRIM25 and treated with 100 μg/ml CHX at different time points (n = 3 biological replicates). **V**) Endogenous Co-IP assay of K48-linked and K63-linked ubiquitination of ATR in siRNA-induced USP5-deficient P2 and P8 NP cells. **W**) Endogenous Co-IP assay of K48-linked and K63-linked ubiquitination of ATR in siRNA-induced TRIM25-deficient P2 and P8 NP cells. **X**) Endogenous Co-IP assay of K48-linked and K63-linked ubiquitination of ATR in siRNA-induced TRIM56-deficient P2 NP cells after transfected with si-USP5. **Y**) Endogenous Co-IP assay of K48-linked and K63-linked ubiquitination of ATR in siRNA-induced TRIM56-deficient P2 NP cells after transfected with si-TRIM25. At least 3 independent experiments were performed.

Figure S6. Cavin-2/lipid raft-dependent endocytosis of EVs was sufficient for the effect exhibition of ATR-engineered EVs in alleviating NP cell senescence

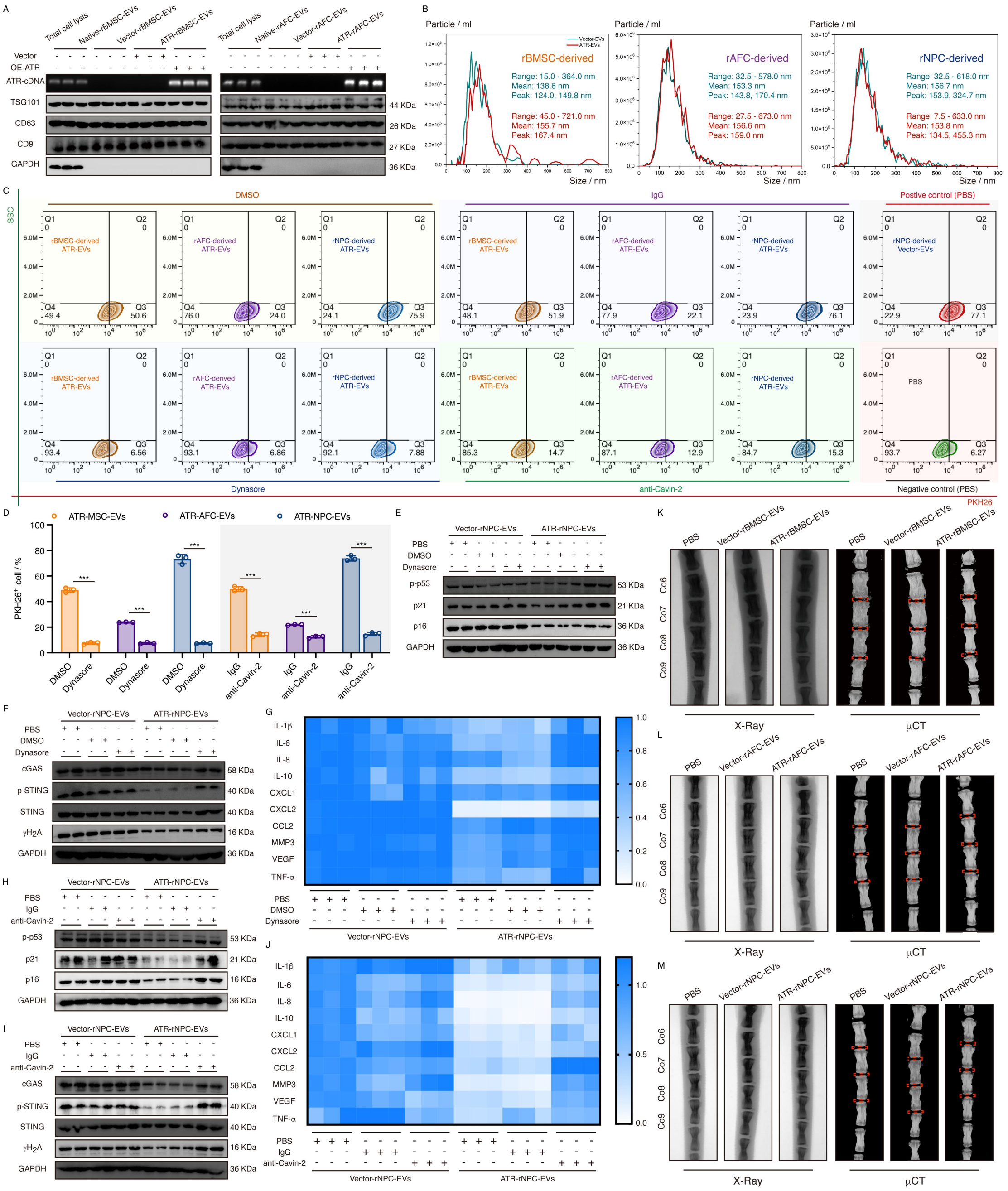

A) Western blotting analysis of EV markers in ATR-EVs or vector-EVs and qPCR followed by DNA agarose gel electrophoresis to determine the efficiency of loading of the ATR-overexpressing plasmid into rBMSC- and rAF-derived EVs. **B**) NTA of ATR-EVs or vector-EVs derived from different cell sources. **C** and **D**) Representative images **C**) and quantitative analysis **D**) of flow cytometry showing the PKH26-labelled EV uptake of rNP P8 cells with different indicated treatments (n = 3 biological replicates). **E**) Representative western blotting images of p-p53, p21 and p16 in P8 rat NP cells after

cocultured with ATR-EVs or vector-EVs and Dynasore (50 μM) or DMSO for 72 h (n = 2 biological replicates). **F**) Representative western blotting images of cGAS, STING, p-STING and γH_2A in P8 rat NP cells after cocultured with ATR-EVs or vector-EVs and Dynasore or DMSO for 72 h (n = 2 biological replicates). **G**) Differential heatmap of SASP expression in P8 rat NP cells after cocultured with ATR-EVs or vector-EVs and Dynasore or DMSO for 72 h (n = 3 biological replicates). **H**) Representative western blotting images of p-p53, p21 and p16 in P8 rat NP cells after cocultured with ATR-EVs or vector-EVs and anti-Cavin-2 (100 ng/ml) or isotype IgG (100 ng/ml) antibody for 72 h (n = 2 biological replicates). **I**) Representative western blotting images of cGAS, STING, p-STING and γH_2A in P8 rat NP cells after cocultured with ATR-EVs or vector-EVs and anti-Cavin-2 or isotype IgG antibody for 72 h (n = 2 biological replicates). **J**) Differential heatmap of SASP expression in P8 rat NP cells after cocultured with ATR-EVs or vector-EVs and anti-Cavin-2 or isotype IgG antibody for 72 h (n = 3 biological replicates). **K**) Representative X-ray images and μCT images of coccygeal vertebrae and IVDs from rats treated with the intradisc injection of ATR-EVs or vector-EVs (n = 5). Unpair students' *t* test (**D**) was used to performed, and data are represented as mean ± SEM. At least 3 independent experiments were performed. * p <0.05; ** p <0.01; *** p <0.001; n.s. not significant.

Supplementary Table 1. Characteristics details of the volunteers for western blotting analysis, immunohistochemistry and telomere length analysis

Case No.	Age (Years)	Gender	Diagnosis	Disc level	Pfirrmann MRI grade
Case 1	42	Male	Lumbar disc herniation	L5-S1	III
Case 2	15	Female	Idiopathic scoliosis	T12-L1	I
Case 3	32	Female	Lumbar disc herniation	L5-S1	II
Case 4	46	Female	Lumbar disc herniation	L5-S1	IV
Case 5	37	Male	Lumbar disc herniation	L5-S1	IV
Case 6	35	Female	Lumbar disc herniation	L5-S1	III
Case 7	14	Male	Idiopathic scoliosis	T12-L1	I
Case 8	18	Female	Idiopathic scoliosis	L1-L2	I
Case 9	55	Male	Lumbar disc herniation	L4-L5	IV
Case 10	28	Female	Lumbar disc herniation	L5-S1	III
Case 11	18	Female	Lumbar disc herniation	L5-S1	II
Case 12	47	Male	Lumbar disc herniation	L5-S1	IV
Case 13	22	Female	Idiopathic scoliosis	T12-L1	I
Case 14	32	Male	Lumbar vertebral fracture	L3-L4	I
Case 15	56	Female	Lumbar disc herniation	L5-S1	IV
Case 16	31	Female	Lumbar disc herniation	L5-L5	IV
Case 17	37	Male	Lumbar disc herniation	L4-L5	II
Case 18	43	Male	Lumbar disc herniation	L4-L5	II
Case 19	17	Male	Lumbar disc herniation	L5-S1	III
Case 20	27	Female	Lumbar disc herniation	L5-S1	III
Case 21	38	Male	Lumbar disc herniation	L4-L5	III
Case 22	45	Male	Lumbar disc herniation	L5-S1	II
Case 23	15	Male	Idiopathic scoliosis	L2-L3	I
Case 24	31	Female	Lumbar disc herniation	L4-L5	II

Supplementary Table 2. Characteristics details of the volunteers for transcriptional RNA sequencing

Case No.	Age (Years)	Gender	Diagnosis	Disc level	Pfirrmann MRI grade
Case 1	18	Male	Idiopathic scoliosis	T12-L1	I
Case 2	29	Male	Lumbar vertebral fracture	L2-L3	I
Case 3	15	Female	Idiopathic scoliosis	L1-L2	I
Case 4	48	Female	Lumbar disc herniation	L5-S1	IV
Case 5	34	Male	Lumbar disc herniation	L5-S1	IV
Case 6	39	Female	Lumbar disc herniation	L4-L5	IV

Supplementary Table 3. Characteristics details of the age- and sex-matched case pairs for telomere length analysis

Pair	Age	Gender	Diagnosis	Segment	MRI grade	Diagnosis	Segment	MRI grade
P1	17	Female	Idiopathic scoliosis	L1-L2	I	Lumbar disc herniation	L4-L5	II
P2	18	Male	Idiopathic scoliosis	T12-L1	I	Lumbar spinal stenosis	L5-S1	III
P3	18	Female	Idiopathic scoliosis	T12-L1	I	Lumbar disc herniation	L5-S1	II
P4	28	Female	Idiopathic scoliosis	L1-L2	I	Lumbar disc herniation	L4-L5	IV
P5	22	Male	Idiopathic scoliosis	L1-L2	I	Lumbar disc herniation	L4-L5	III
P6	29	Female	Idiopathic scoliosis	L1-L2	I	Lumbar spinal stenosis	L5-S1	IV
P7	19	Female	Idiopathic scoliosis	T12-L1	I	Lumbar spinal stenosis	L4-L5	III
P8	15	Male	Idiopathic scoliosis	L1-L2	I	Lumbar spondylolisthesis	L5-S1	V
P9	42	Female	Idiopathic scoliosis	L2-L3	I	Lumbar disc herniation	L5-S1	IV
P10	34	Male	Idiopathic scoliosis	L1-L2	I	Lumbar disc herniation	L4-L5	IV

Supplementary Table 4. Characteristics details of the volunteers for CUT&Tag sequencing

Case No.	Age (Years)	Gender	Diagnosis	Disc level	Pfirrmann MRI grading
Case 1	19	Male	Idiopathic scoliosis	L1-L2	I
Case 2	17	Male	Idiopathic scoliosis	L1-L2	I
Case 3	26	Male	Idiopathic scoliosis	L2-L3	I
Case 4	19	Male	Lumbar disc herniation	L5-S1	III
Case 5	17	Male	Lumbar disc herniation	L4-L5	IV
Case 6	21	Male	Lumbar disc herniation	L5-S1	IV

Supplementary Table 5. Characteristics details of the volunteers for Co-IP analysis

Case No.	Age (Years)	Gender	Diagnosis	Disc level	Pfirrmann MRI grading
Case 1	19	Female	Idiopathic scoliosis	L1-L2	I
Case 2	20	Female	Idiopathic scoliosis	L2-L3	I
Case 3	28	Female	Lumbar disc herniation	L5-S1	III
Case 4	31	Female	Lumbar disc herniation	L4-L5	III
Case 5	26	Male	Idiopathic scoliosis	T12-L1	I
Case 6	29	Female	Idiopathic scoliosis	L1-L2	I
Case 7	21	Male	Lumbar disc herniation	L5-S1	III
Case 8	30	Female	Lumbar disc herniation	L4-L5	IV
Case 9	18	Male	Idiopathic scoliosis	T12-L1	I
Case 10	29	Male	Idiopathic scoliosis	L1-L2	I
Case 11	28	Male	Lumbar disc herniation	L5-S1	IV
Case 12	17	Male	Lumbar disc herniation	L5-S1	V

Supplementary Table 6. Primer sequences used in PCR genotyping

Gene	Forward (5'-3')	Reverse (5'-3')
Homo IL-1β	GGTTGAGTTTAAGCCAATCCA	TGCTGACCTAGGCTTGATGA
Homo IL-6	GCCCAGCTATGAACTCCTTCT	GAAGGCAGCAGGCAACAC
Homo IL-8	AGACAGCAGAGCACACAAGC	ATGGTTCCTTCCGGTGGT
Homo IL-10	TGCCTTCAGCAGAGTGAAGA	GCTTGGCAACCCAGGTAA
Homo CXCL1	GCTGAACAGTGACAAATCCAAC	CTTCAGGAACAGCCACCAGT
Homo CXCL2	CCCATGGTTAAGAAAATCATCG	CTTCAGGAACAGCCACCAAT
Homo CCL2	AGTCTCTGCCGCCCTTCT	GTGACTGGGGCATTGATTG
Homo TNF-α	CAGCCTCTTCTCCTTCCTGAT	GCCAGAGGGCTGATTAGAGA
Homo VEGF	AGGGCAGAATCATCACGAAGT	AGGGTCTCGATTGGATGGCA
Homo MMP3	CAAAACATATTTCTTTGTAGAGGACAA	TTCAGCTATTTGCTTGGGAAA
Homo ATR	TGAAGACGGTGTGCTCATGC	GGGTTTAGAGACGAGCTGAG
Homo TRIM56	GTGGAGGCTGCCGAAGAAGC	GATTACCACACTATTCTGCTG
Homo GAPDH	CAAGAAGGTGAAGCAGG	TCAAAGGTGGAGGAGTGGGT
Homo g-LAD12	ACTCCTCCACCTTTGACGCT	AGTTGTCAGGGCCCTTTTTCTGA
Homo g-L1ORF1	AGAACGCCACAAAGATACTCCTCG	CTCTCTTCTGGCTTGTAGGGTTTCTG
Homo g-18S RNA	GTAACCCGTTGAACCCCATT	CCATCCAATCGGTAGTAGCG
Homo g-POLG1	CTGCCATCCCGTTTCGCAGGT	CTCCTTTCCGTCAACAGCTC
Rat g-ATR	CGACACCTGAGGTTGTTCCT	AAGTCTGAATGTGCGTGAGGT

Supplementary Table 7. siRNA sequences used in siRNA transfection and shRNA sequences used in vivo experiments

Sequence name	Sense (5'-3')	Antisense (5'-3')
Homo si-Scrambled	CAAGCCUUAGUGUUGGUUCACCUUU	AAAGGUGAACCAACACUAAGGCUUG
Homo si-ATR-1	CAACCUCCGUGAUGUUGCUUGAUUU	AAAUCAAGCAACAUCACGGAGGUUG
Homo si-ATR-2	CAGAGUACUUGUUGGGCCCACUUUA	UAAAGUGGGCCCAACAAGUACUCUG
Homo si-Scrambled	CCAAGGUGUGGACAUUAUAGGCGAA	UUCGCCUAUAAUGUCCACACCUUGG
Homo si-TRIM56-1	CCACGUGGAGGUGUACAAUAUGGAA	UUCCAUAUUGUACACCUCCACGUGG
Homo si-TRIM56-2	CAGCAGAAUAGUGUGGUAAUCUGUG	CACAGAUUACCACACUAUUCUGCUG
Homo si-Scrambled	CAGCUAUGAUACGGUCUAAAUCGUU	AACGAUUUAGACCGUAUCAUAGCUG
Homo si-cGAS-1	CAGCUUCUAAGAUGCUGUCAAAGUU	AACUUUGACAGCAUCUUAGAAGCUG
Homo si-cGAS-2	CCUUGUACCCAAGCAUGCAAAGGAA	UUCCUUUGCAUGCUUGGGUACAAGG
Homo si-Scrambled	CAGCUAAGAUAAUAGCUGCGCCAAA	UUUGGCGCAGCUAUUAUCUUAGCUG
Homo si-AIM2-1	CAGCCAUCAGAAAUGAUGUCGCAAA	UUUGCGACAUCAUUUCUGAUGGCUG
Homo si-AIM2-2	CGCCUCACGUGUGUUAGAUGCUGAA	UUCAGCAUCUAACACACGUGAGGCG
Homo si-Scrambled	CGUUCGGCGUUGUGGAACAAUUGUA	UACAAUUGUUCCACAACGCCGAACG
Homo si-USP5-1	CGUUUCUGGGCUUUGGGAAACAGUA	UACUGUUUCCCAAAGCCCAGAAACG
Homo si-USP5-2	CCUGAUGGAGCUGACGUGUACUCAU	AUGAGUACACGUCAGCUCCAUCAGG
Homo si-Scrambled	CAGAACAGAACAUAAGCACGUCAAA	UUUGACGUGCUUAUGUUCUGUUCUG
Homo si-TRIM25-1	CAGCUACAACAAGAAUACACGGAAA	UUUCCGUGUAUUCUUGUUGUAGCUG
Homo si-TRIM25-2	GAGACCACCUCGACAAGGAAGAUAA	UUAUCUUCCUUGUCGAGGUGGUCUG
Rat sh-Scrambled	CCGCGAGAAGAGGACUCUGUUAGAA	UUCUAACAGAGUCCUCUUCUCGCGG
Rat sh-TRIM56-1	CCGAUGAGCAGAACAGGGUCUUGAA	UUCAAGACCCUGUUCUGCUCAUCGG
Rat sh-TRIM56-2	ACCACCAUGGUAGAUGGCAAAUACU	AGUAUUUGCCAUCUACCAUGGUGGU

Supplementary Table 8. Antibody information

Antibody	Company	Catalog#	Application/ Dilution
anti-phosphorylated p53 (Ser15)	Abcam	ab278683	IHC (1:150); WB (1: 500)
anti-p21 (CDKN1A)	Cell Signaling Technology	2947	IHC (1:100); WB (1: 500)
anti-γH_2A	Abcam	ab81299	IHC (1:150); WB (1: 500); IF (1:250)
anti-53BP1	Abcam	ab175933	IHC (1:100)
anti-p16 (INK4a)	Affinity	AF5484	WB (1: 500)
anti-HP1γ	Abcam	ab213167	IF (1:250)
anti-cGAS	Cell Signaling Technology	83623	WB (1: 500)
anti-cGAS	Santa Cruz	sc-515802	IP (4 µg/1000 µg)
anti-AIM2	Proteintech	66902-1-Ig	IP (4 µg/1000 µg); WB (1: 1000)
anti-p-STING	Cell Signaling Technology	#50907	WB (1: 1000)
anti-STING	Proteintech	19851-1-AP	WB (1: 1000)
anti-p65	Cell Signaling Technology	#8242	CUT&Tag-seq (1:100)
anti-NLRP3	Cell Signaling Technology	#15101	IP (4 µg/1000 µg); WB (1: 500)
anti-ASC	Proteintech	10500-1-AP	WB (1: 500)
anti-pro caspase-1	Proteintech	22915-1-AP	WB (1: 500)
anti-cleaved caspase-1	Affinity	#AF4005	WB (1: 500)
anti-GSDMD	Abcam	ab219800	WB (1: 500)
anti-cleaved GSDMD	Abcam	ab215203	WB (1: 500)
anti-ATR	Cell Signaling Technology	13934	IP (5 µg/1000 µg); WB (1: 500)
anti-ATR	Santa Cruz	sc-515173	WB (1: 100); IF (1:50)
anti-TRIM56	Abcam	ab154862	IP (5 µg/1000 µg); WB (1: 500); IF (1:100)
anti-USP10	Cell Signaling Technology	#8501	IP (5 µg/1000 µg); WB (1: 1000)
anti-USP5	Proteintech	10473-1-AP	IP (5 µg/1000 µg); WB (1: 2000)
anti-TRIM25	Proteintech	12573-1-AP	IP (5 µg/1000 µg); WB (1: 500)
anti-MKRN1	Affinity	#DF13815	IP (5 µg/1000 µg); WB (1: 500)

anti-ubiquitin	Abcam	ab134953	WB (1: 500)
anti-ubiquitin (K48 linkage)	Abcam	ab140601	WB (1: 500)
anti-ubiquitin (K63 linkage)	Abcam	ab179434	WB (1: 500)
anti-Flag tagged epitope	Proteintech	66008-4-AP	IP (5 μg/1000 μg); WB (1: 500)
anti-Myc tagged epitope	Proteintech	16286-1-AP	IP (5 μg/1000 μg); WB (1: 500)
anti-HA tagged epitope	Proteintech	51064-2-AP	WB (1: 500)
anti-GAPDH	Proteintech	60004-1-Ig	WB (1: 2000)
anti-β-actin	Proteintech	66009-1-Ig	WB (1: 2000)
anti-Tubulin	Proteintech	66031-1-Ig	WB (1: 1000)
anti-PCNA	Proteintech	60097-1-Ig	WB (1: 1000)

Reference

1. Pfirrmann CW, Metzdorf A, Zanetti M, Hodler J, and Boos N. Magnetic resonance classification of lumbar intervertebral disc degeneration. *Spine.* 2001;26(17):1873-8.
2. Cheng X, Zhang L, Zhang K, Zhang G, Hu Y, Sun X, et al. Circular RNA VMA21 protects against intervertebral disc degeneration through targeting miR-200c and X linked inhibitor-of-apoptosis protein. *Annals of the rheumatic diseases.* 2018;77(5):770-9.
3. Kerur N, Fukuda S, Banerjee D, Kim Y, Fu D, Apicella I, et al. cGAS drives noncanonical-inflammasome activation in age-related macular degeneration. *Nature medicine.* 2018;24(1):50-61.
4. Chen S, Wu X, Lai Y, Chen D, Bai X, Liu S, et al. Kindlin-2 inhibits Nlrp3 inflammasome activation in nucleus pulposus to maintain homeostasis of the intervertebral disc. *Bone research.* 2022;10(1):5.
5. Wang D, Peng P, Dudek M, Hu X, Xu X, Shang Q, et al. Restoring the dampened expression of the core clock molecule BMAL1 protects against compression-induced intervertebral disc degeneration. *Bone research.* 2022;10(1):20.
6. Han Y, Zhou CM, Shen H, Tan J, Dong Q, Zhang L, et al. Attenuation of ataxia telangiectasia mutated signalling mitigates age-associated intervertebral disc degeneration. *Aging cell.* 2020;19(7):e13162.
7. Cawthon RM. Telomere measurement by quantitative PCR. *Nucleic acids research.* 2002;30(10):e47.
8. Chatzidoukaki O, Stratigi K, Goulielmaki E, Niotis G, Akalestou-Clocher A, Gkirtzimanaki K, et al. R-loops trigger the release of cytoplasmic ssDNAs leading to chronic inflammation upon DNA damage. *Science advances.* 2021;7(47):eabj5769.
9. Chung KW, Dhillon P, Huang S, Sheng X, Shrestha R, Qiu C, et al. Mitochondrial Damage and Activation of the STING Pathway Lead to Renal Inflammation and Fibrosis. *Cell metabolism.* 2019;30(4):784-99.e5.
10. Jauhari A, Baranov SV, Suofu Y, Kim J, Singh T, Yablonska S, et al. Melatonin inhibits cytosolic mitochondrial DNA-induced neuroinflammatory signaling in accelerated aging and neurodegeneration. *The Journal of clinical investigation.* 2020;130(6):3124-36.
11. Yu CH, Davidson S, Harapas CR, Hilton JB, Mlodzianoski MJ, Laohamonthonkul P, et al.

TDP-43 Triggers Mitochondrial DNA Release via mPTP to Activate cGAS/STING in ALS. *Cell.* 2020;183(3):636-49.e18.

12. Wu J, Chen Y, Liao Z, Liu H, Zhang S, Zhong D, et al. Self-amplifying loop of NF-κB and periostin initiated by PIEZO1 accelerates mechano-induced senescence of nucleus pulposus cells and intervertebral disc degeneration. *Molecular therapy : the journal of the American Society of Gene Therapy.* 2022.

13. Li G, Ma L, He S, Luo R, Wang B, Zhang W, et al. WTAP-mediated m(6)A modification of lncRNA NORAD promotes intervertebral disc degeneration. *Nature communications.* 2022;13(1):1469.

14. Guo M, Wu F, Hu G, Chen L, Xu J, Xu P, et al. Autologous tumor cell-derived microparticle-based targeted chemotherapy in lung cancer patients with malignant pleural effusion. *Science translational medicine.* 2019;11(474).

15. Ma L, Li G, Lei J, Song Y, Feng X, Tan L, et al. Nanotopography Sequentially Mediates Human Mesenchymal Stem Cell-Derived Small Extracellular Vesicles for Enhancing Osteogenesis. *ACS nano.* 2021.

16. Kanada M, Bachmann MH, Hardy JW, Frimannson DO, Bronsart L, Wang A, et al. Differential fates of biomolecules delivered to target cells via extracellular vesicles. *Proceedings of the National Academy of Sciences of the United States of America.* 2015;112(12):E1433-42.
