# Supplementary material for: Disassembly of the TRIM56-ATR complex promotes cytoDNA/cGAS/STING axis–dependent intervertebral disc inflammatory degeneration
Source: J Clin Invest. 2024 Jan 23;134(6):e165140. doi: 10.1172/JCI165140 (PMC10940101; doi:10.1172/JCI165140)

Figure 1E

γH2A

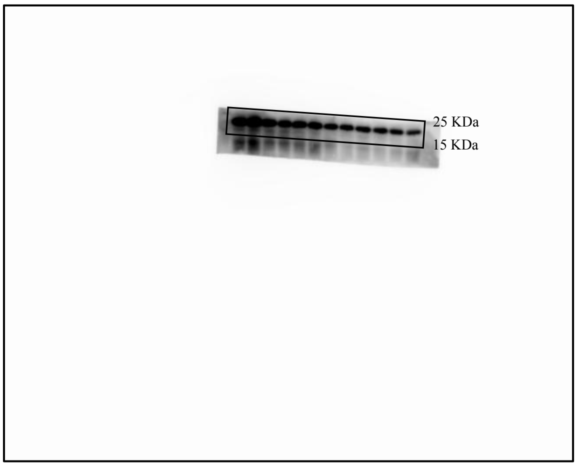

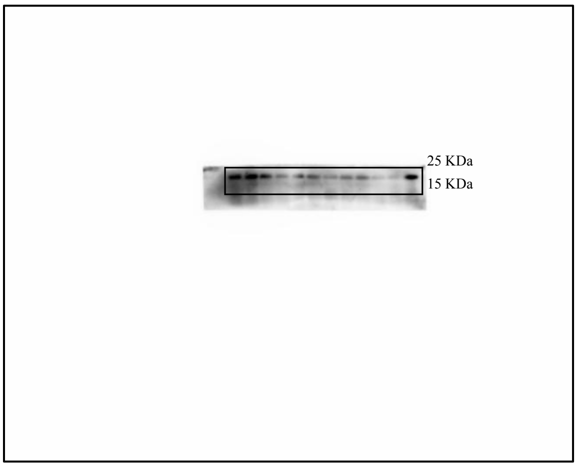

p-p53

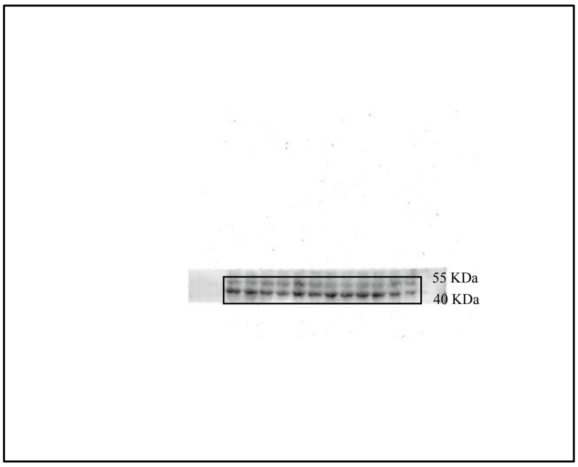

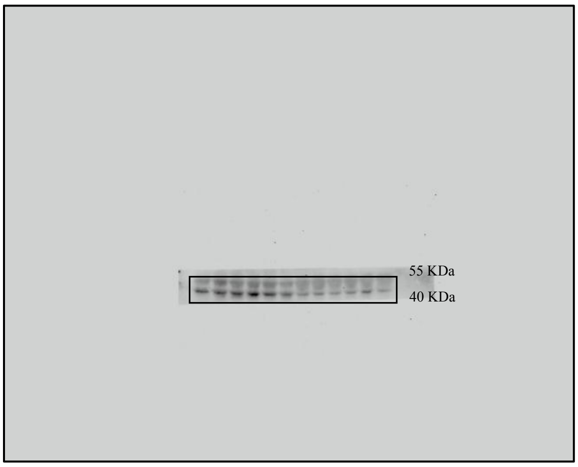

p21

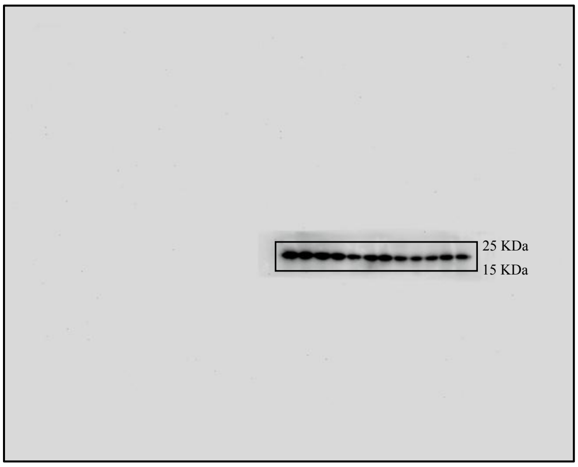

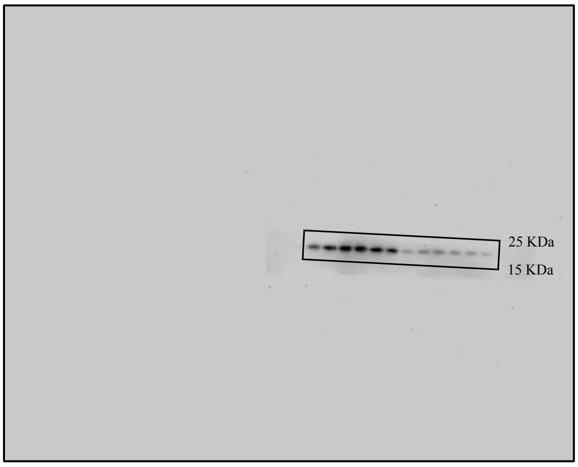

p16

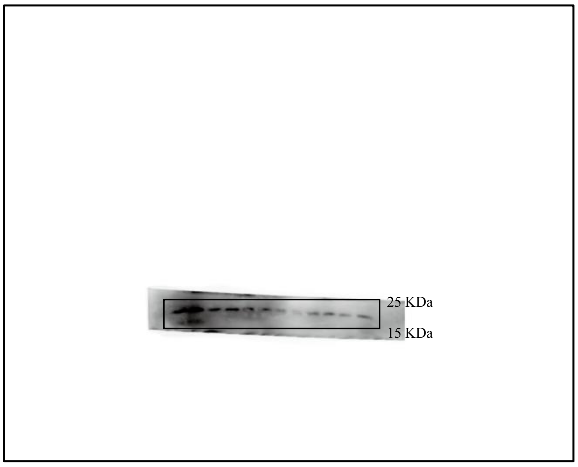

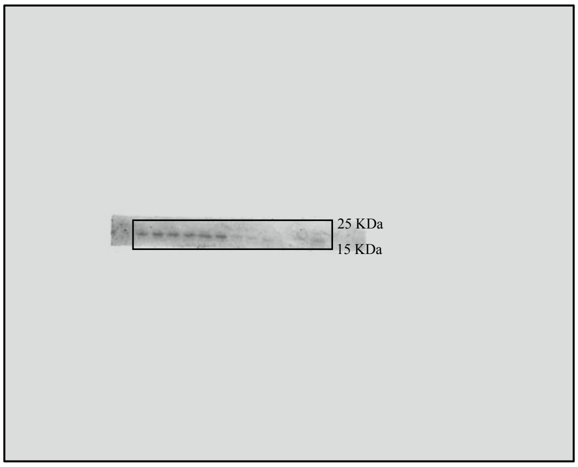

GAPDH

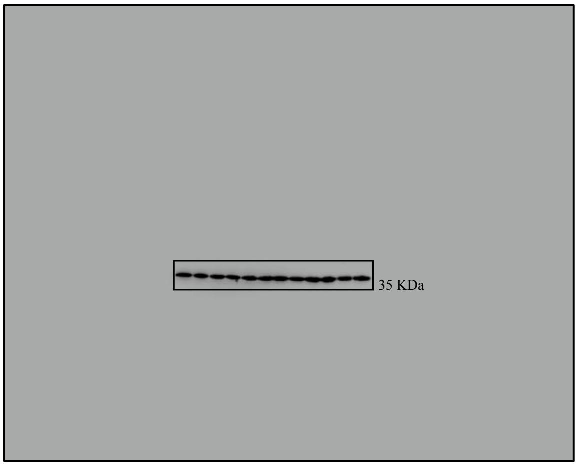

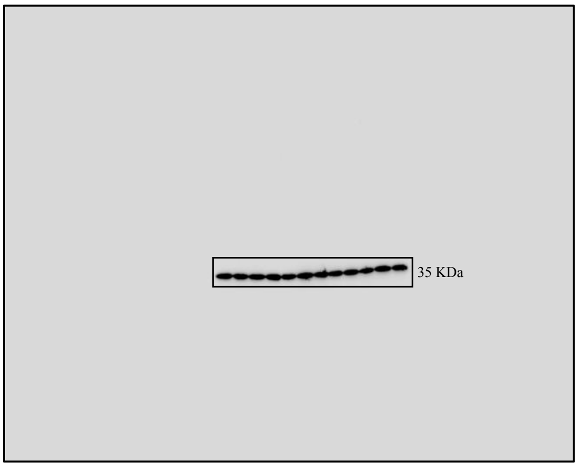
35 KDa

Figure 1G

p-p53

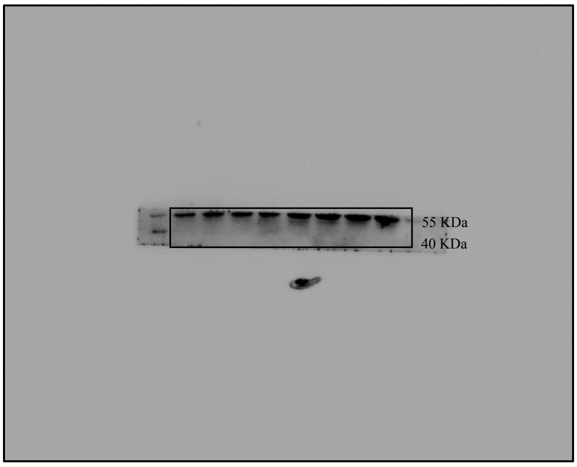

p21

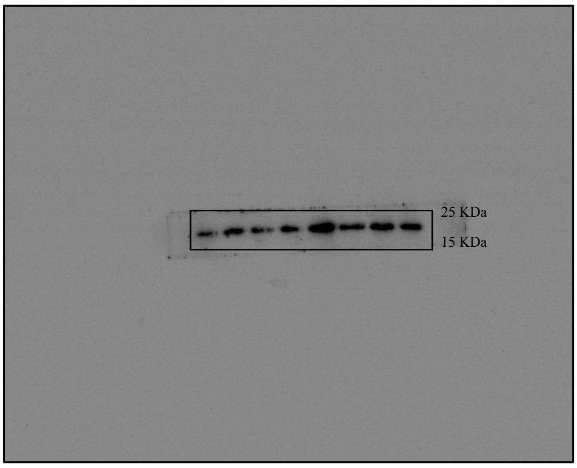

p16

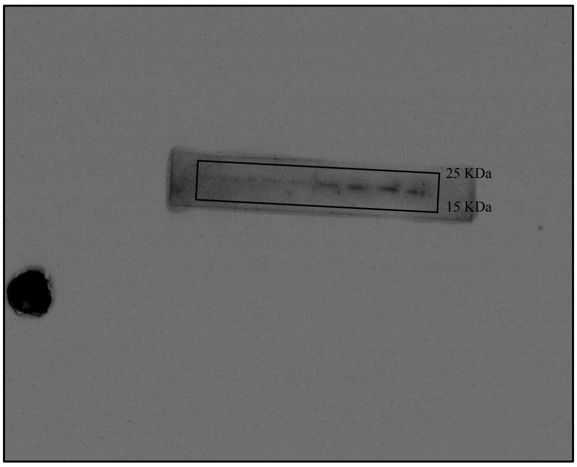

GAPDH

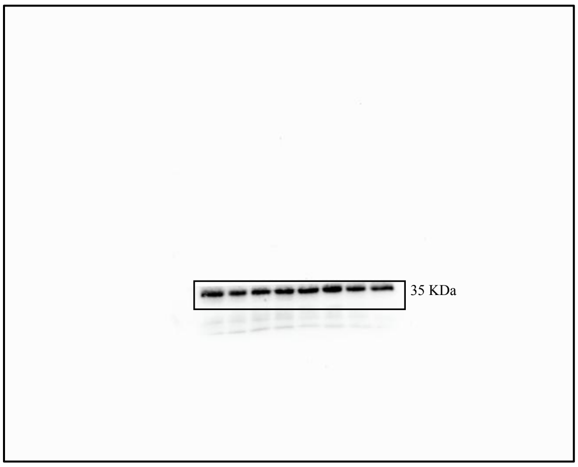

53BP

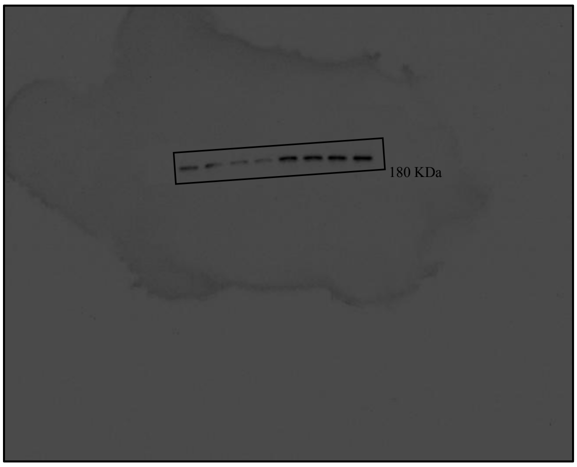

γH2A

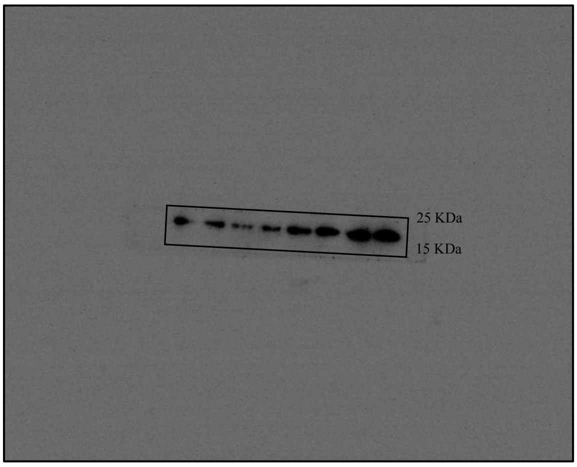

GAPDH

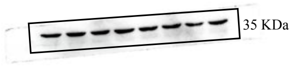
35 KDa

Figure 3H

p-p53 (Ser)

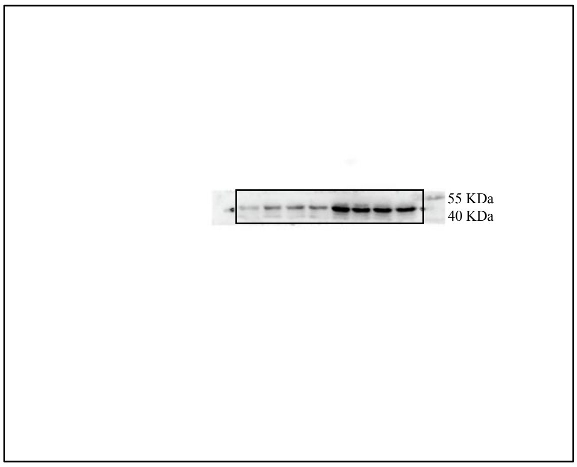

p21

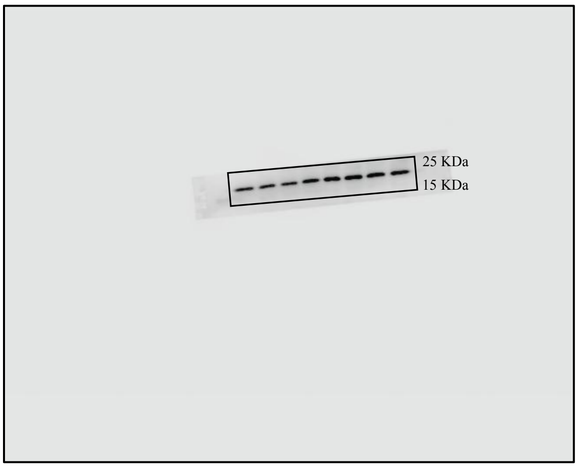

p16

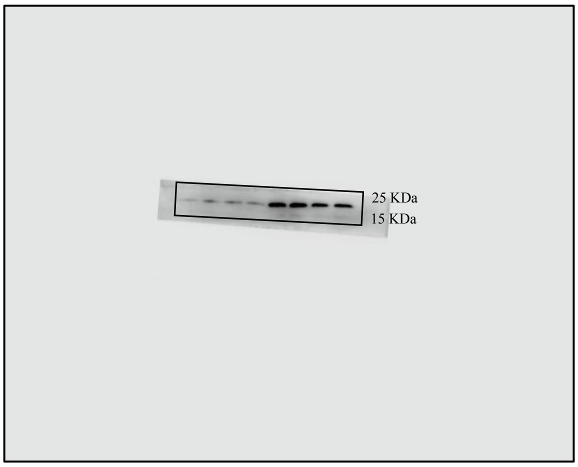

GAPDH

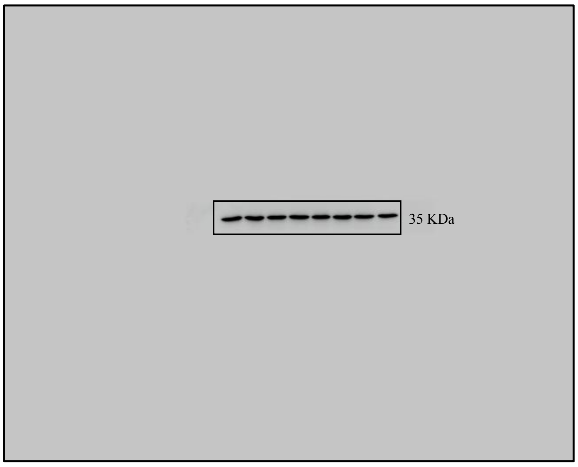
35 KDa

Figure 3I

53BP

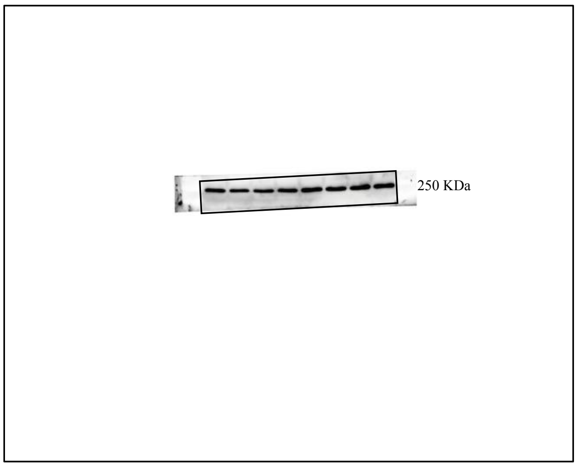

γH2A

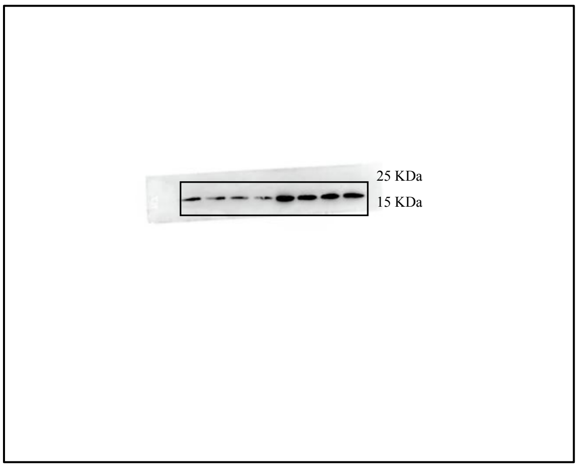

GAPDH

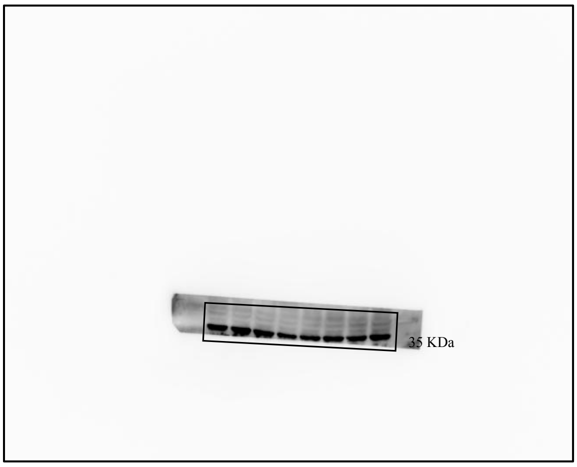

Figure 4B

gDNA: LAD12

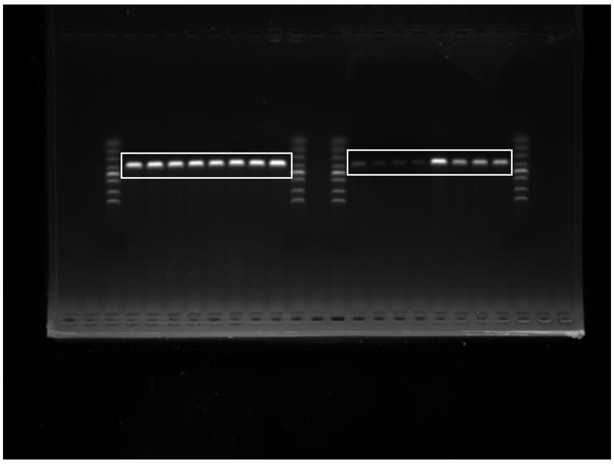

gDNA: 18S RNA

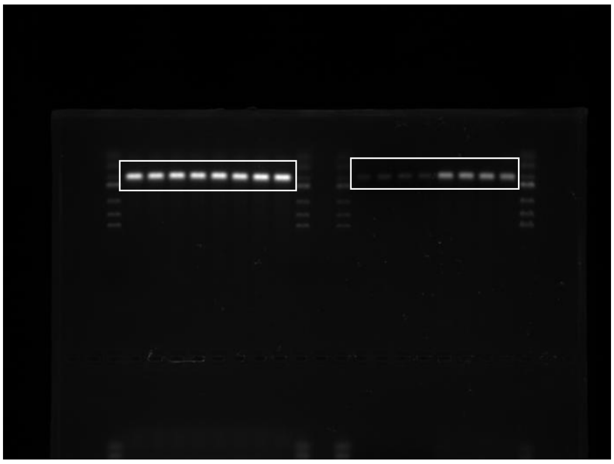

gDNA: POLG1; L1OFR1

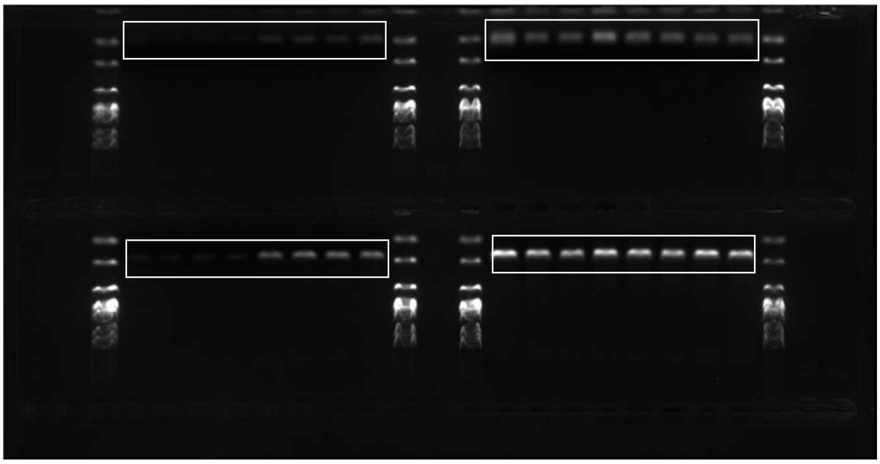

Figure 4E

cGAS

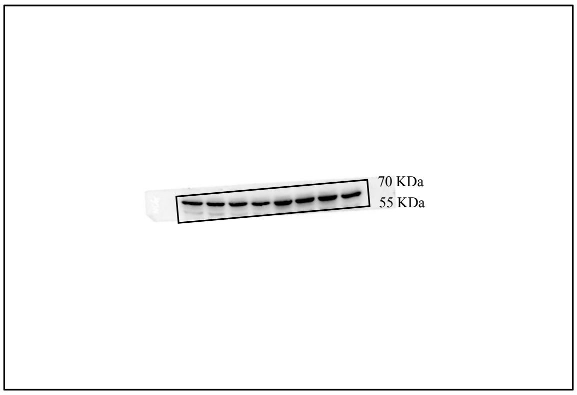

p-STING

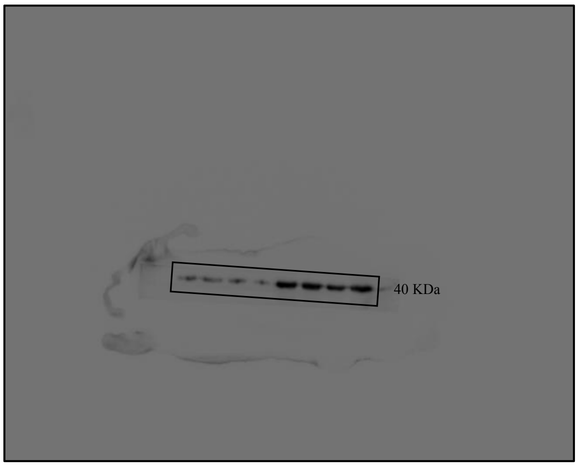

STING

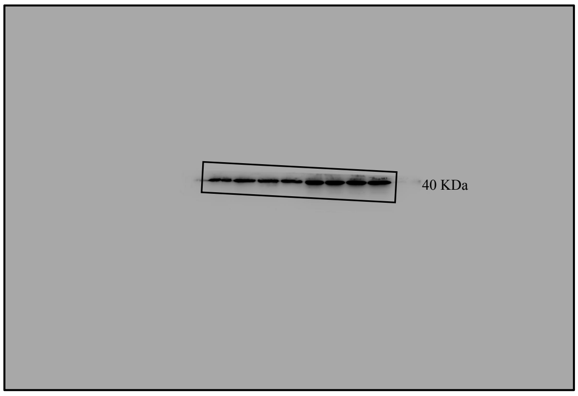

AIM2

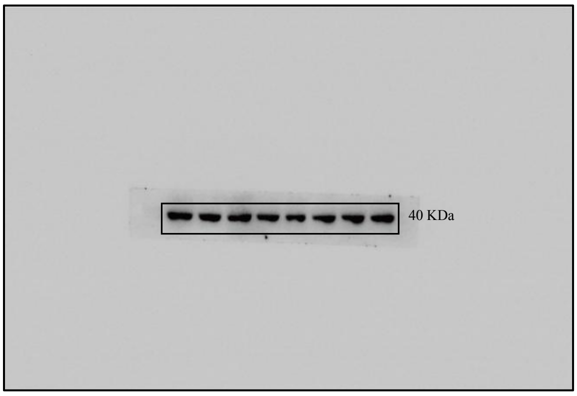

GAPDH

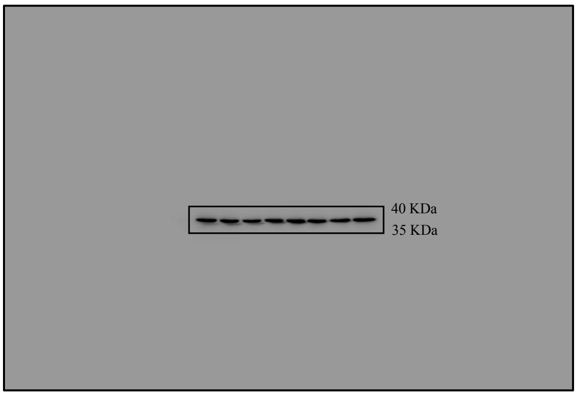

Figure 4I

cGAS

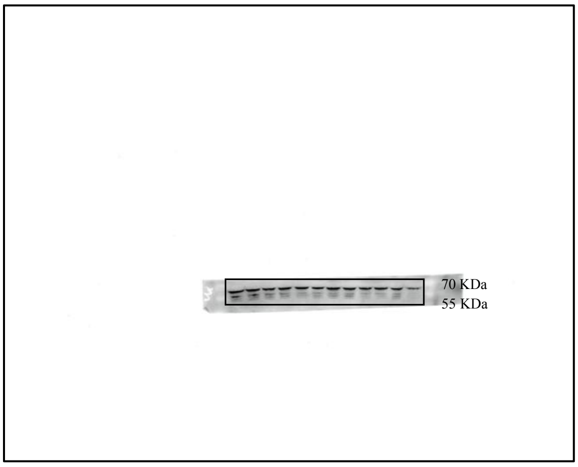

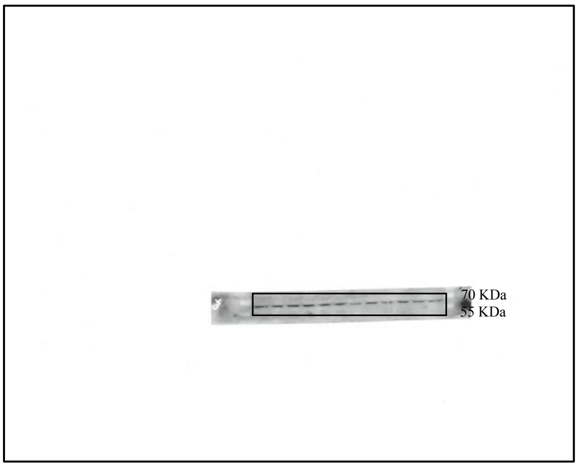

p-STING

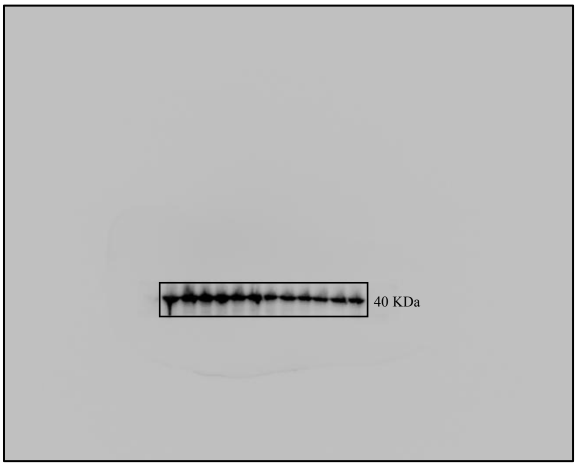

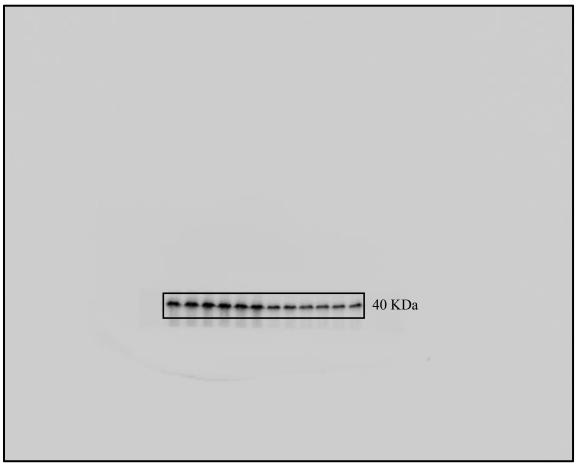

STING

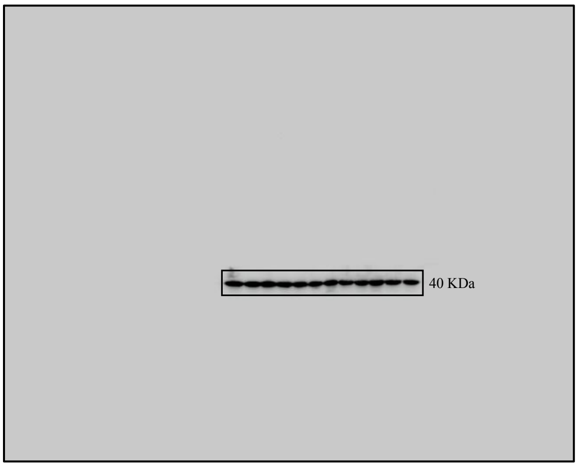

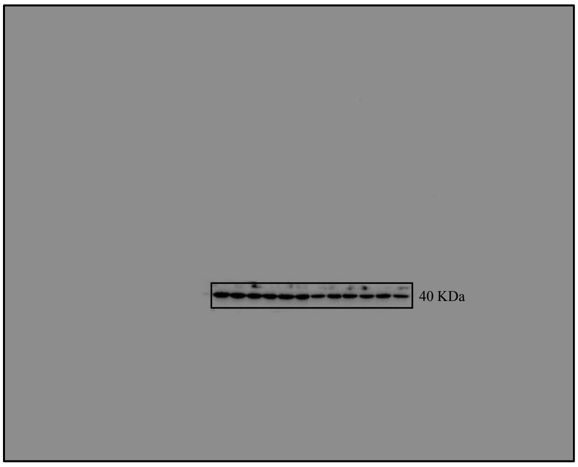

GAPDH

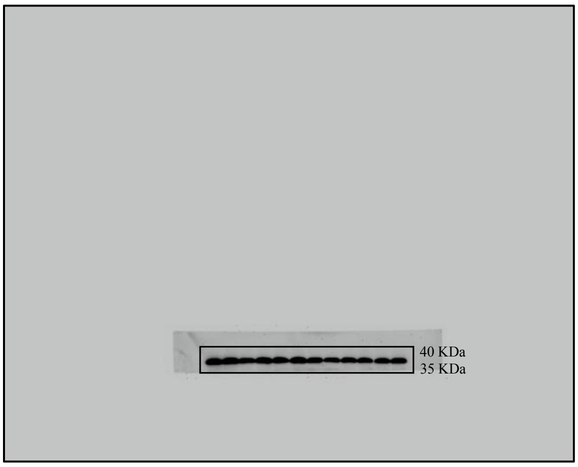
40 KDa
35 KDa

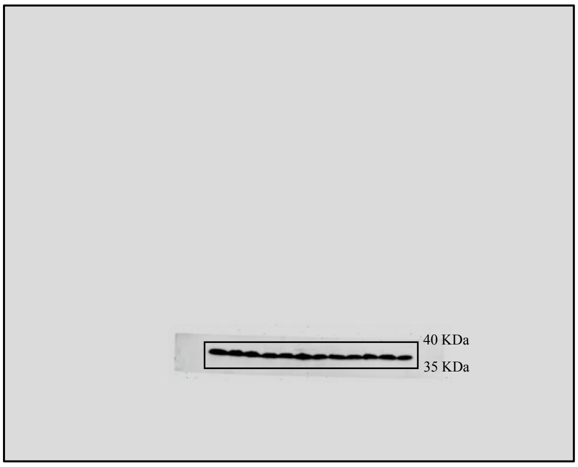
40 KDa
35 KDa

Figure 6B

ATR

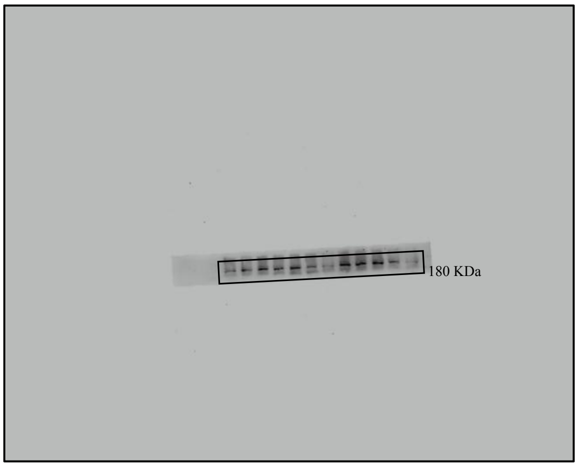

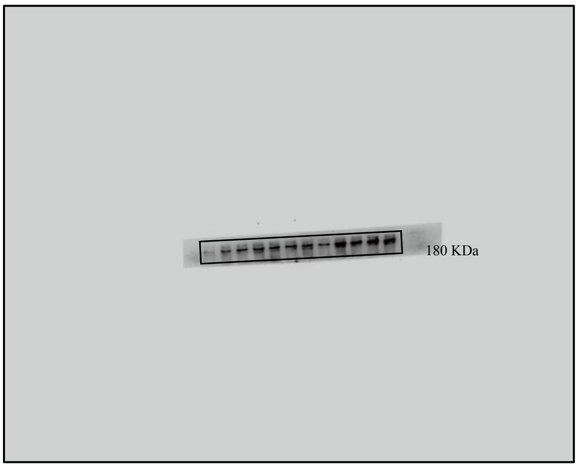

GAPDH

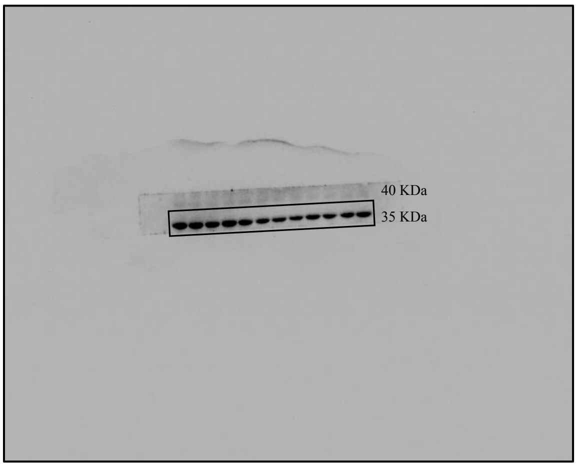

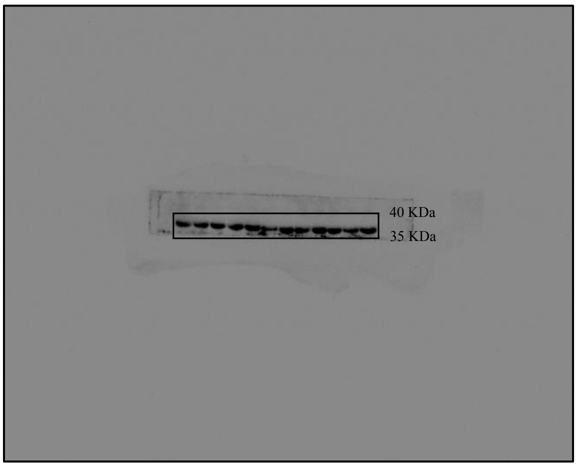
40 KDa
35 KDa

Figure 6C

ATR

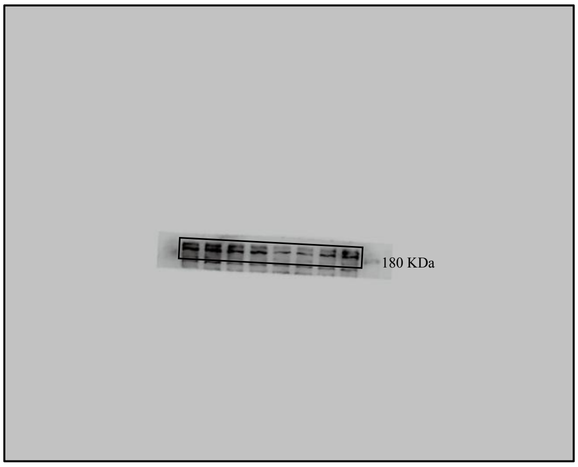

GAPDH

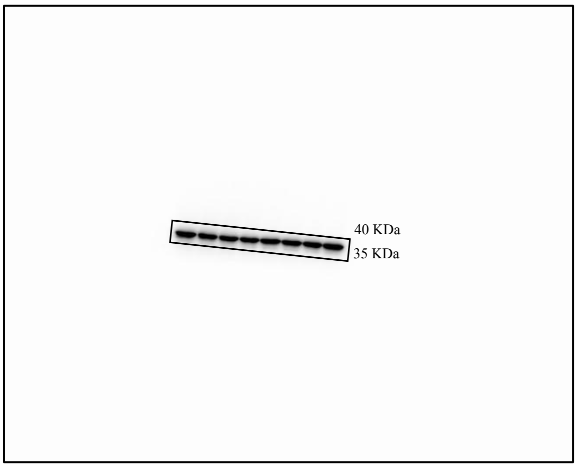

Figure 6H

p-p53

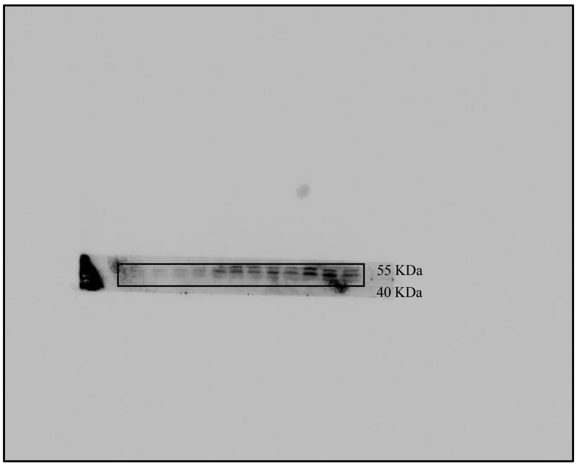

p21

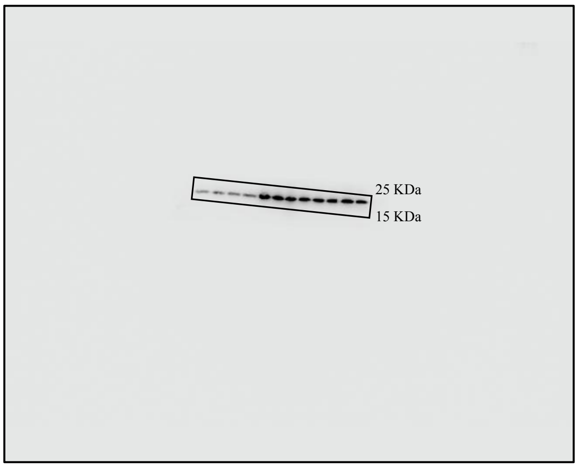

GAPDH

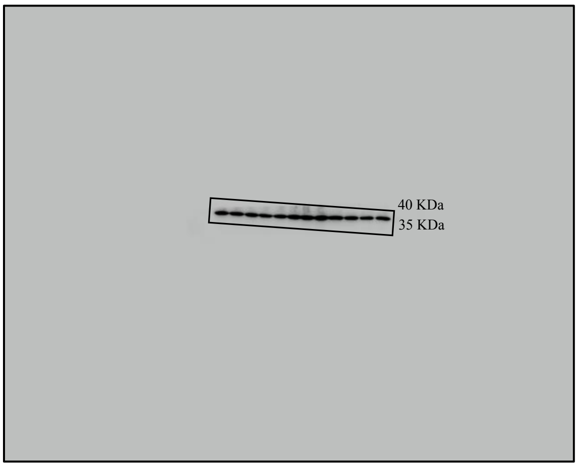

Figure 6I

cGAS

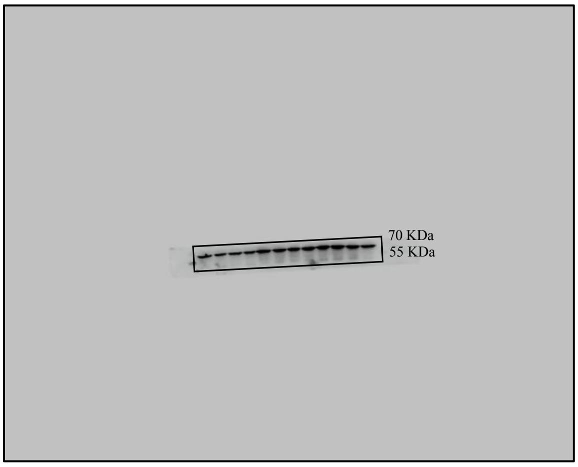

STING

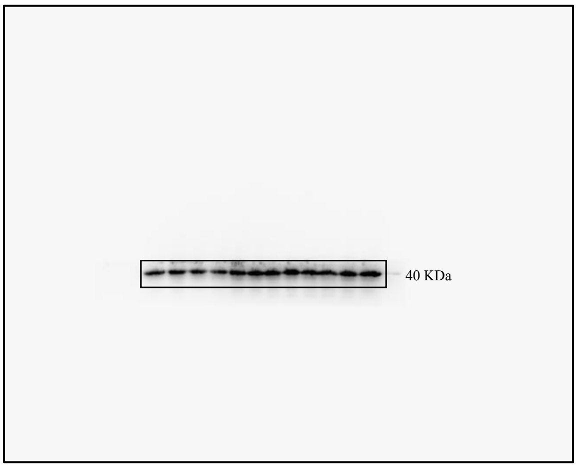

γH2A

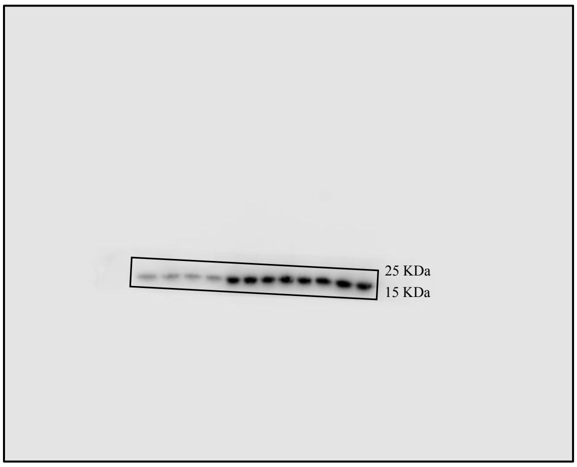

GAPDH

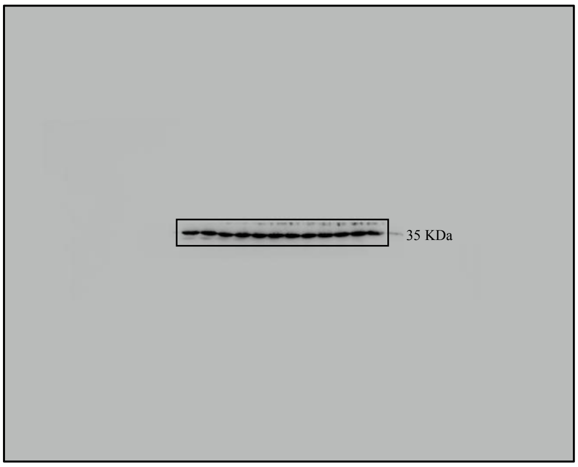
35 KDa

Figure 7D

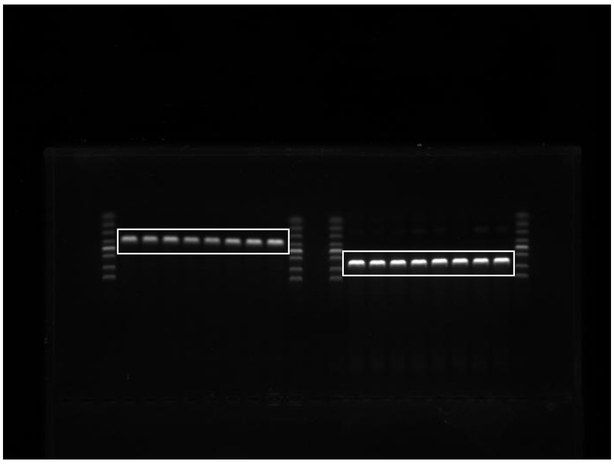

Figure 7E

ATR

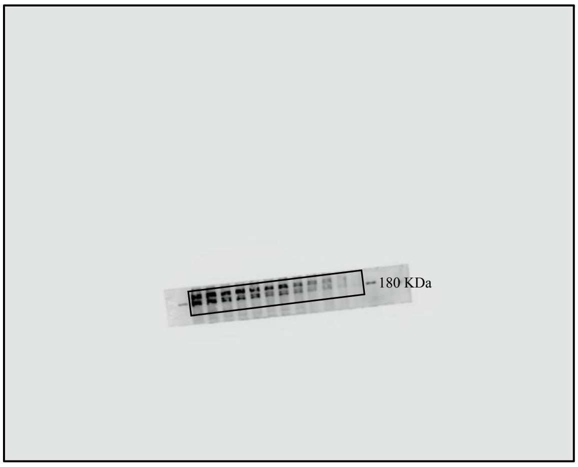

GAPDH

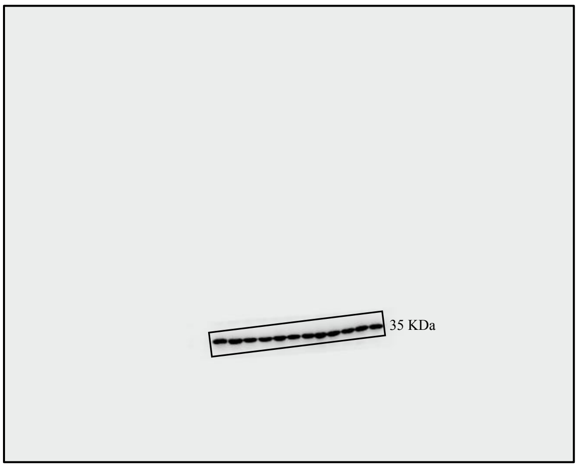

Figure 7F

ATR

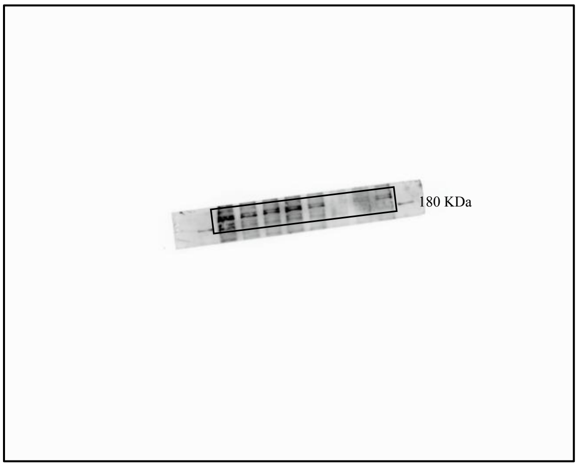

GAPDH

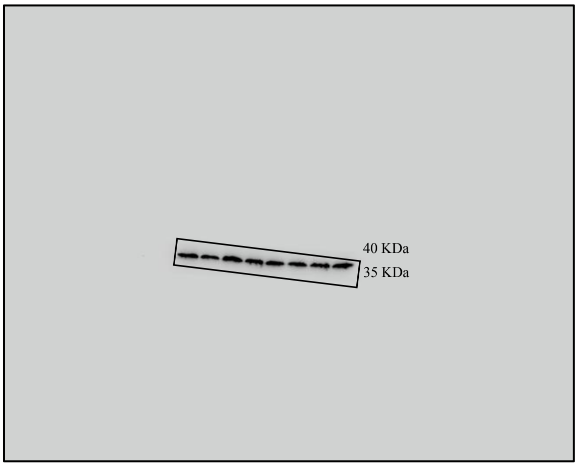

Figure 7G

IB: ATR

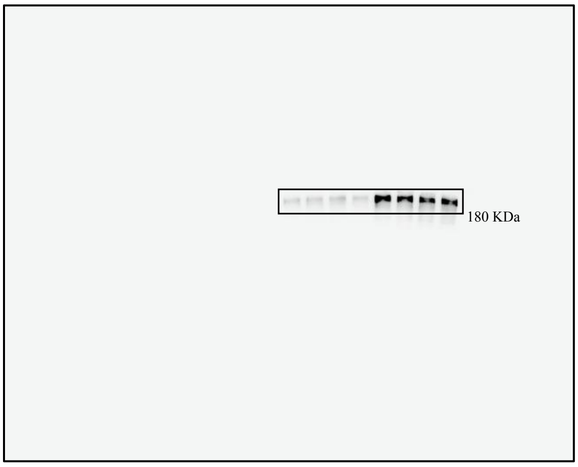

IB: Ub

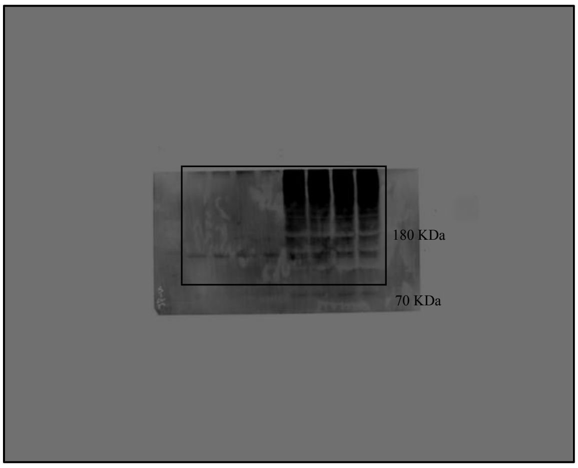

Input: ATR

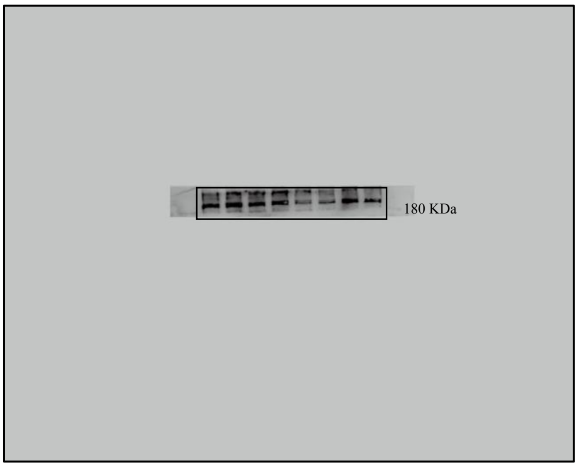

Input: Ub

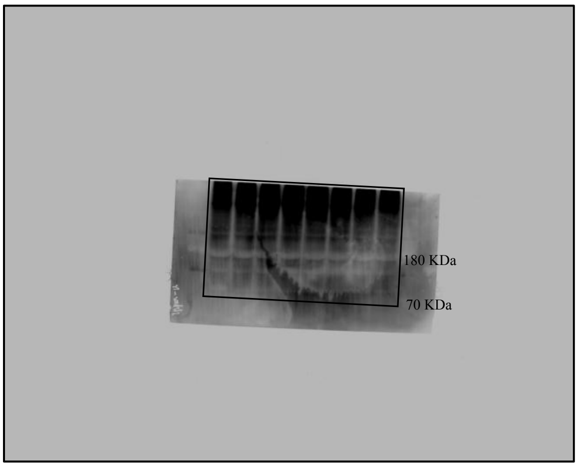

Input: GAPDH

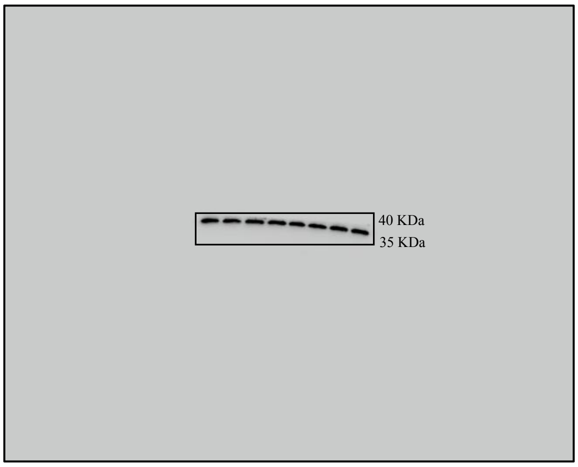

Figure 7H

IB: ATR

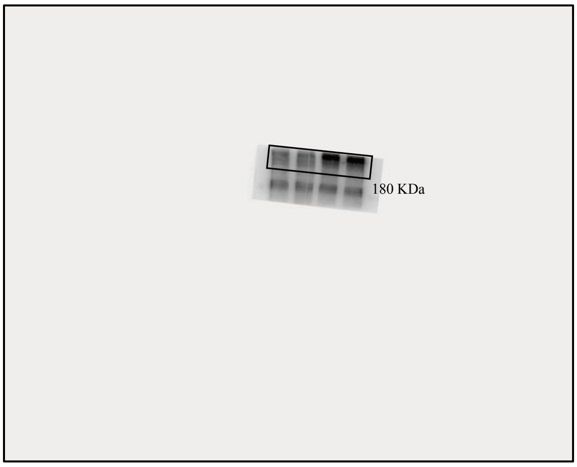

IB: K48-Ub

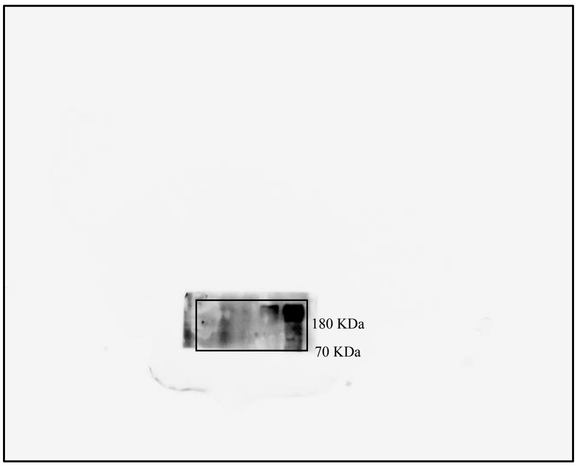

K63-Ub

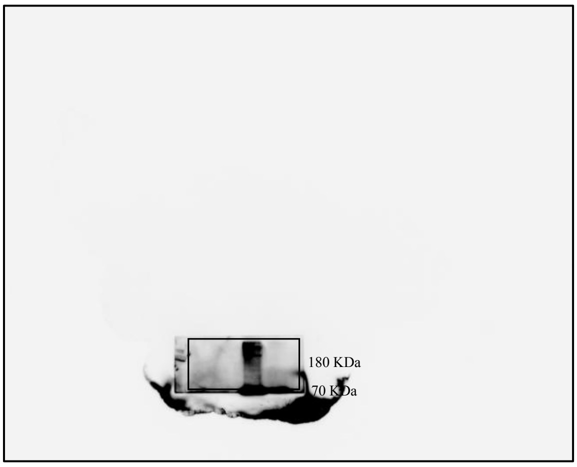

Input: ATR

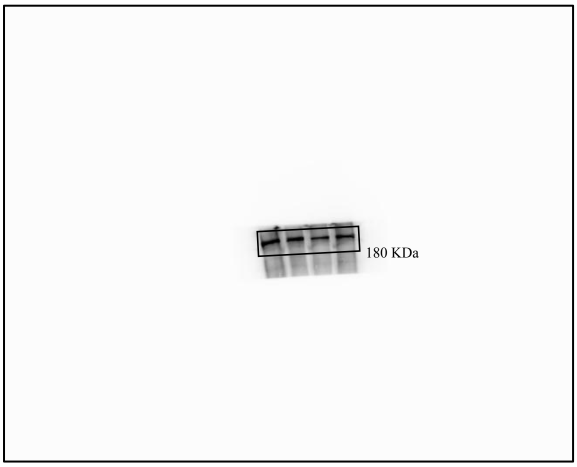

Input: K48-Ub

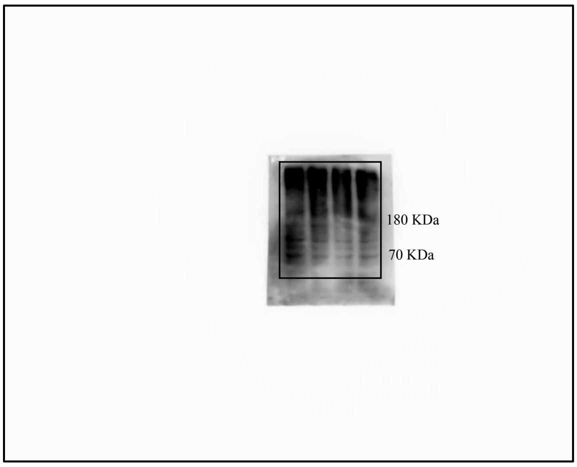

Input: K63-Ub

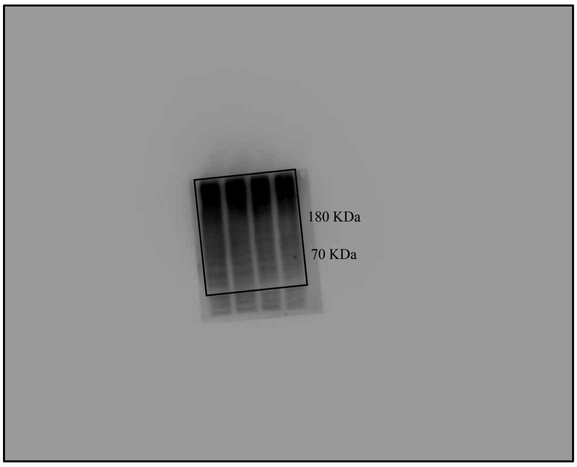

Input: GAPDH

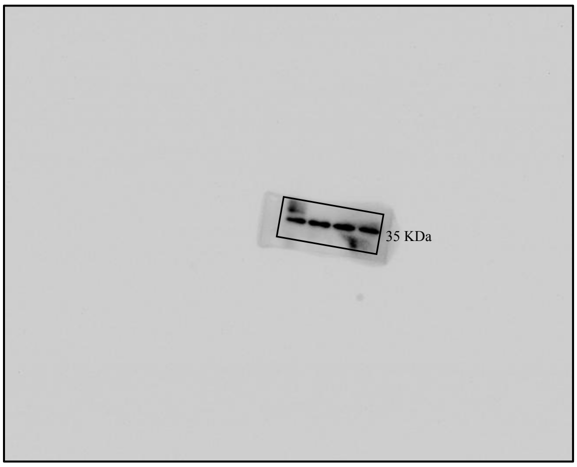
35 KDa

Figure 7I

ATR-1

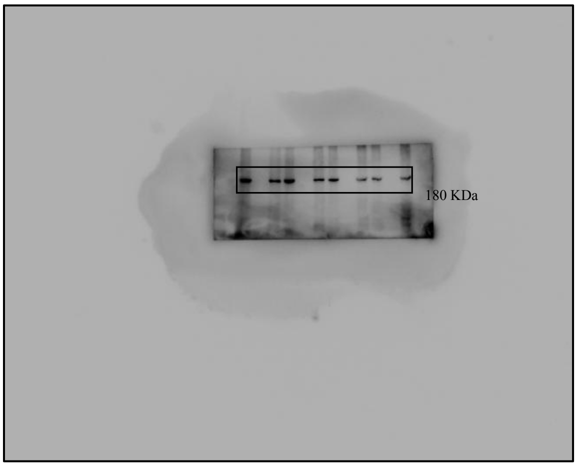

Ub-1

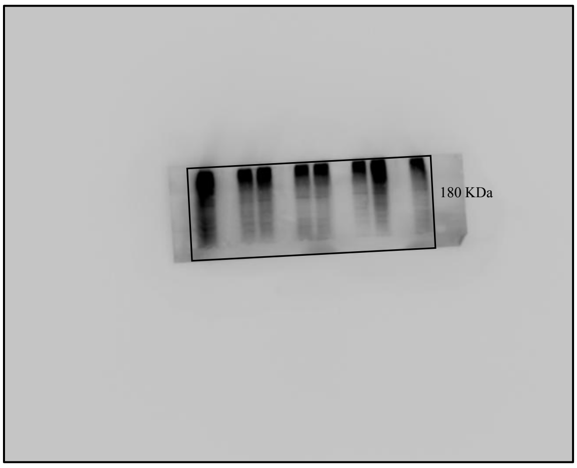

K63-Ub-1

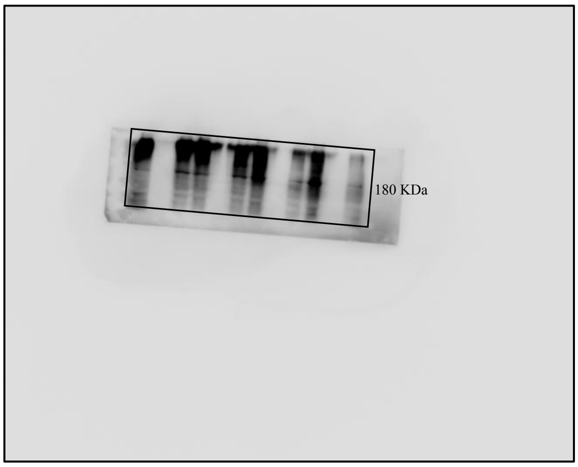

K48-Ub-1

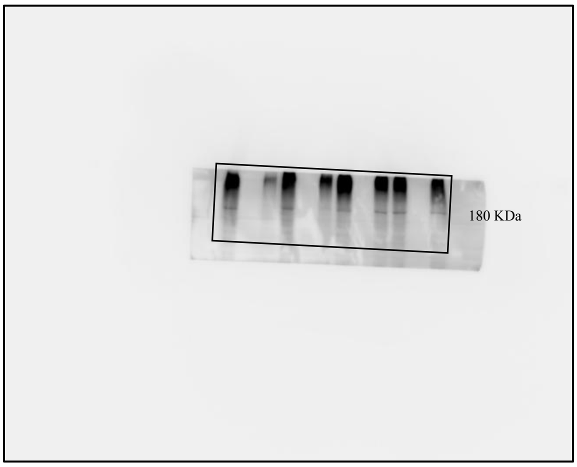

GAPDH-1

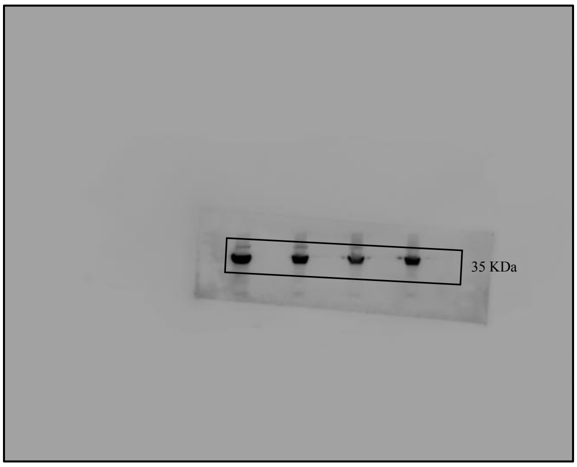

ATR-2

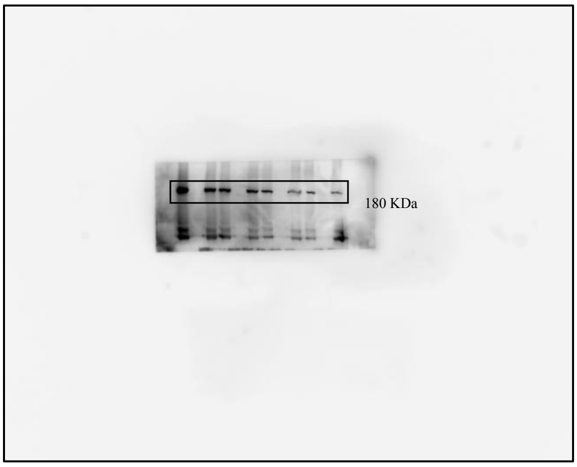

Ub-2

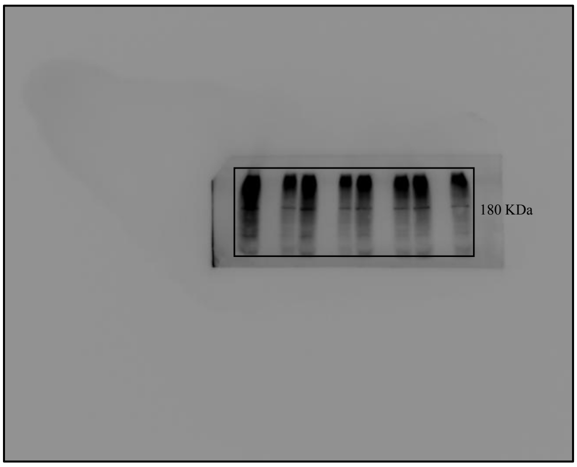

K63-Ub-2

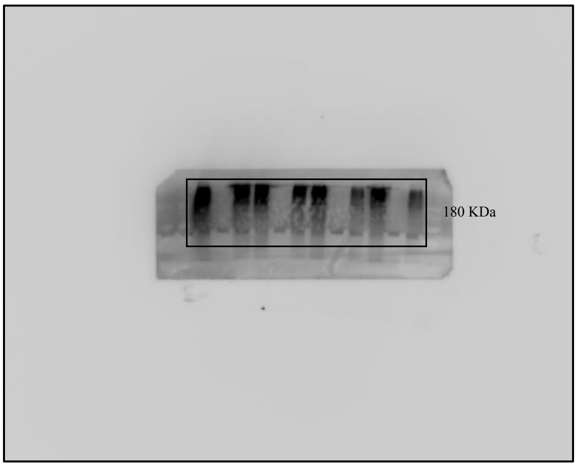

K48-Ub-2

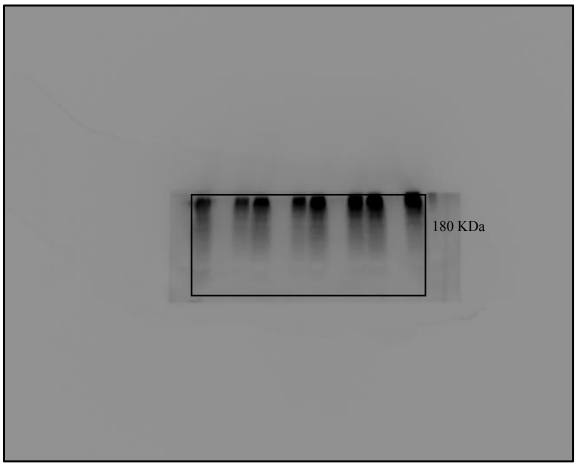

GAPDH-2

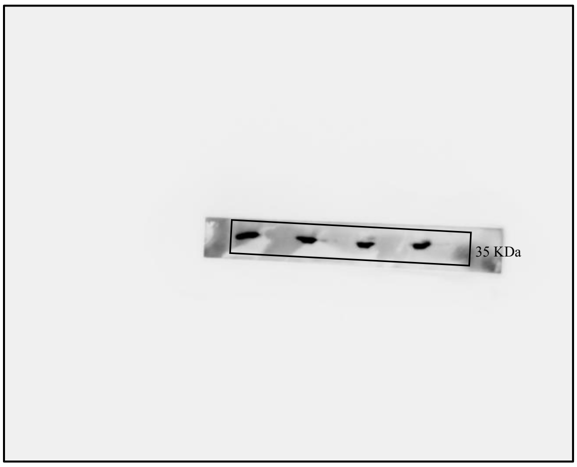

ATR-3

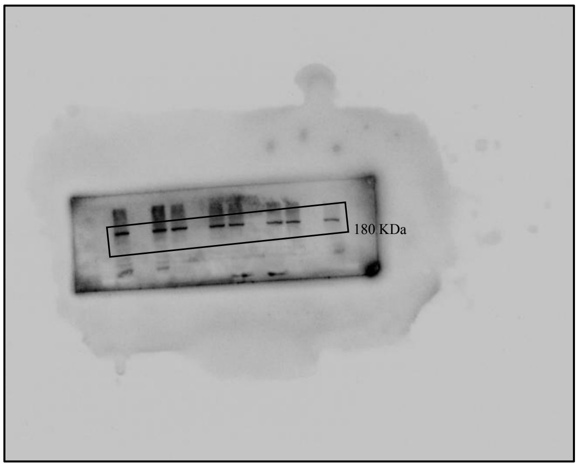

Ub-3

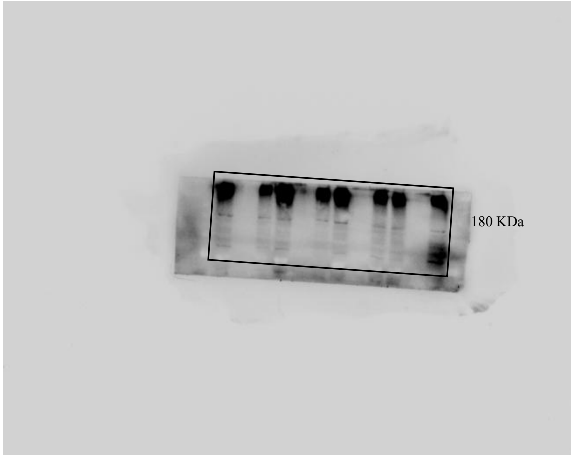

K63-Ub-3

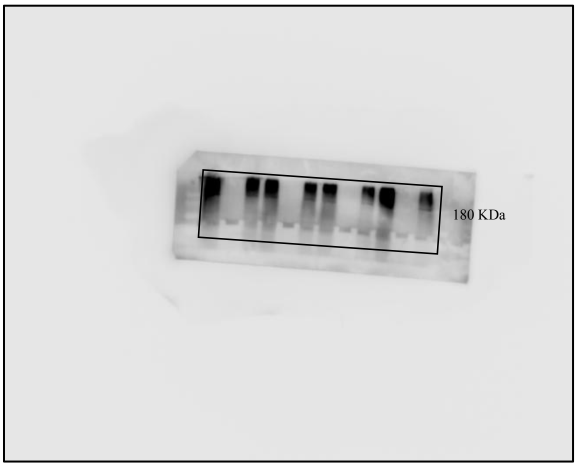

K48-Ub-3

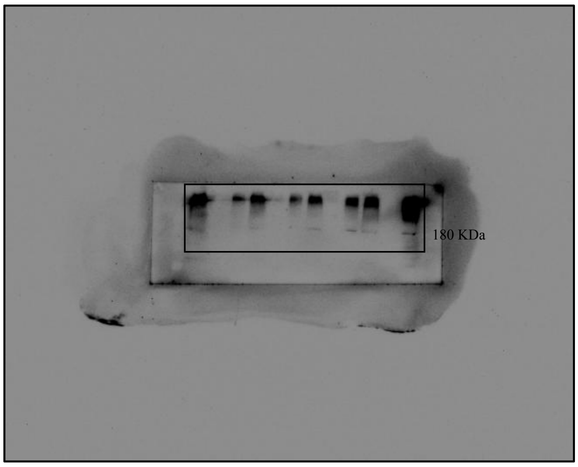

GAPDH-3

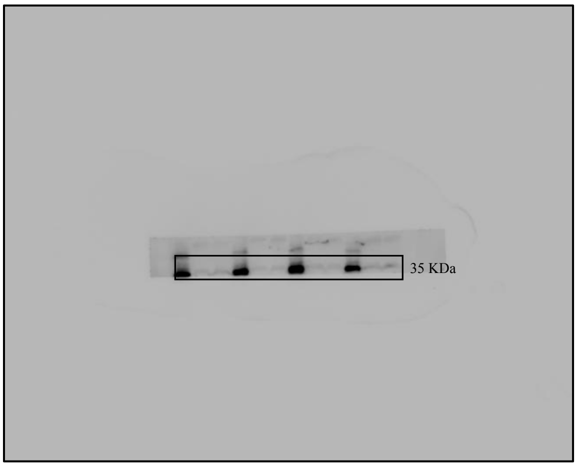

Figure 8D

IB: Flag

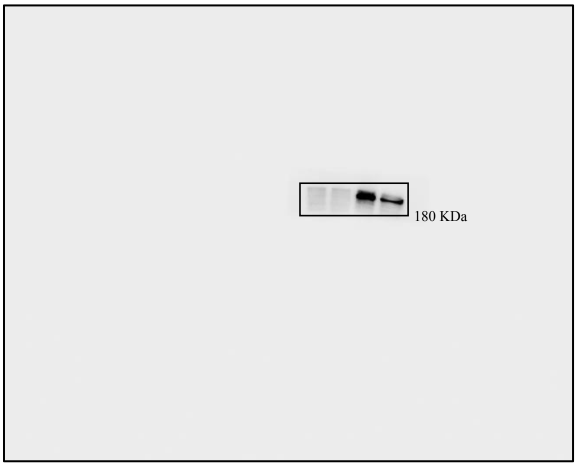

IB: Myc

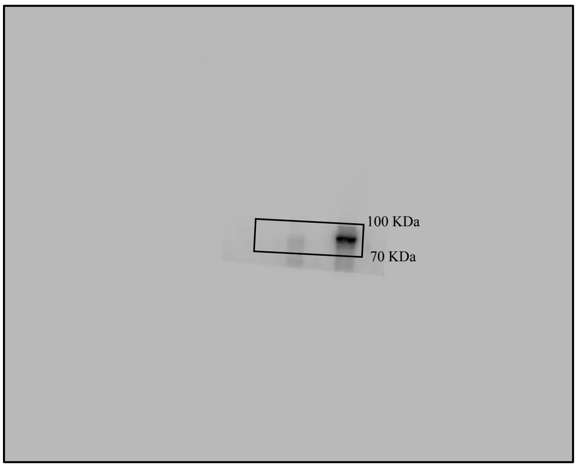

Input: Flag

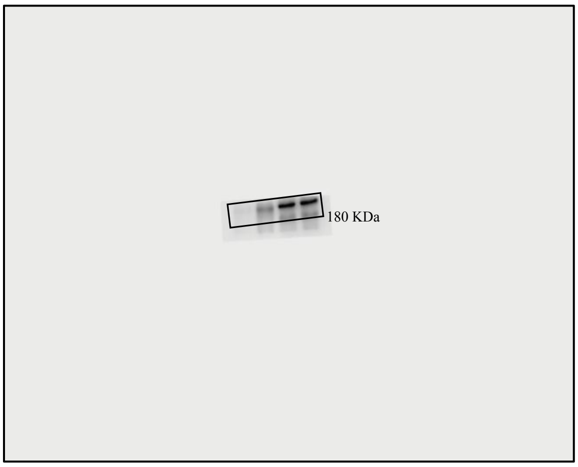

Input: Myc

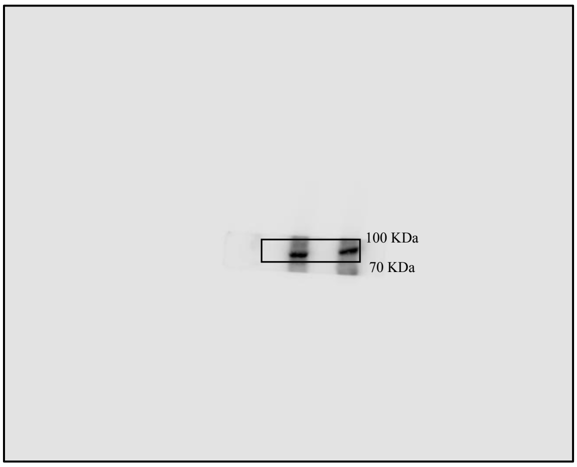

Input: GAPDH

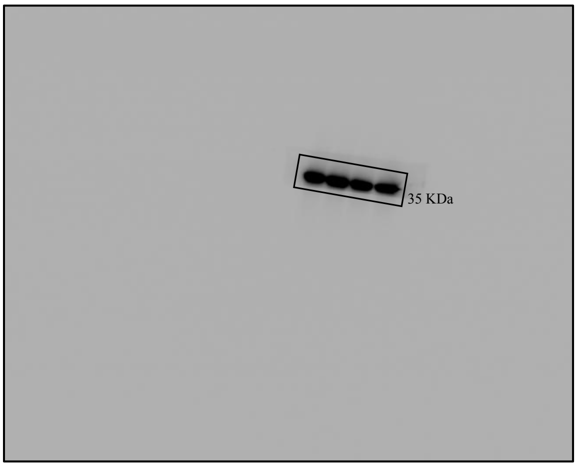

IB: Flag

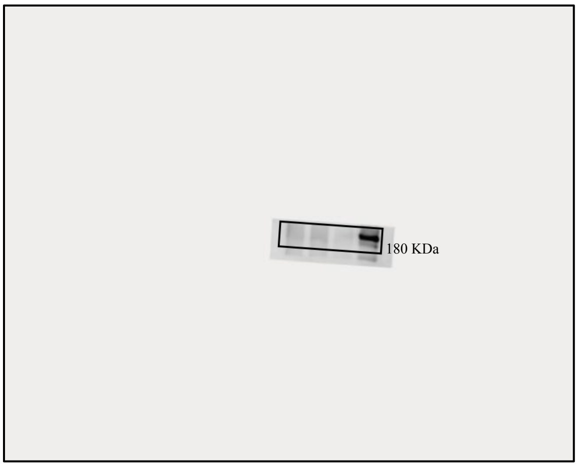

IB: Myc

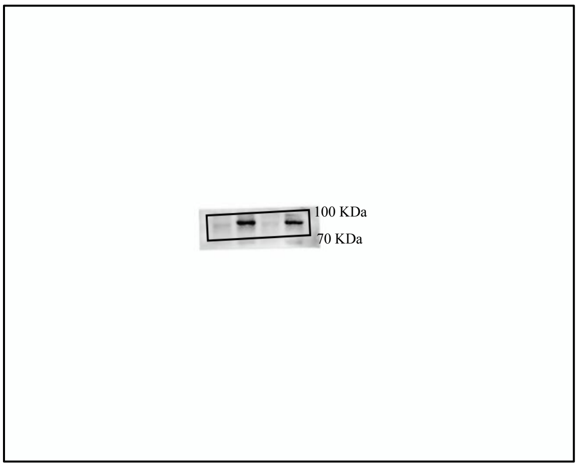

Input: Flag

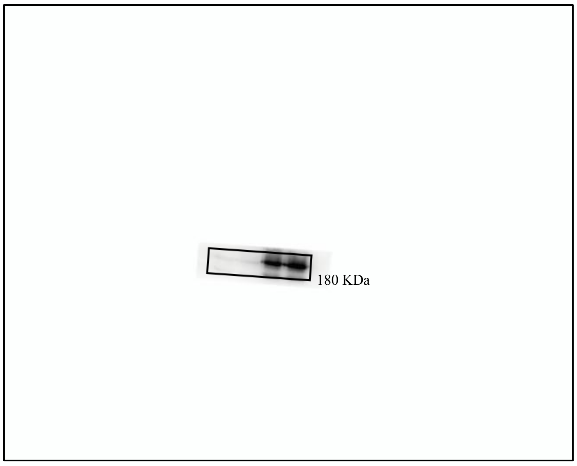

Input: Myc

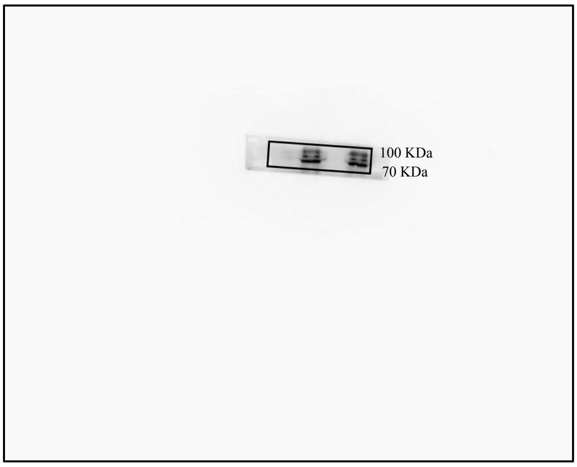

Input: GAPDH

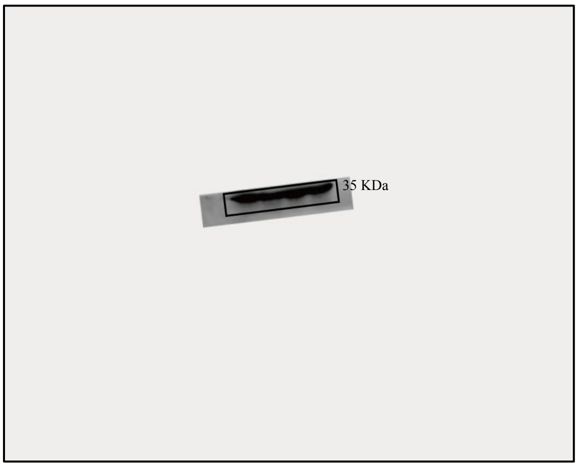
35 KDa

Figure 8E

ATR

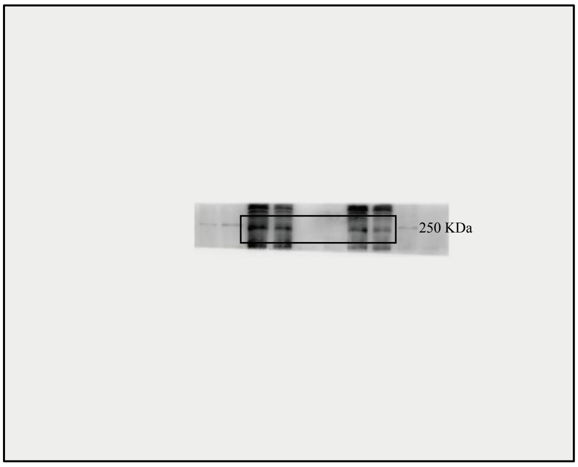

TRIM56

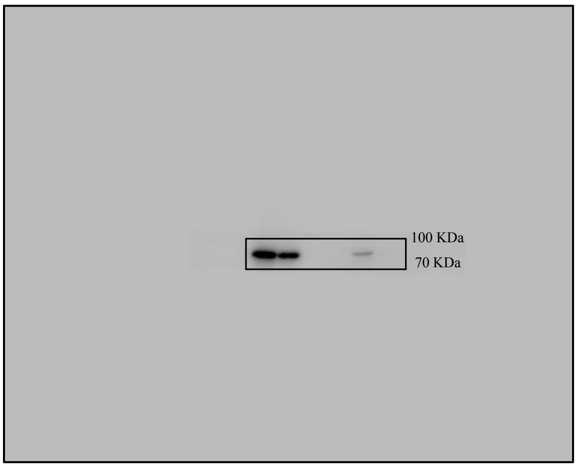

GAPDH

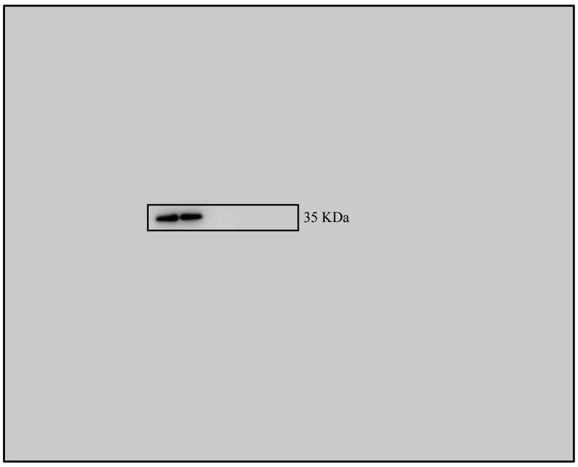

IB: ATR

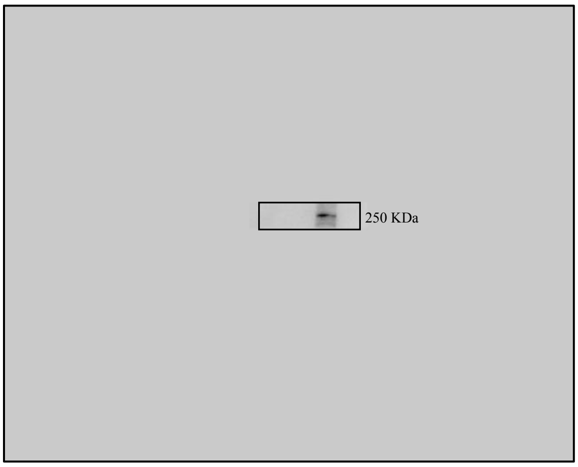

IB: TRIM56

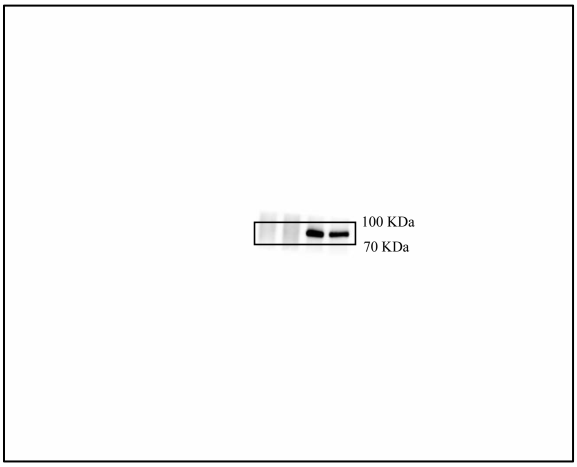

Input: ATR

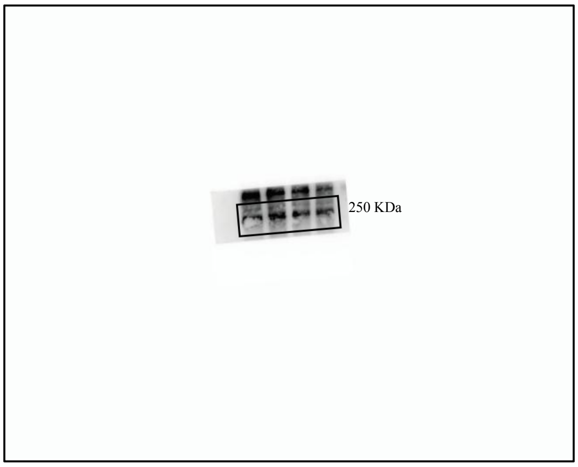

Input: TRIM56

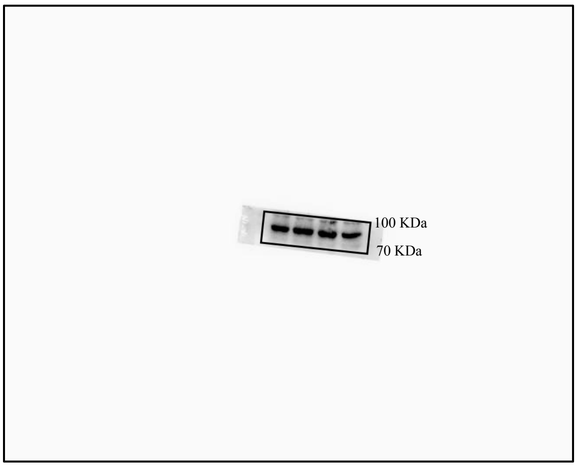

Input: GAPDH

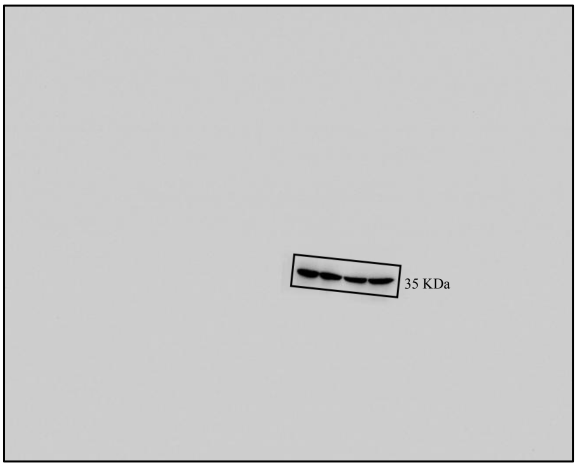

Figure 8I

Input: Flag

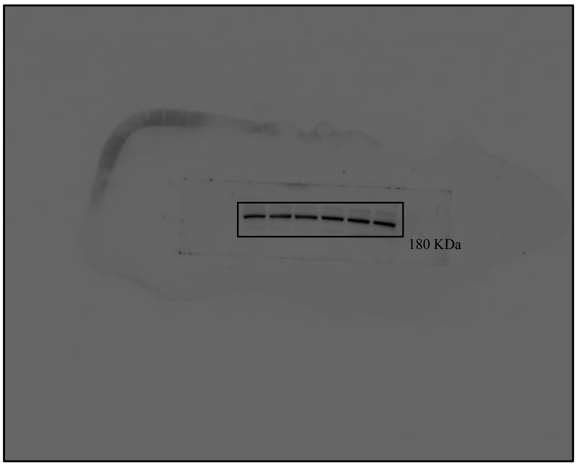

Input: TRIM56

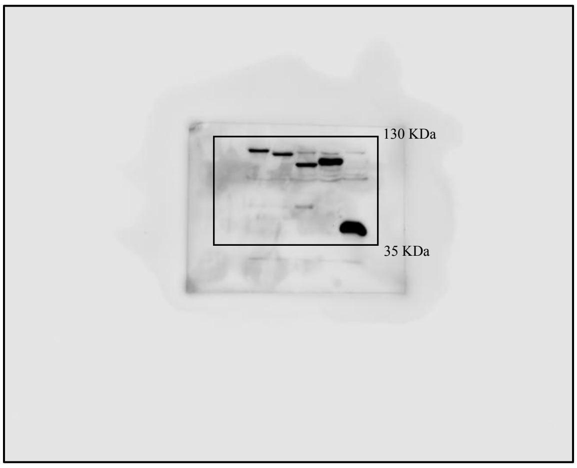

Input: GAPDH

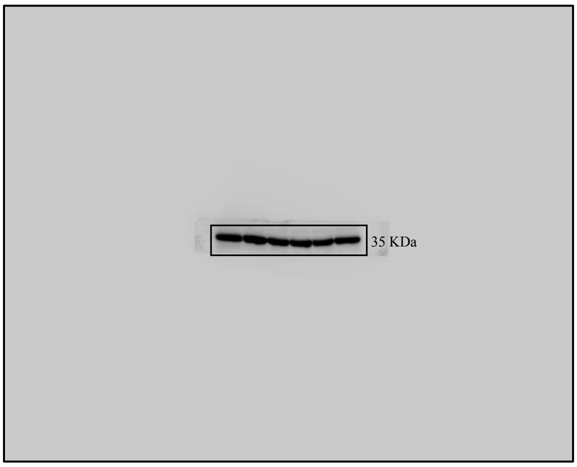

IB: Flag

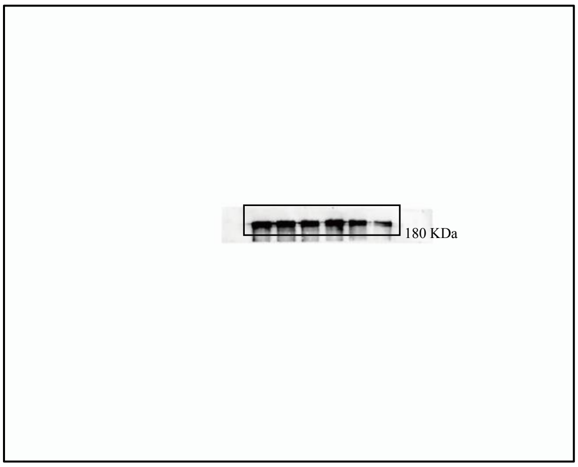

IB: TRIM56

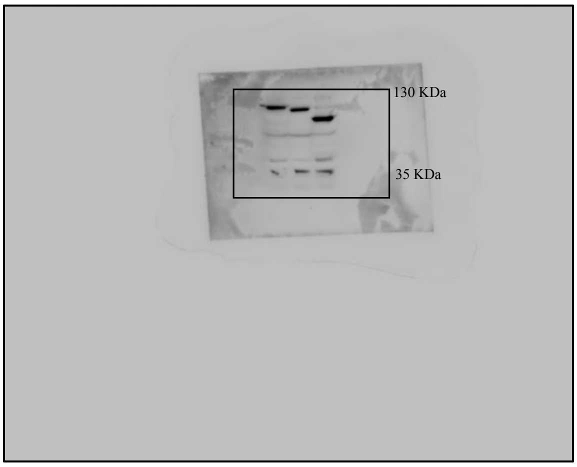

Figure 8J

Input: Myc

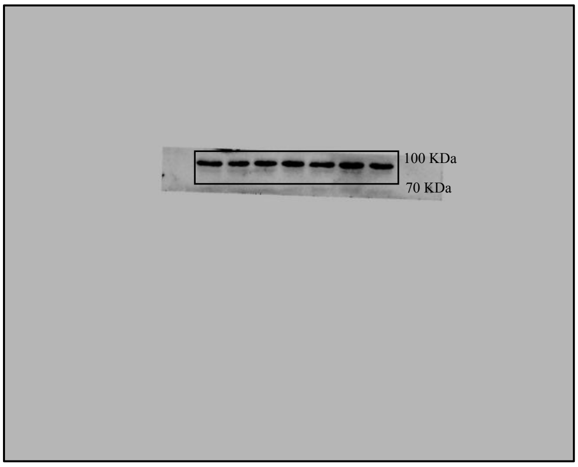

Input: Flag

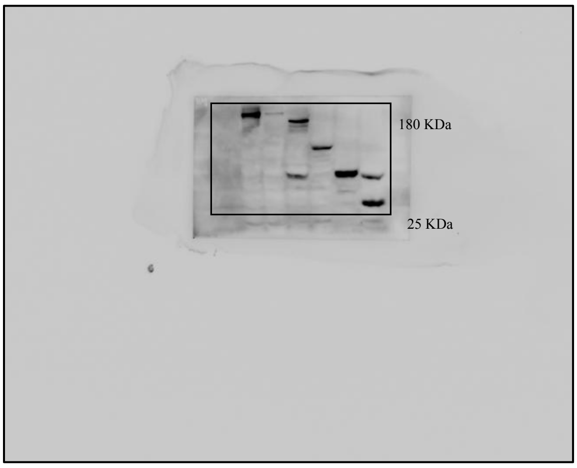

Input: GAPDH

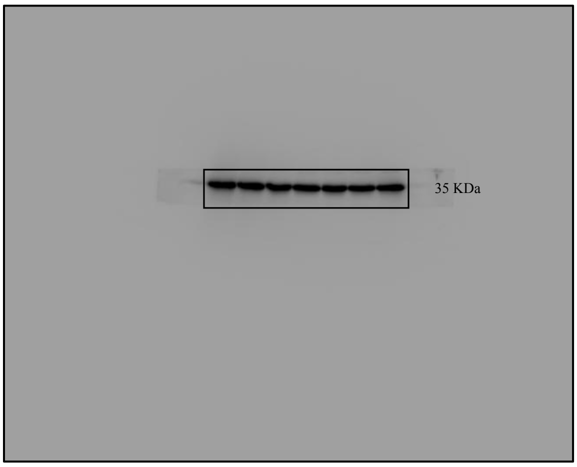

IB: Myc

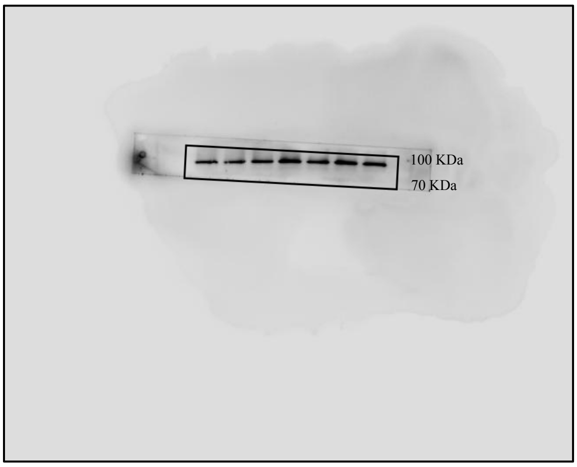

IB: Flag

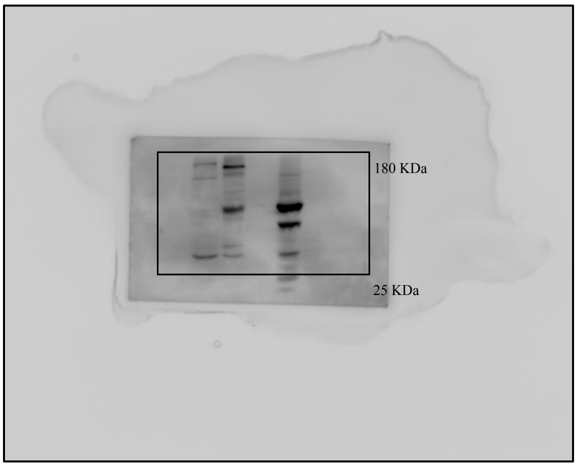

Figure 9A

IB: HA

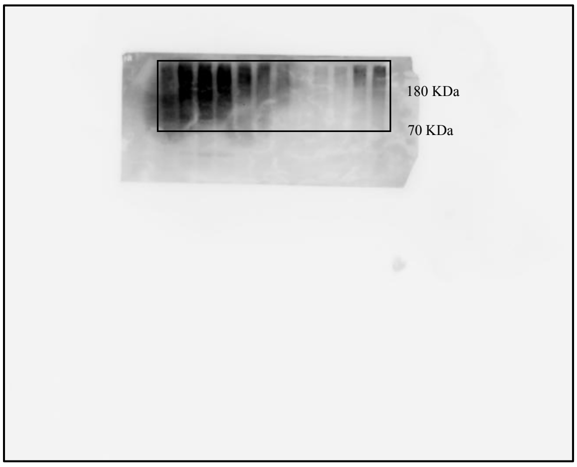

IB: Flag

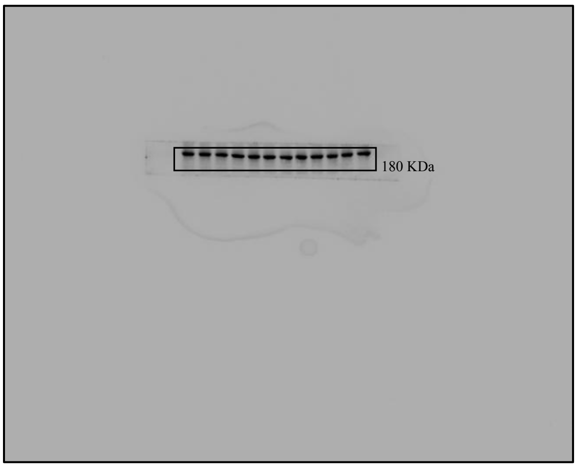

Input: Flag

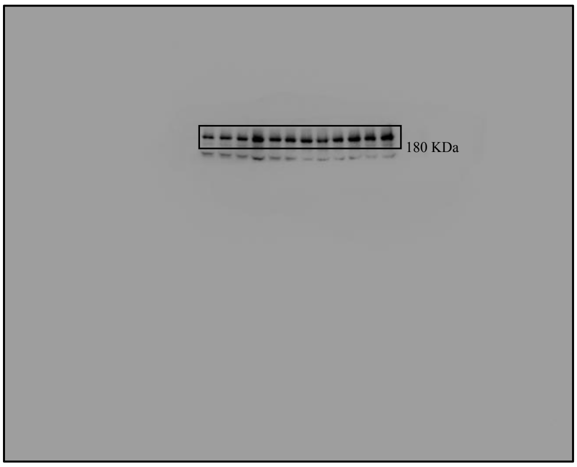

Input: Myc

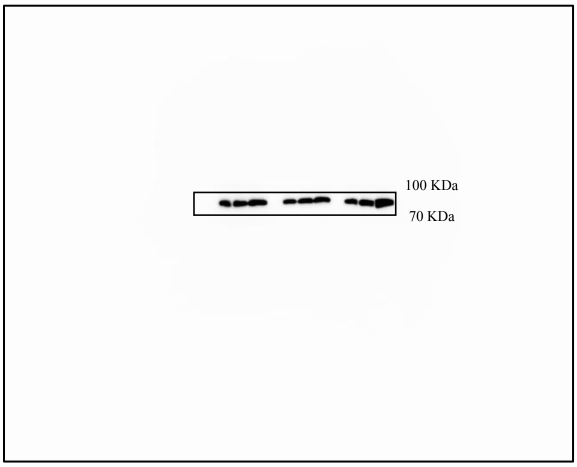

Input: HA

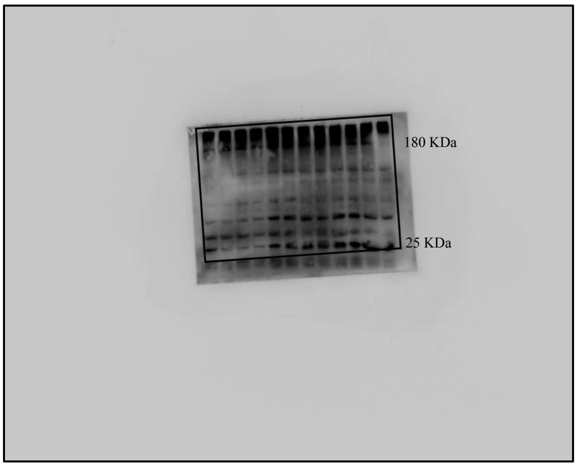

GAPDH

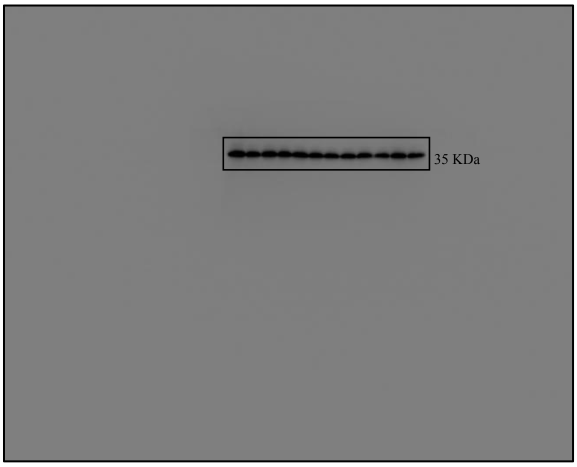

Figure 9B

IB: Flag

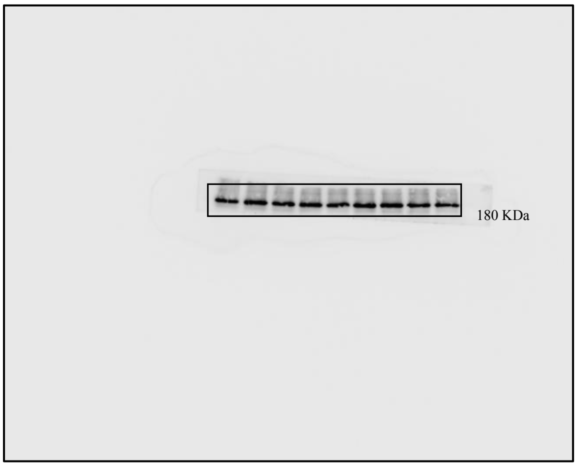

IB: HA

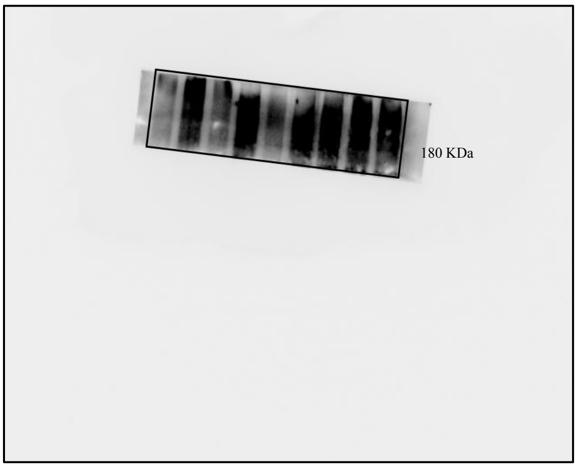

IB: Myc

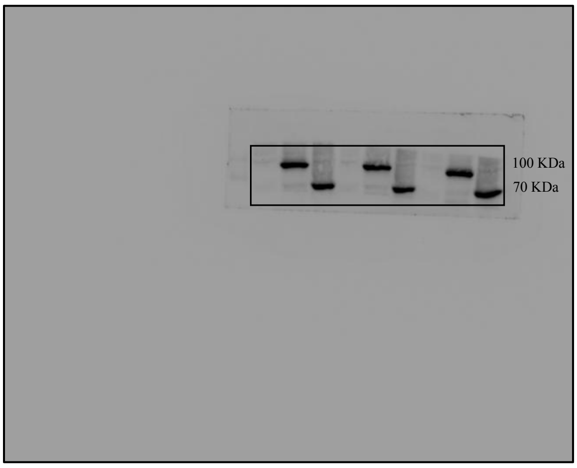

Input: Flag

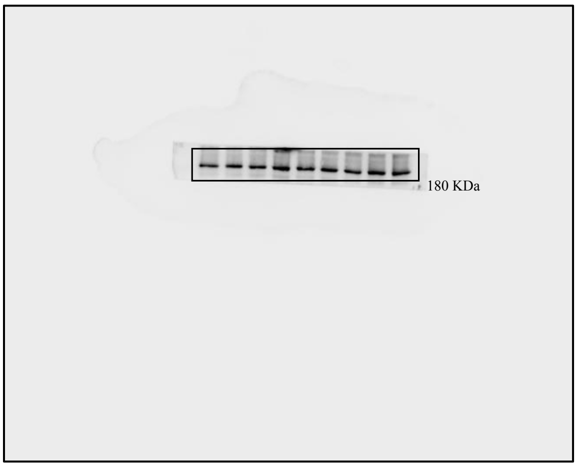

Input: Myc

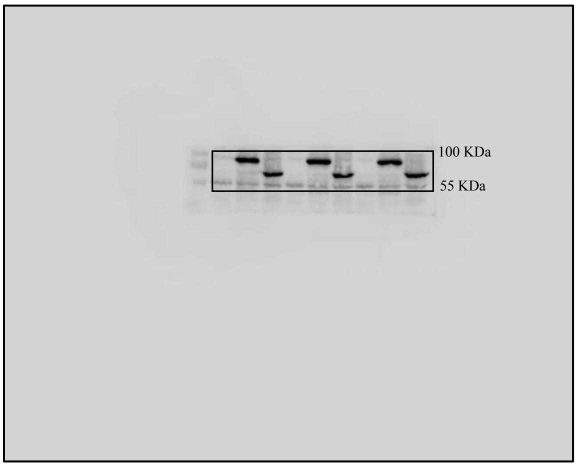

Input: HA

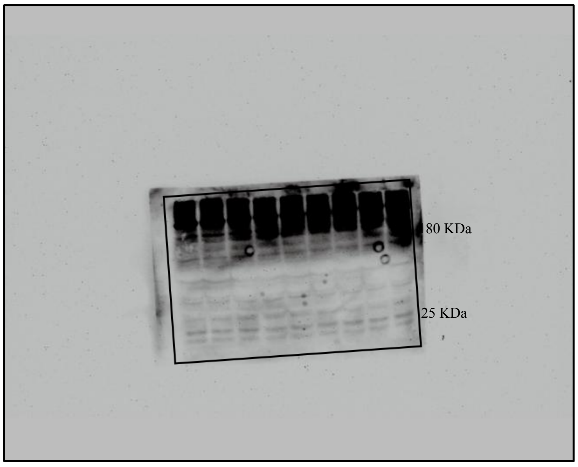

Input: GAPDH

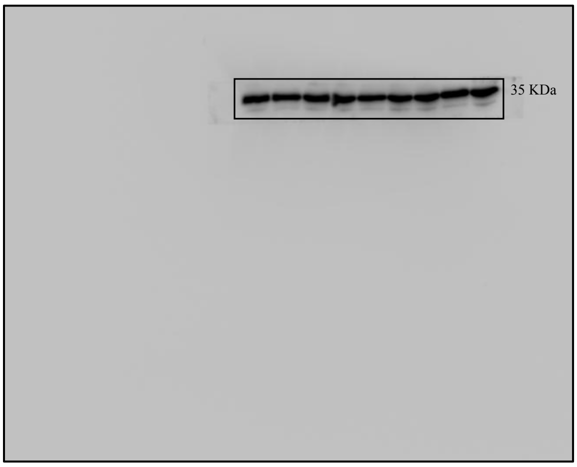
35 KDa

Figure 9C

IB: HA

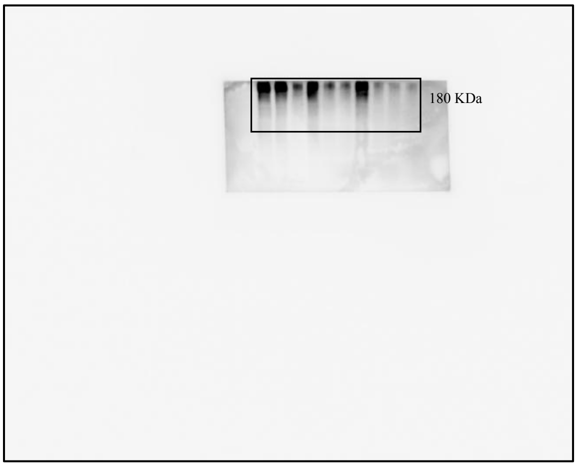

IB: Flag

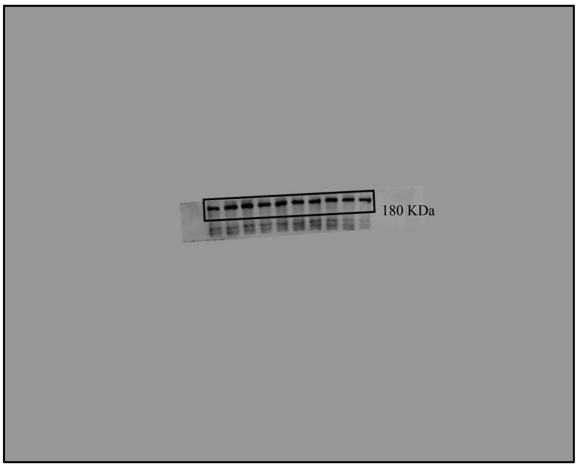

Input: Flag

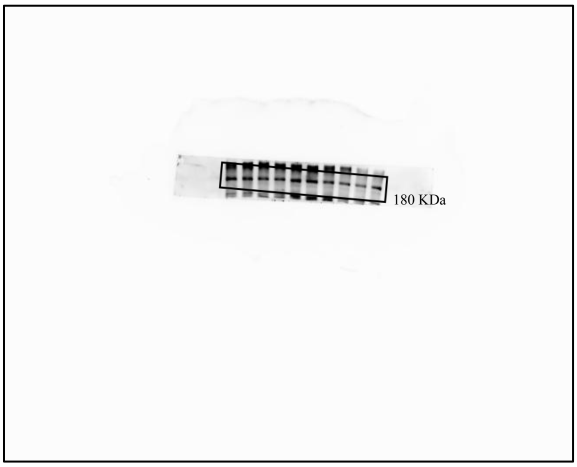

Input: Myc

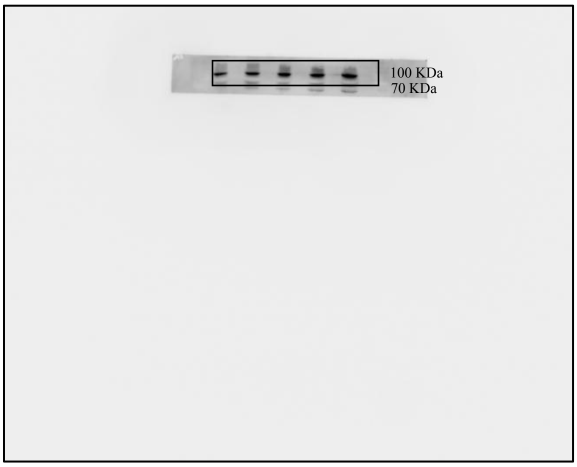

Input: GAPDH

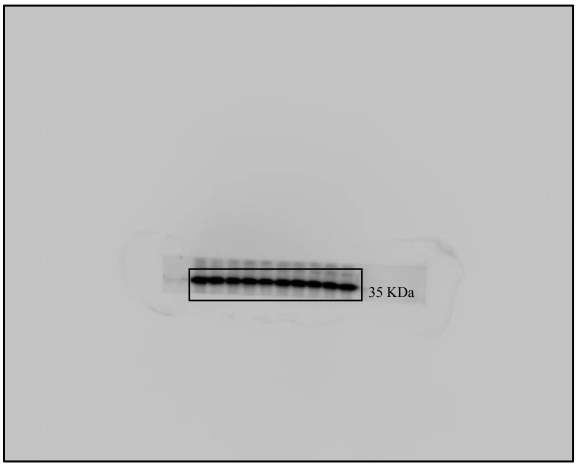

Figure 9F

IB: HA

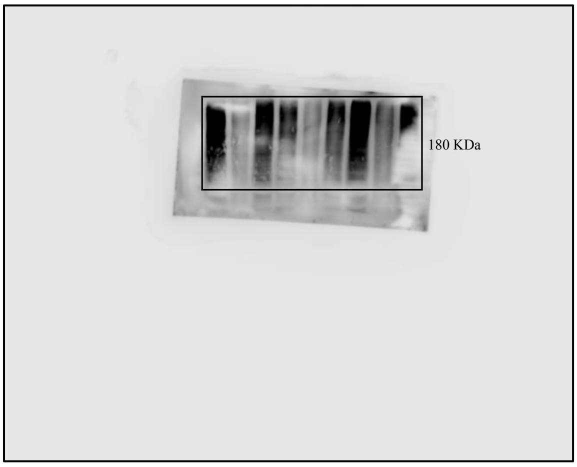

IB: Flag

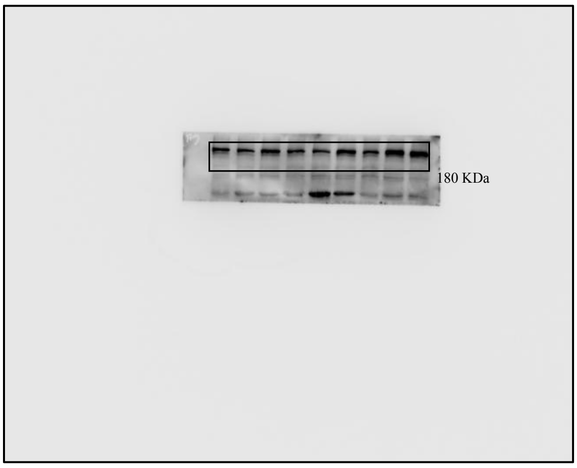

IB: Myc

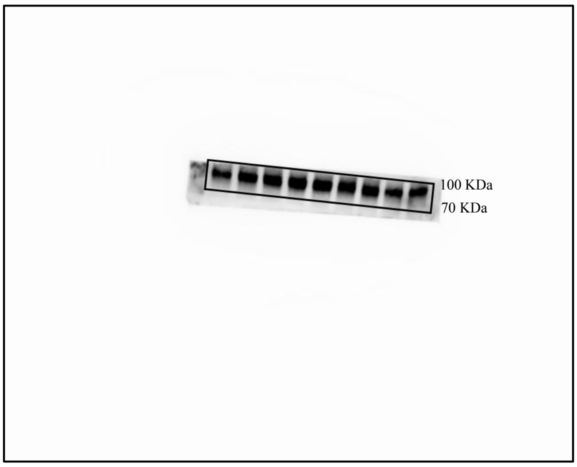

Input: Flag

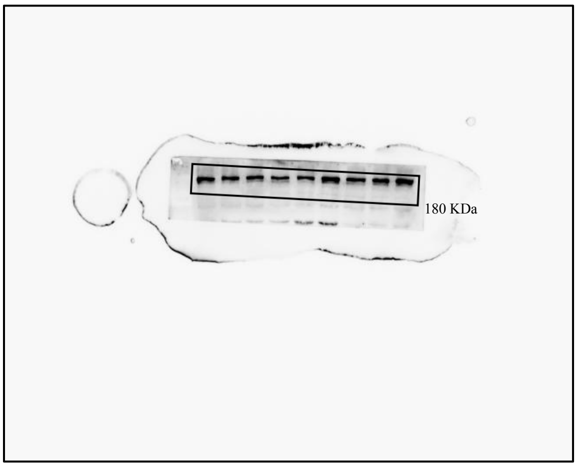

Input: HA

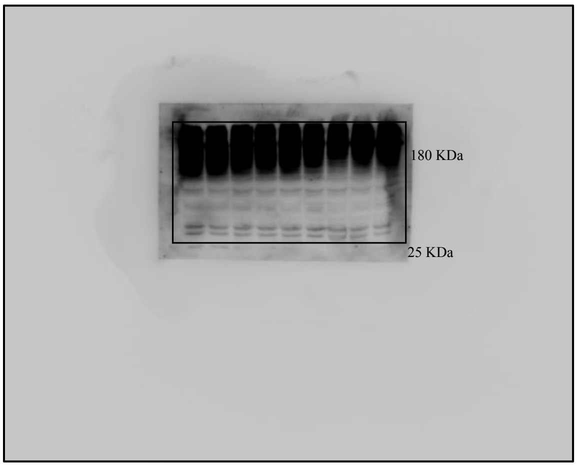

Input: Myc

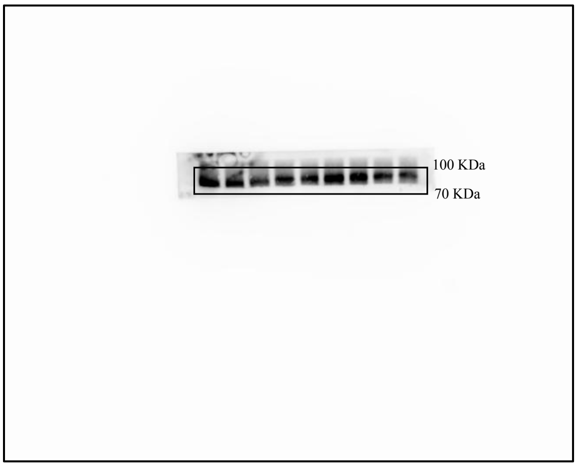

Input: GAPDH

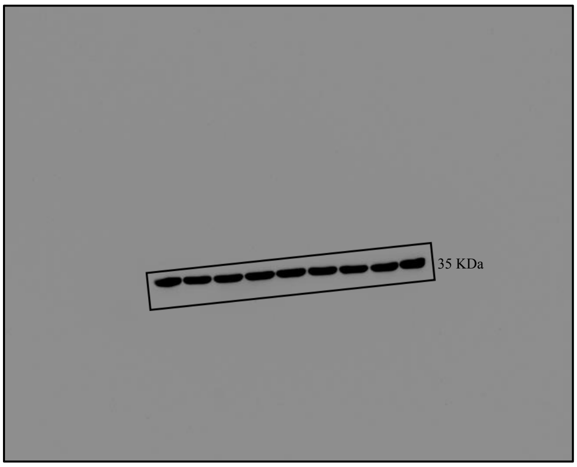
35 KDa

Figure 9G

IB: HA

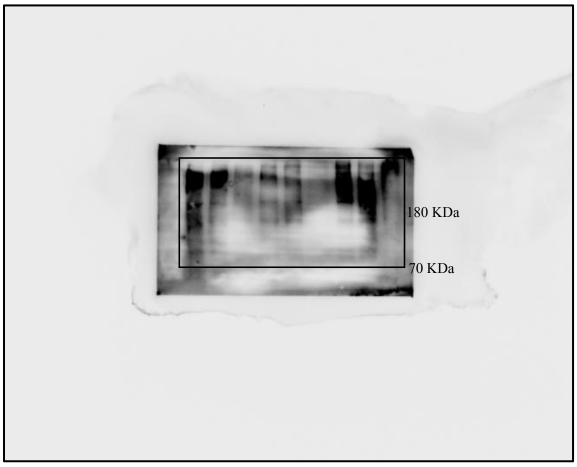

IB: Flag

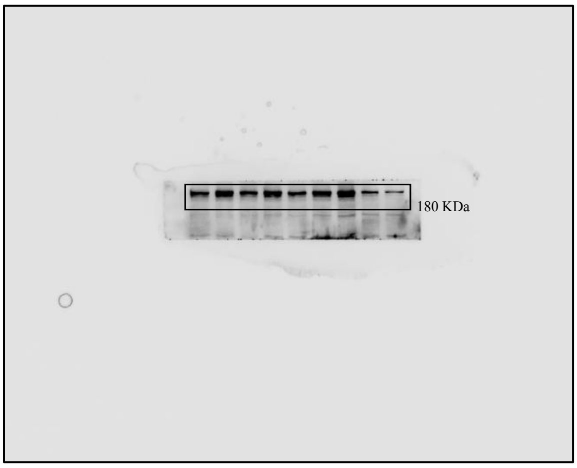

IB: TRIM56

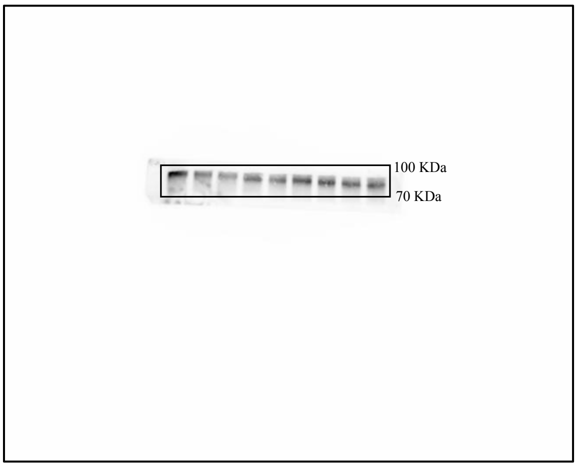

Input: Flag

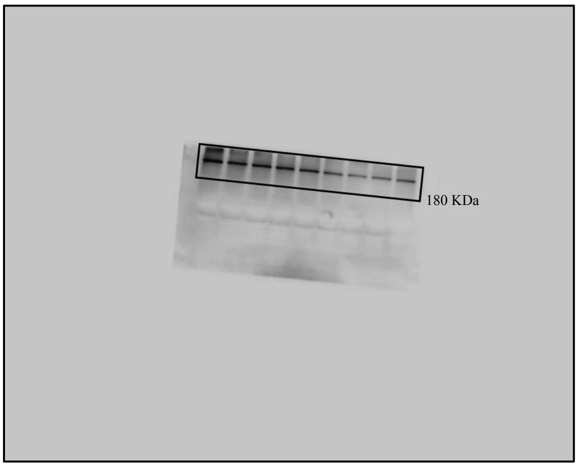

Input: HA

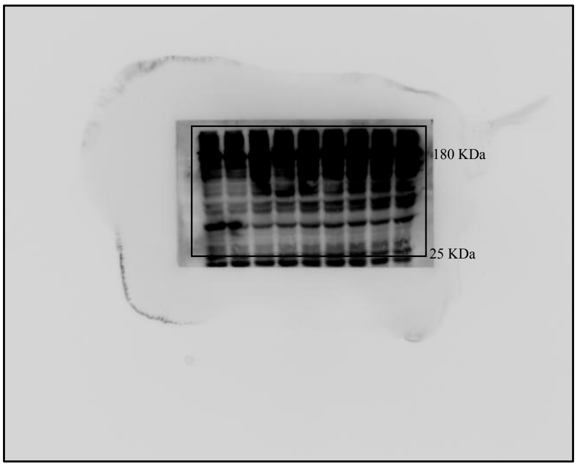

Input: Myc

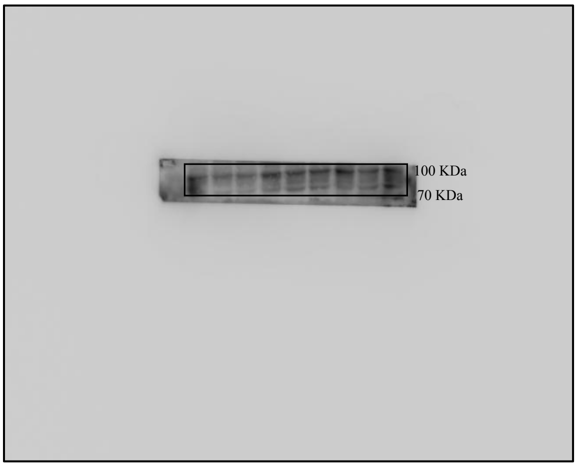

Input: GAPDH

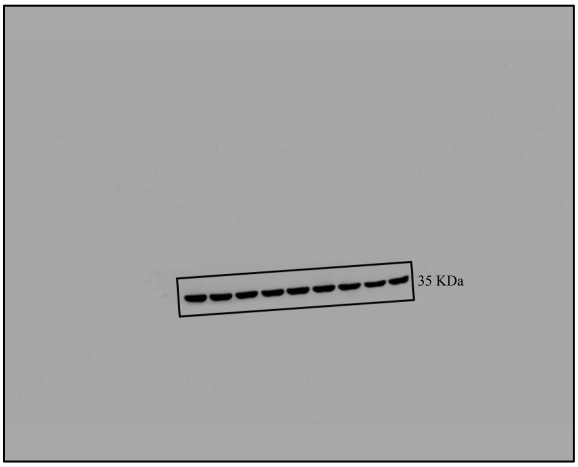
35 KDa

Figure 10A

p-p53

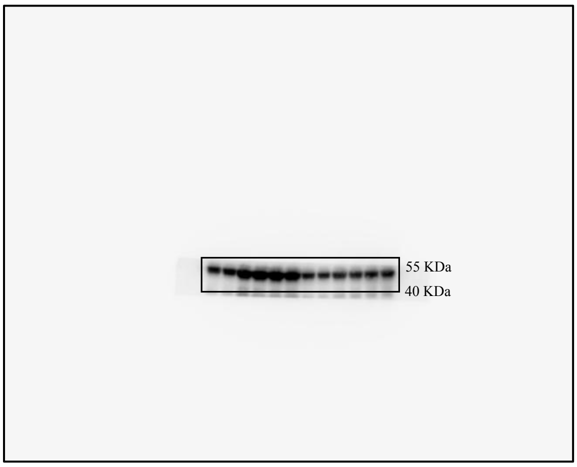

p21

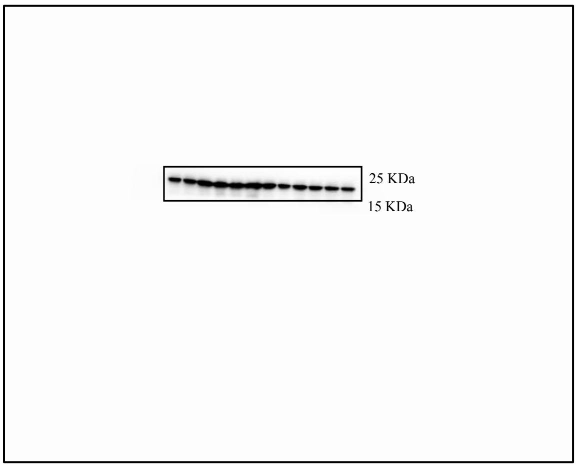

p16

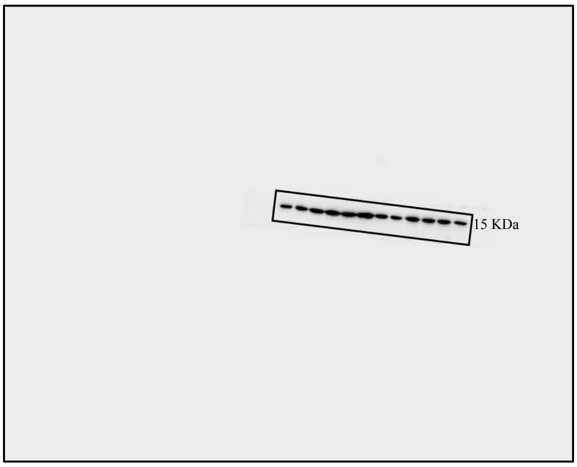

GAPDH

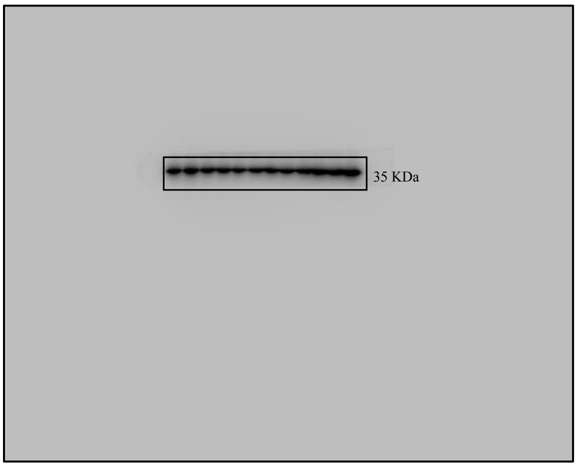
35 KDa

Figure 10D

cGAS

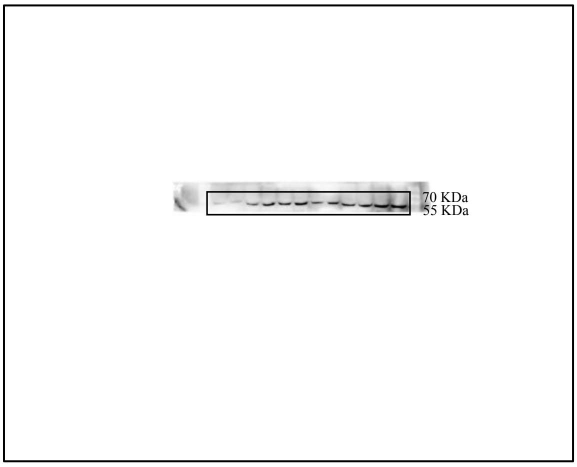

p-STING

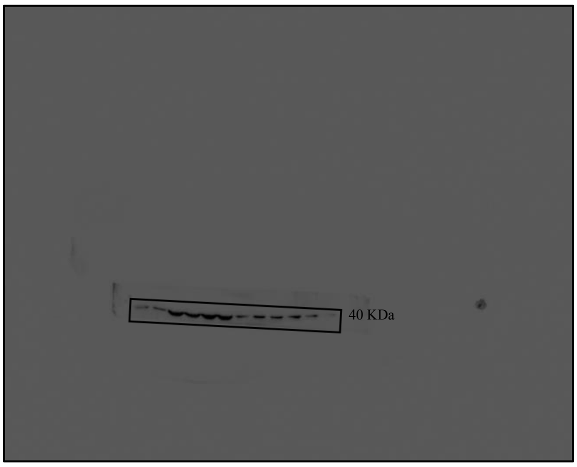

STING

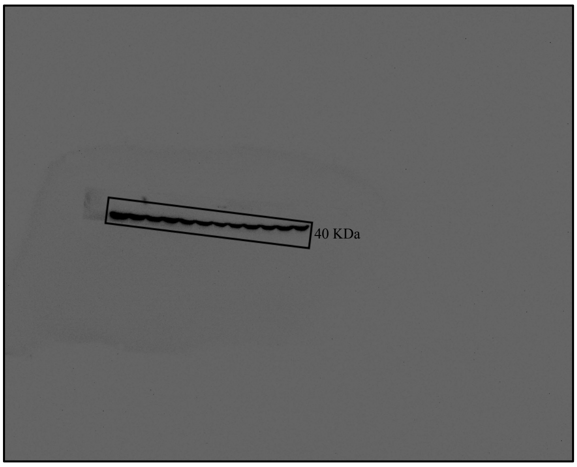

GAPDH

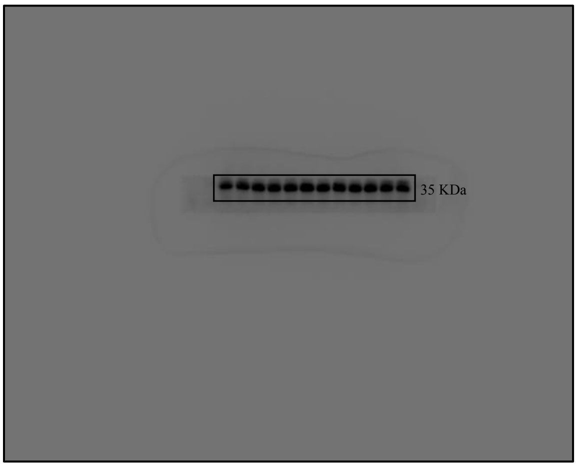

γH2A

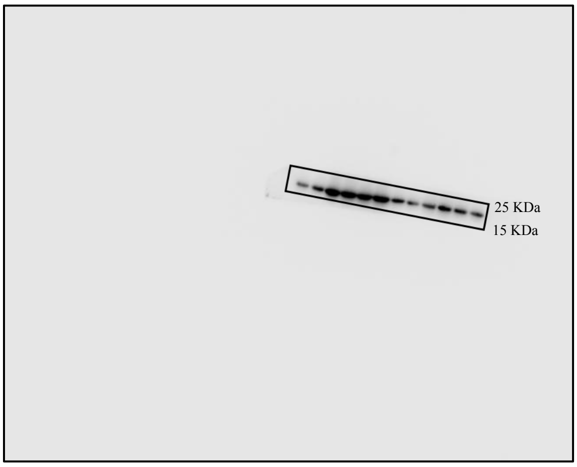

GAPDH

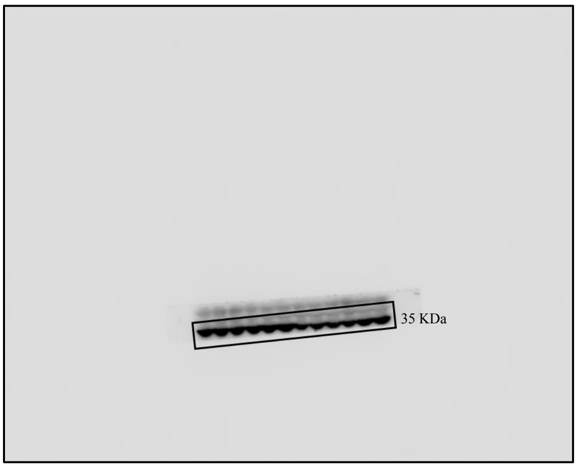

Figure 10F

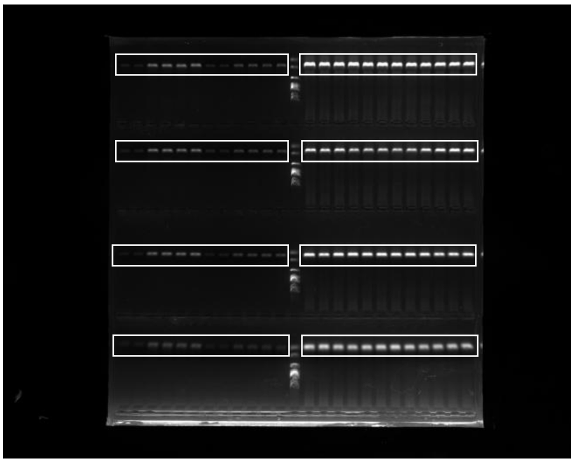

Figure 10G

gDNA

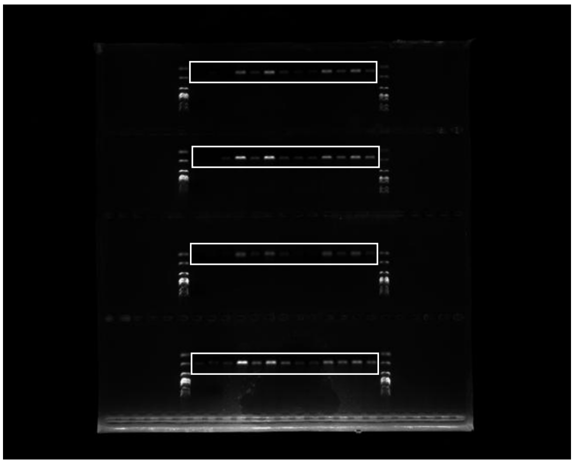

IB: cGAS

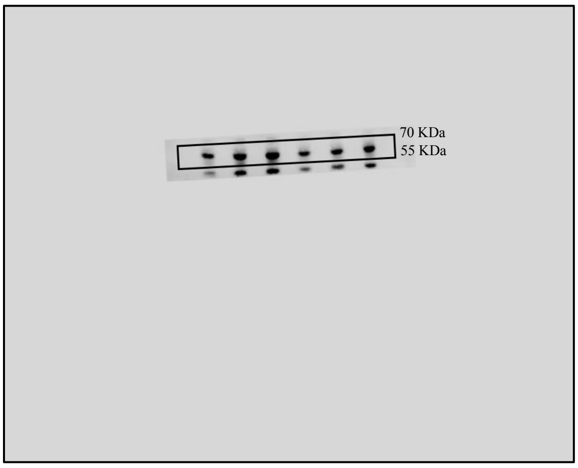

IB: AIM2

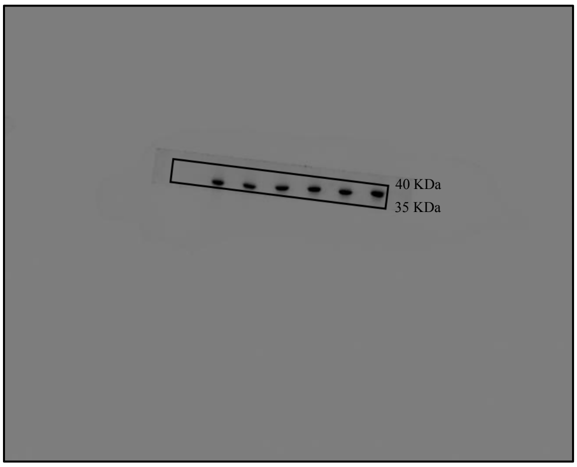

Input: gDNA

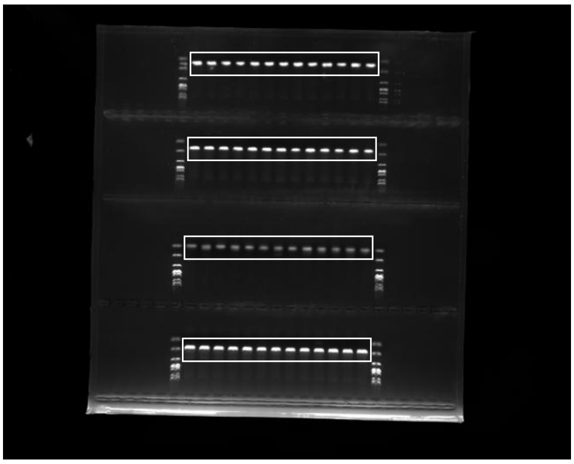

Input: cGAS

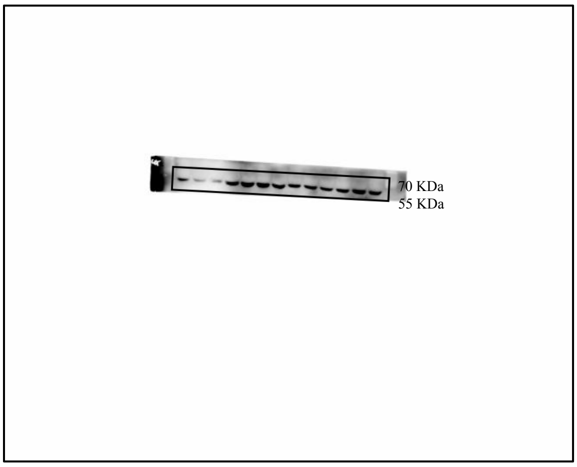

Input: AIM2

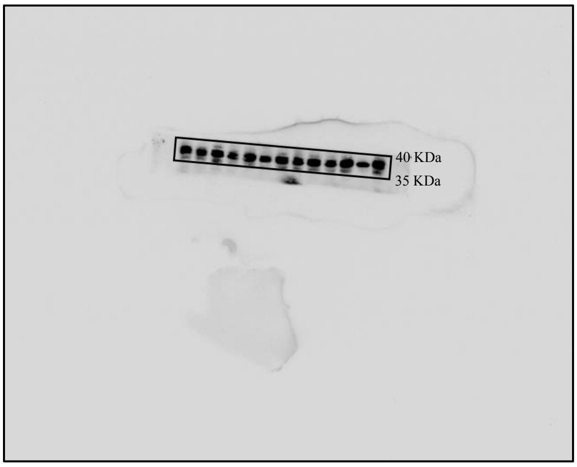

Input: GAPDH

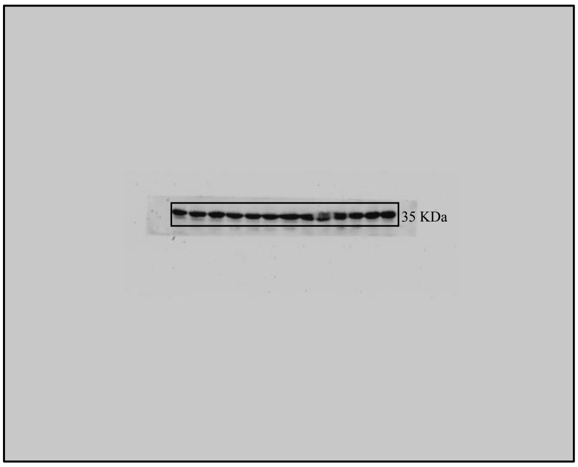
35 KDa

Figure 12A

USP10

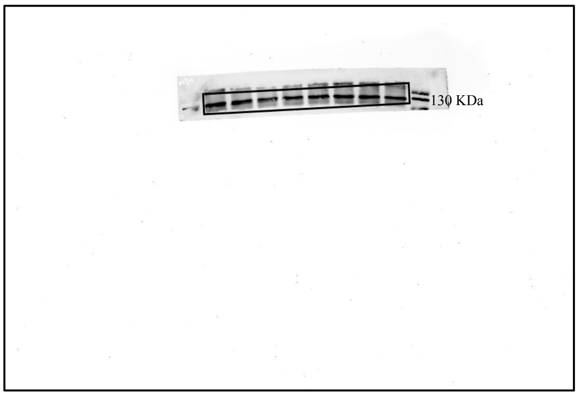

USP5

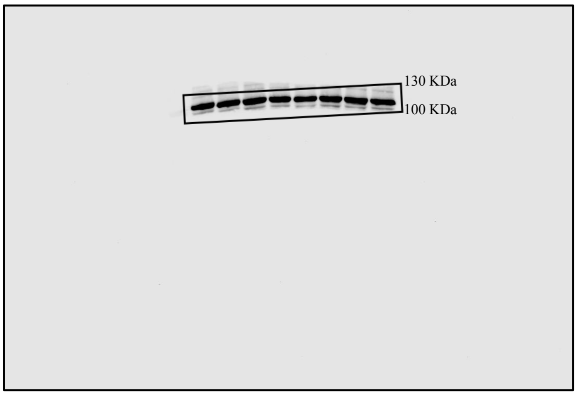

TIRM56

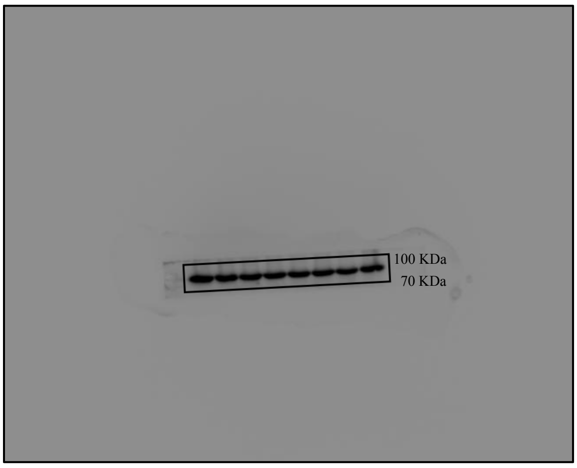

TRIM25

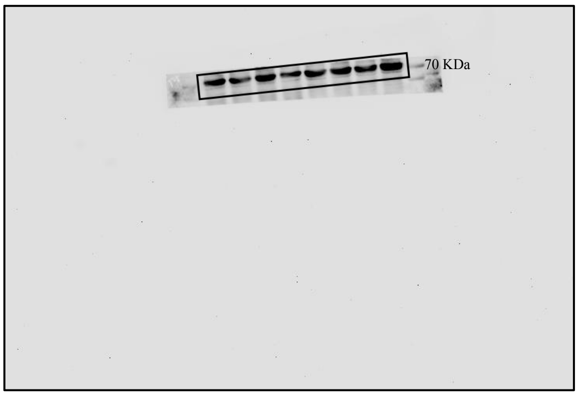

MKRN1

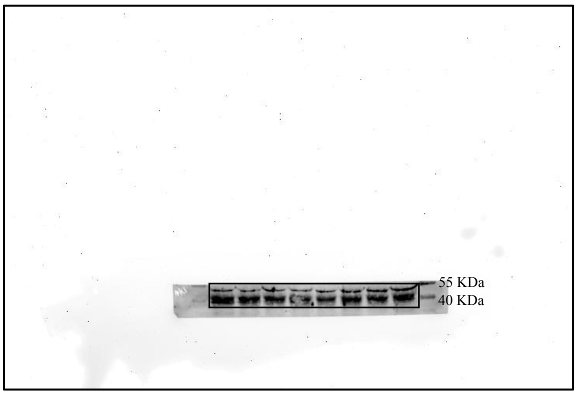

GAPDH

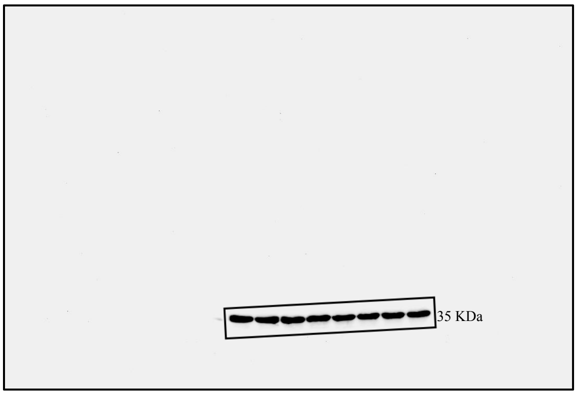

Figure 12F

USP10

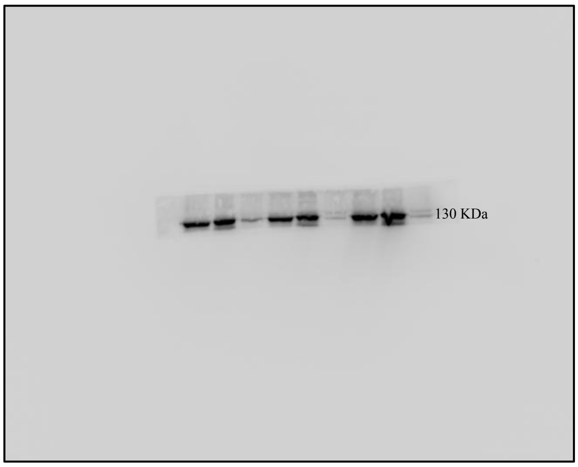

USP5

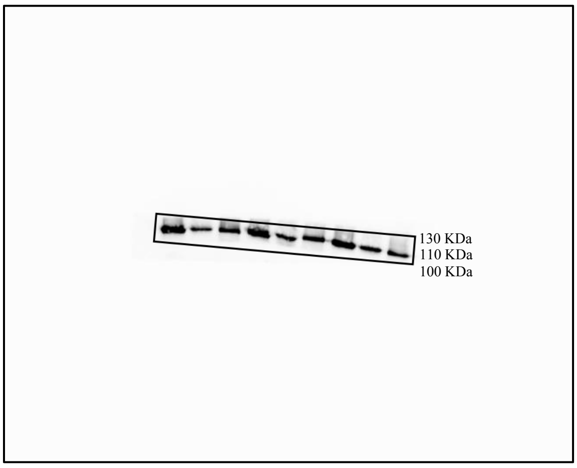

TRIM56

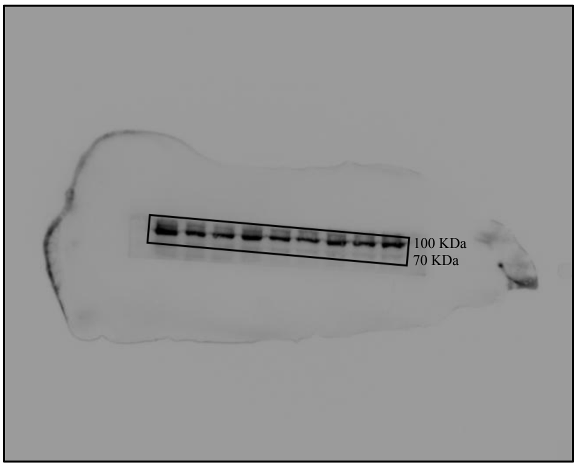

TRIM25

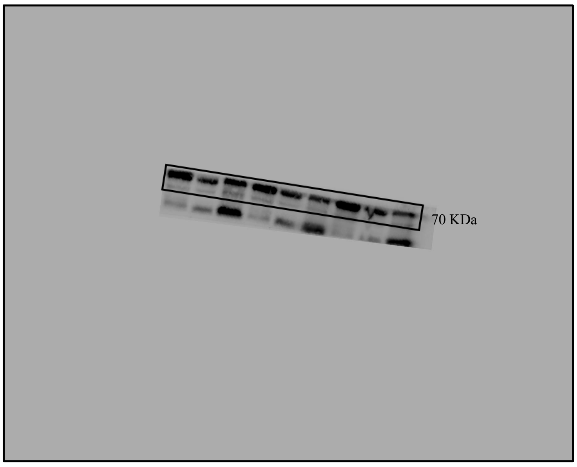

MKRN1

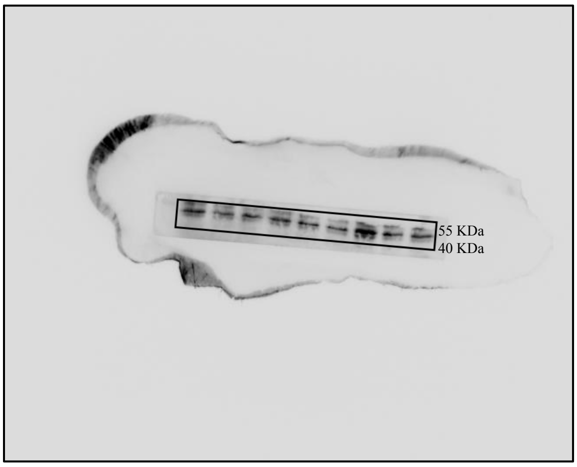

Tubulin

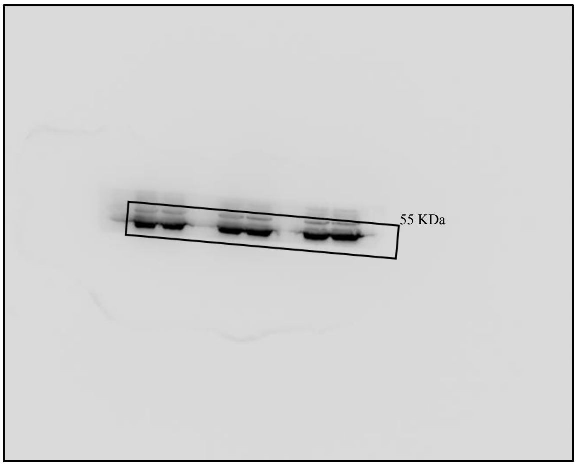

PCNA

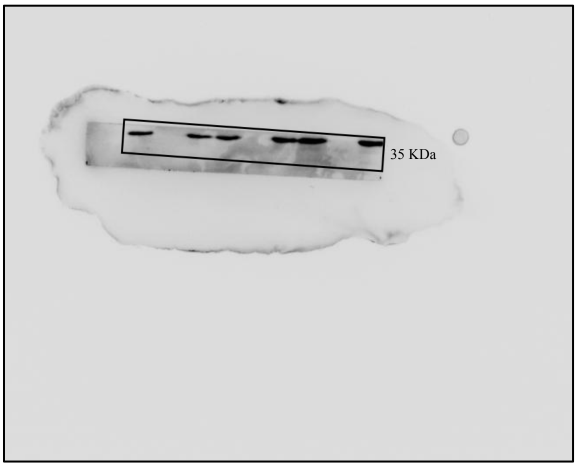

USP10

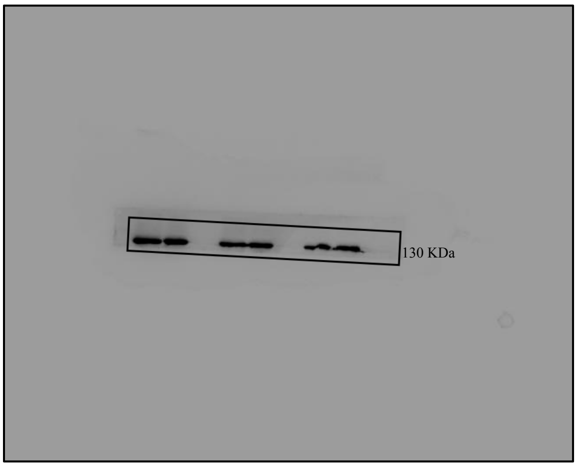

USP5

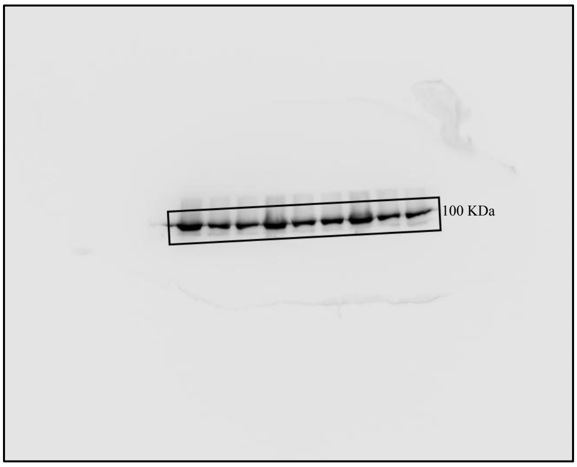

TRIM56

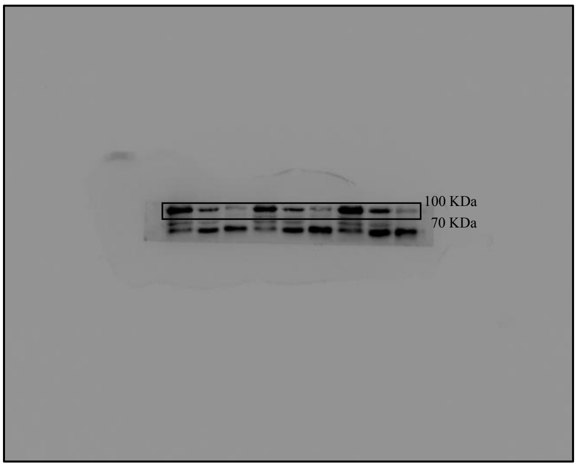

TRIM25

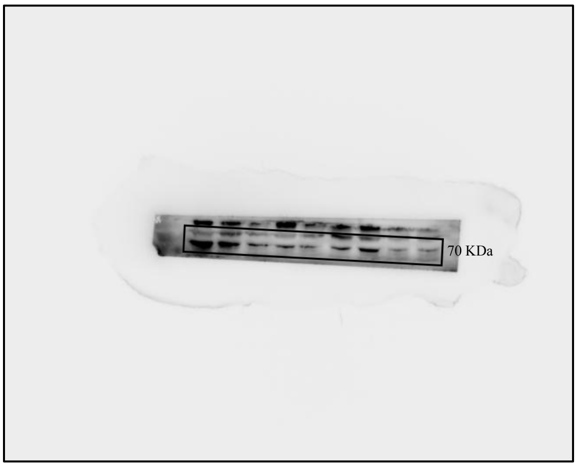

MKRN1

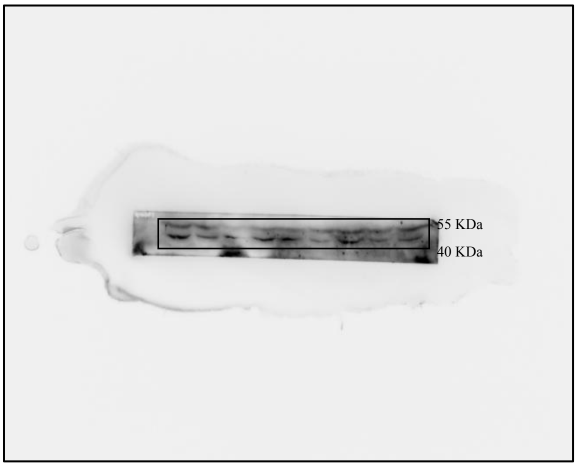

Tubulin

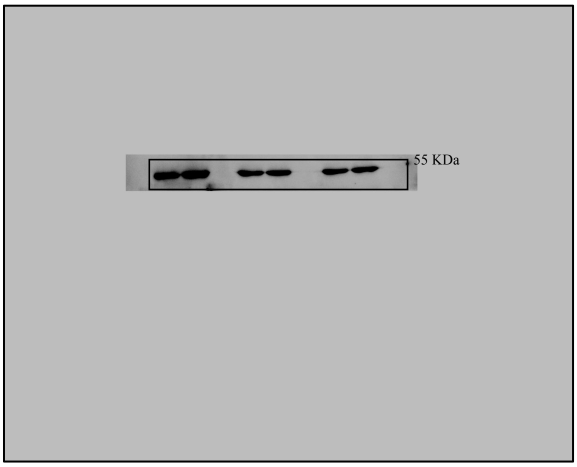

PCNA

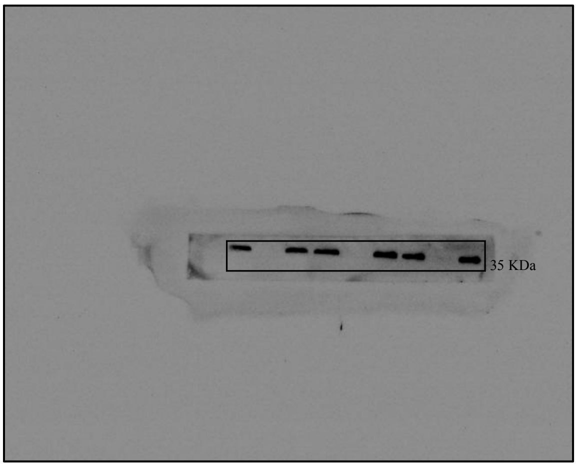

Figure 12I

Myc

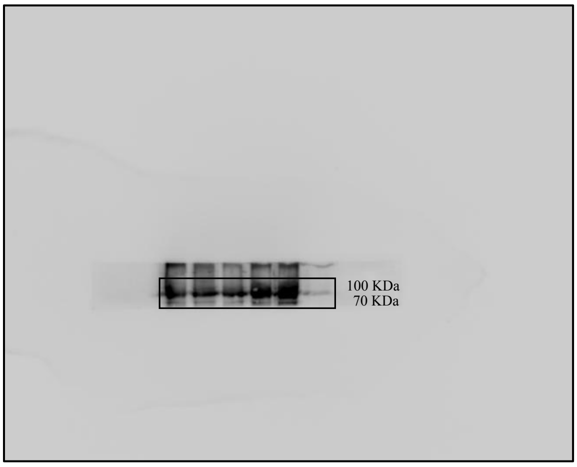

Tubulin-PCNA

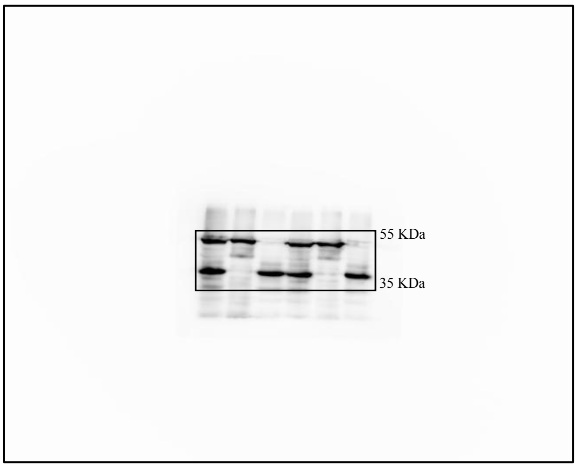

Figure 13A

ATR

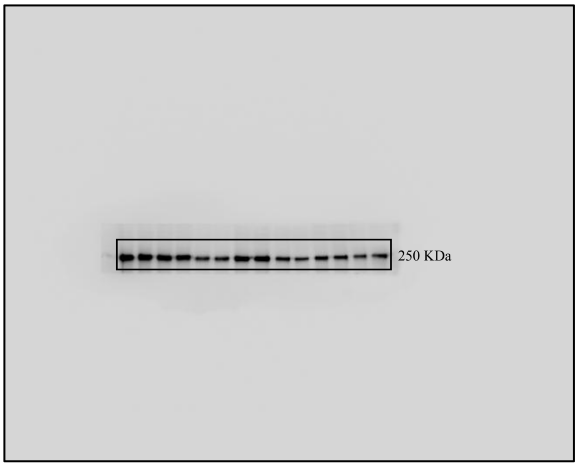

GAPDH

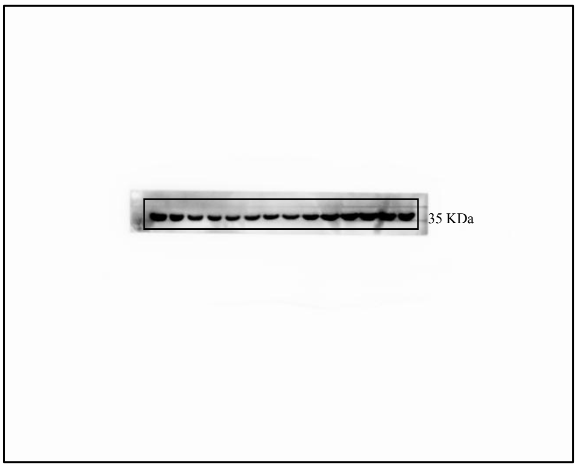

Figure 13B

ATR

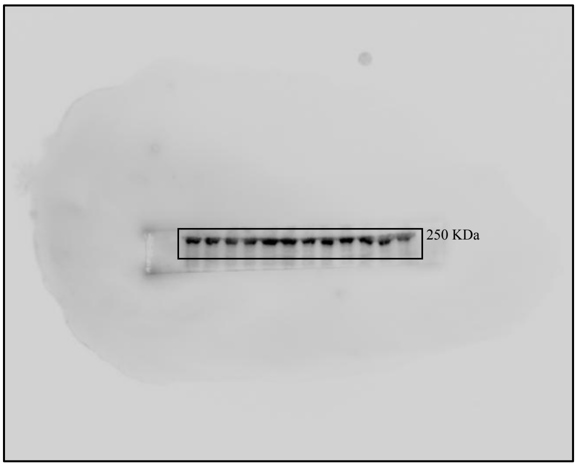

GAPDH

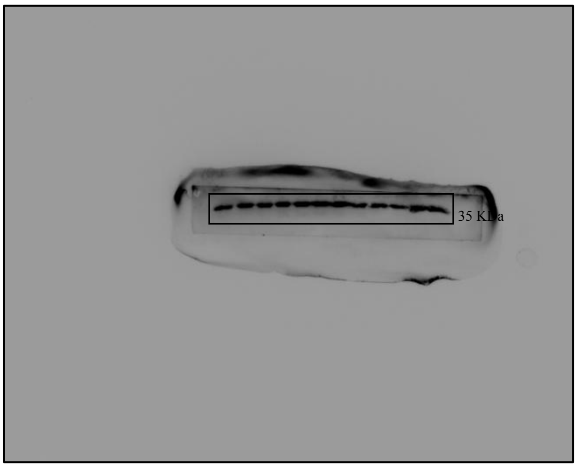

Figure 13C

ATR

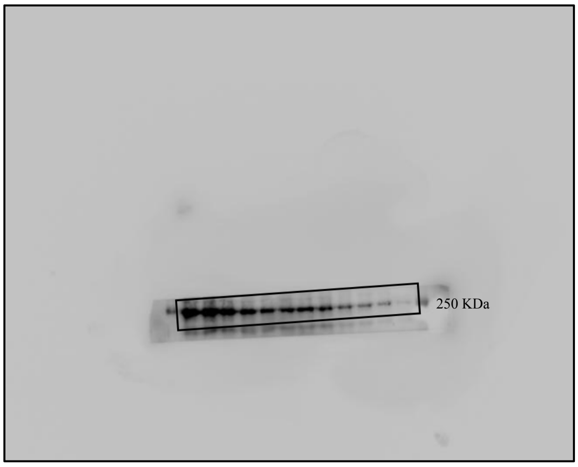

GAPDH

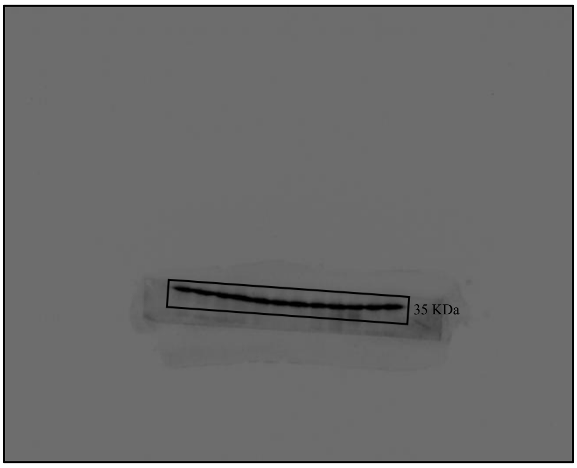

Figure 13C

ATR

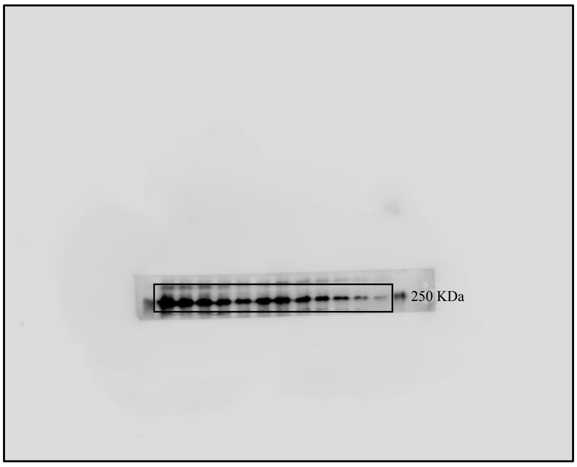

GAPDH

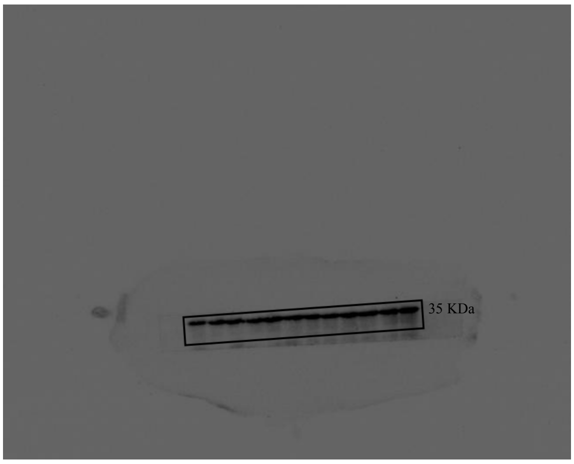

Figure 13E

IP: K63-Ub

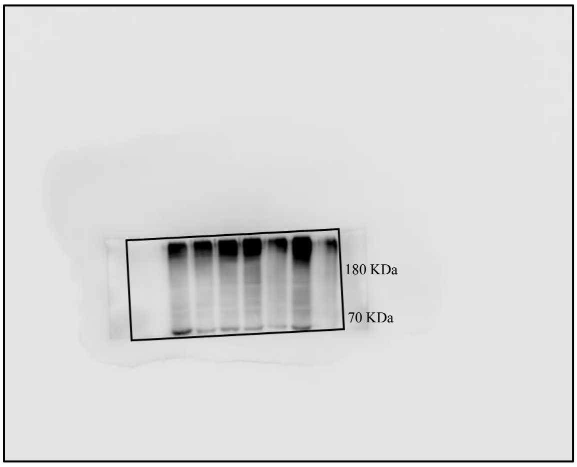

IP: K48-Ub

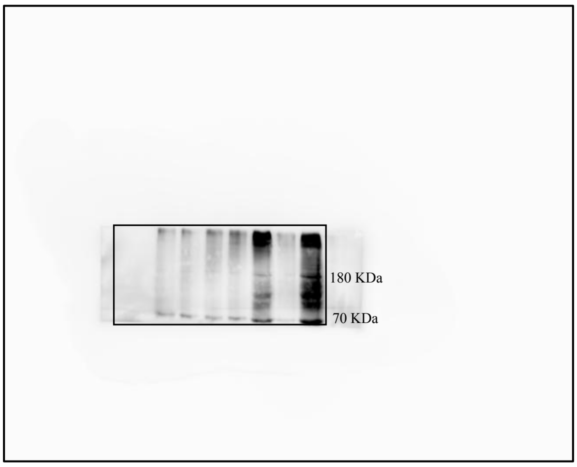

IP:ATR

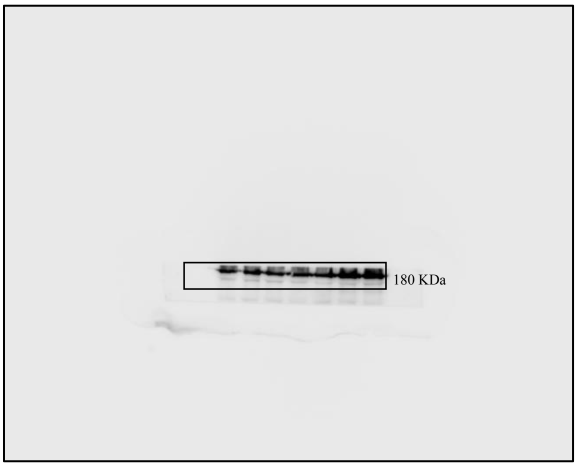

Input: ATR

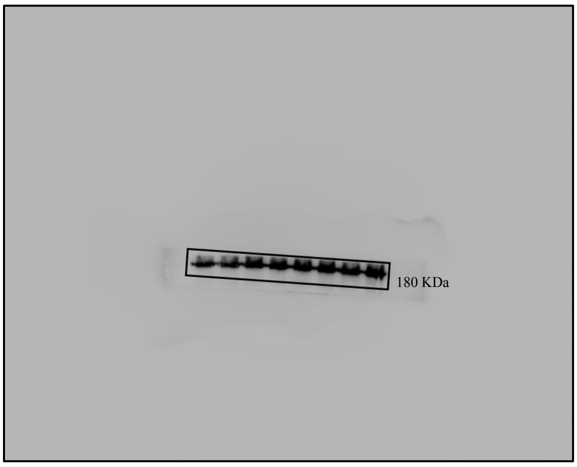

Input: GAPDH

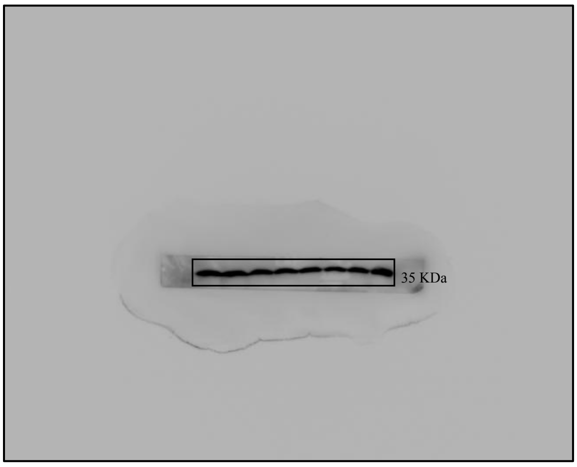

Figure 13F

p-p53

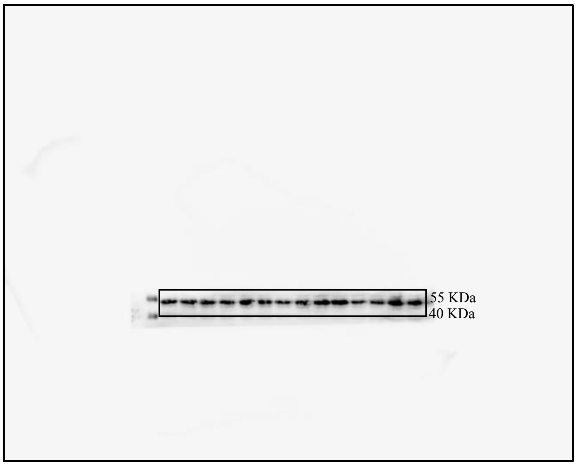

p21

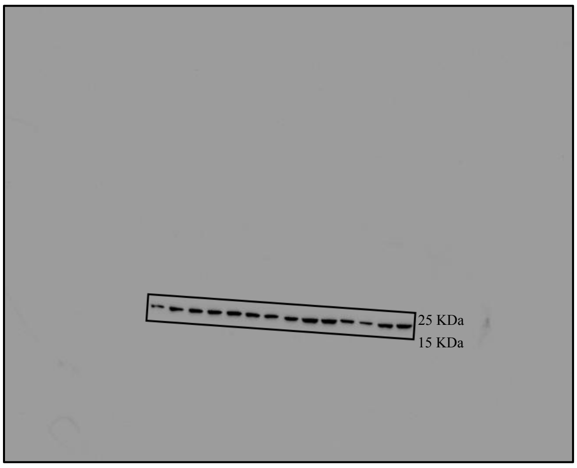

GAPDH

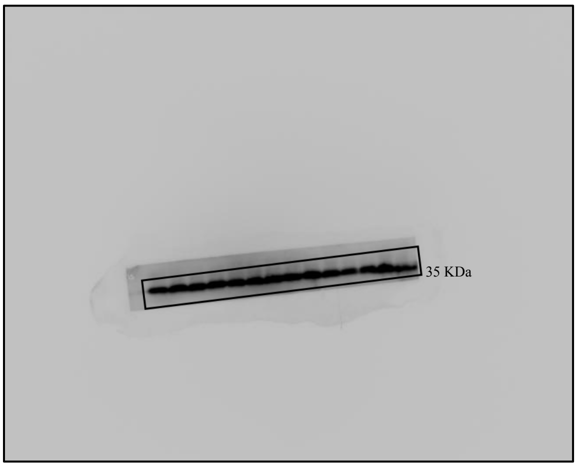

Figure 13G

cGAS

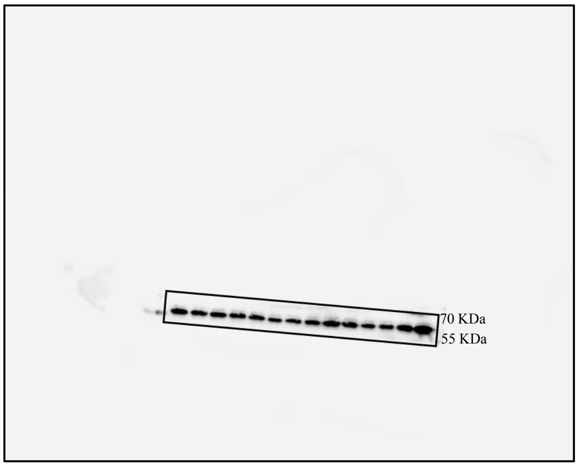

p-STING

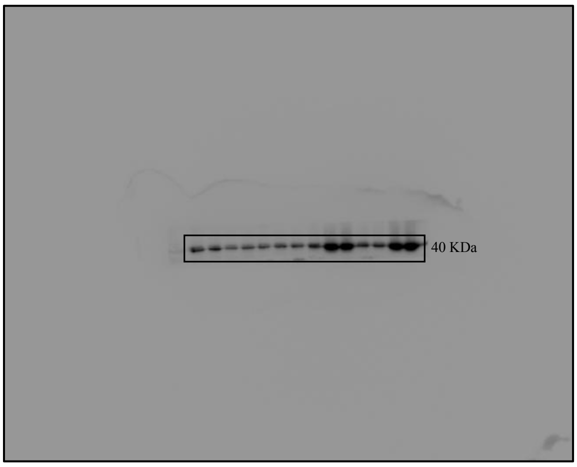

STING

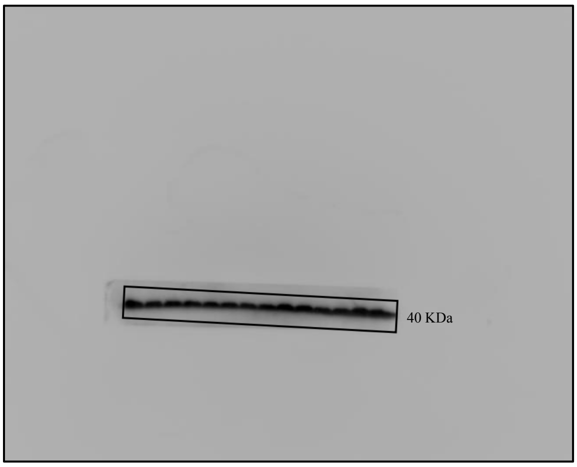

γH2A

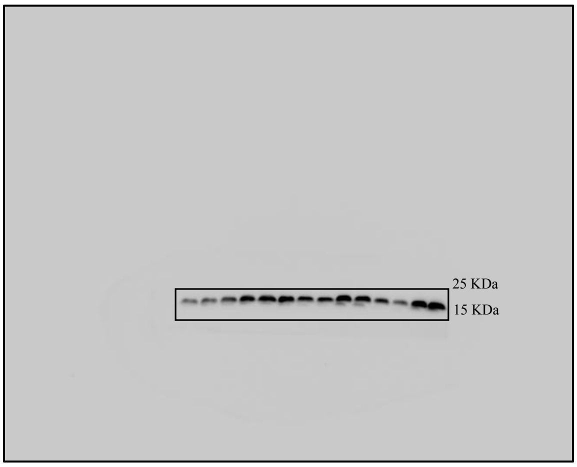

GAPDH

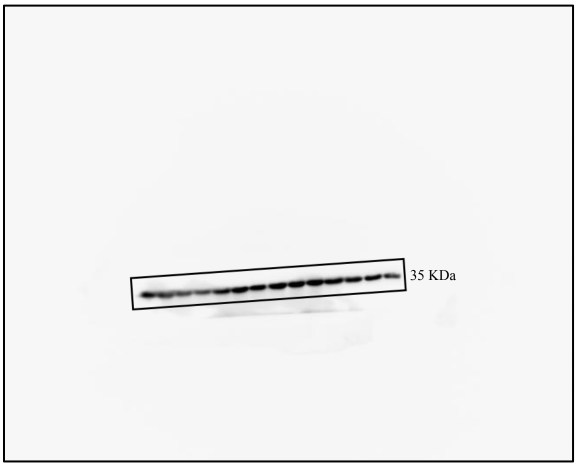

Figure 13I

p-p53

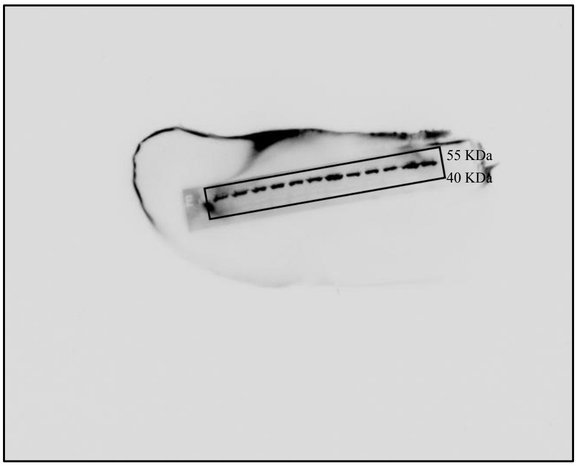

p21

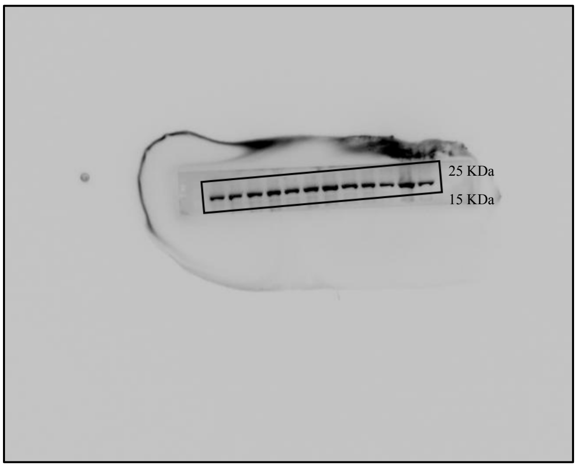

GAPDH

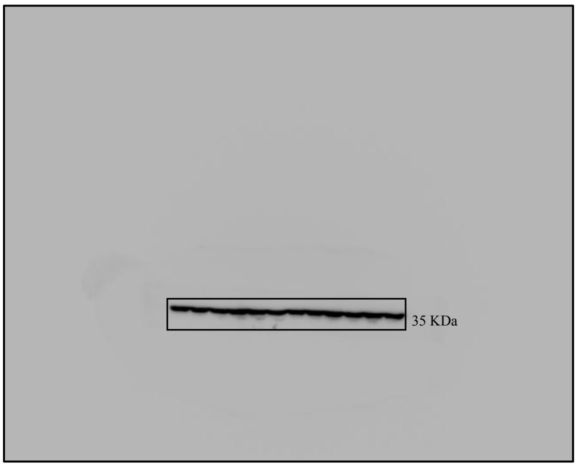

Figure 13J

cGAS

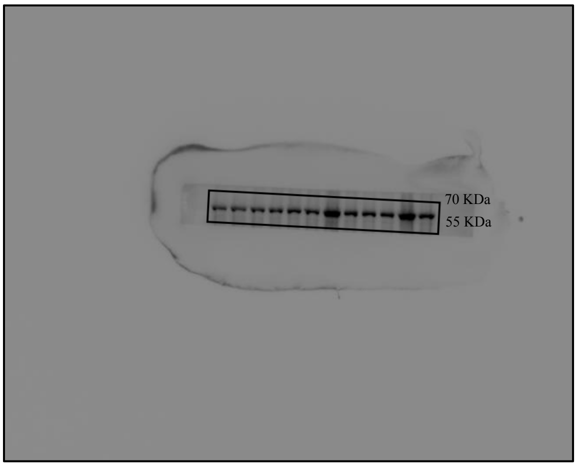

p-STING

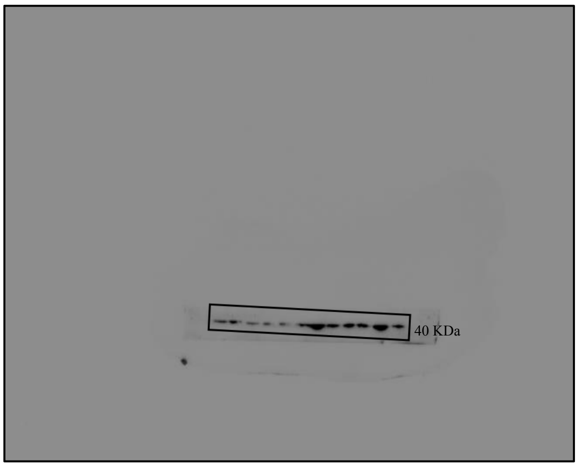

STING

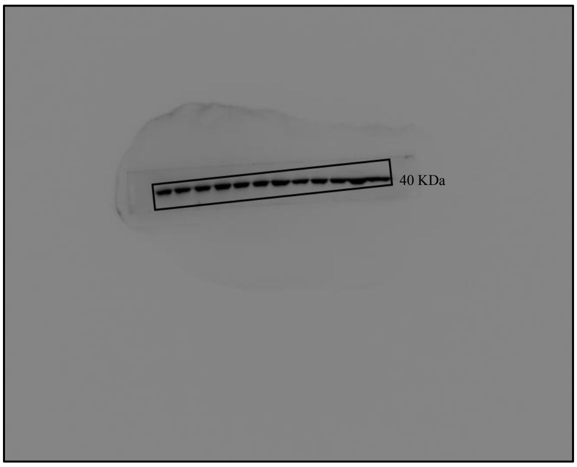

γH2A

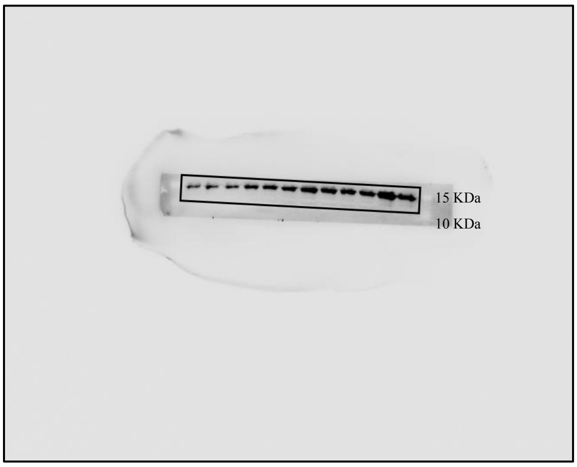

GAPDH

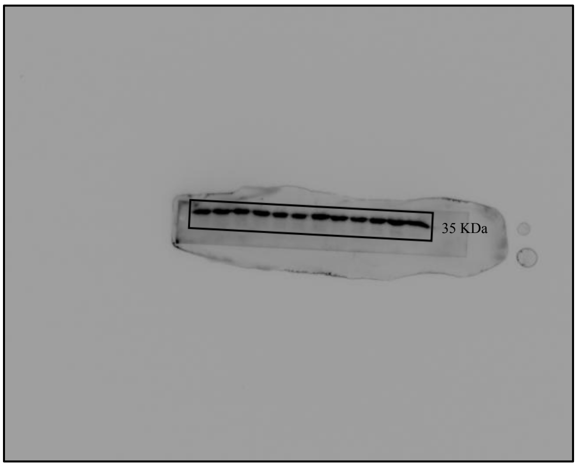

Figure 14C

ATR

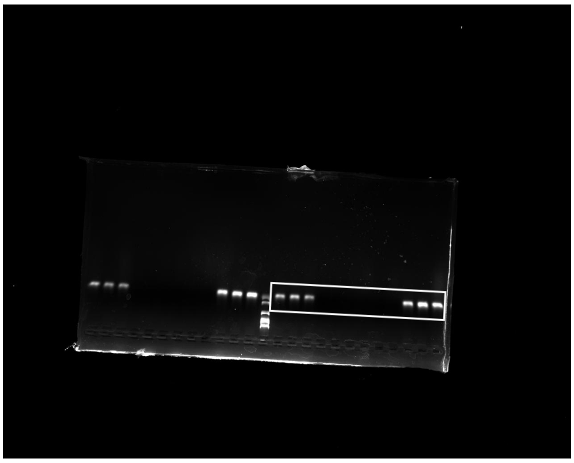

TSG101

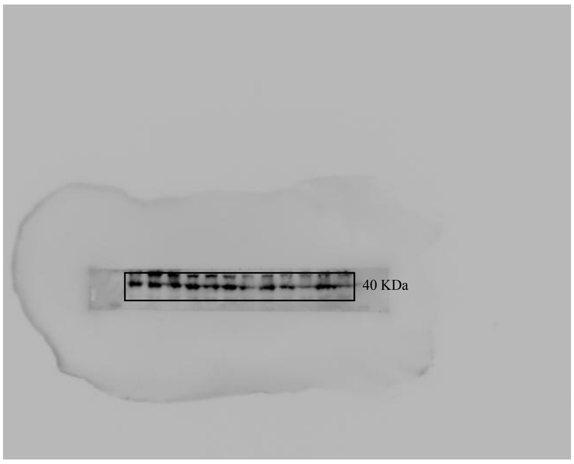

CD63

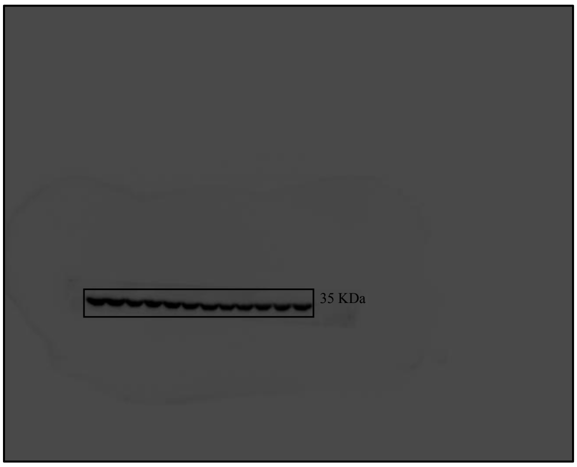

CD9

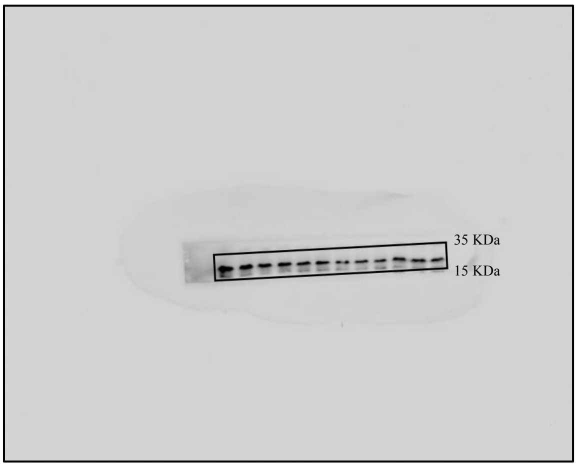

GAPDH

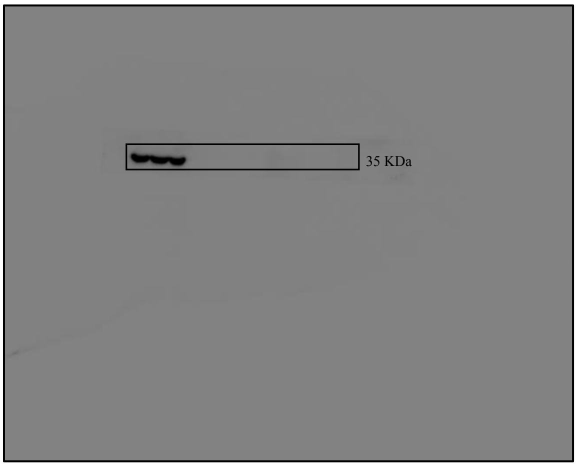

Figure 14D

ATR

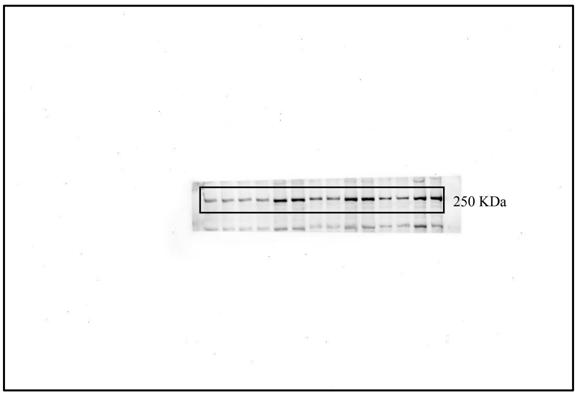

GAPDH

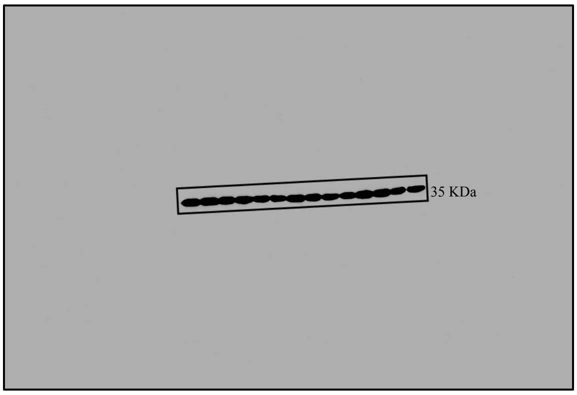

Figure 14E

p-p53

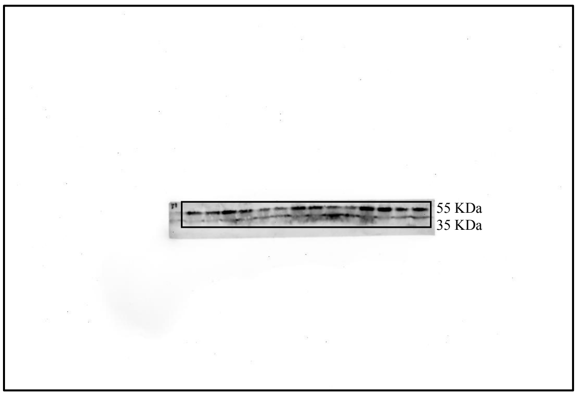

p21

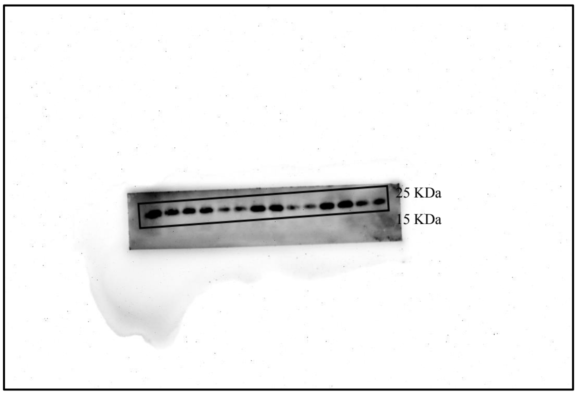

p16

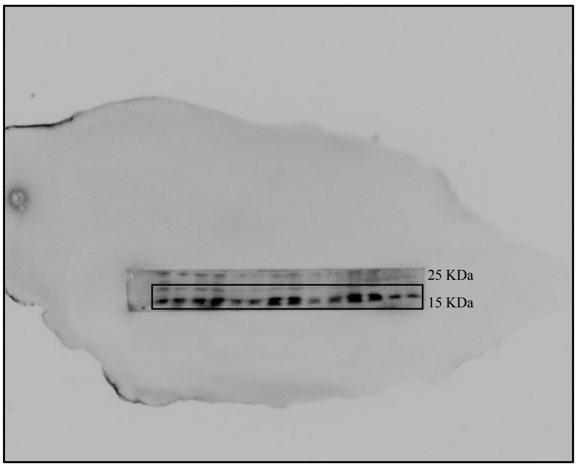

GAPDH

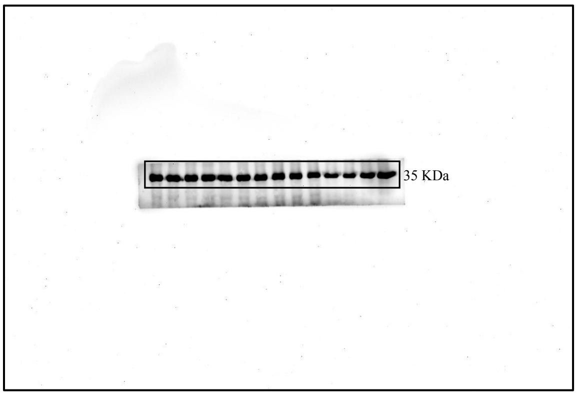
35 KDa

Figure 14F

cGAS

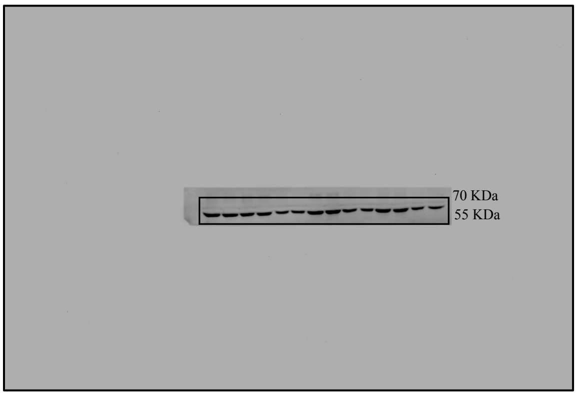

STING

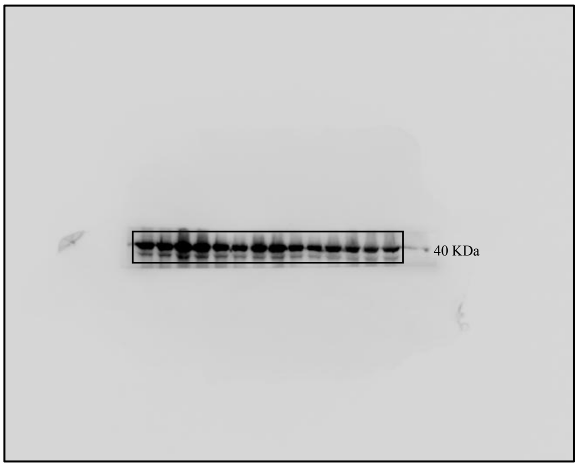

γH2A

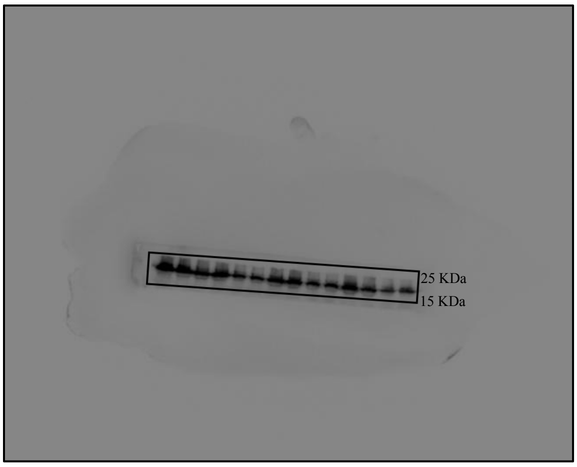

GAPDH

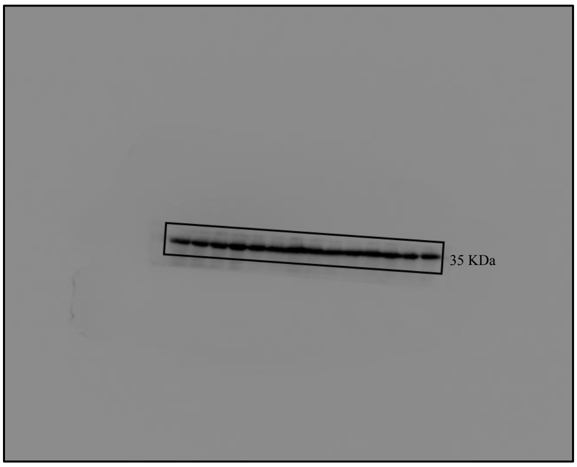
35 KDa

Figure S1H

Tubulin-PCNA

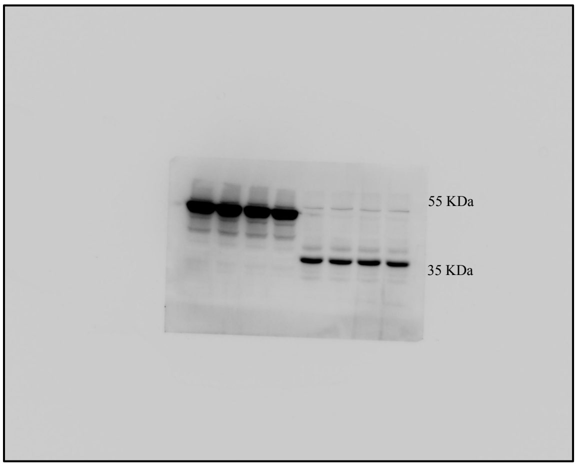

Figure S1K

IB: AIM2

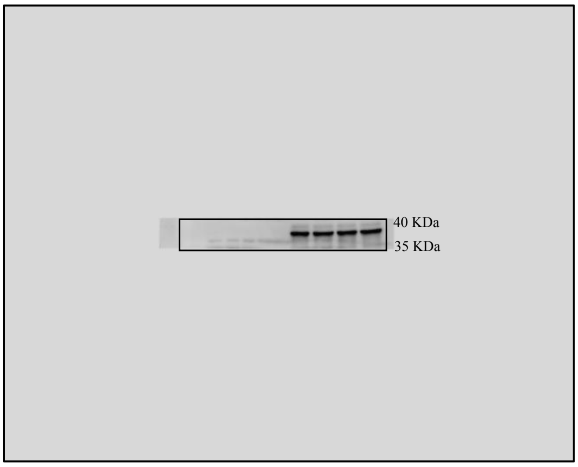

IB: pro caspase-1

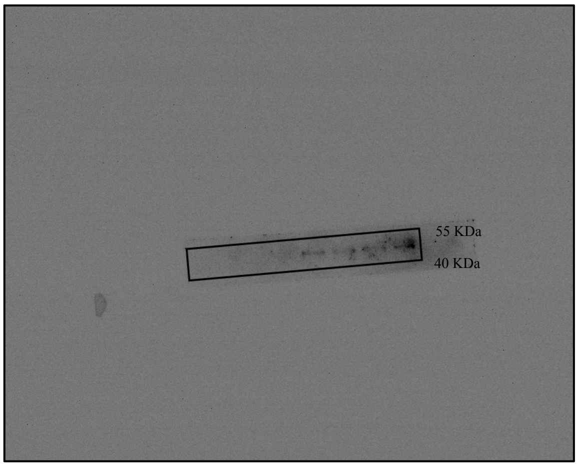

IB: ASC

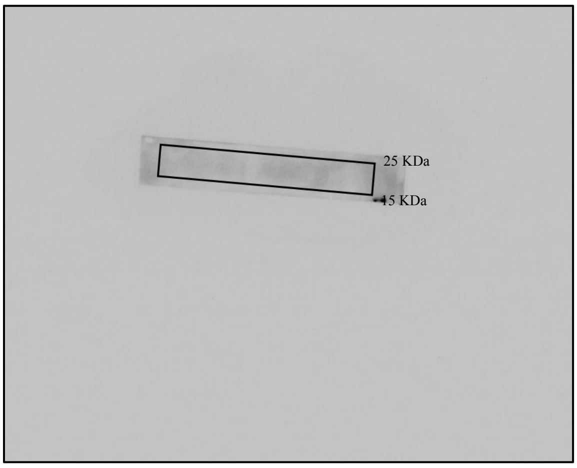

Input: AIM2

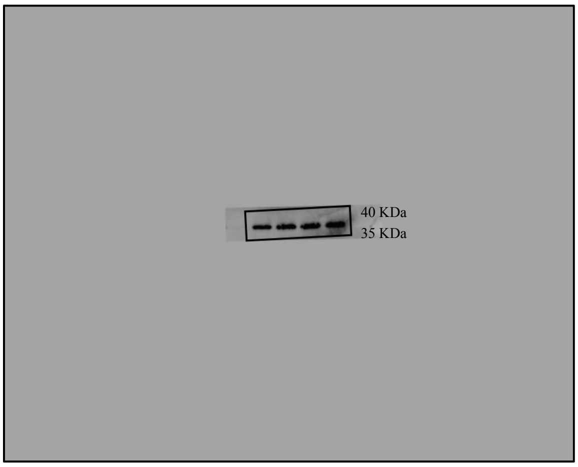

Input: pro-caspase-1

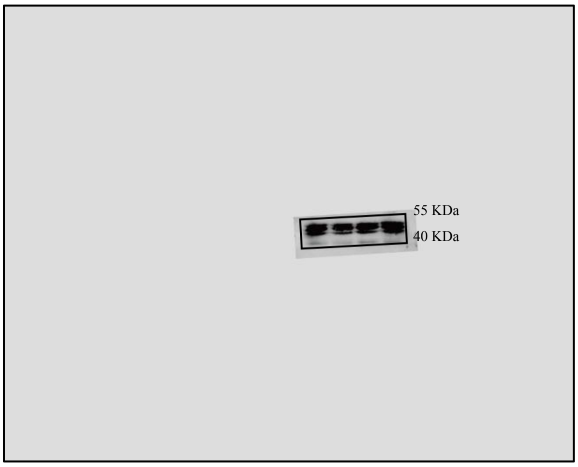

Input: ASC

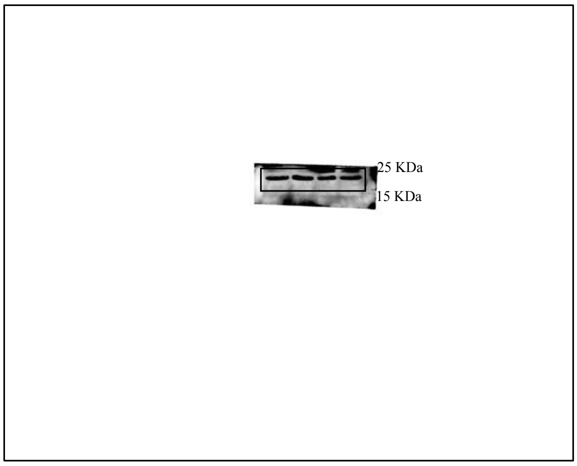

Input: GAPDH

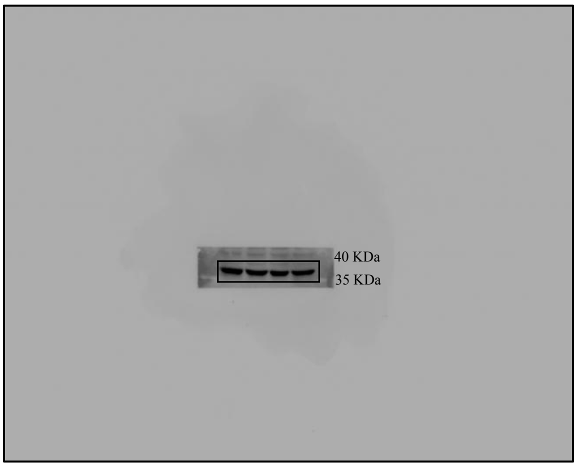
40 KDa
35 KDa

Figure S1L

gDNA：POLG1; LAD12

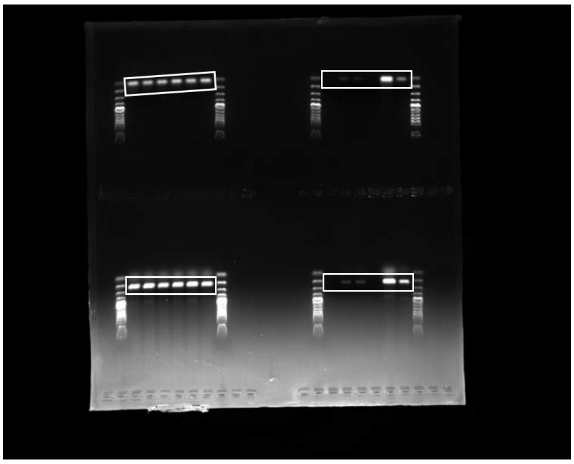

gDNA: L1ORF1; 18S RNA

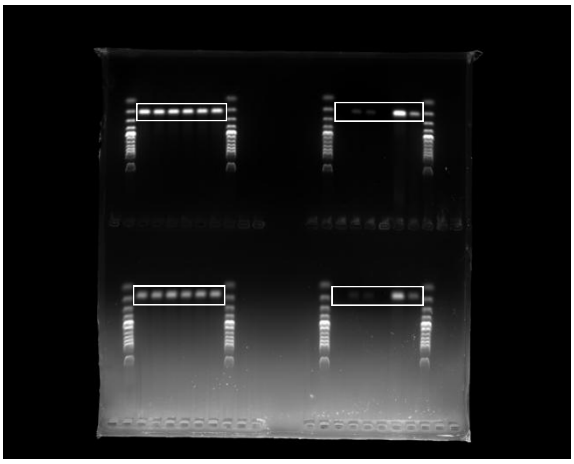

IB: cGAS

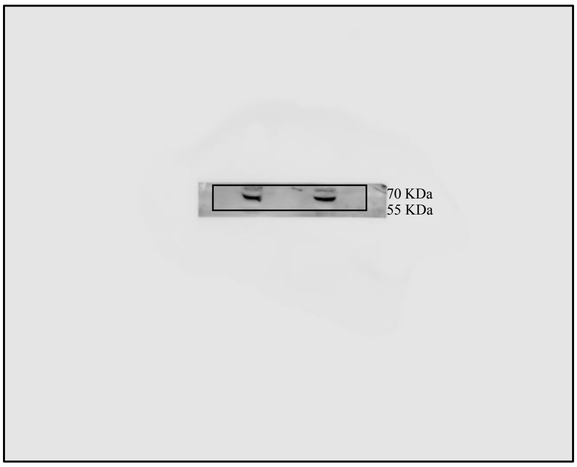

IB: AIM2

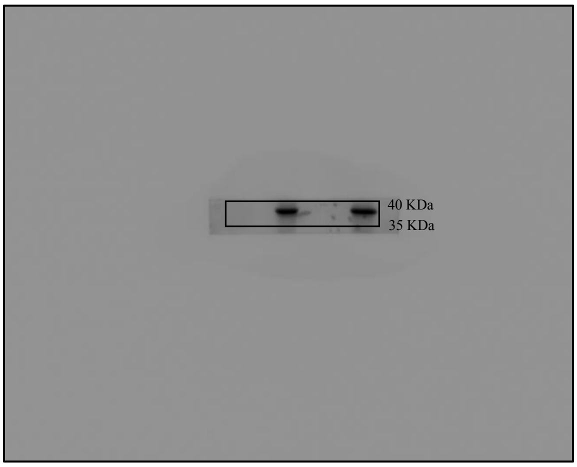

Input: cGAS

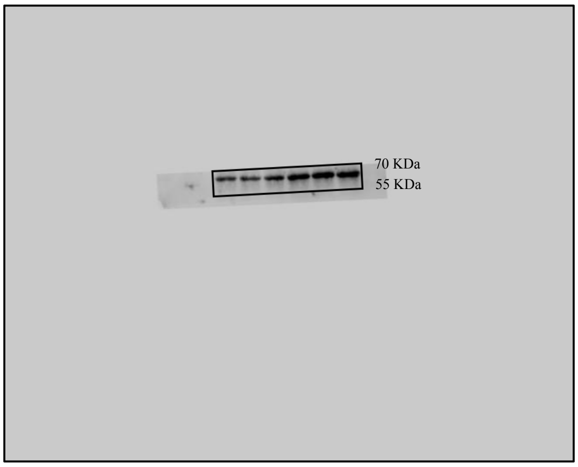

Input: AIM2

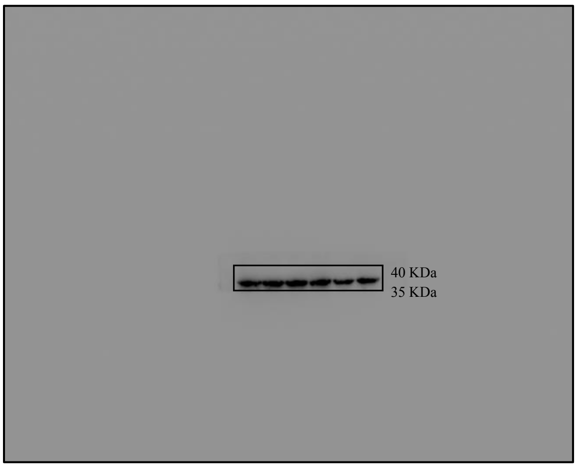

Input: β-actin

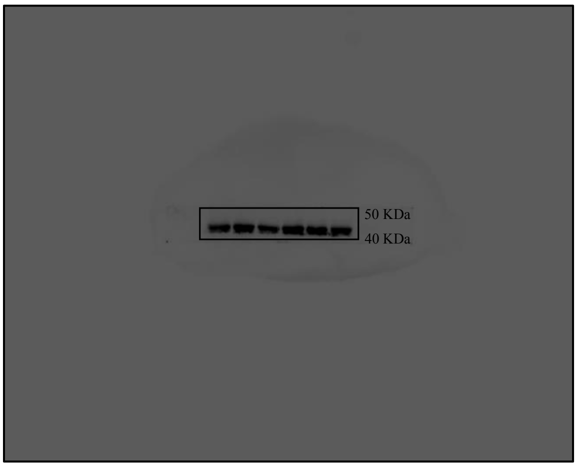
50 KDa
40 KDa

Figure S1M

cGAS

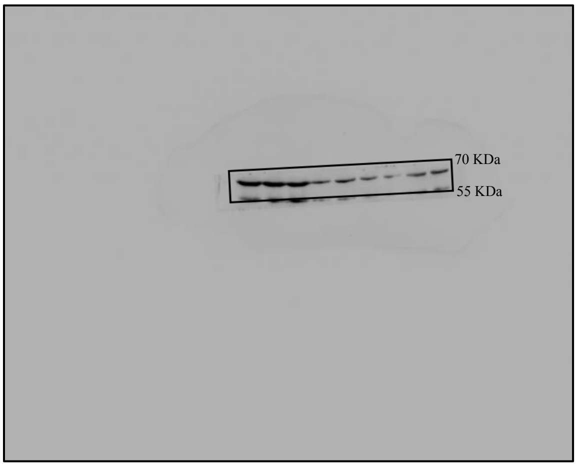

GAPDH

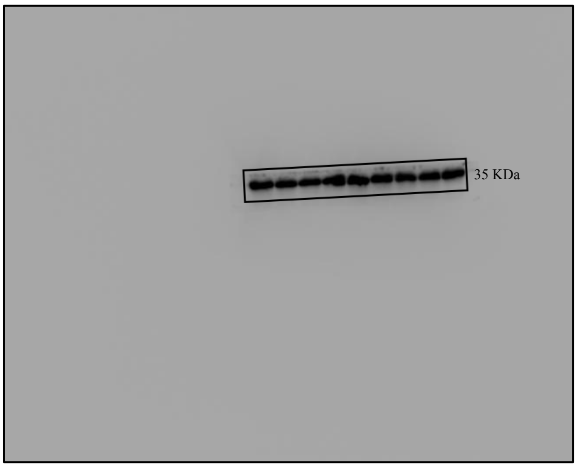

AIM2

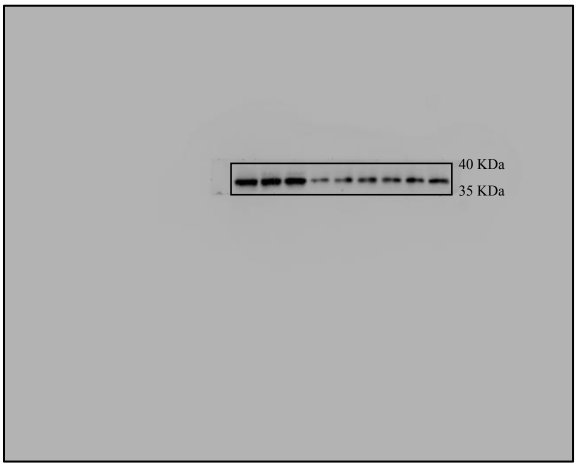

GAPDH

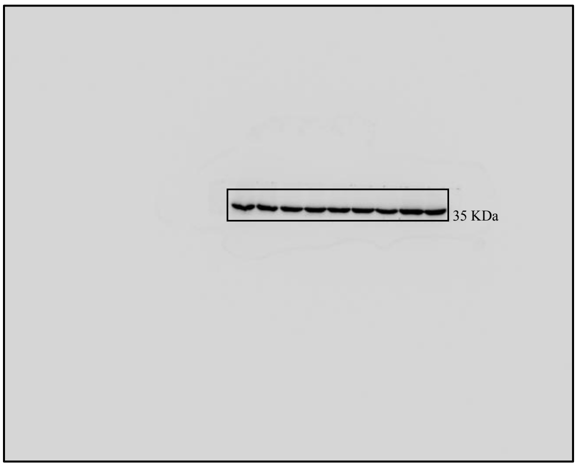
35 KDa

Figure S1N

53BP

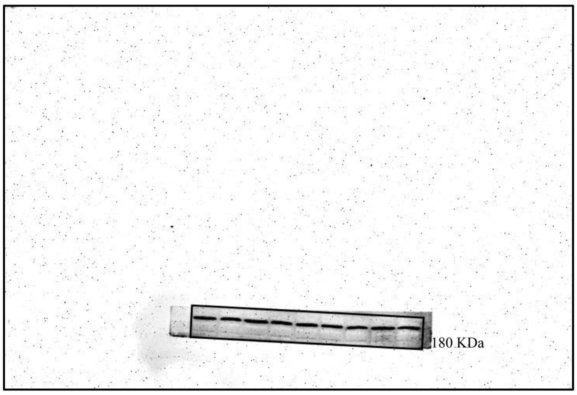

γH2A

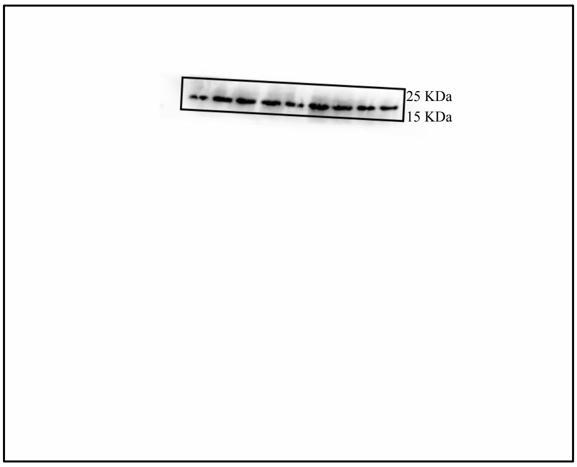

GAPDH

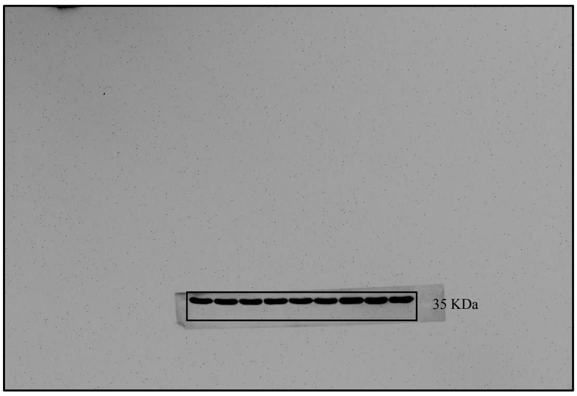

53BP

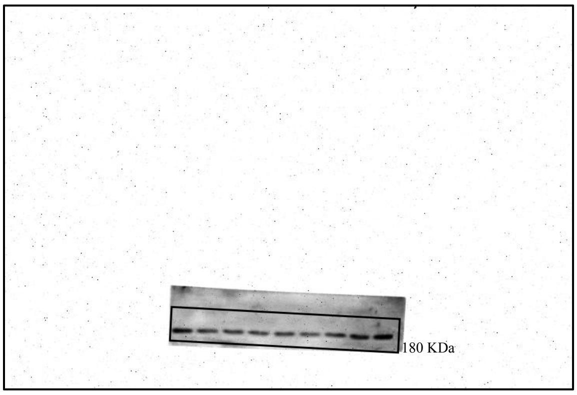

γH2A

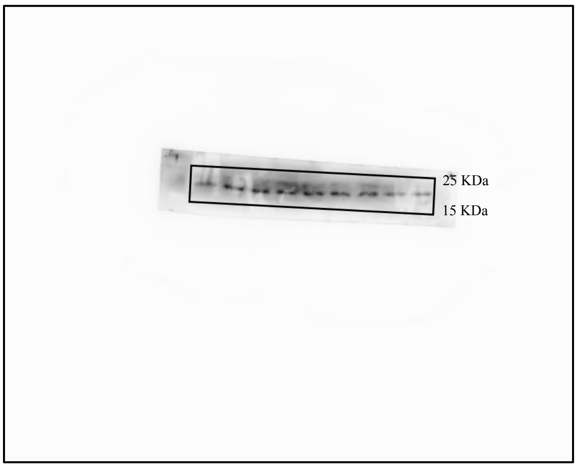

GAPDH

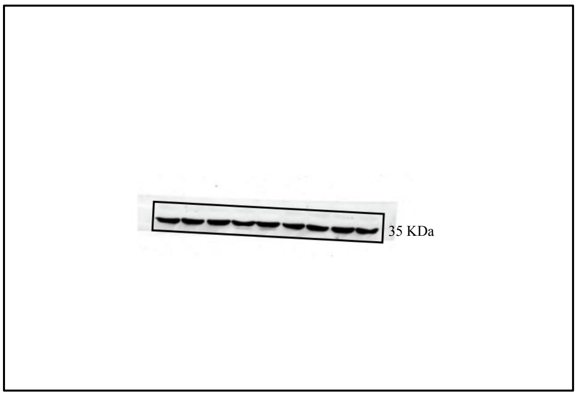

Figure S1O

p-p53

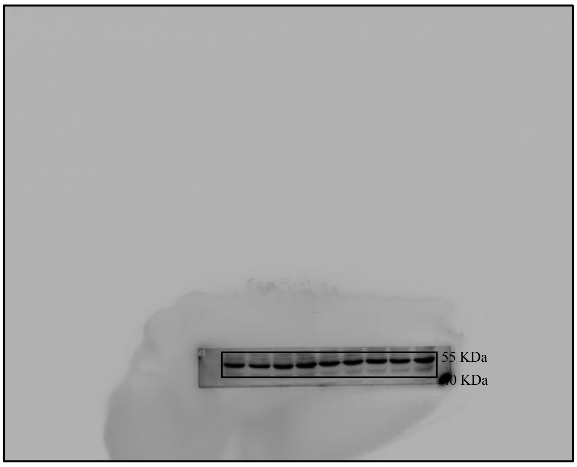

p21

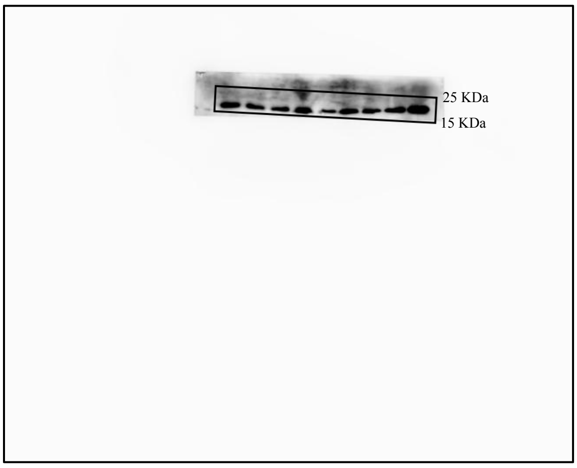

GAPDH

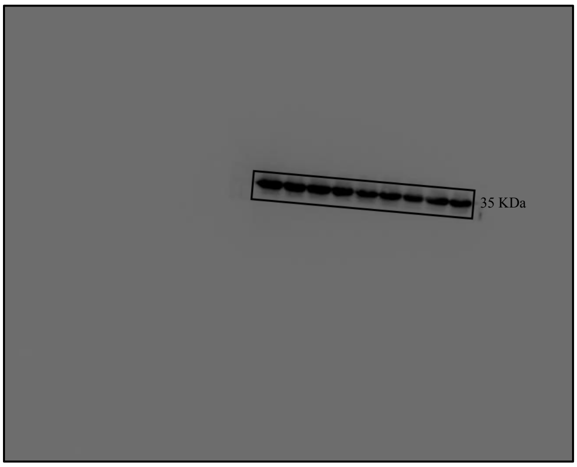

p-p53

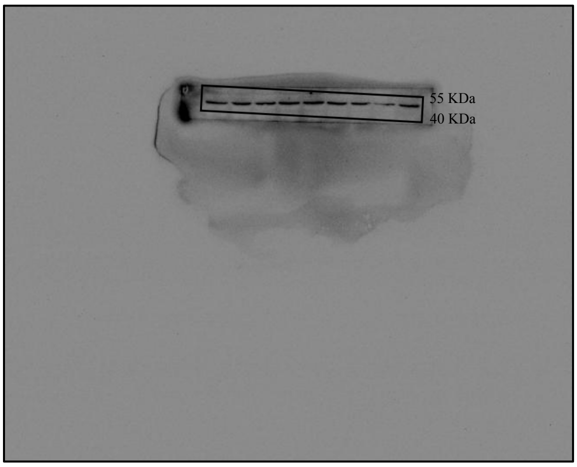

p21

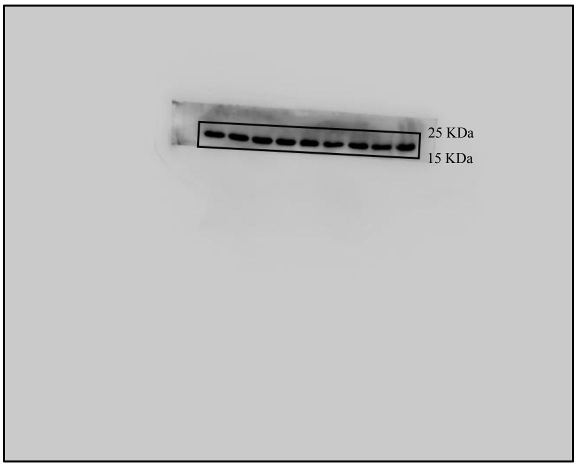

GAPDH

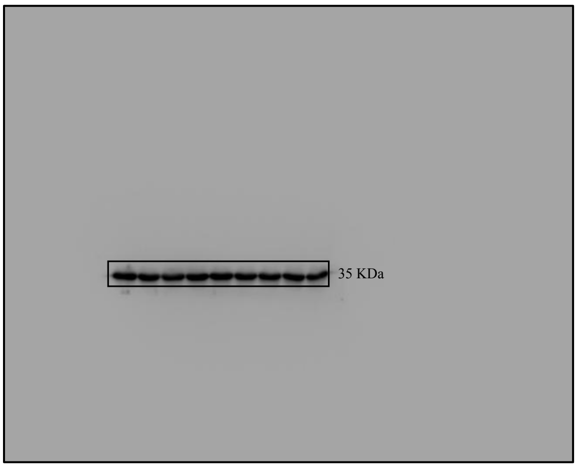

Figure S1P

cGAS

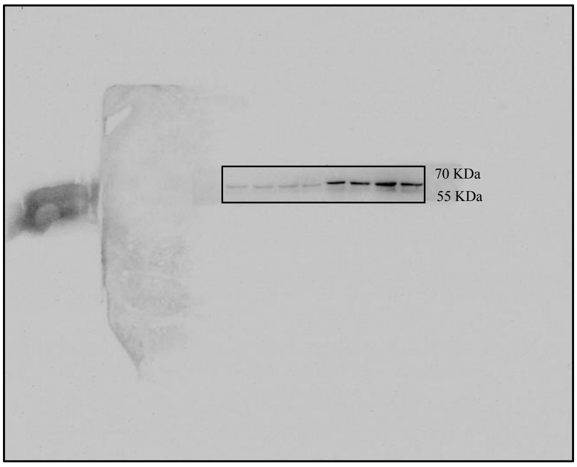

p-STING

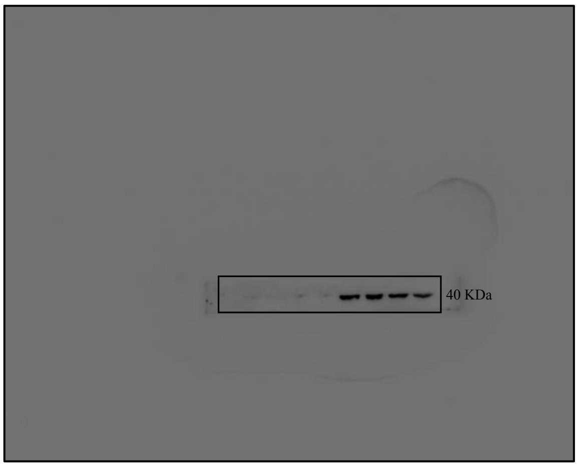

STING

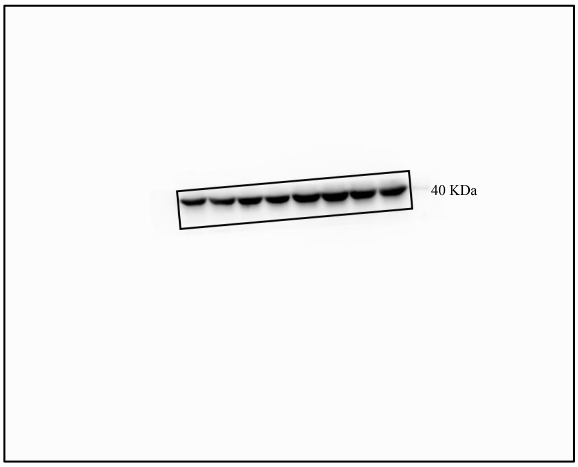

GAPDH

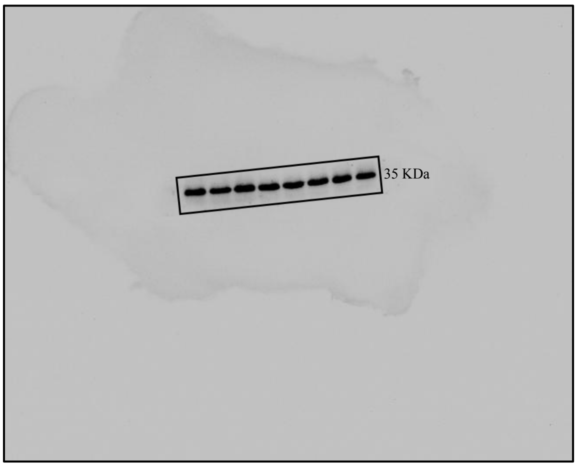
35 KDa

Figure S3B

NLRP3

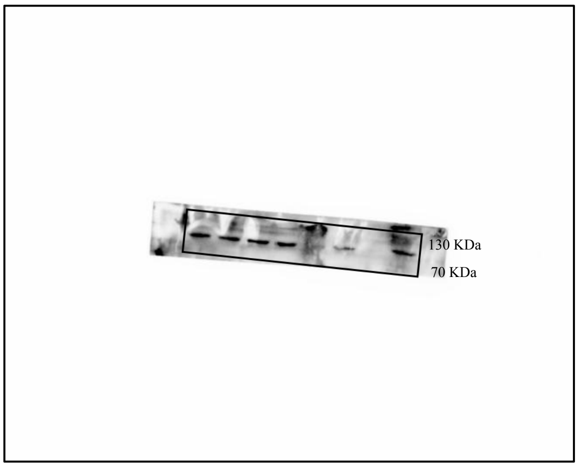

Caspase-1

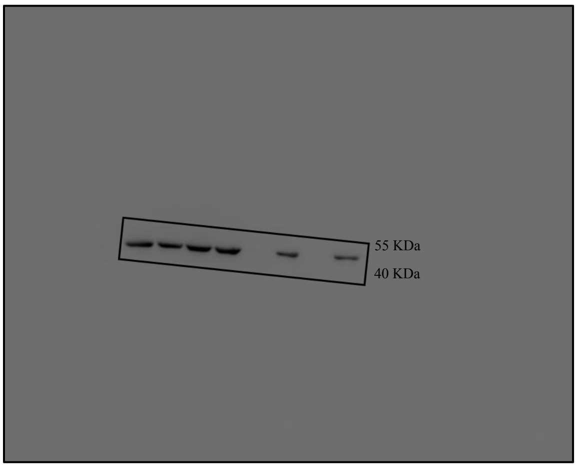

ASC

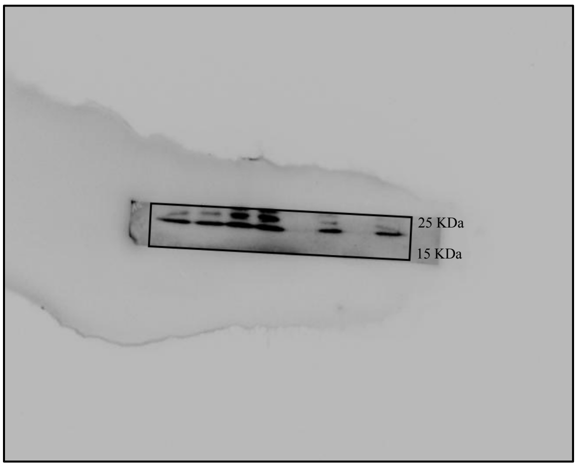

GAPDH

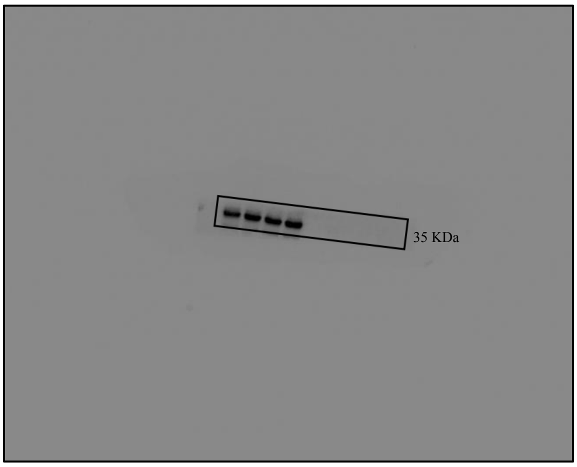
35 KDa

Figure S3D

NLRP3

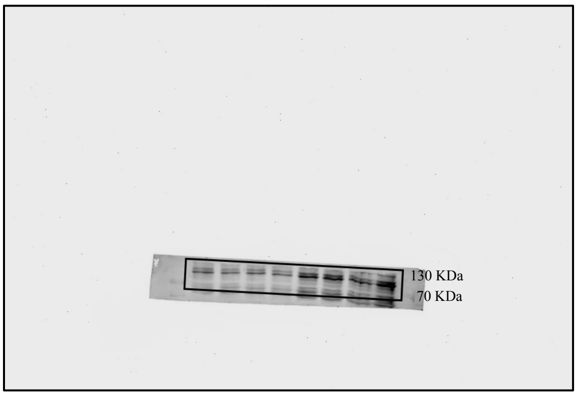

caspase-1

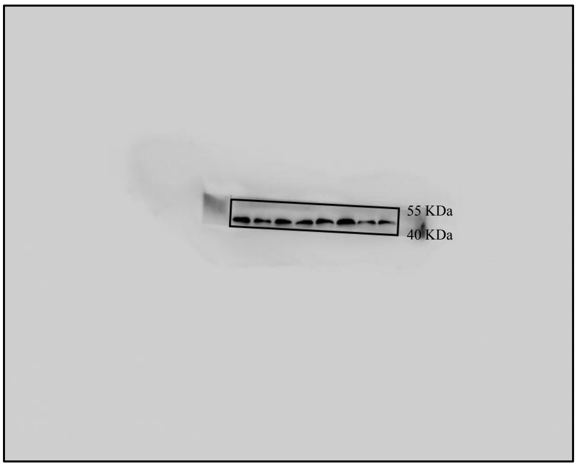

ASC

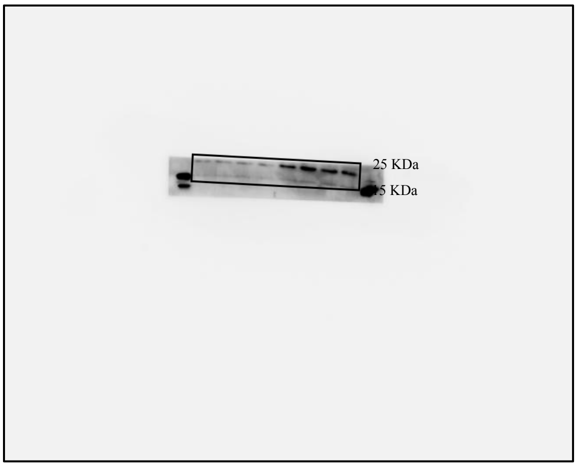

GSDMD

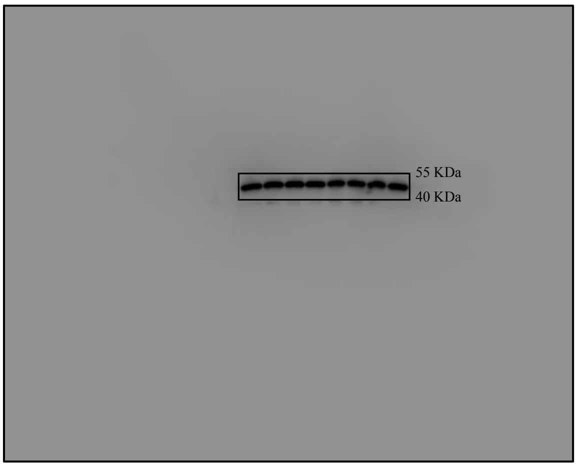

Cleaved caspase-1

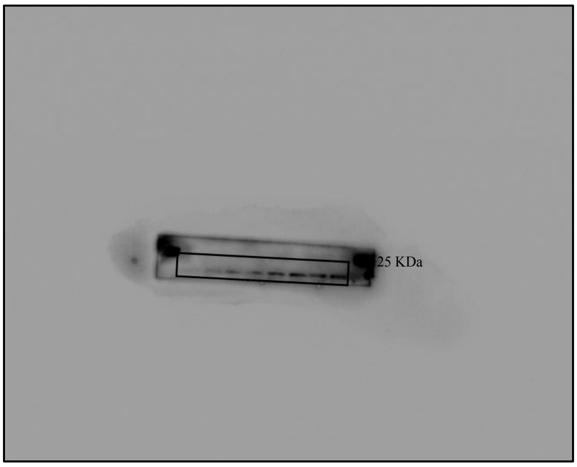

Cleaved GSDMD

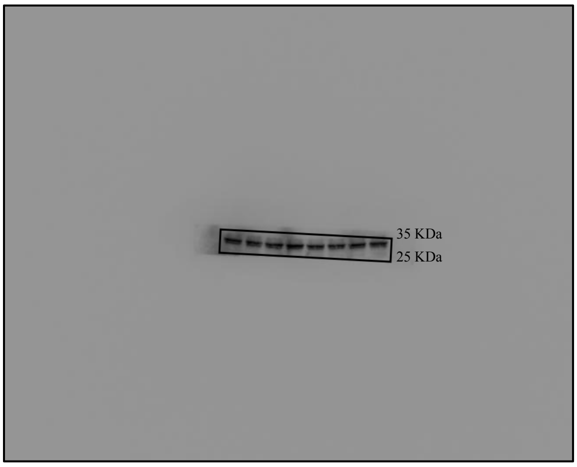

GAPDH

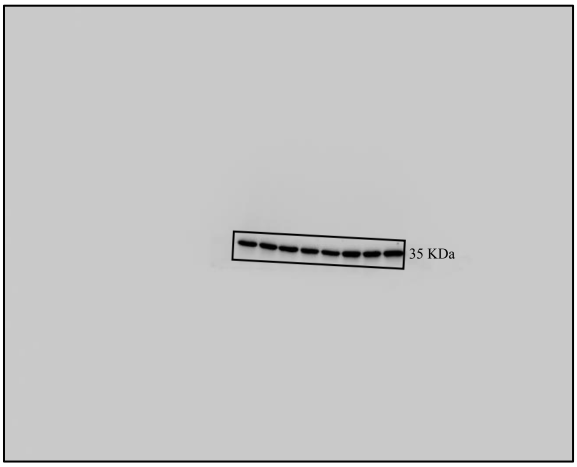
35 KDa

Figure S3F

NLRP3

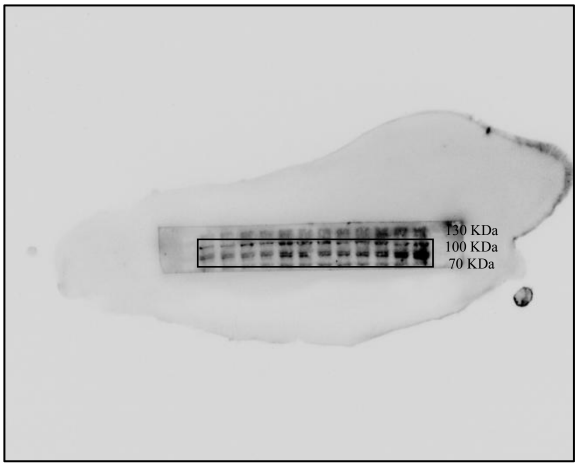

GSDMD

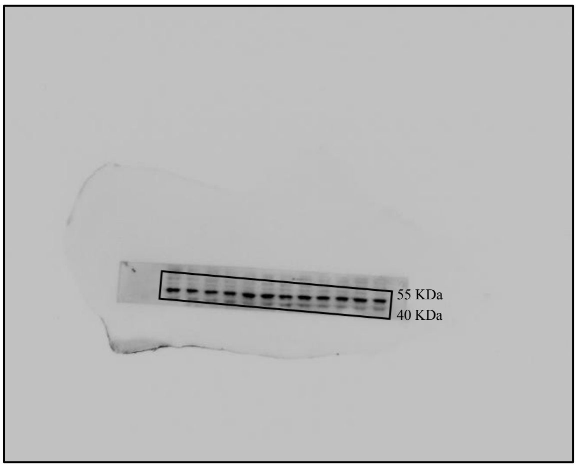

Cleaved GSDMD

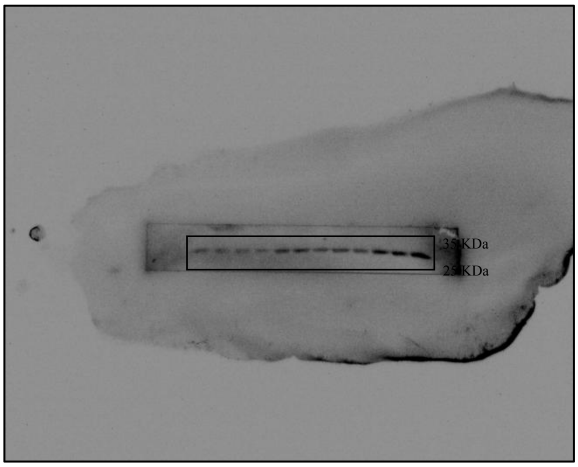

Caspase-1

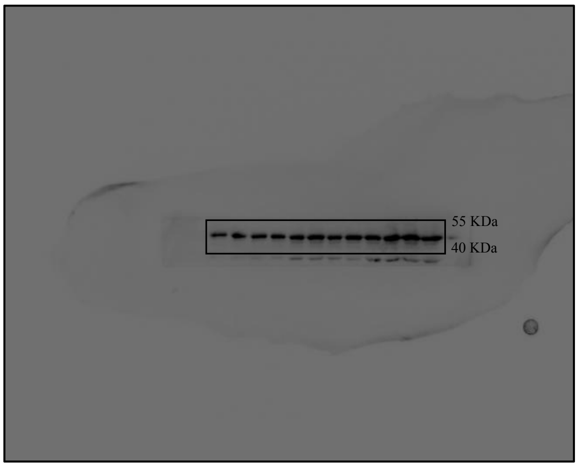

Cleaved caspase-1

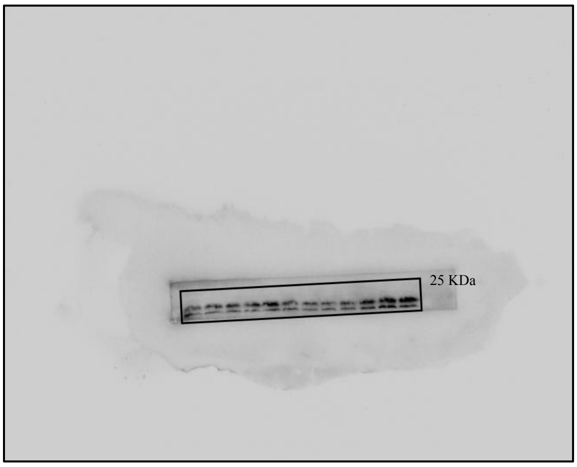

GAPDH

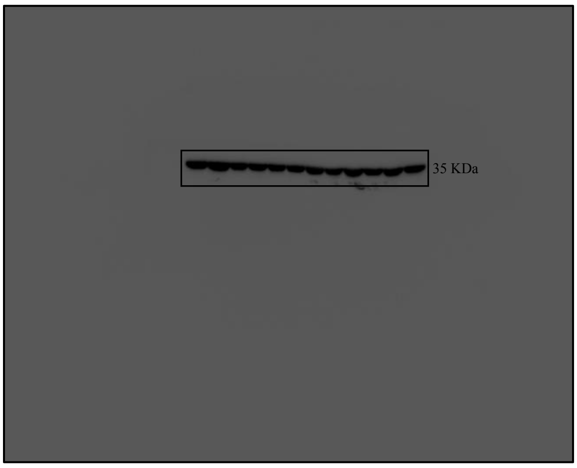

Figure S3G

IP:NLRP3-IP

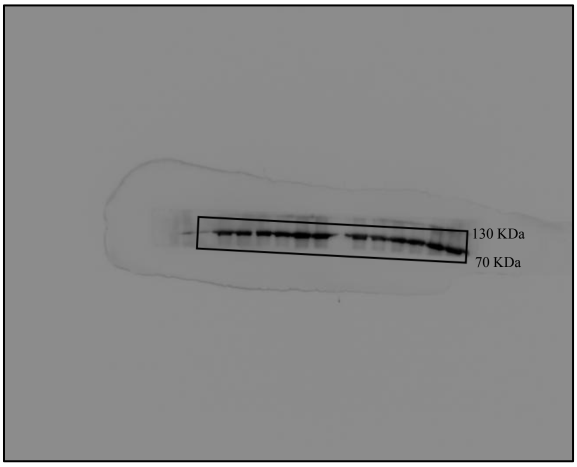

IP: caspase-1-IP

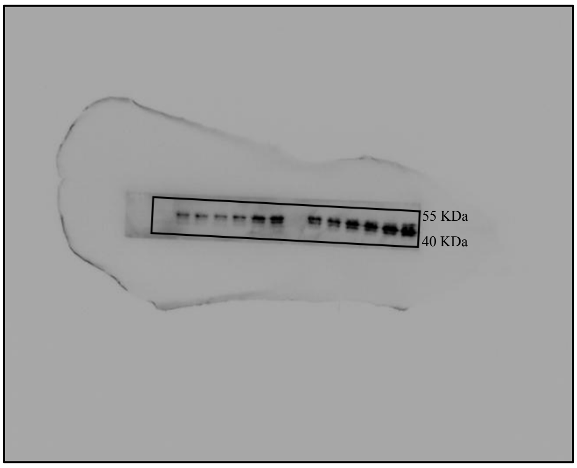

IP: ASC

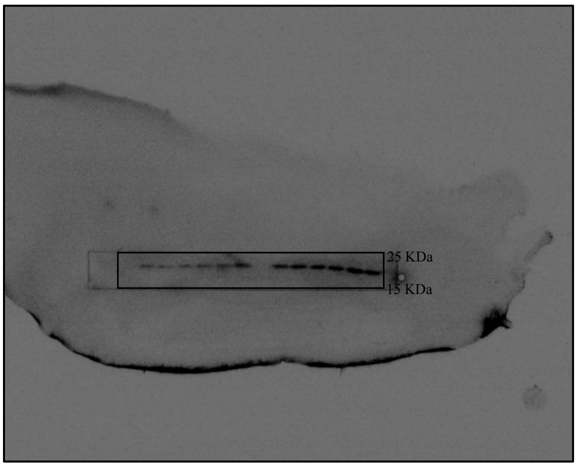

Input: NLRP3

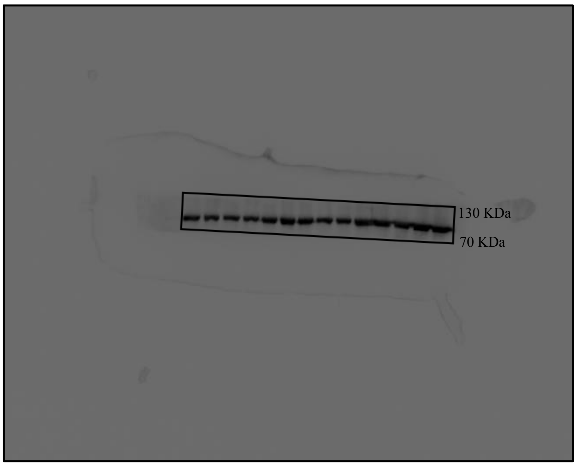

Input: Caspase-1

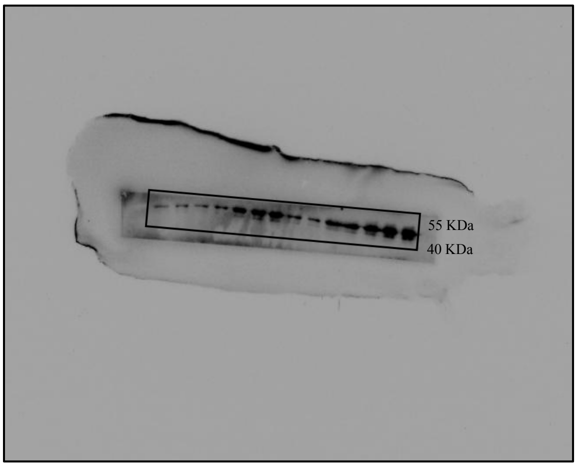

Input: ASC

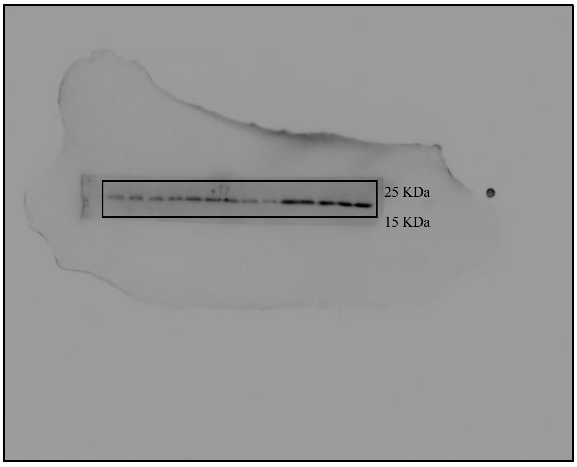

Input: GAPDH

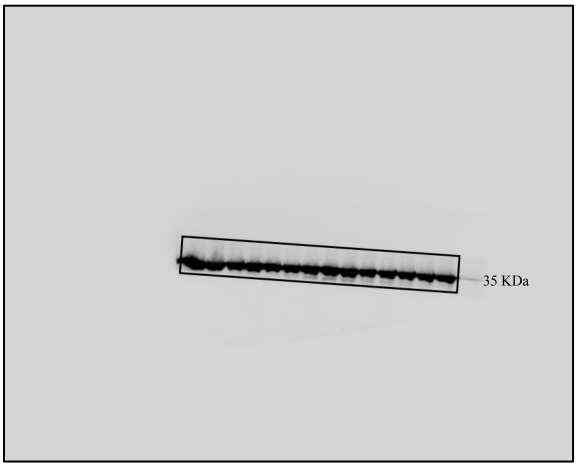
35 KDa

Figure S4A

cDNA: ATR

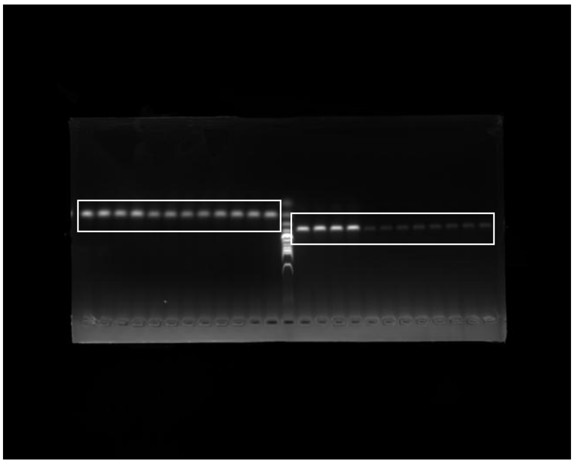

ATR

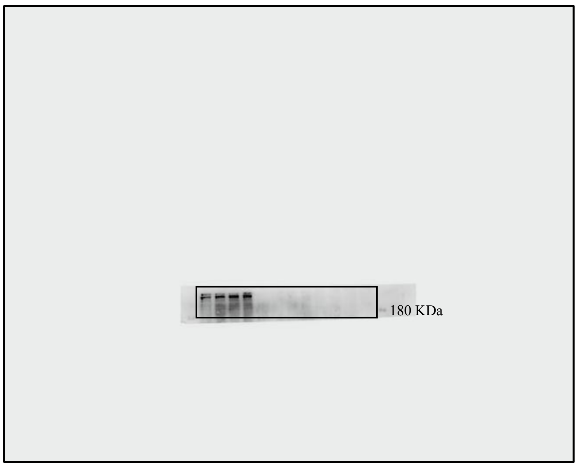

GAPDH

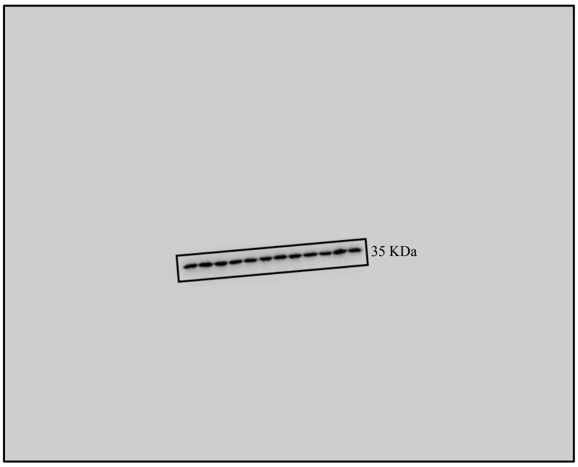

Figure S4D

p-p53

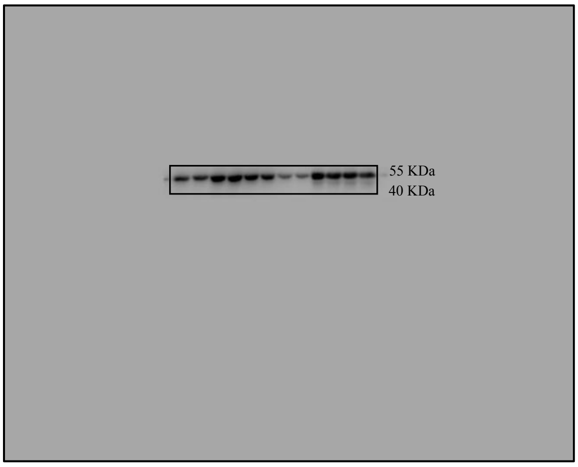

p21

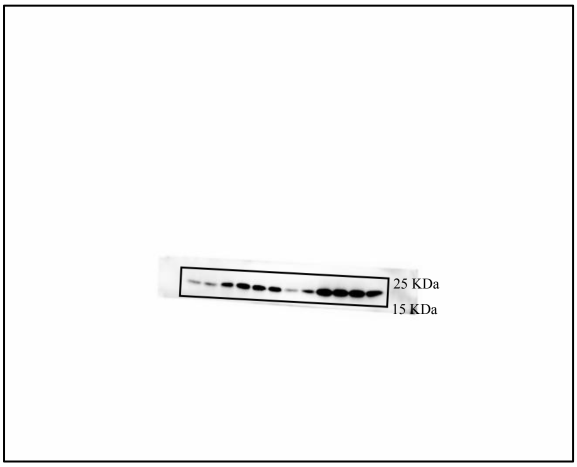

GAPDH

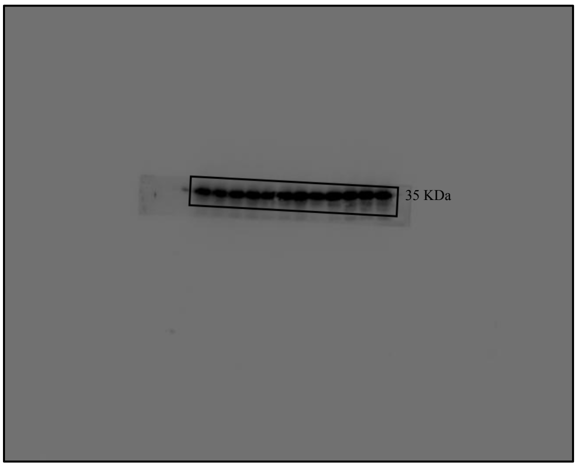

Figure S4E

cGAS

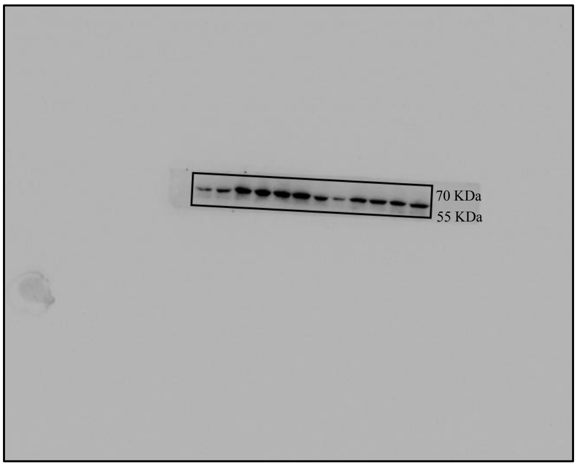

p-STING

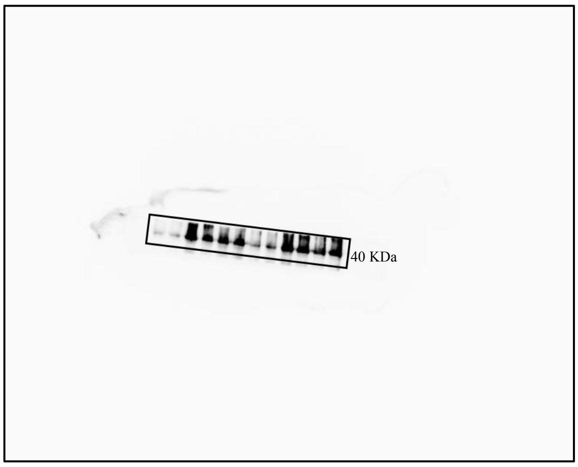

STING

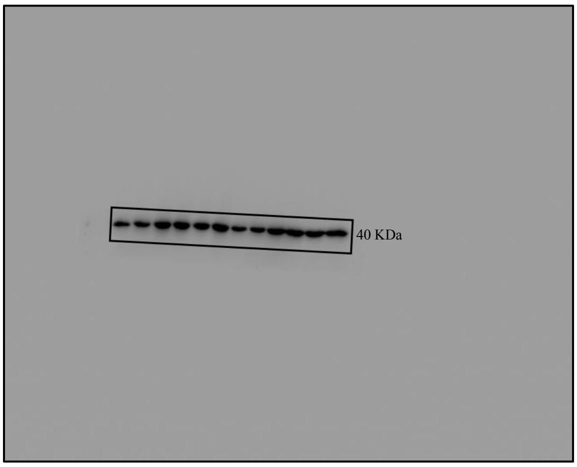

γH2A

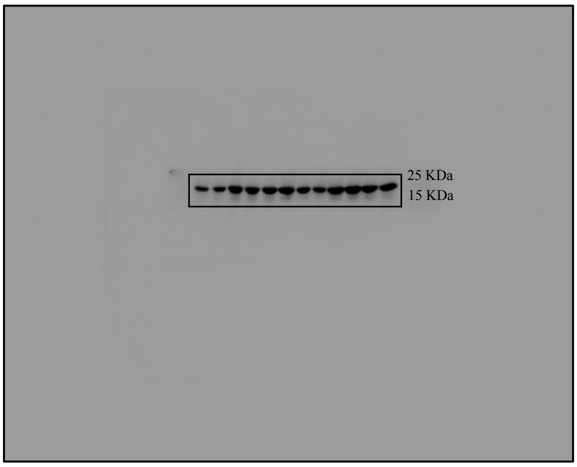

GAPDH

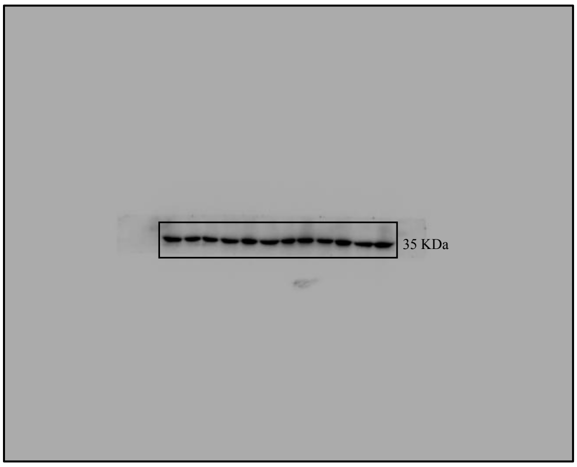

Figure S4F

ATR

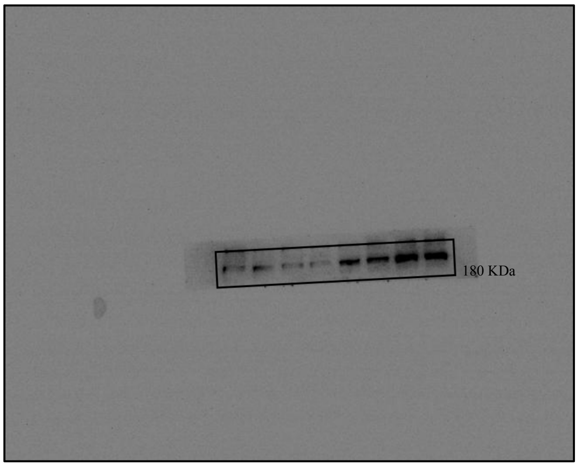

GAPDH

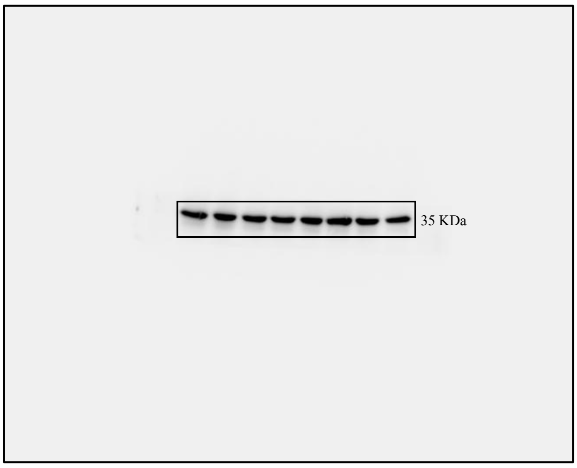

Figure S4G

p-p53

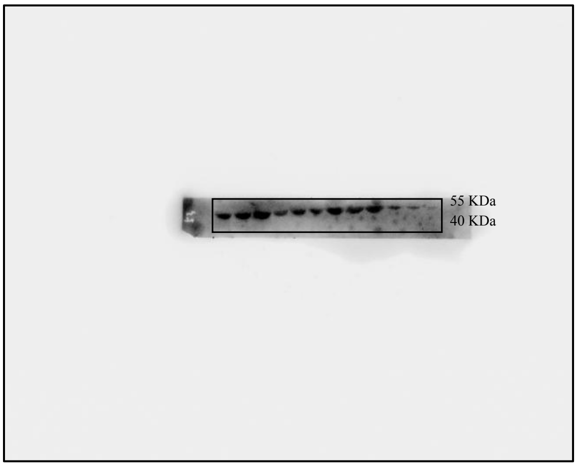

p21

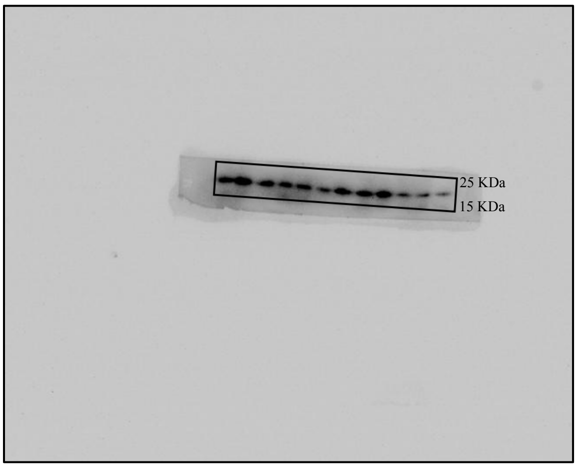

GAPDH

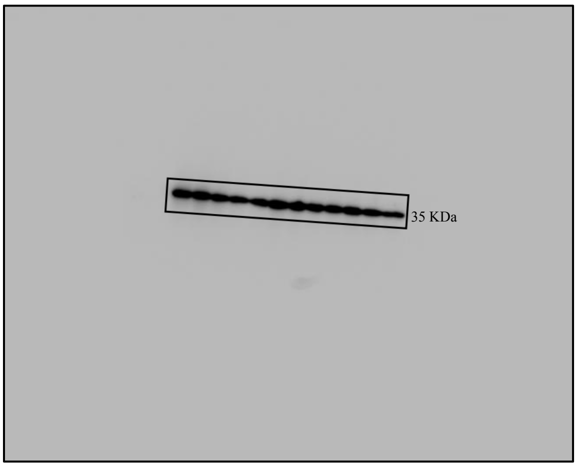

Figure S4K

cGAS

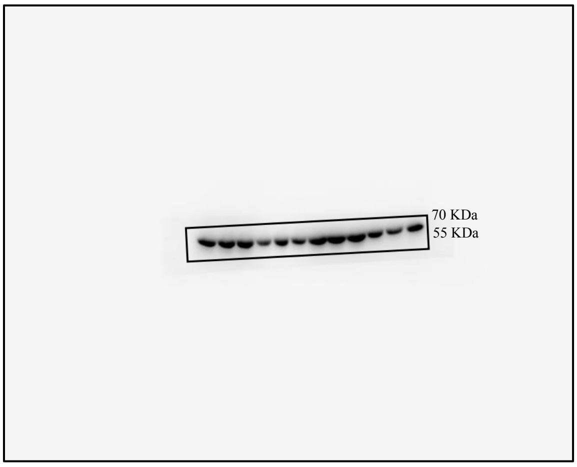

p-STING

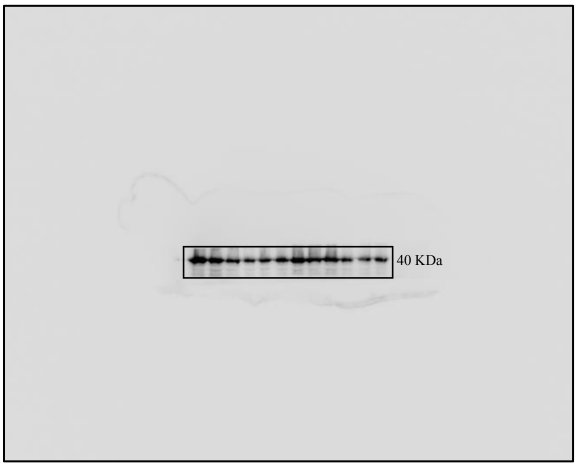

STING

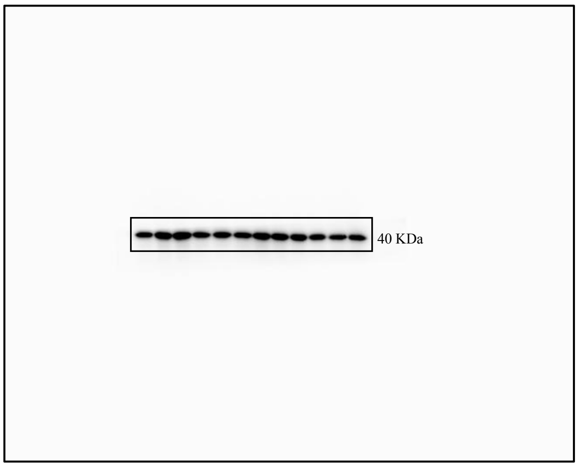

γH2A

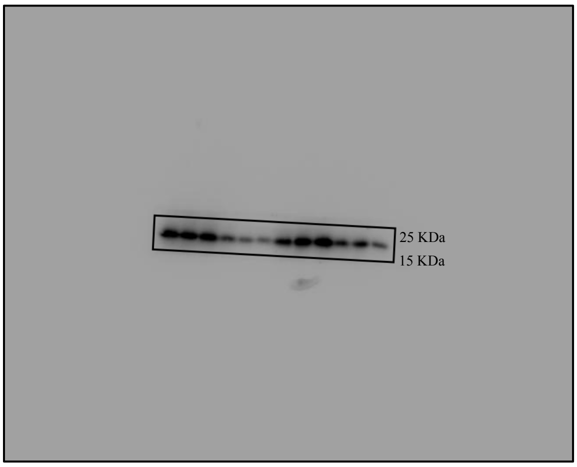

GAPDH

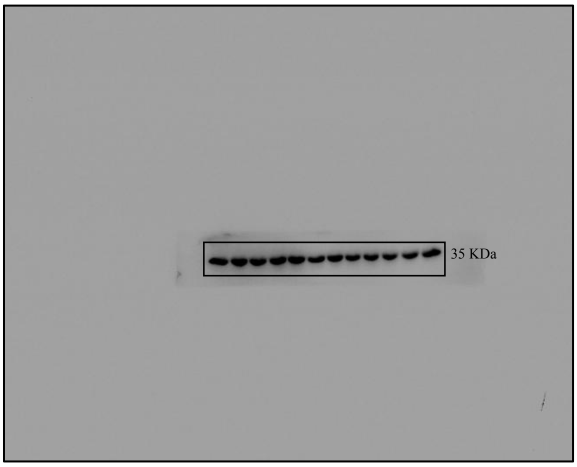

Figure S4N

p-p53

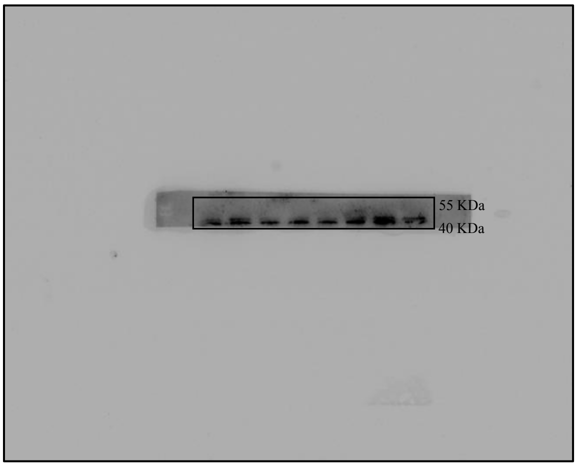

p21

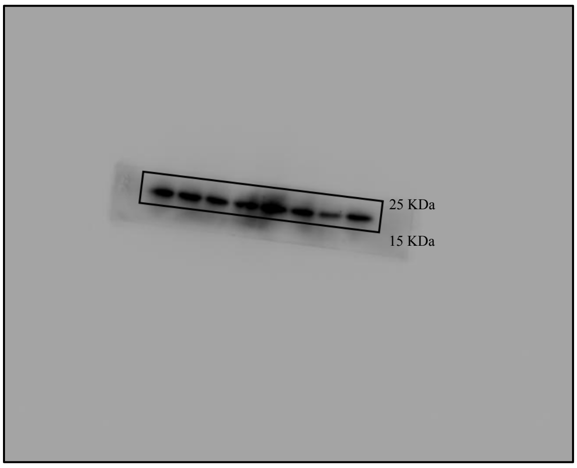

p16

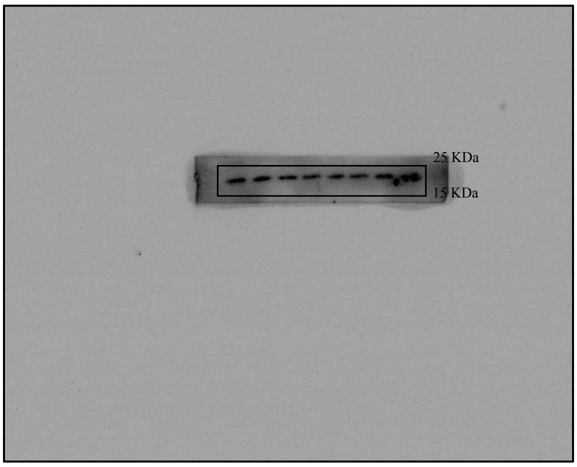

GAPDH

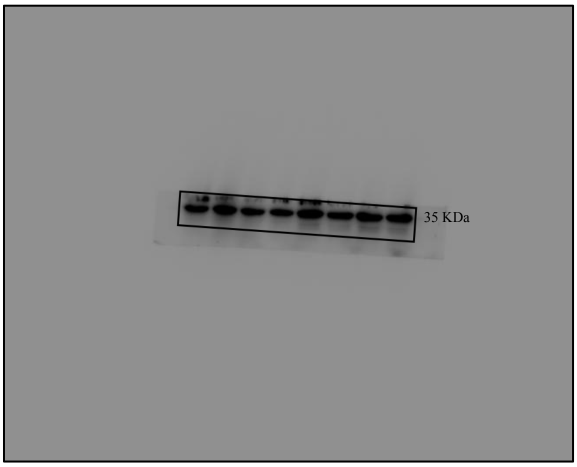

cGAS

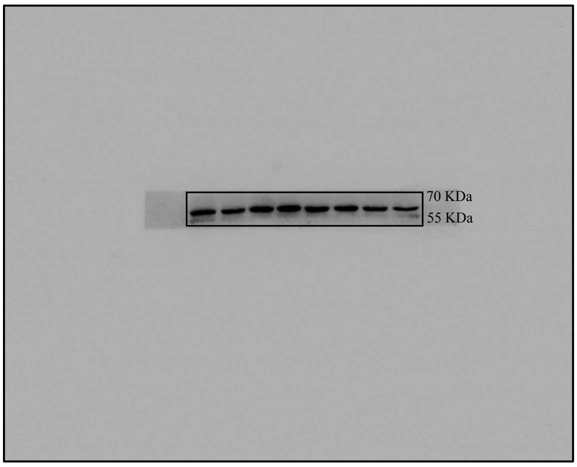

STING

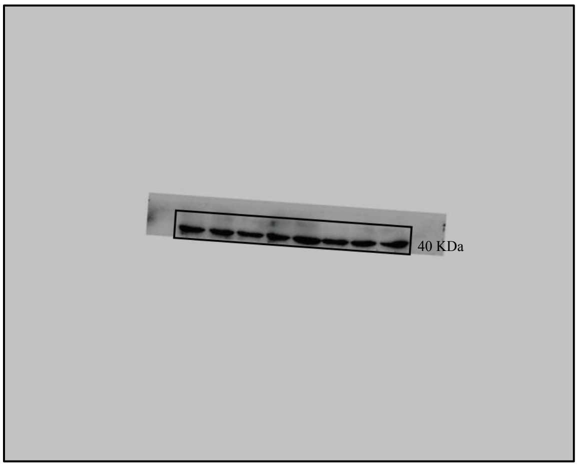

γH2A

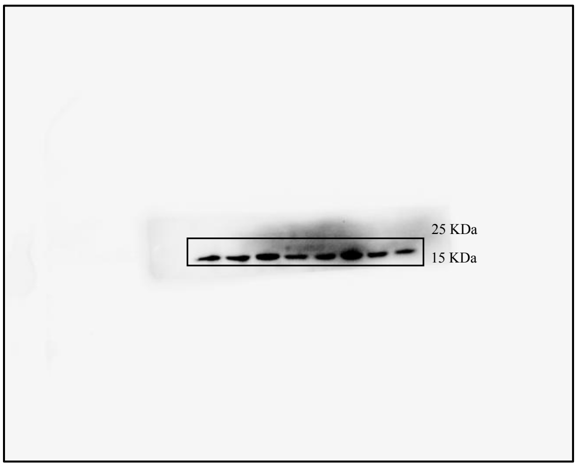

GAPDH

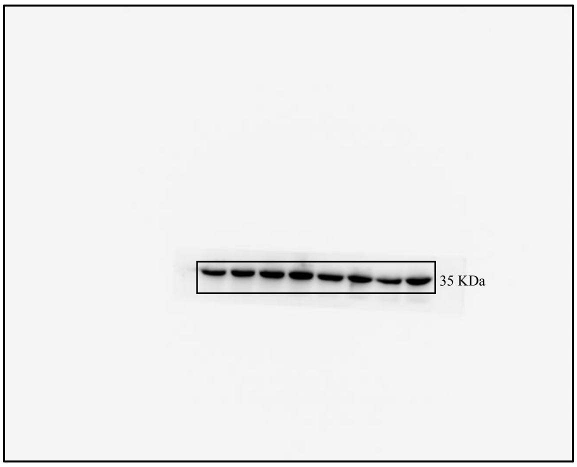

Figure S5A

ATR

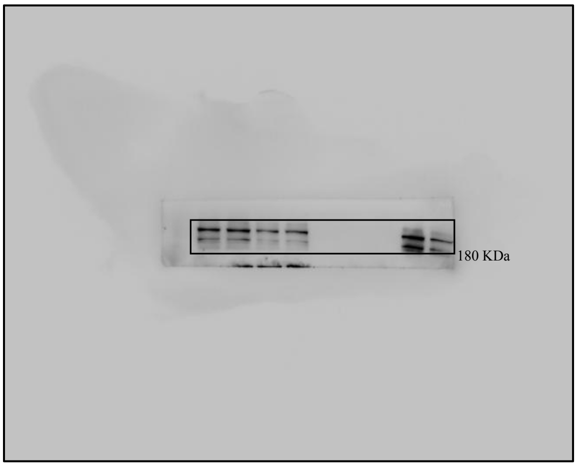

USP10

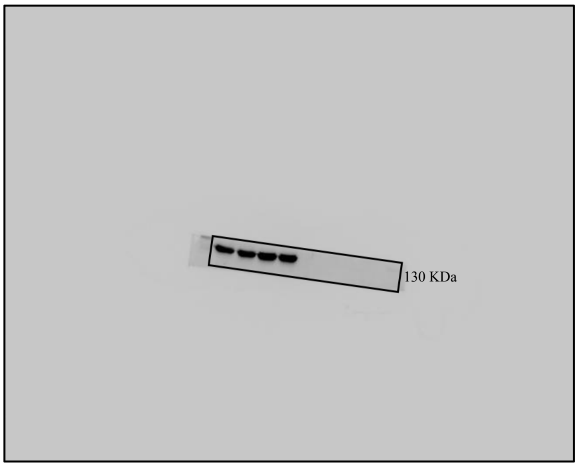

USP5

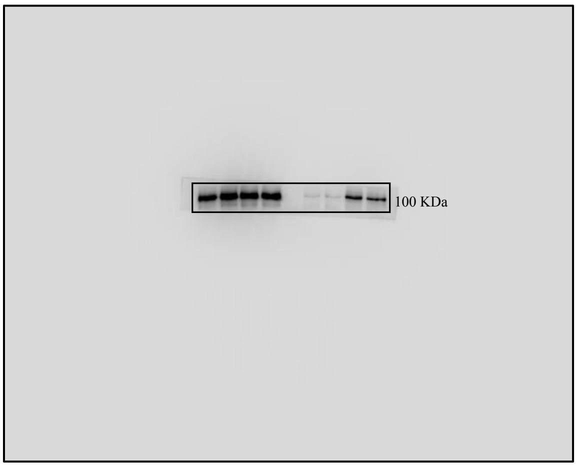

TRIM25

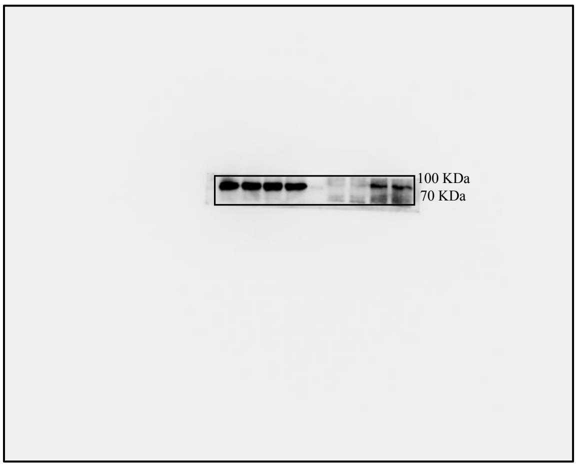

MKRN1

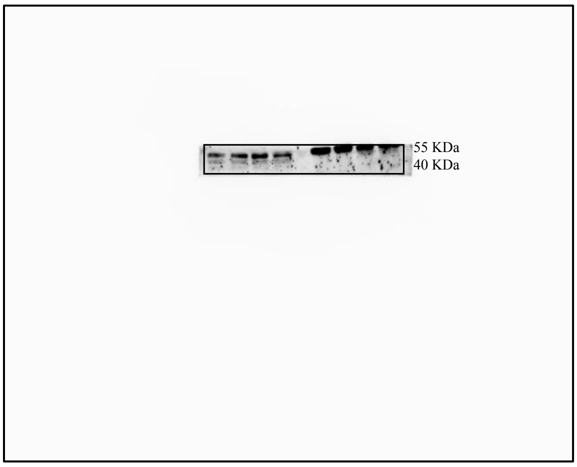

GAPDH

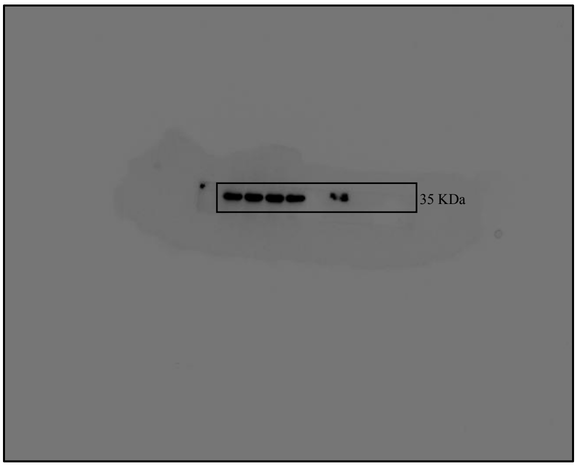

Figure S5B

ATR

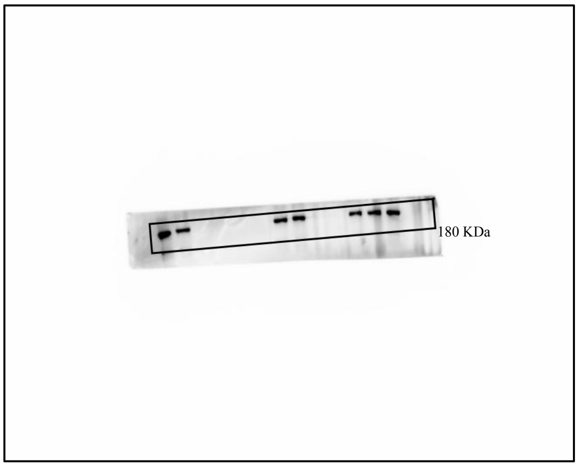

USP10

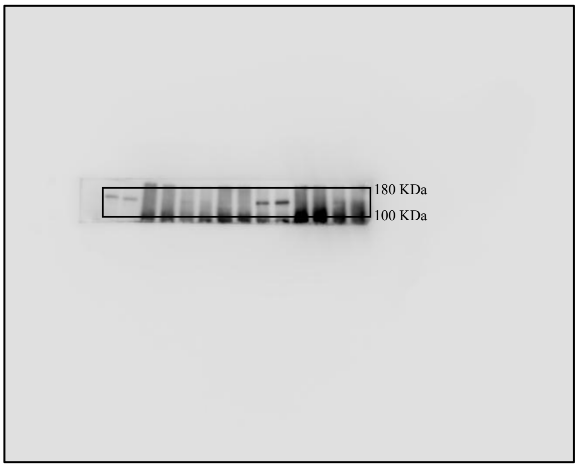

USP5

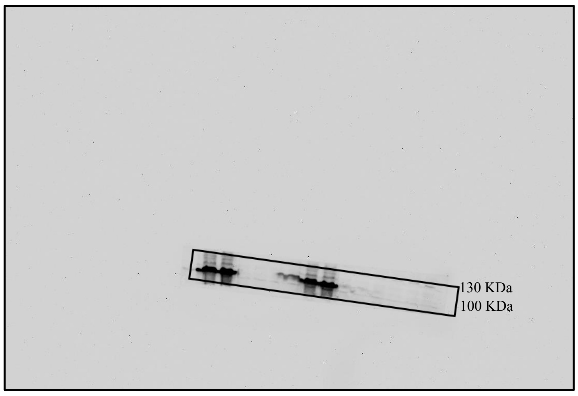

TRIM25

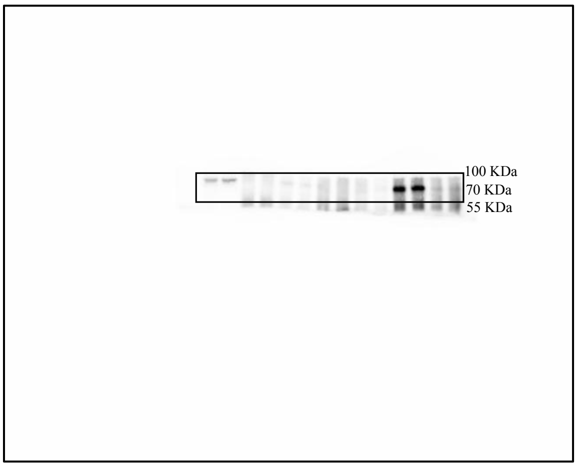

MKRN1

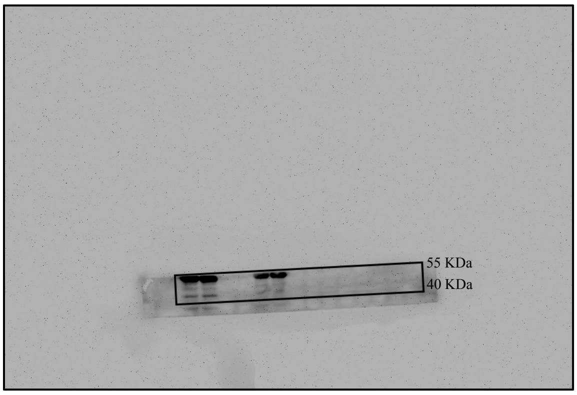

GAPDH

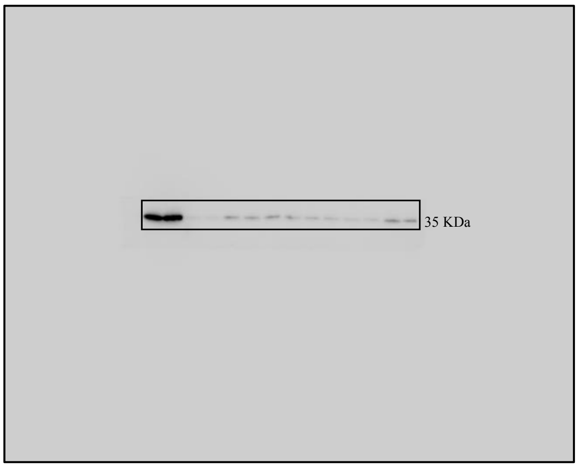

Figure S5C

IP:FLAG

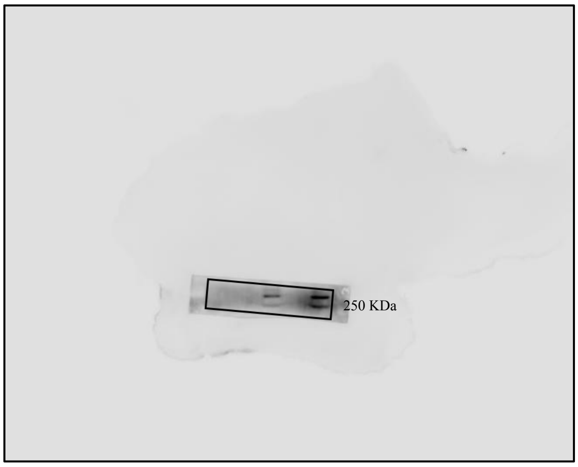

IP:MYC

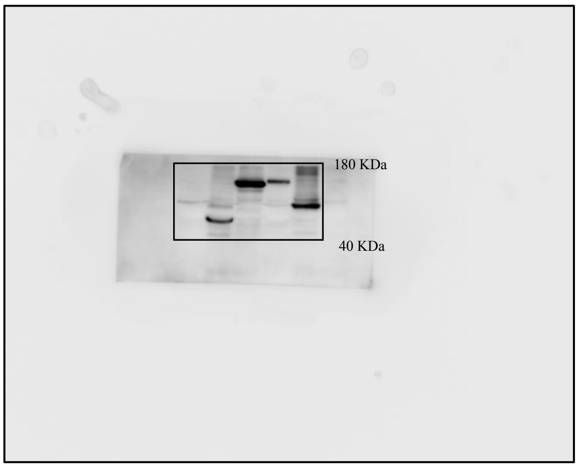

Input: Flag

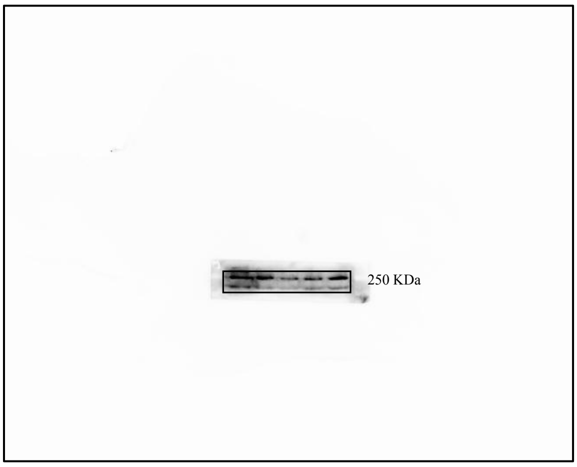

Input: MYC

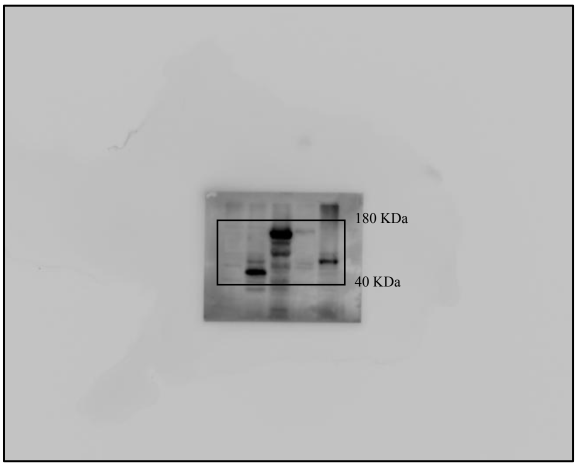

Input: GAPDH

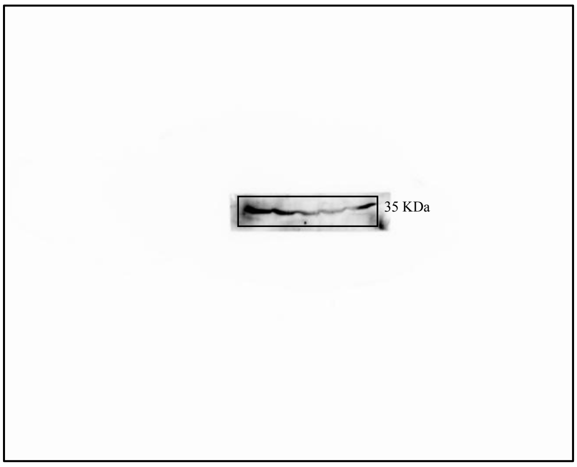

IP: Flag

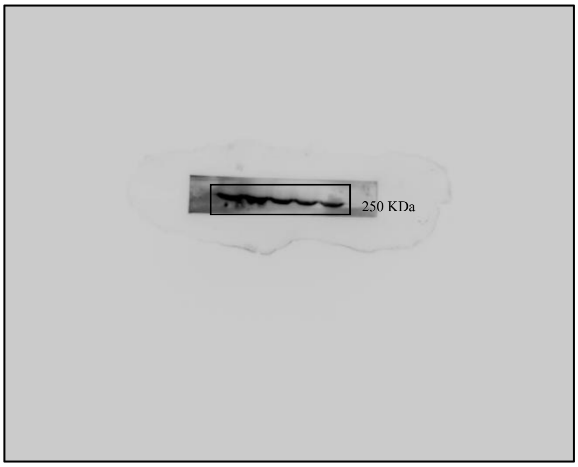

IP: MYC

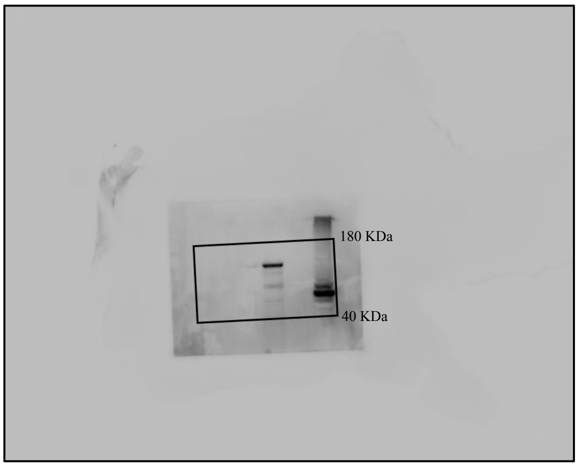

Input: Flag

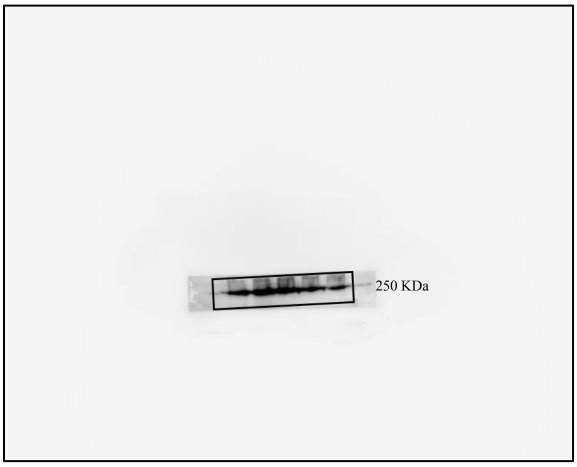

Input: MYC

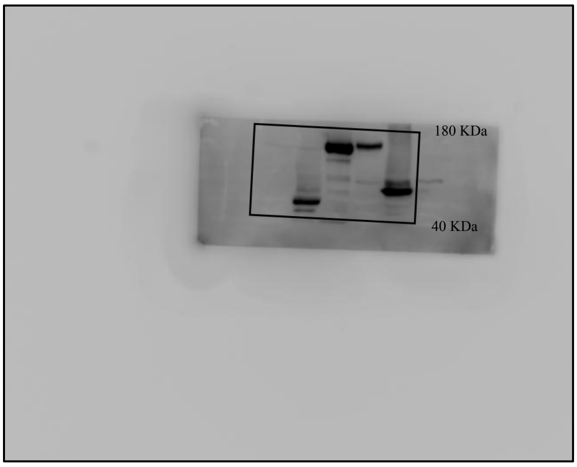

Input: GAPDH

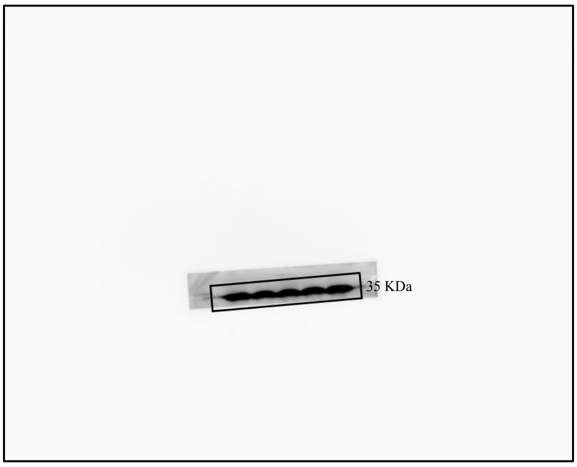
35 KDa

Figure S5D

TRIM56

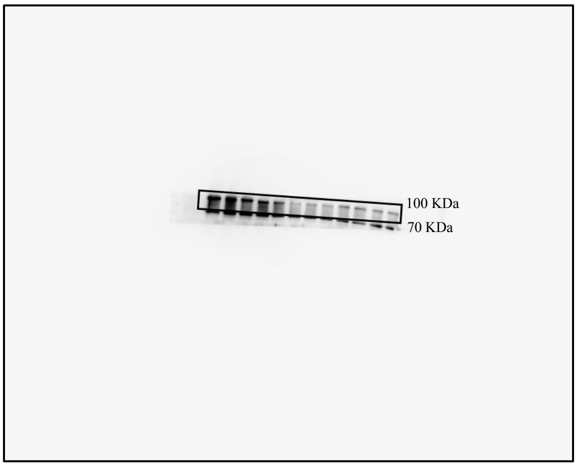

GAPDH

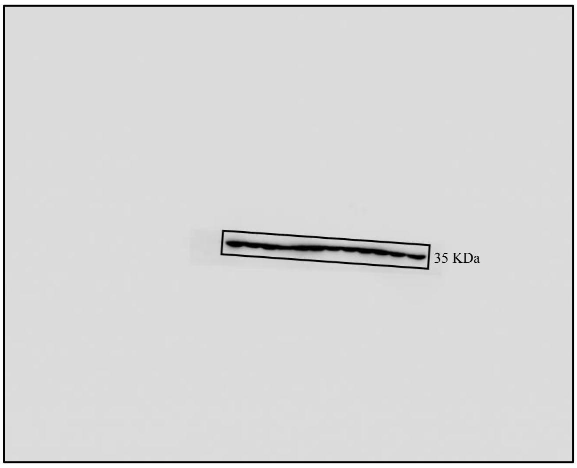

USP5

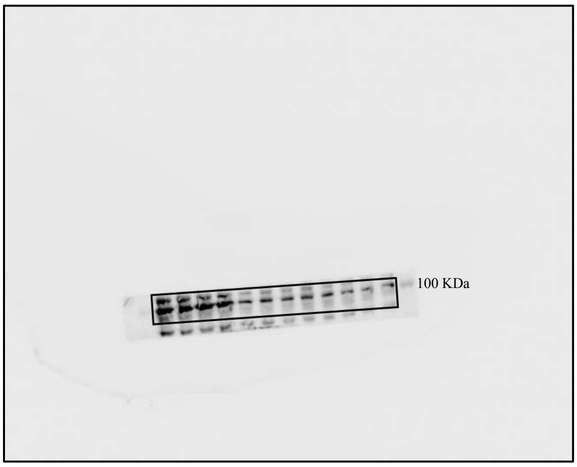

GAPDH

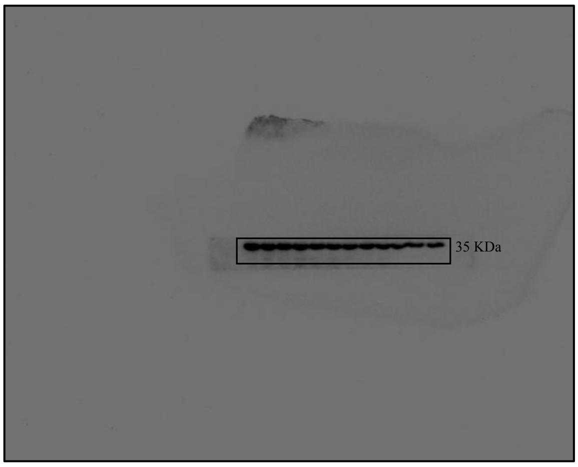

TRIM25

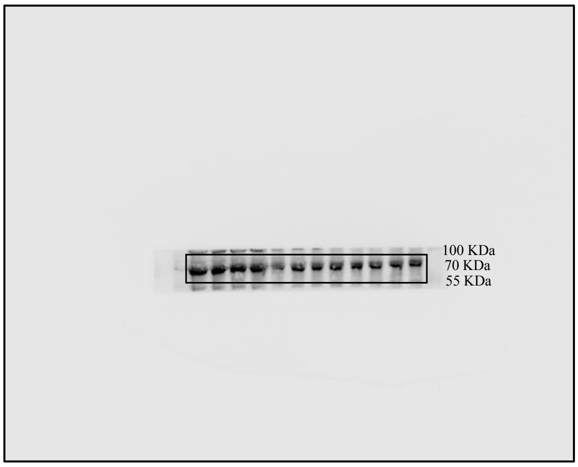

GAPDH

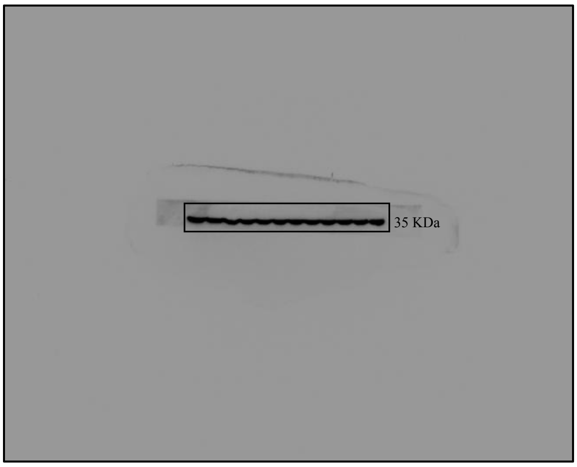

Figure S5E

ATR

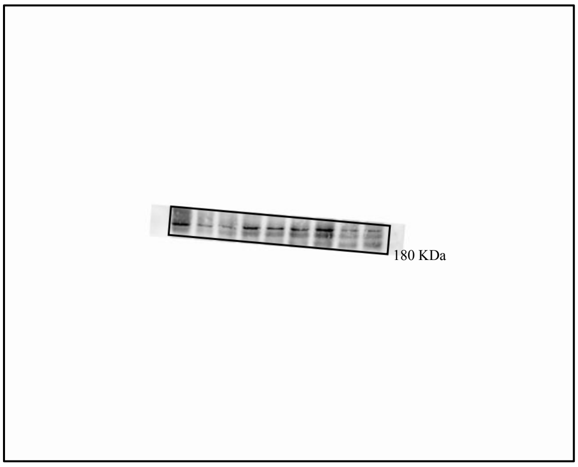

GAPDH

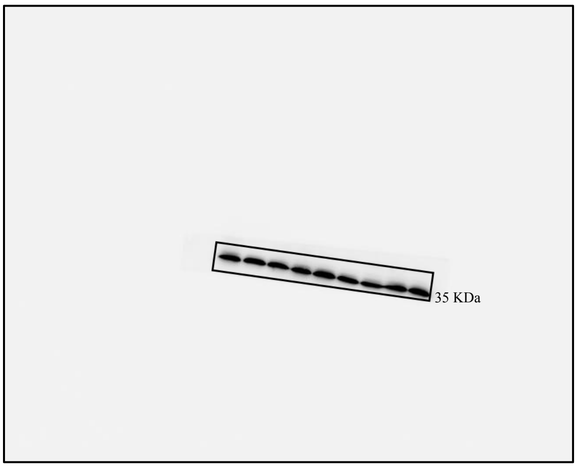

Figure S5F

ATR

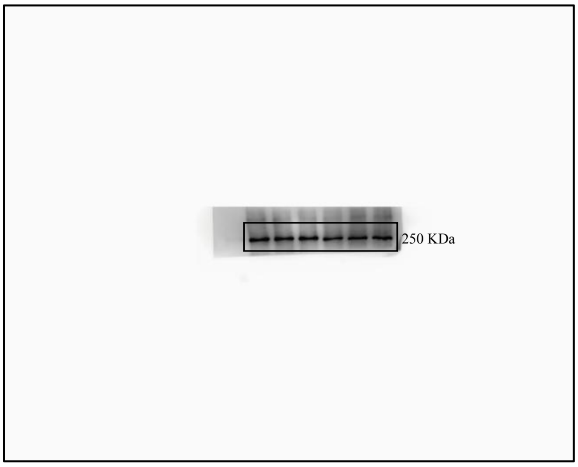

GAPDH

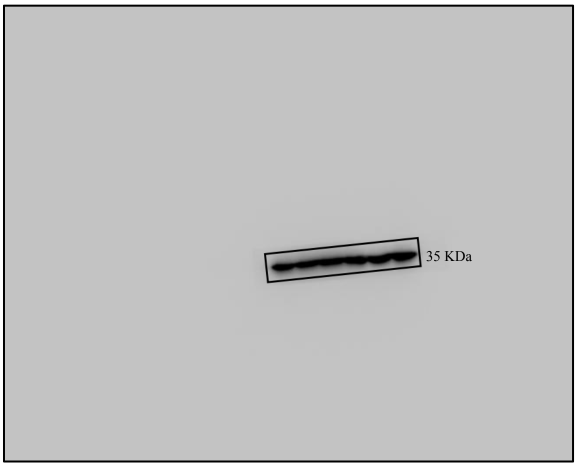

ATR

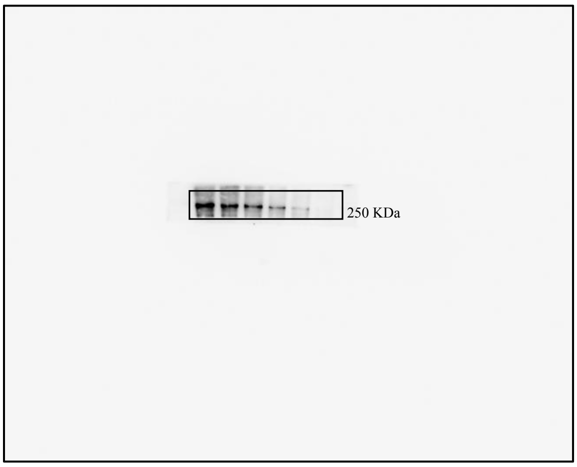

GAPDH

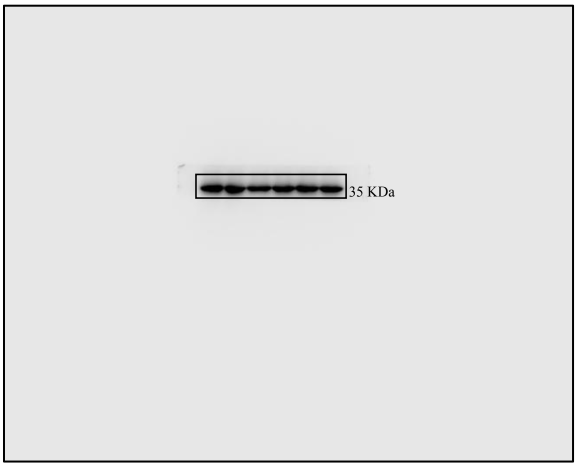

ATR

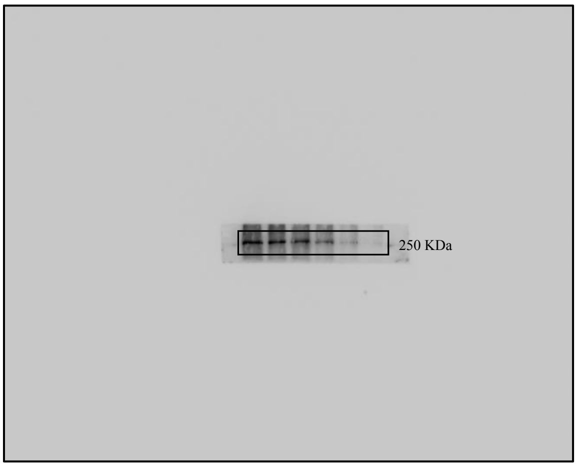

GAPDH

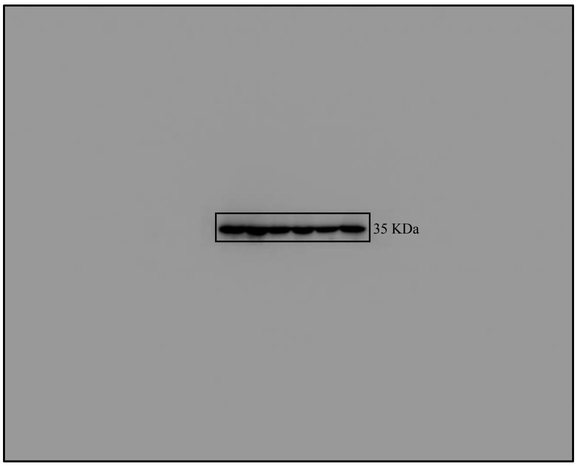

Figure S5H

IB: K63-Ub

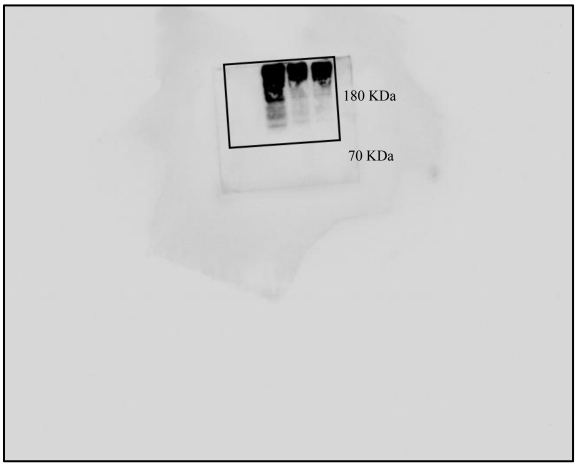

IB: ATR

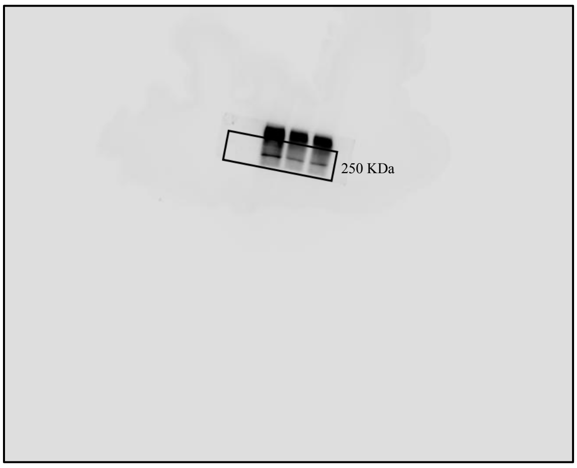

Input: ATR

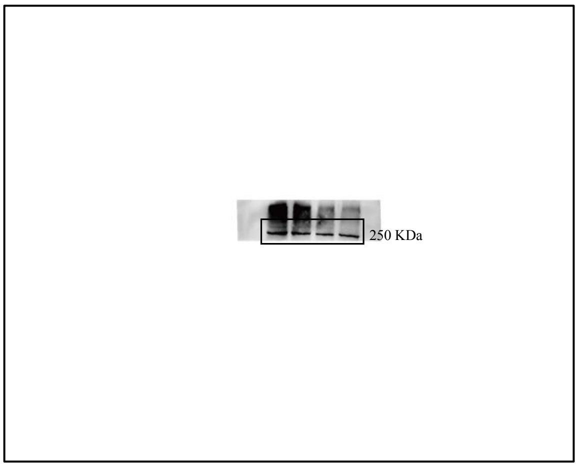

Input: GAPDH

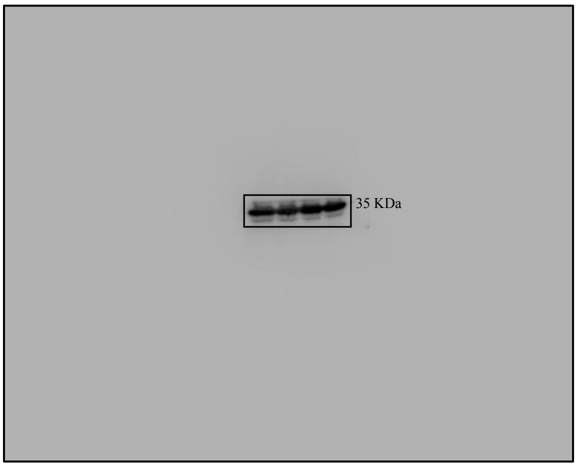

Input: K63-Ub

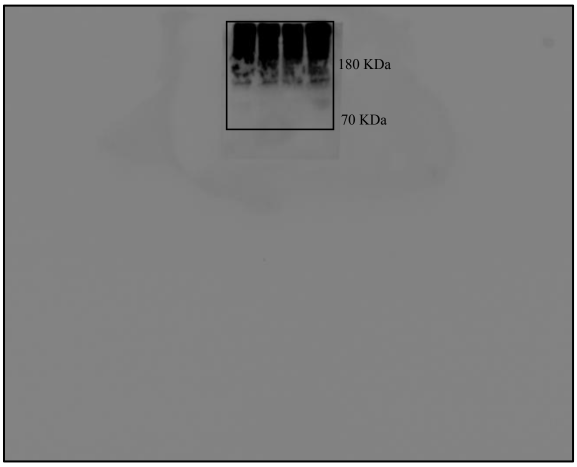

IB: K48-Ub

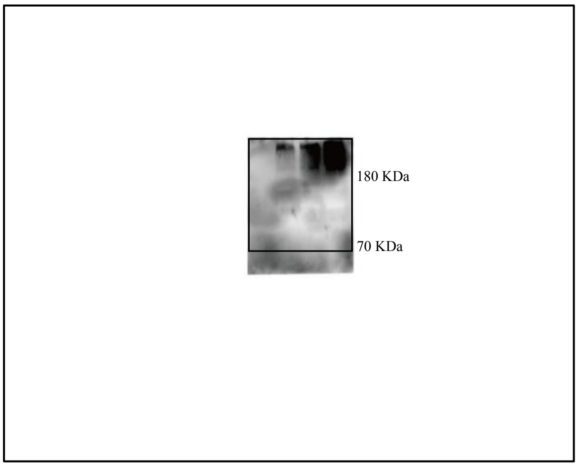

IB: ATR

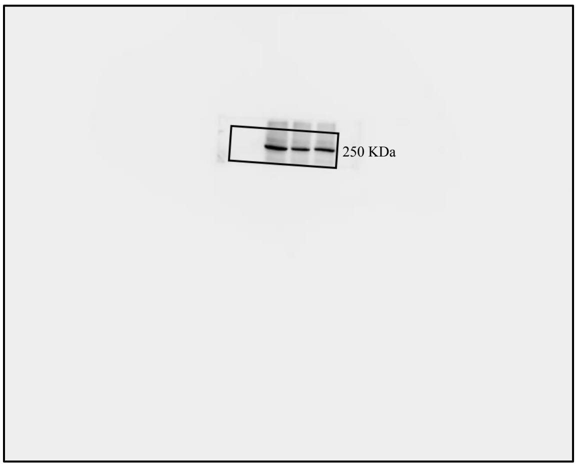

Input: ATR

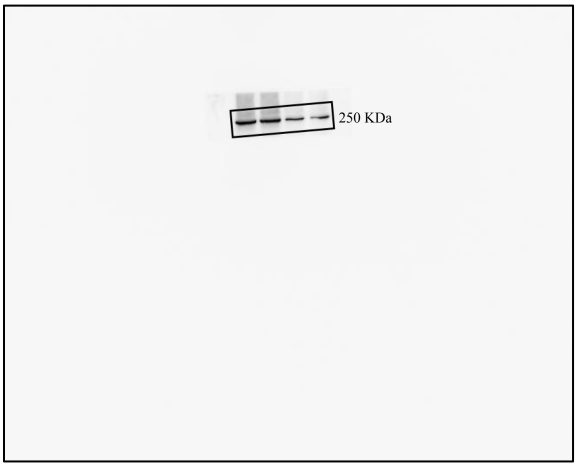

Input: GAPDH

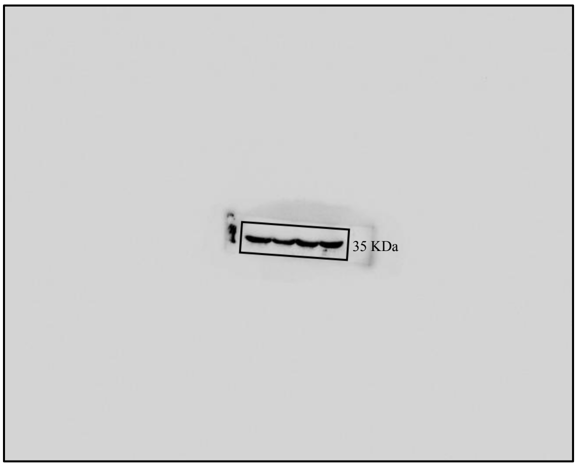

Input: K48-Ub

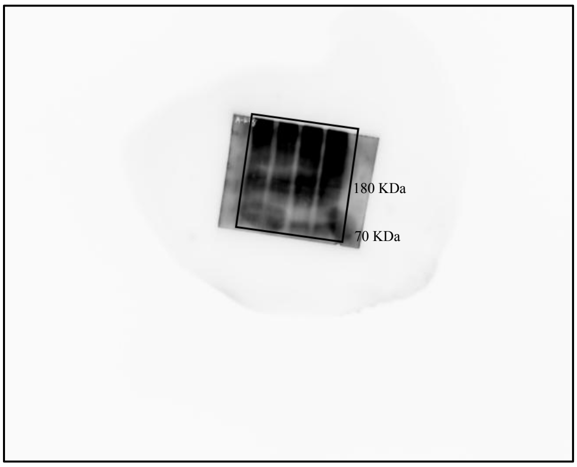
180 KDa
70 KDa

Figure S5I

IB: K63-Ub

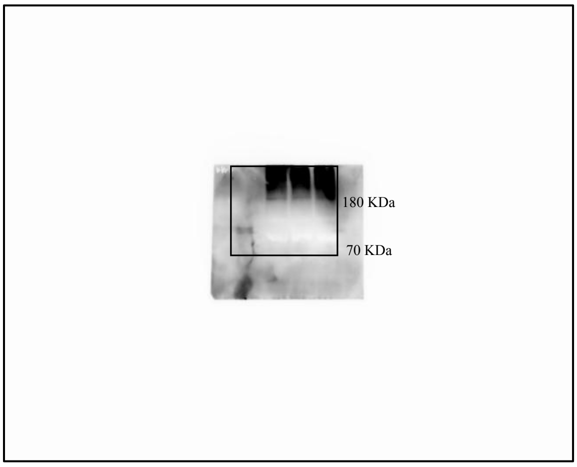

IB: STING

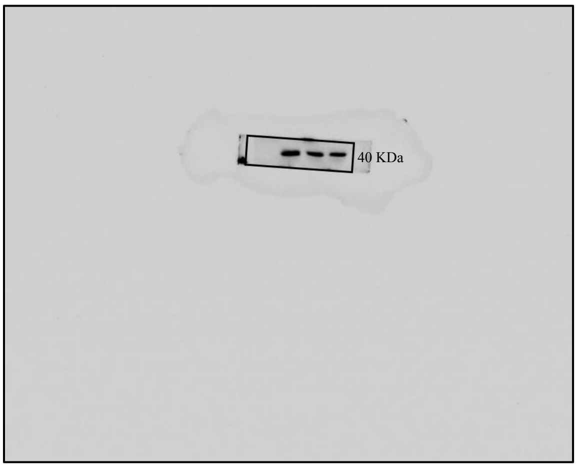

Input: K63-Ub

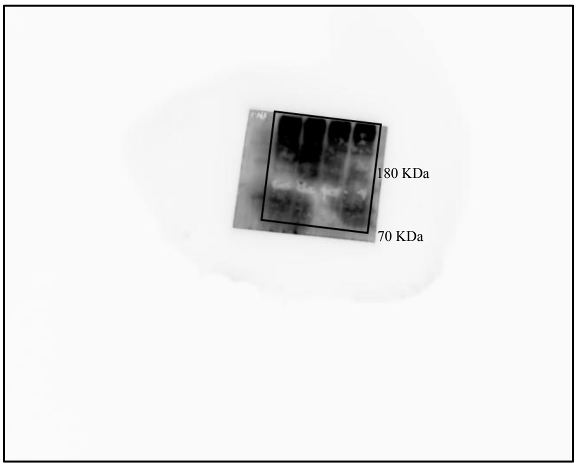

Input: STING

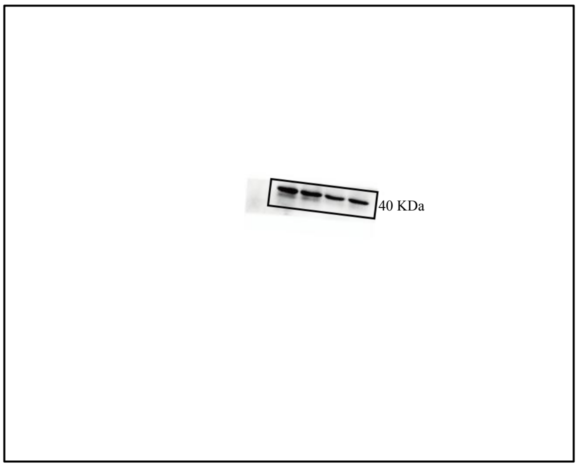

Input: GAPDH

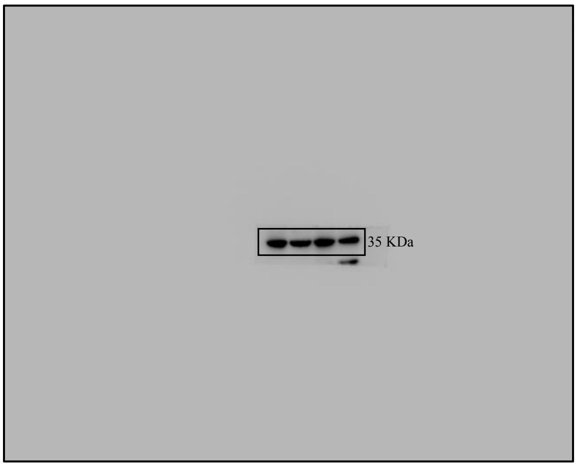

IB: Ub

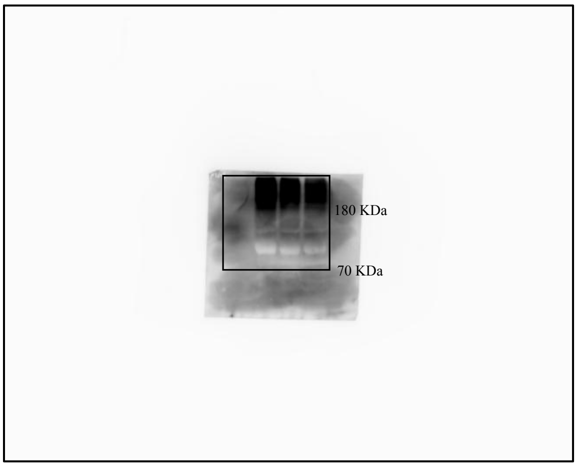

IB: STING

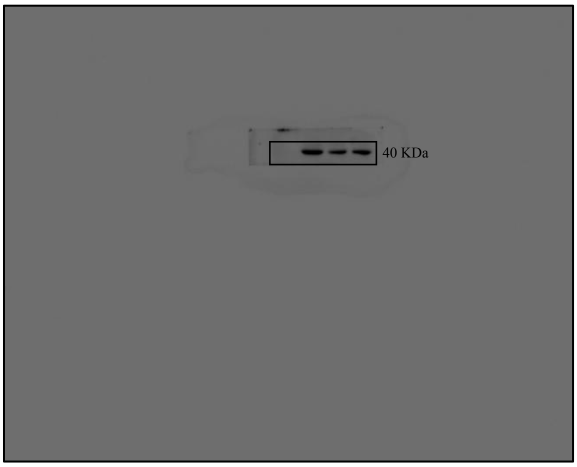

Input: Ub

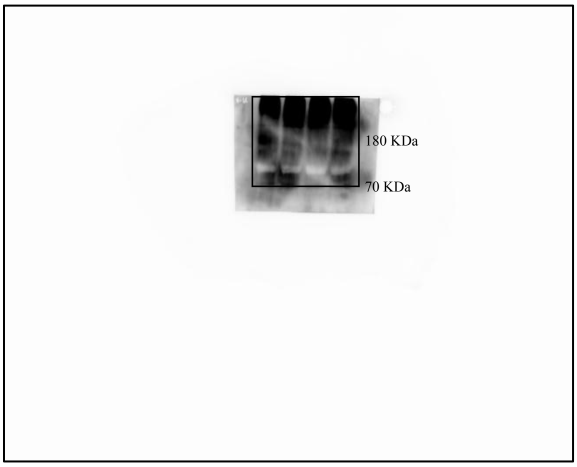

Input: STING

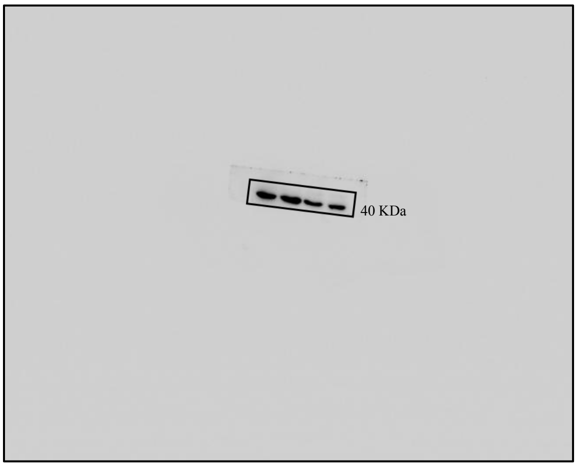

Input: GAPDH

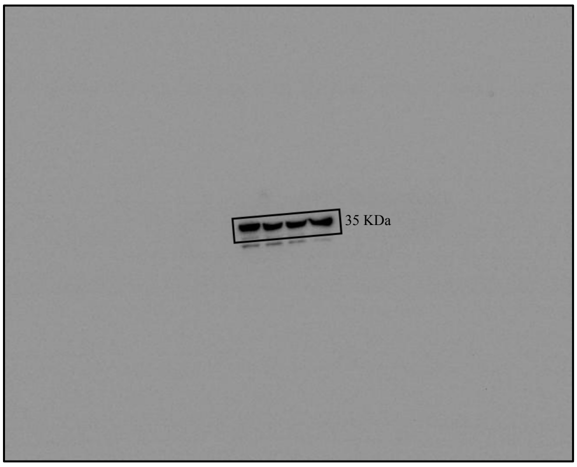
35 KDa

Figure S5J

ATR

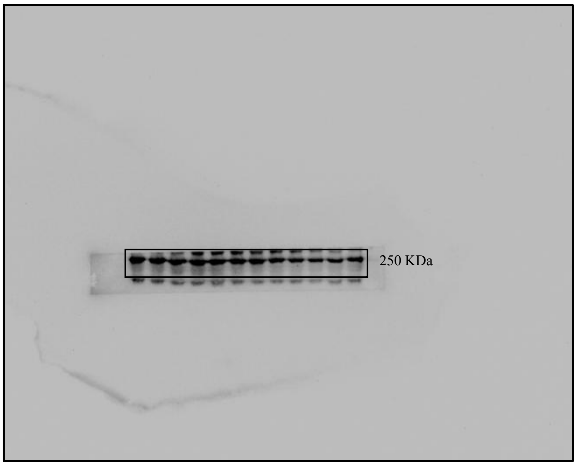

GAPDH

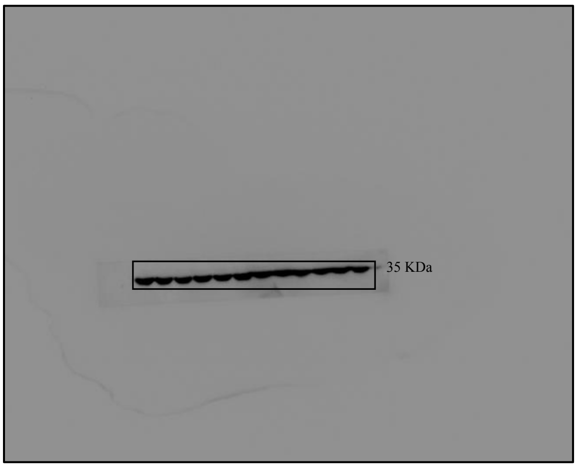

Figure S5K

ATR

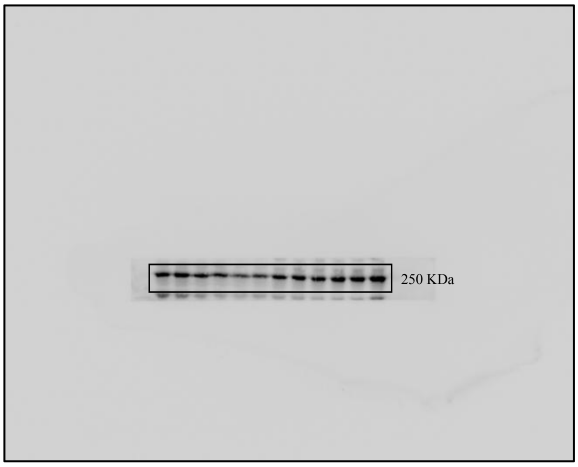

GAPDH

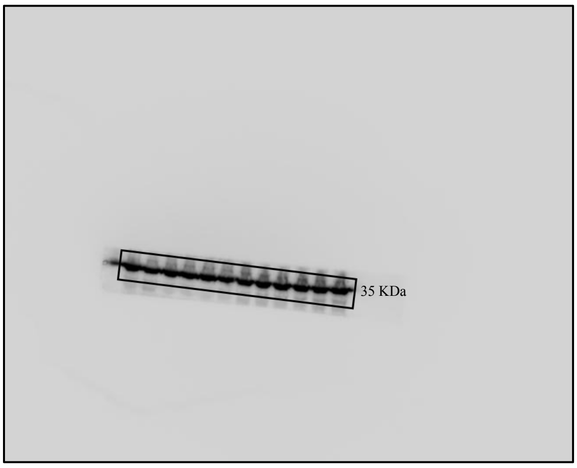

Figure S5L

ATR

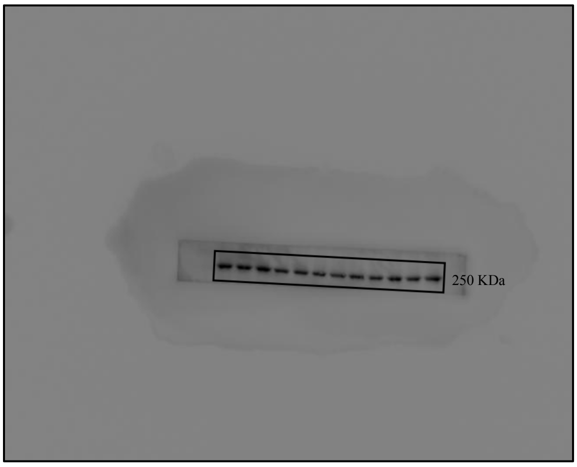

GAPDH

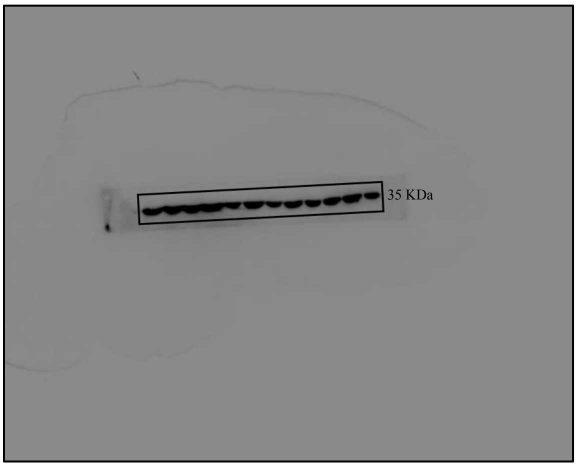

Figure S5M

ATR

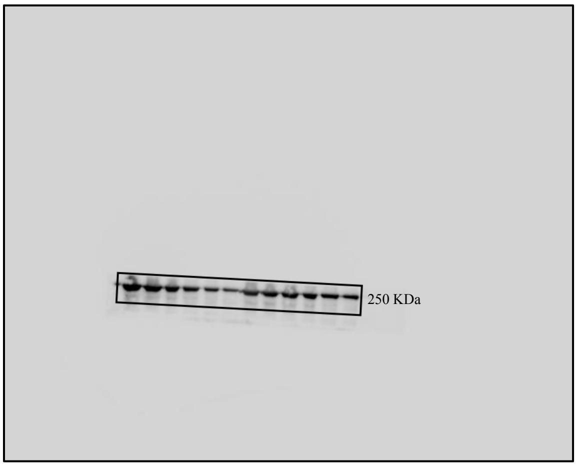

GAPDH

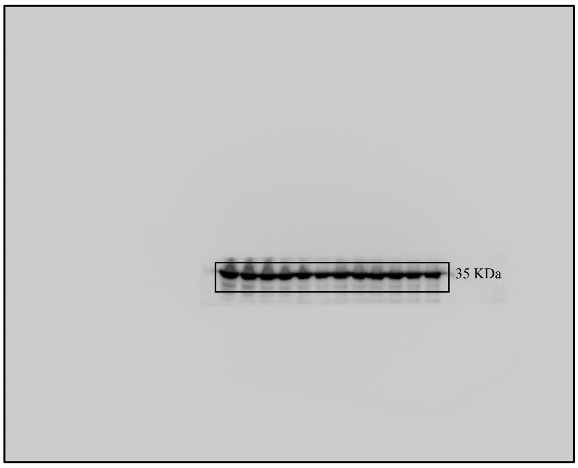

Figure S5P

ATR

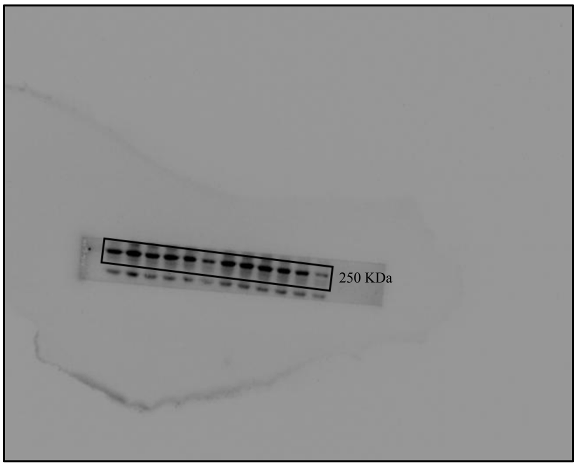

GAPDH

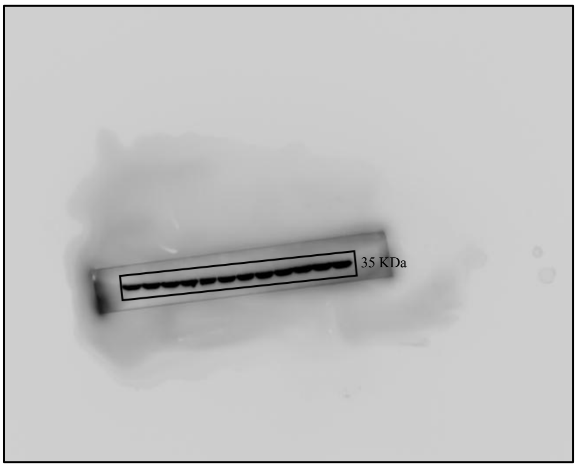

Figure S5Q

ATR

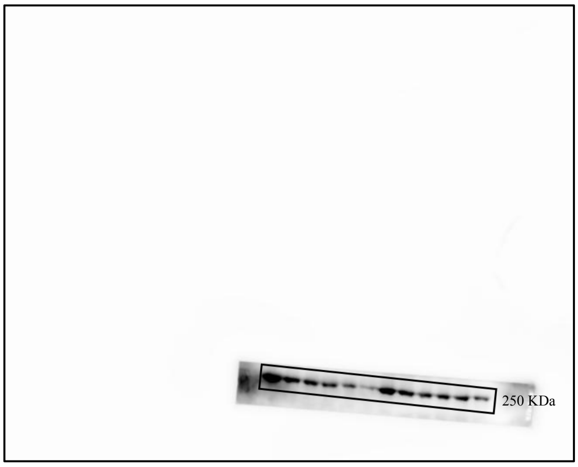

GAPDH

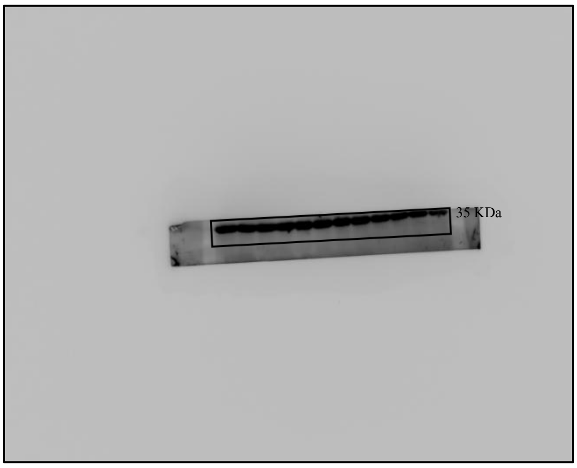

Figure S5R

ATR

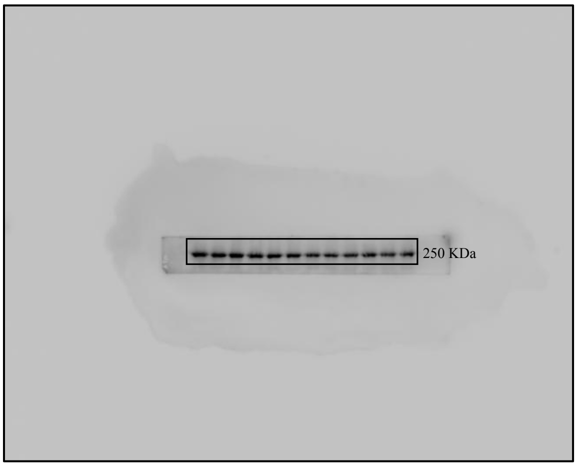

GAPDH

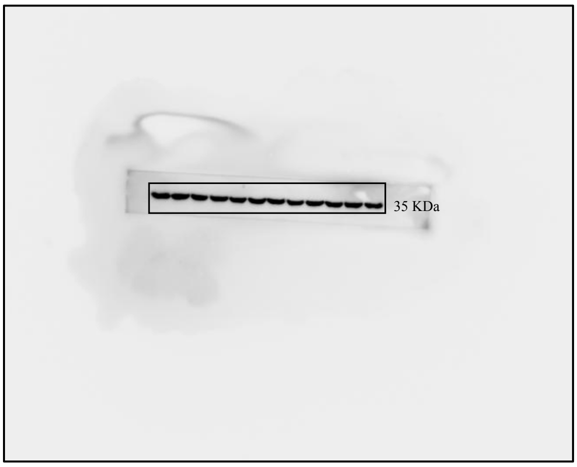

Figure S5S

ATR

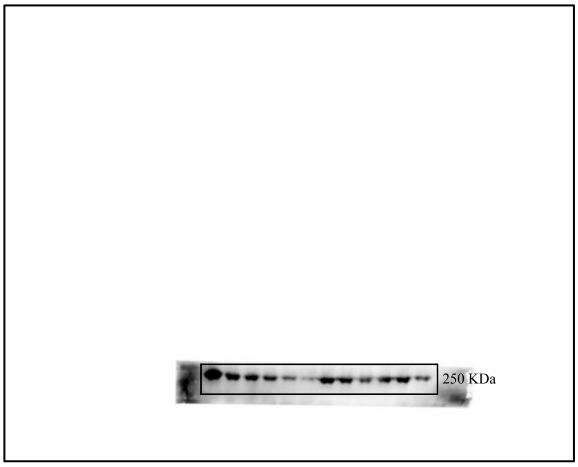

GADPH

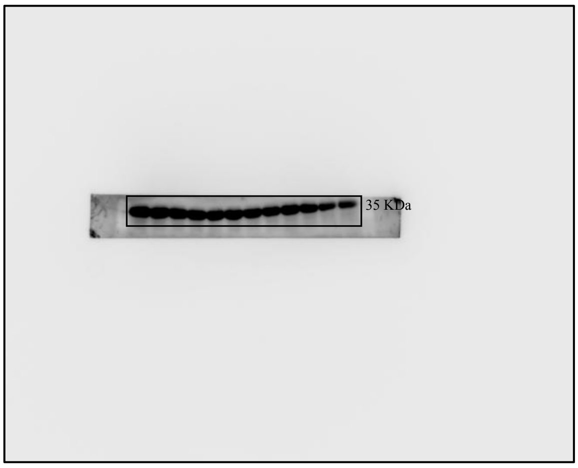

Figure S5V

K63-Ub

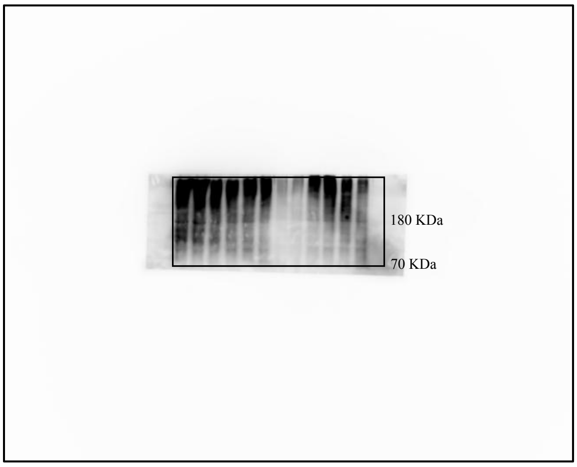

K48-Ub

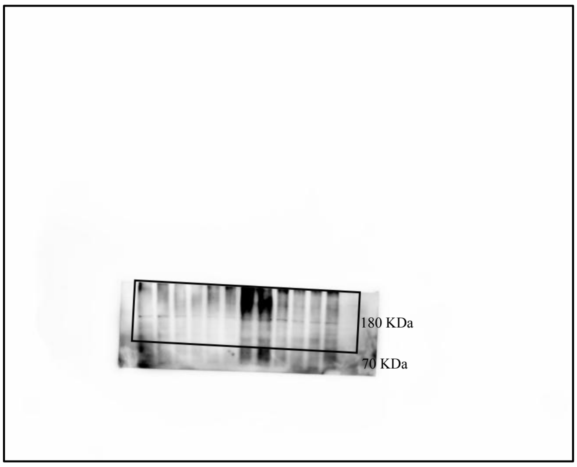

IP: ATR

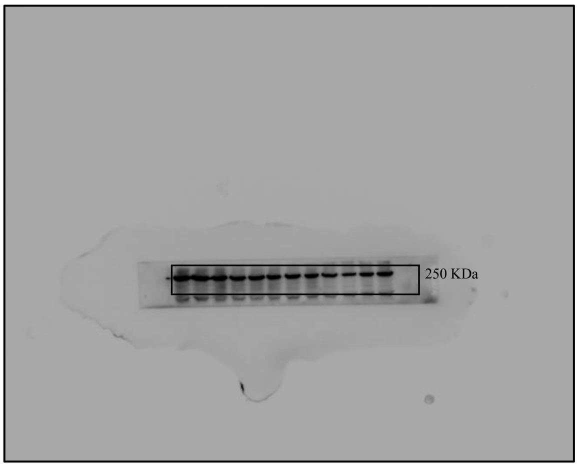

Input: ATR

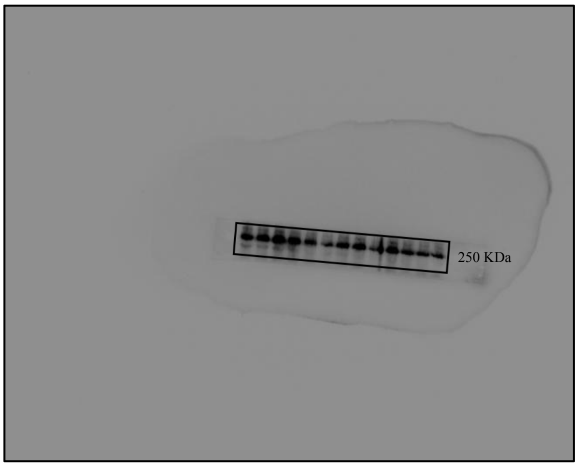

Input: GAPDH

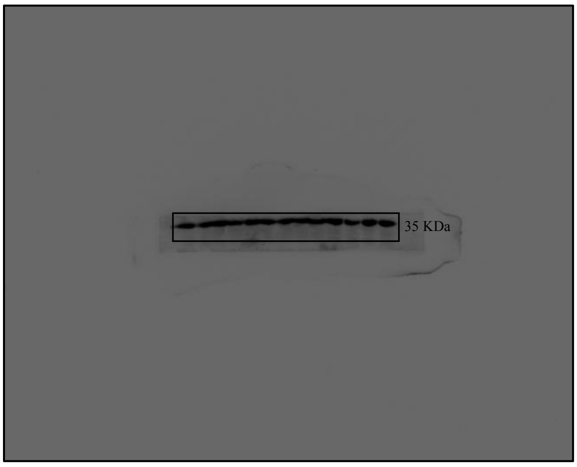

Figure S5W

K63-Ub

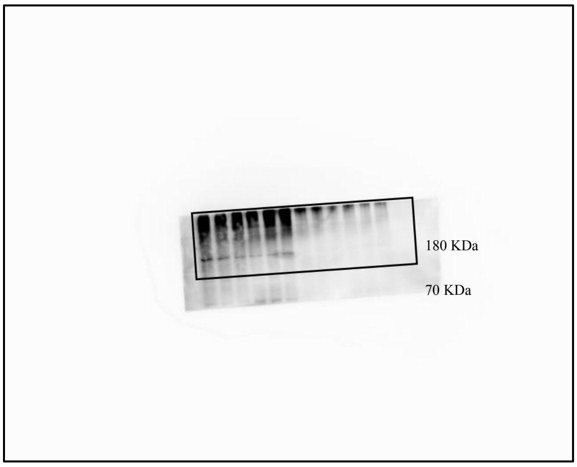

K48-Ub

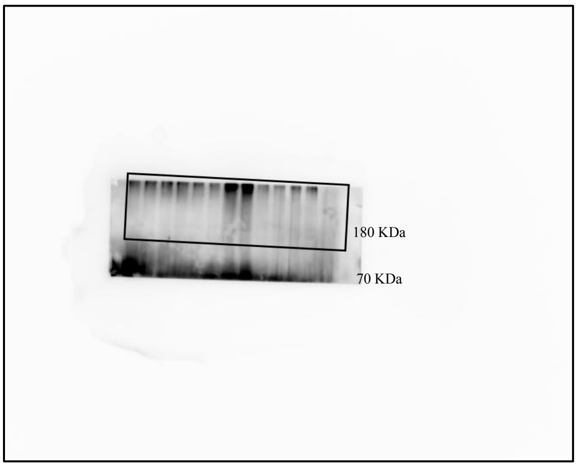

IP: ATR

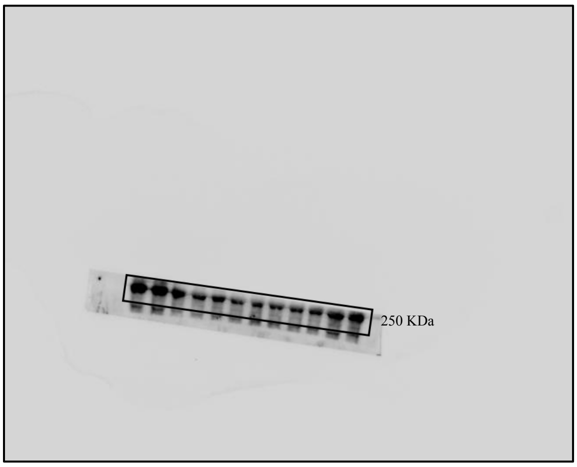

Input:ATR

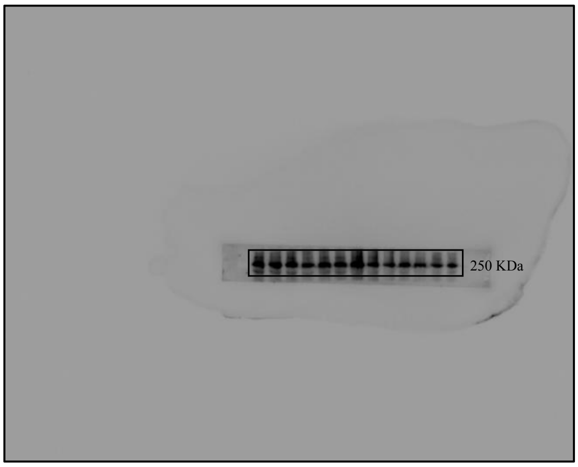

Input: GAPDH

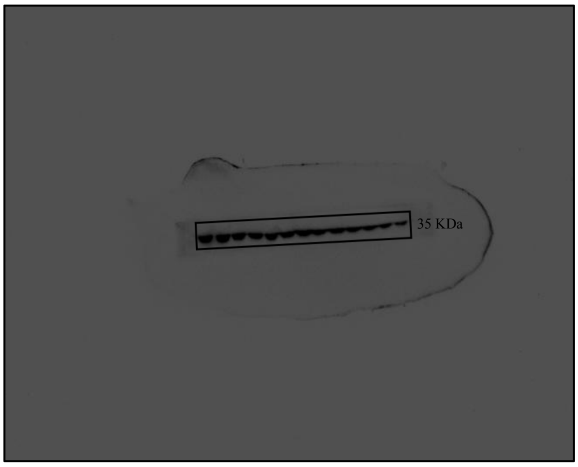

Figure S5X

K63-Ub

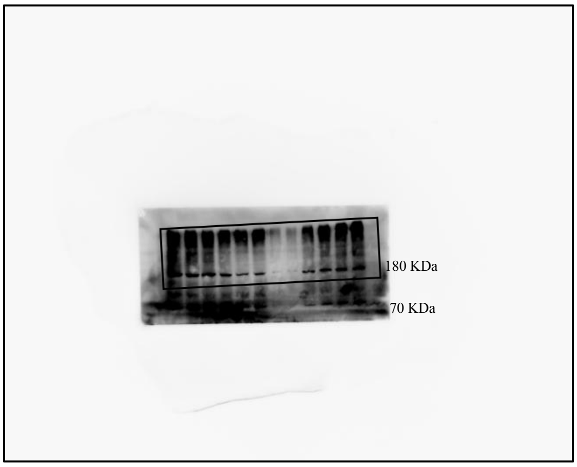

K48-Ub

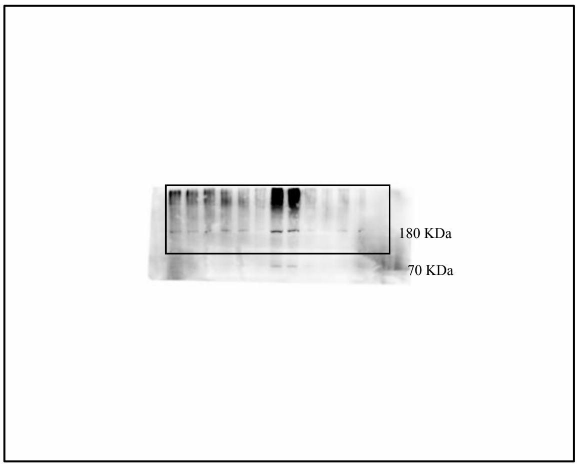

IP: ATR

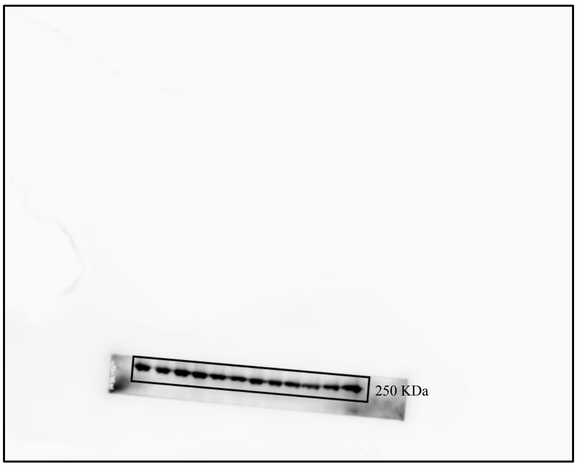

Input: ATR

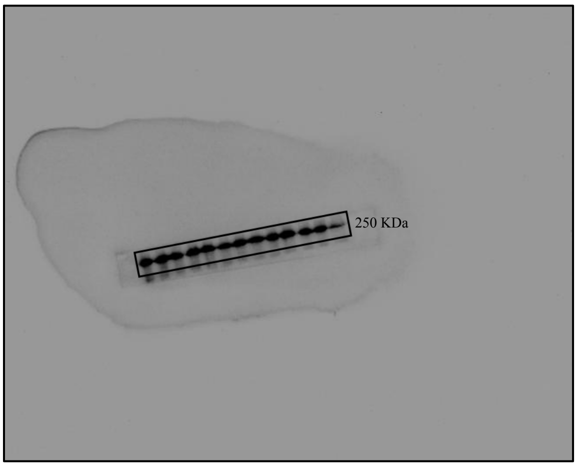

Input: GAPDH

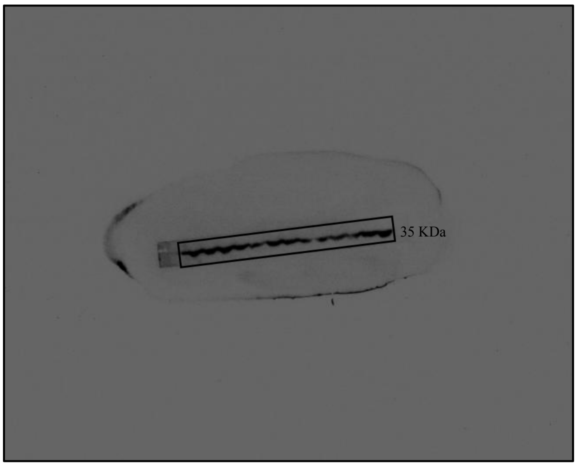

Figure S5Y

K63-Ub

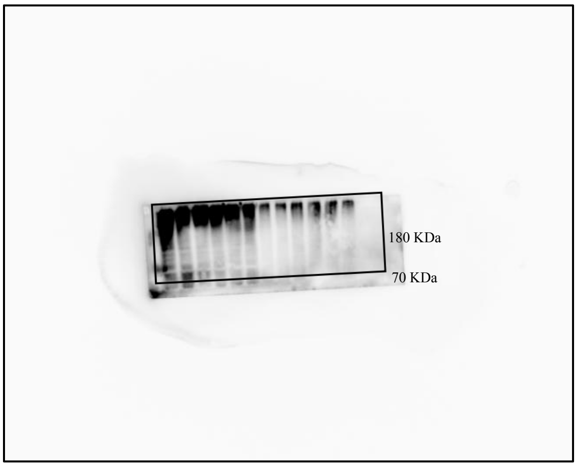

K48-Ub

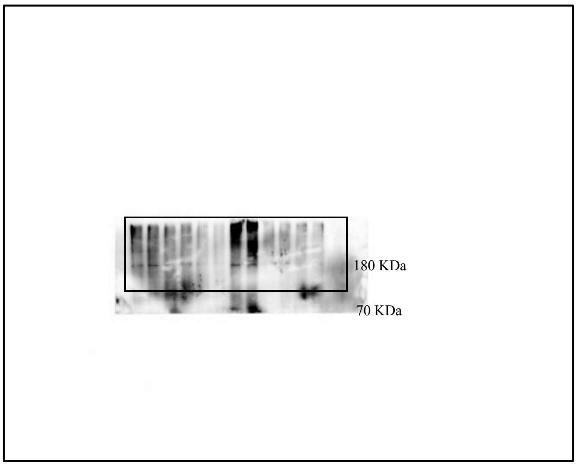

IP: ATR

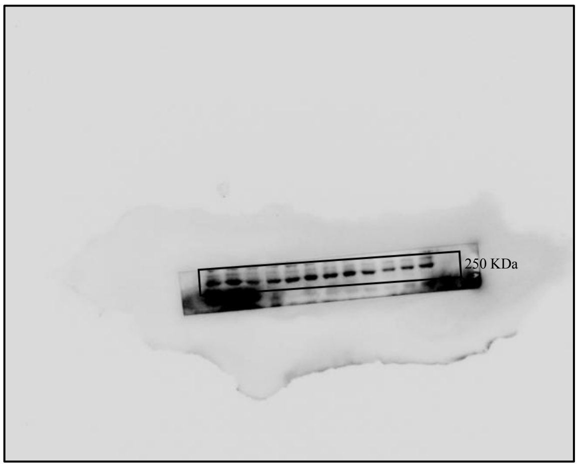

Input: ATR

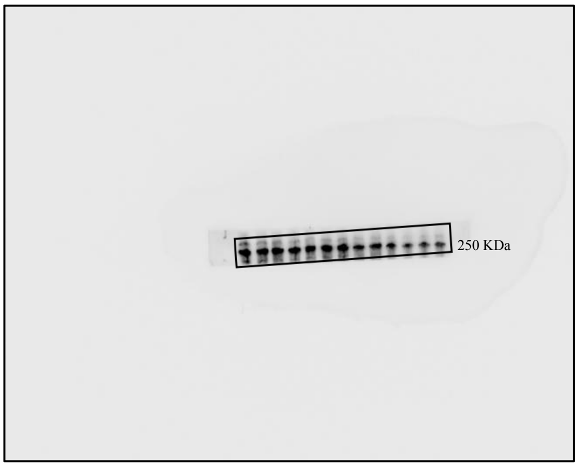

Input: GAPDH

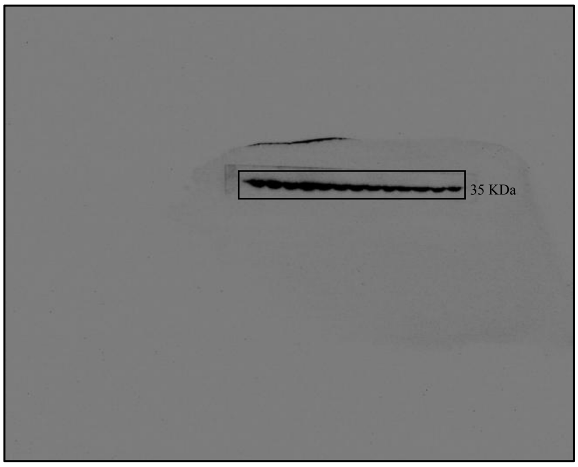

Figure S6A

ATR

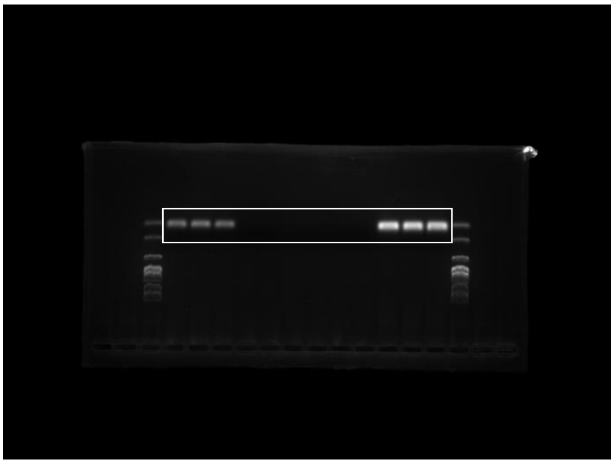

TSG101

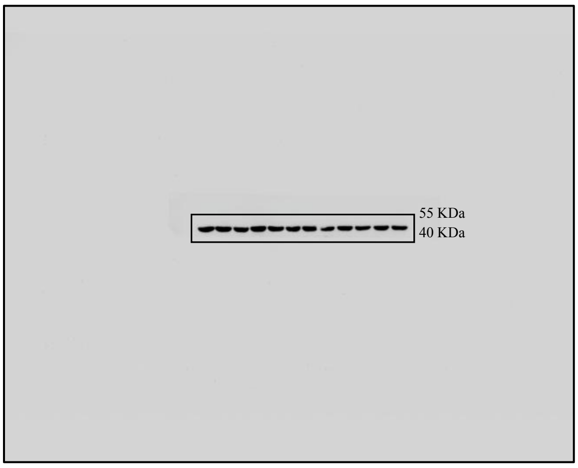

CD63

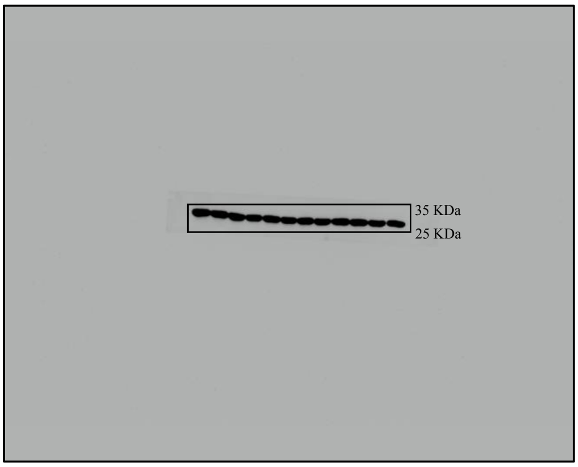

CD9

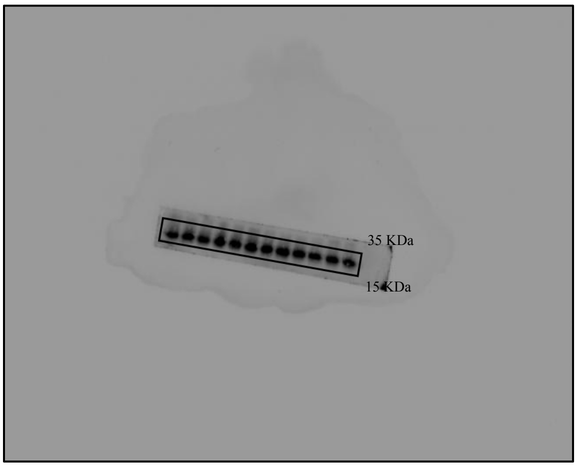

GAPDH

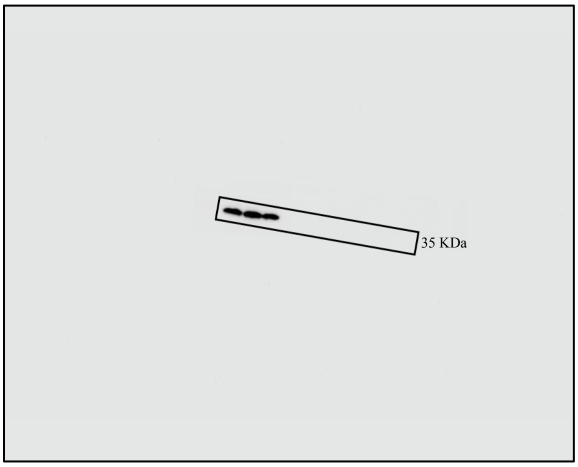

ATR

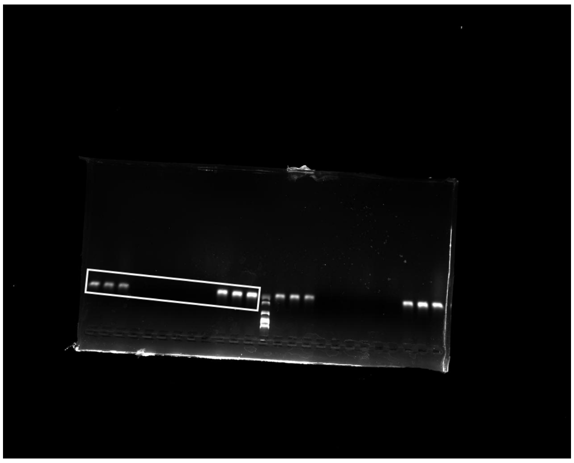

TSG

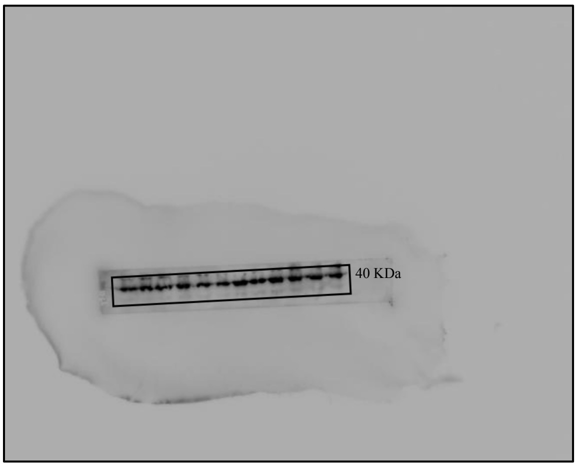

CD63

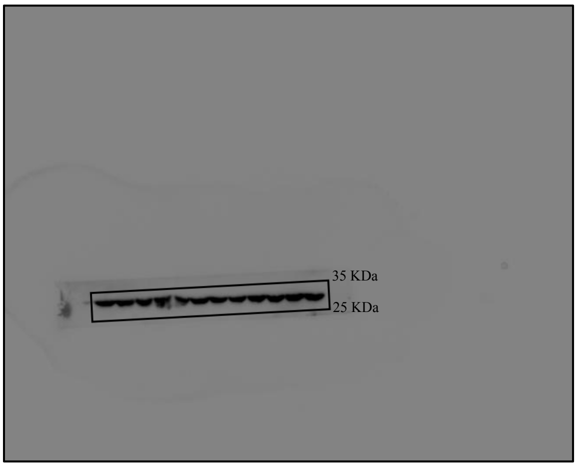

CD9

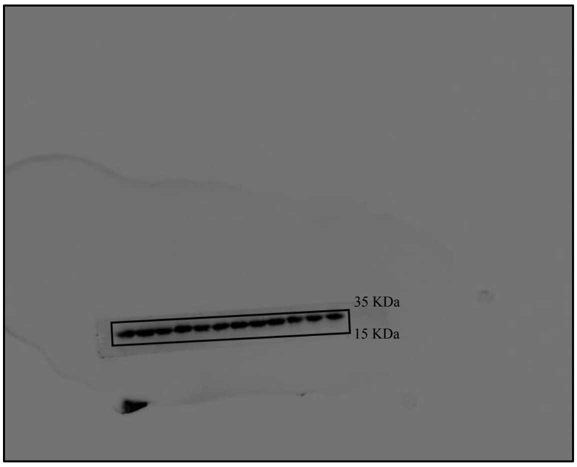

GAPDH

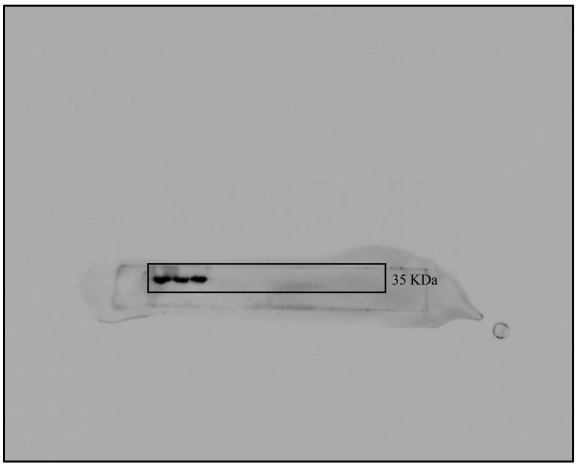
35 KDa

Figure S6E

p-p53

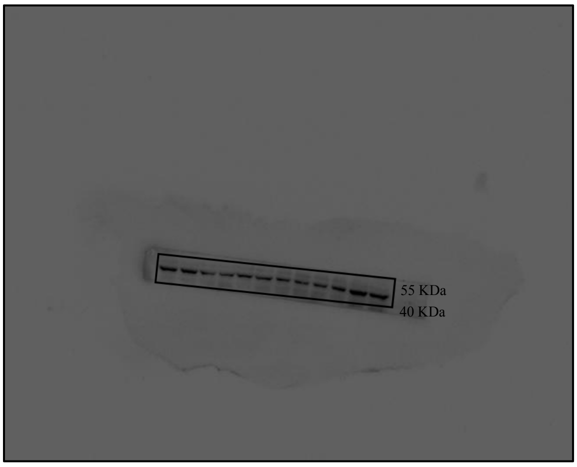

p21

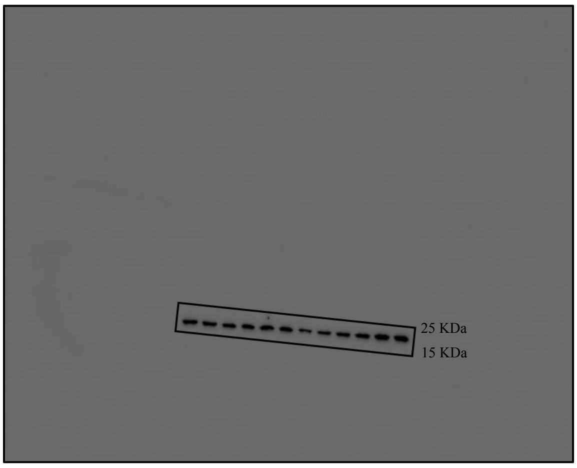

p16

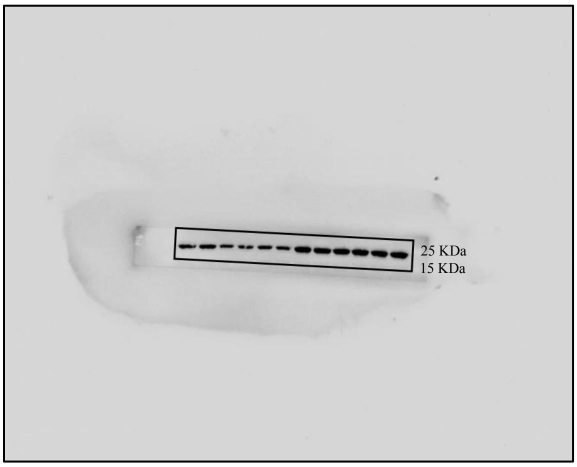

GAPDH

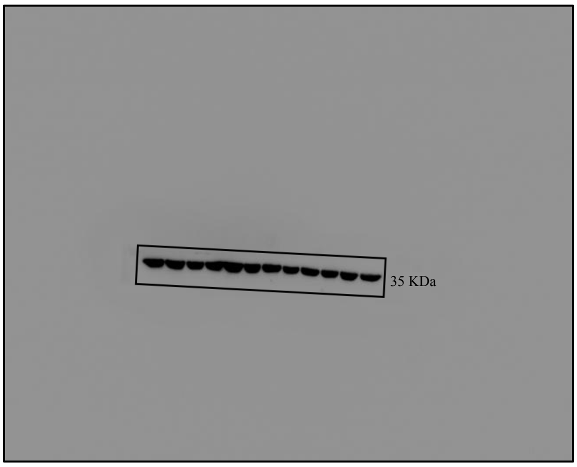
35 KDa

Figure S6F

cGAS

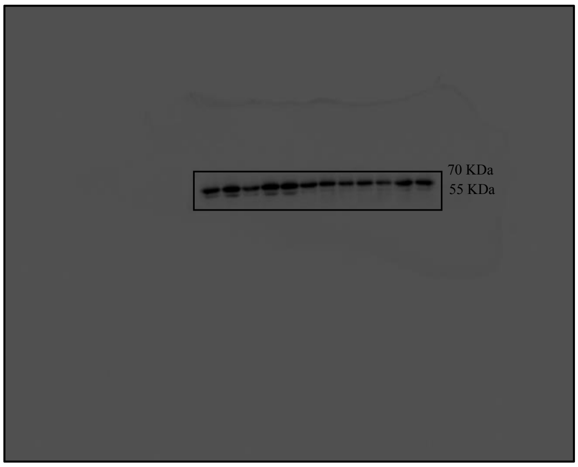

p-STING

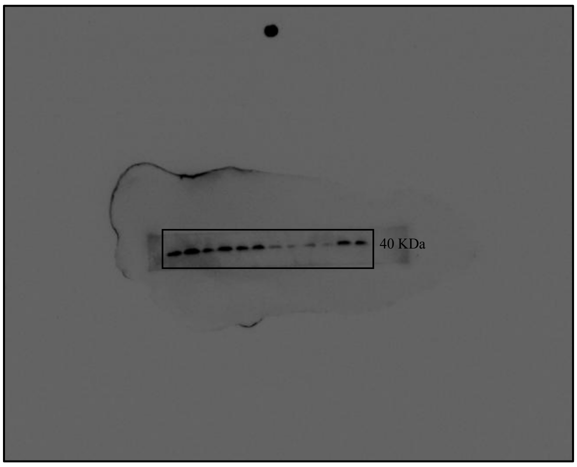

STING

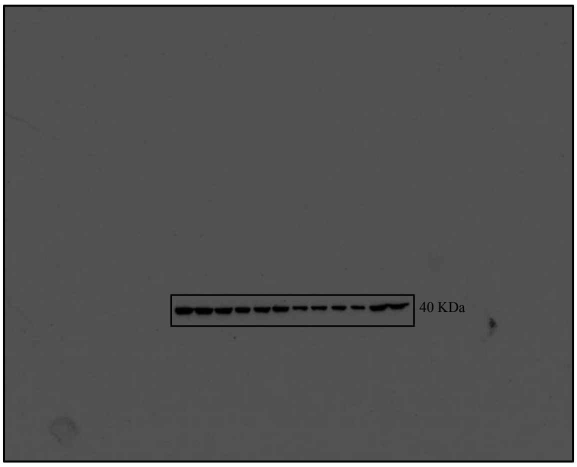

γH2A

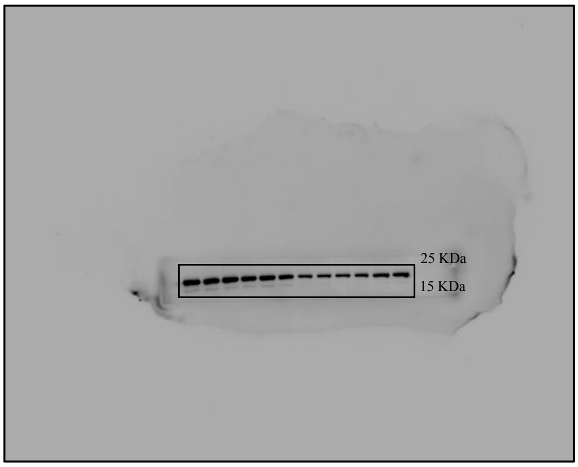

GAPDH

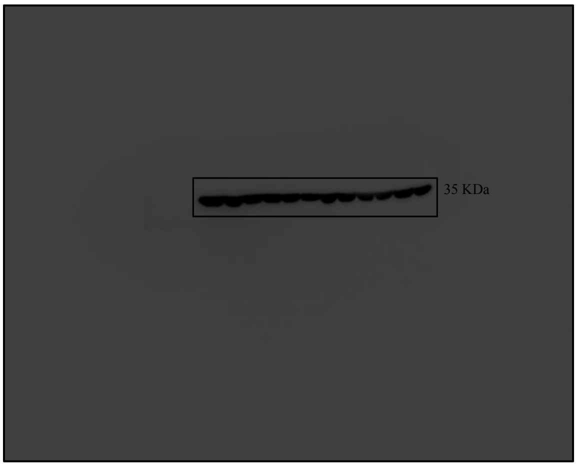

Figure S6H

p-p53

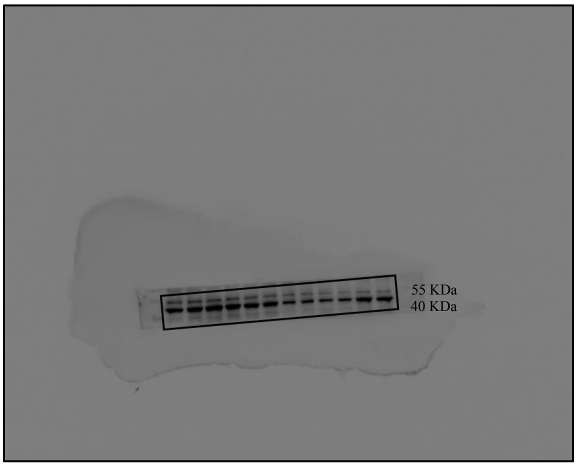

p21

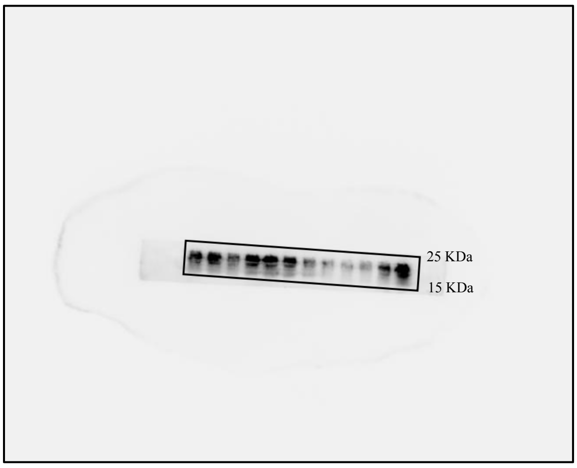

p16

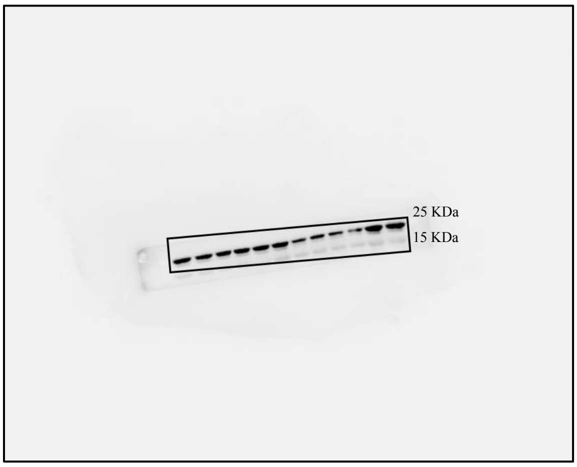

GAPDH

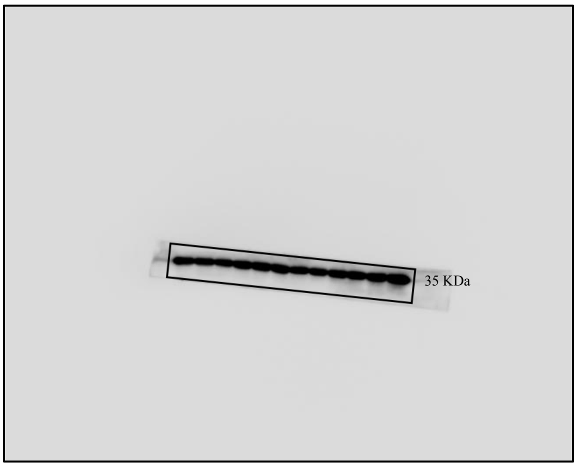
35 KDa

Figure S6I

cGAS

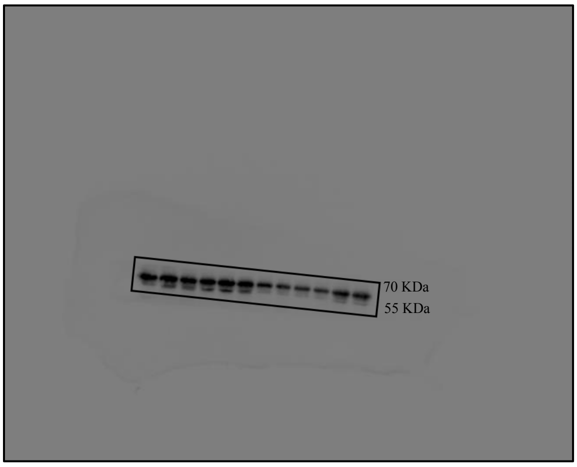

p-STING

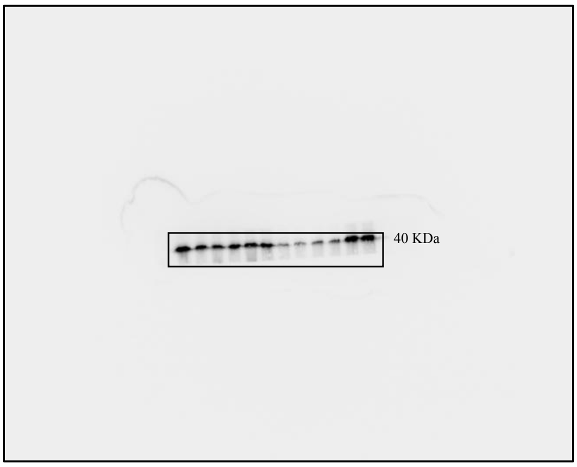

STING

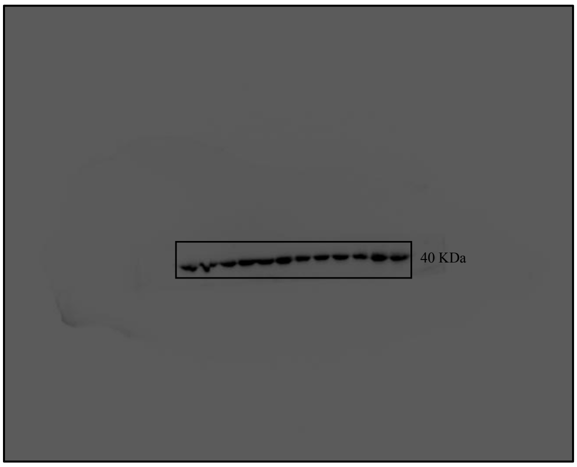

γH2A

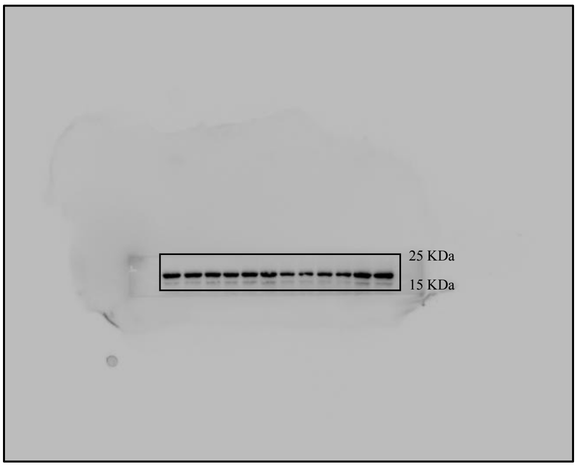

GAPDH

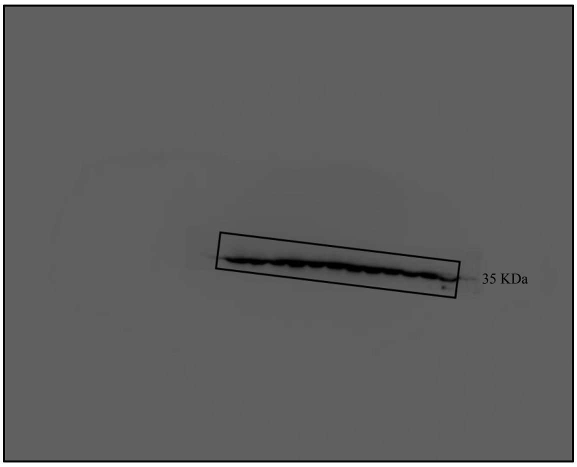

Supplement: Unedited blot and gel images [file jci-134-165140-s018.pdf]
